# Effective Dipole Moment Model for Axially Symmetric *C*_3*v*_ Molecules: Application to the Precise Study of Absolute Line Strengths of the *ν*_6_ Fundamental of CH_3_^35^Cl

**DOI:** 10.3390/ijms241512122

**Published:** 2023-07-28

**Authors:** Oleg Ulenikov, Elena Bekhtereva, Olga Gromova, Anna Fomchenko, Yulia Morzhikova, Sergei Sidko, Christian Sydow, Sigurd Bauerecker

**Affiliations:** 1 Research School of High-Energy Physics, National Research Tomsk Polytechnic University, 634050 Tomsk, Russia; bextereva@tpu.ru (E.B.); olgerda@tpu.ru (O.G.); fomchenko@tpu.ru (A.F.); morzhikova@tpu.ru (Y.M.); sss60@tpu.ru (S.S.); 2 Institut für Physikalische und Theoretische Chemie, Technische Universität Braunschweig, D-38106 Braunschweig, Germany; christian.sydow@tu-braunschweig.de (C.S.); s.bauerecker@tu-bs.de (S.B.)

**Keywords:** CH_3_^35^Cl, effective rotational and effective dipole moment operators, absolute line positions and strengths, *C*_3*v*_-symmetry molecule

## Abstract

The effective dipole moment model for molecules of axial C${}_{3v}$ symmetry is derived on the basis of the symmetry properties of a molecule which, on the one hand, is of the same order of efficiency (but much simpler and clearer in applications) as the analogous models derived on the basis of the irreducible tensorial sets theory, and, on the other hand, mathematically more correct in comparison with concepts like the Herman–Walles function used in the models. As an application of the general results obtained, we discuss high-resolution infrared spectra of CH${}_{3}{}^{35}$Cl, recorded with the Zürich prototype ZP2001 (Bruker IFS125 HR) Fourier transform infrared spectrometer at a resolution of 0.001 cm${}^{-1}$ and analyzed in the region of 880–1190 cm${}^{-1}$ ($\nu_{6}$ bending fundamental centered at $\nu_{0}$ = 1018.070790 cm${}^{-1}$). Absolute strengths of more than 2800 transitions (2081 lines) were obtained from the fit of their shapes both with Voigt and Hartmann–Tran profiles, and parameters of the effective dipole moment of the $\nu_{6}$ band were determined by the computer code SYMTOMLIST (SYMmetric TOp Molecules: LIne STrengths), created on the basis of a derived theoretical model. As the first step of the analysis of the experimental data, assignments of the recorded lines were made. A total of 5124 transitions with $J^{\max}$ = 68, $K^{\max}$ = 21 were assigned to the $\nu_{6}$ band. The weighted fit of 2077 upper energy values obtained from the experimentally recorded transitions was made with a Hamiltonian which takes into account different types of ro–vibrational effects in doubly degenerate vibrational states of the $C_{3v}$-symmetric molecule. As the result, a set of 25 fitted parameters was obtained which reproduces the initial 2077 upper “experimental” ro–vibrational energy values with a root mean square deviation $d_{\mathrm{rms}}=4.7\times 10^{-5}$ cm${}^{-1}$. At the second step of the analysis, the computer code SYMTOMLIST was used for determination of the parameters of the derived effective dipole moment model. Six effective dipole moment parameters were obtained from the weighted fit procedure which reproduces absolute experimental strengths of the 2804 initial experimental transitions with a relative $d_{\mathrm{rms}}=3.4\%$.

## 1. Introduction

Correct description of absolute line strengths is one of the most important problems of molecular physics and chemistry both because of a number of applications in astrophysics and astrochemistry, study of planetary atmospheres, numerous industrial problems, and because of numerous pure academical scientific problems, such as, e.g., as determination of intramolecular multidimensional dipole moment hypersurface, unimolecular reaction rate theory, fundamental biomolecular reaction dynamics, etc. In turn, correct description of absolute line strengths of molecules is based on two points: the first is the ability of the modern equipment which allows us now to achieve accuracies in line strengths determination in parts of percent for simple molecules (or, 1–3% for molecules with 4–6 nuclei). The second point is a theoretical model which is used for description of experimental data. We do not speak here about the general theory, but will only mention that the modern molecular physics (based on the peculiarities and symmetry properties of concrete types of polyatomic molecules) uses different modifications of methods and approaches which are mostly efficiently adapted to description of absolute line strengths of molecules of different types of symmetry. As an illustration, we mention the fundamental results which are presented in Ref. [1] and widely used by the modern spectroscopic community for description of line strengths of asymmetric top molecules (molecules with nondegenerate vibrational modes). Another type of molecules (namely, spherical top molecules) in the modern molecular physics are described on the basis of results (and corresponding computer codes) which are derived from the irreducible tensorial sets theory, which is efficient for applications in high-symmetry molecules (see, e.g., [2,3]). Another very important class of molecules is that of symmetric top molecules, which occupy an intermediate position between asymmetric top and spherical top molecules. Probably just because of their intermediate position, in the modern molecular physics/chemistry, two methods are used. The first variant is the use of the irreducible tensorial sets theory (analogously to the spherical top molecules) for deriving the corresponding model (see, e.g., [4,5]). An alternative model looks rather phenomenological, which in reality is an extension of the Herman–Walles function of diatomic and linear molecules to symmetric top molecules (see, e.g., [6]). In our opinion, both of these models are not very promising for applications. As a consequence, we made an attempt in this study to develop a model of the effective dipole moment of the $C_{3v}$ symmetric top molecule which, on the one hand, would be more powerful (from the mathematical point of view) than the model which uses the Herman–Walles function but, on the other hand, would be easier to handle and simpler in practical implementation in comparison with the models of the Ref. [4] type. An example of the efficiency of the obtained results is illustrated by their application to the analysis of the absolute line strengths of the $\nu_{6}$ band of the methyl chloride molecule (see Section 3.4 and Section 3.6).

Methyl chloride (CH${}_{3}$Cl) is a colorless, flammable, toxic gas that was used widely as a refrigerant and has many current industrial applications, including its use as a local anesthetic, a chemical intermediate in silicone polymer production and drug manufacturing, an extractant for oils and resins, a solvent in butyl rubber and petroleum refining, a propellant in polystyrene foam production, a methylating and chlorinating agent in organic chemistry, and an herbicide. Exposure to methyl chloride can cause a wide variety of issues from frostbite, drowsiness, and dizziness to paralysis, seizures, and coma, depending on the route and level (concentration and duration) of exposure. Methyl chloride is particularly important in the global atmosphere as a major natural source of chlorine to the stratosphere [7], a compound involved in the destruction of the ozone layer [8,9,10,11] (methyl chloride, with concentrations between 500 and 1000 parts per trillion (ppt) [12,13], is the most abundant halocarbon in the Earth’s atmosphere, representing 30% of the total chlorine content). The production of methyl chloride is dominated by natural sources, but, to a lesser extent, also by anthropogenic sources, such as agricultural fumigation and/or biomass burning [14]. As an absorber of infrared radiation, methyl chloride is of interest for its potential effect on the tropospheric energy balance and greenhouse heating as well as for chemical interactions [15,16]. Particular interest in the study of methyl chloride has arisen in recent years as one of the chlor–organic compounds that have a negative impact on the processes of industrial transportation and processing of petroleum products [17]. For all of these reasons, many laboratory spectroscopic studies of methyl chloride and its isotopologues have been fulfilled during the preceding years (see [18,19,20,21,22,23,24,25,26,27,28,29,30,31,32,33,34,35,36,37,38,39,40,41,42,43,44,45,46,47,48]). There are also some theoretical studies where potential energy surfaces, computations of vibrational energy levels, and some other aspects of the CH${}_{3}$Cl ro–vibrational problems have been discussed [49,50,51,52,53,54,55,56].

This paper is constructed as follows. In Section 2, details of our experiment are discussed. Because line position analysis is an essential part of the precise line strengths analysis, we first of all made such line position analysis, obtained highly accurate data of the upper ro–vibrational energy values, determined spectroscopic parameters of the $\left(v_{6}=1\right)$ vibrational state, and derived accurate values of corresponding ro–vibrational wave functions which are necessary for the line position analysis. All of this work is discussed in Section 3.1, Section 3.2 and Section 3.3. Experimental aspects of the line strength analysis (in particular, determination of absolute line strengths on the basis of their shapes fit with the Hartmann–Tran profile function) are discussed in Section 3.4. In Section 3.5, we discuss problems which are connected with the deriving of the new modification of the effective dipole moment operator. The general results of Section 3.5 are used in Section 3.6 for the absolute line strength analysis of the $\nu_{6}$ band of CH${}_{3}$Cl.

## 2. Methods and Materials

In the spectral region of 500–5000 cm${}^{-1}$, two spectra of CH${}_{3}$Cl were recorded in the infrared laboratory of the Technische Universität Braunschweig with the Bruker IFS125HR Fourier transform Zürich prototype infrared spectrometer [57]. The sample of chloromethane, also called methyl chloride or refrigerant-40 (R40), was purchased by Air Liquide and has a purity of 99.8%. The experimental conditions are summarized in Table 1. For the measurements, a multiple-path White cell, made from stainless steel, was applied. For all spectra, a globar radiation source together with a KBr beamsplitter and a liquid-nitrogen-cooled MCT detector were used at an optical resolution of 0.0025 and 0.003 cm${}^{-1}$ adding 1000 and 1860 scans for the two spectra to improve the signal/noise ratio which increases with the square root of the scan number. The sample gas pressure was 50 and 300 Pa, the optical path length 4 ± 0.002 m and 24 ± 0.012 m, and the aperture 1.5, and 1.0 mm. The total line width is between 0.0019 and 0.009 cm${}^{-1}$ and can be approximated by the root sum square of the convolution of the three line widths conferring to Doppler, pressure, and instrumental line widths. Doppler line width is between 0.0024 and 0.0087 cm${}^{-1}$, pressure line width is 0.00014 and 0.00084 cm${}^{-1}$, and instrumental line width is 0.0017 and 0.0020 cm${}^{-1}$, being the product of nominal instrumental resolution of 0.0025 (0.003) cm${}^{-1}$ and Boxcar apodization factor of 0.68. The total line width is Doppler-dominated, as the pressure line width is almost negligible. By use of the Beer–Lambert–Bouguer law, the line strength *S* can be derived from the area of a single absorption line $A_{\mathrm{Line}}$, the (partial) pressure *P* of the gaseous species (CH${}_{3}$Cl), the temperature *T*, and the optical path length *L* (compare line strength analysis in following sections):

(1) $$\begin{array}{c} S=\frac{k_{B}T}{PL}A_{\mathrm{Line}}, \end{array}$$

using the decadic logarithm $\mathrm{lg}=\log_{10}$:

(2) $$\begin{array}{c} A_{\mathrm{Line}}=\frac{1}{\mathrm{lg}\left(e\right)}\int \mathrm{lg}\left(\frac{I_{0}\left(\nu \right)}{I(\nu )}\right)d\nu . \end{array}$$

The line intensities were obtained by direct integration of the measured effective line absorbance, which can be fitted well by a Voigt or Hartmann–Tran line profile. Further, it was assumed that the ideal gas law is applicable for sample pressures below 200 hPa. As the line area $A_{\mathrm{Line}}$ is taken from single lines out of the absorbance spectrum, the line absorbance should preferably lie in the range between 0.2 and 0.7 in the experimental spectra to minimize noise on the one hand and avoid saturation effects on the other.

For the present spectra, we used in situ calibration with H${}_{2}$O and CO${}_{2}$ (from HITRAN) molecules which were included in the gas sample and spectrometer in traces. Three freeze–pump–thaw cycles of the gas sample were performed directly before the measurements of each spectrum to minimize foreign gas concentrations in the sample. The temperature was monitored with an Ahlborn Almemo 2590 thermometer using a PT100 resistance and could be kept within a temperature interval smaller than ±0.3 K for both spectra. As for line integral and intensity determination, precise pressure monitoring is crucial; we used temperature compensating CMR capacitance sensor gauges from Pfeiffer AG in ceramic technology in a cascade with pressure ranges up to 10 and 100 hPa. These factory-calibrated sensors are independent of the gas species and are resistant against aggressive gaseous media. The manufacturer claims an accuracy on the measured value of 0.2%. We estimate the total error of the sample pressure during measurement to be in the range of ±0.5%, which results in 50±0.25 Pa and 300±1.5 Pa for the present experiments. For optimizing the spectra recording, line calibration, line strength, and broadening analyses, we applied data and procedures described in Refs. [57,58,59].

## 3. Results and Discussion

### 3.1. General Information and Assignment of Transitions

**Figure 1:**
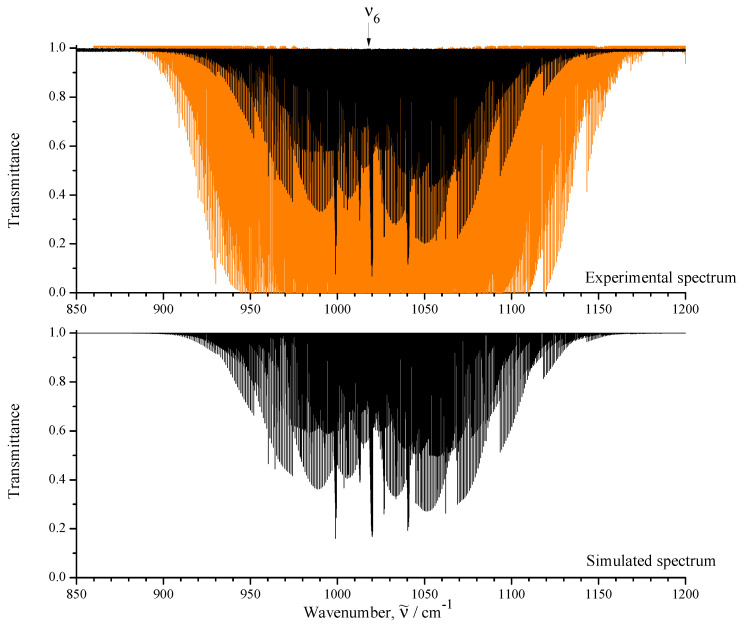
**Upper trace**: overview of the experimental spectra I (black) and II (orange) of CH${}_{3}$Cl in the region of the $\nu_{6}$ band (for the experimental conditions, see Table 1 and Section 2). **Lower trace**: simulated spectrum I.

**Figure 2:**
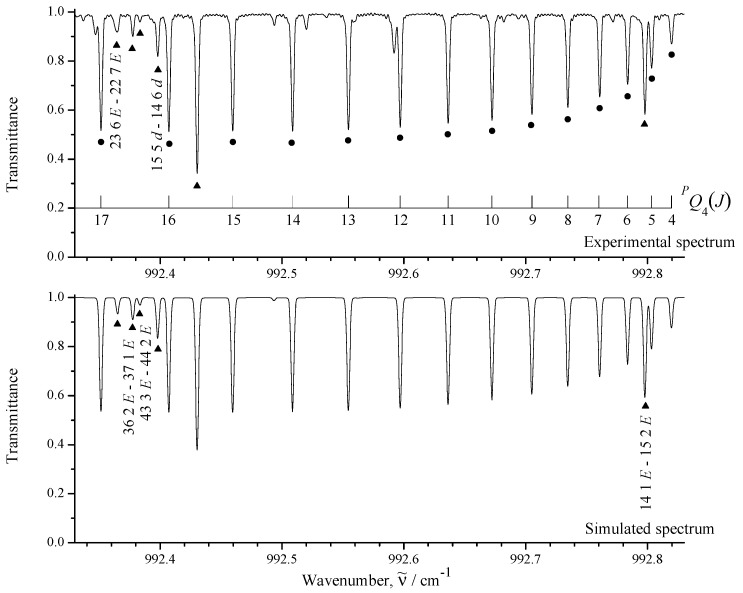
Small part of the high-resolution spectrum I in the region of the Q—branch of CH${}_{3}{}^{35}$Cl. The ${}^{P}Q_{4}\left(J\right)$ transitions are marked by dark circles. Dark triangles denote other transitions (not belonging to the ${}^{P}Q_{4}\left(J\right)$ set). Unmarked lines belong probably to the CH${}_{3}{}^{37}$Cl $\nu_{6}$ band transitions.

**Figure 3:**
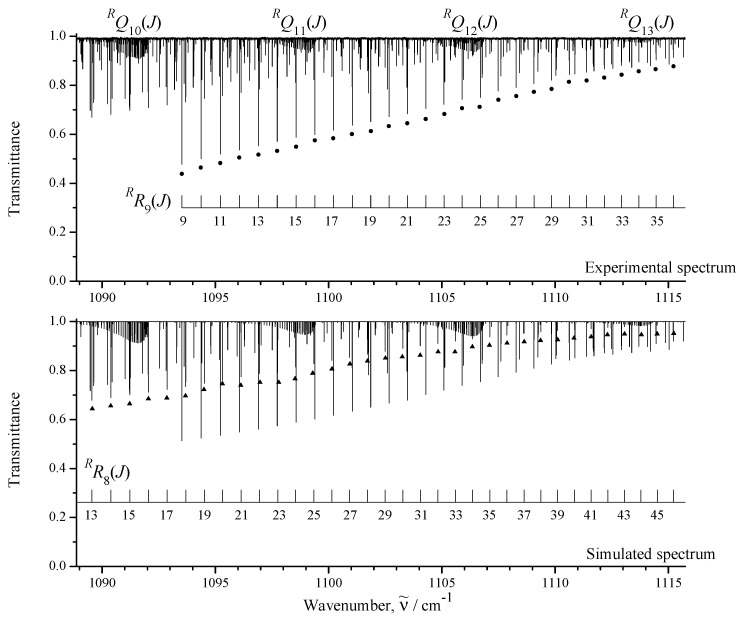
Part of the high-resolution spectrum I in the region of the R—branch of CH${}_{3}{}^{35}$Cl. Two sets of transitions (${}^{R}R_{9}\left(J\right)$ marked by dark circles, and ${}^{R}R_{8}\left(J\right)$ marked by dark triangles) are shown. Some sets of the ${}^{R}Q_{K}\left(J\right)$ clusters are also seen.

**Figure 4:**
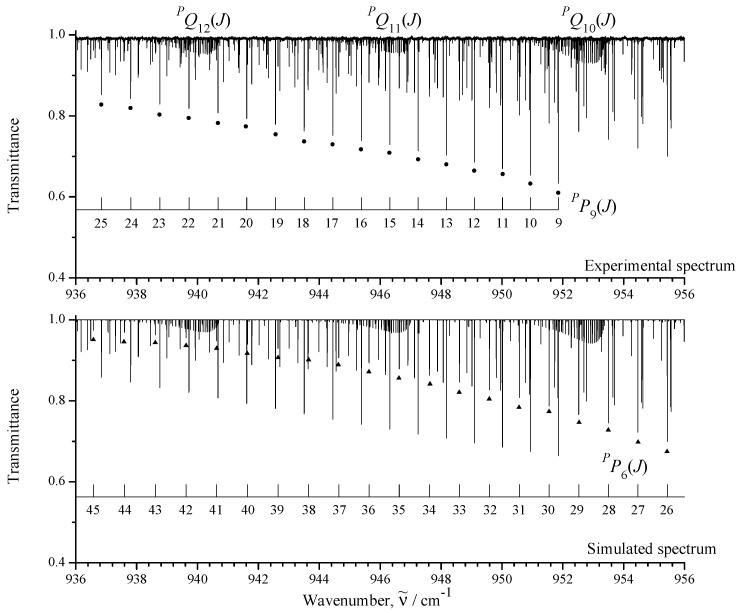
Detail of the infrared spectrum I of the $\nu_{6}$ band of CH${}_{3}{}^{35}$Cl showing sets of ${}^{P}P_{9}\left(J\right)$ (dark circles) and ${}^{P}P_{6}\left(J\right)$ (dark triangles) transitions. Three Q-type clusters are also indicated.

The upper part of Figure 1 shows an overview of the IR spectra I (black) and II (orange) of CH${}_{3}$Cl in the region of 850–1200 cm${}^{-1}$ where the $\nu_{6}$ fundamental is located. To illustrate the quality of the recorded experimental data, some small portions of the high-resolution spectrum I are shown in Figure 2, Figure 3 and Figure 4.

CH${}_{3}$Cl is a symmetric top with an equilibrium structure of $C_{3v}$ point group symmetry. As a consequence, vibrational states of CH${}_{3}$Cl have $A_{1}$, $A_{2}$, or *E* symmetry. The symmetry allowed $\nu_{6}$ fundamental transitions considered in the present study is a “perpendicular” ($E\leftarrow A_{1}$-type) band with the selection rules for “allowed” transitions [60,61,62]:

(3) $$\begin{array}{c} \Delta J=0,\pm 1\;\;\mathrm{and}\;\;\Delta K=\pm 1. \end{array}$$

In addition to “allowed” transitions, the so-called “forbidden” transitions, which are considerably weaker in comparison with “allowed” ones, can be seen in absorption. Selection rules for “forbidden” transitions do not have the limitation for ΔK (the ΔK-value for “forbidden” transition can be arbitrary in principle) and they are

(4) $$\begin{array}{c} \Delta J=0,\pm 1\;\;\mathrm{and}\;\;a_{1}↔a_{2},\;\;e\leftarrow e, \end{array}$$

The assignment of transitions was carried out by means of the ground state combination differences (GSCD) method, as used and discussed by the authors of [63,64,65,66,67,68,69] in the analysis of various polyatomic molecules. Ground state rotational energies, which are necessary for the analysis, were calculated with the parameters from Ref. [43]. As a result of the analysis, 5124 transitions with the values of quantum numbers $J^{\max}$ = 68 and $K^{\max}$ = 21 were assigned to the $\nu_{6}$ band of ${}^{12}$CH${}_{3}$${}^{35}$Cl (see also the statistical information given in Table 2). The list of assigned transitions is presented as Supplementary Data S1 to this work (see columns 1–3 of Supplementary Data S1). Some exemplary small parts of Supplementary Data S1 are shown in Table 3 for illustration.

**Table 2:** Statistical information for the $\nu_{6}$ band of CH${}_{3}{}^{35}$Cl.

Band	Center/cm${}^{-1}$	$J^{\max}$	$K_{a}^{\max}$	$N_{\mathrm{tr}}$ ${}^{\left(a\right)}$	$N_{l}$ ${}^{\left(b\right)}$	$m_{1}$ ${}^{\left(c\right)}$	$m_{2}$ ${}^{\left(c\right)}$	$m_{3}$ ${}^{\left(c\right)}$
$\nu_{6}$	1118.070790	68	21	5124	2077	88.9	8.8	2.3
$d_{\mathrm{rms}}$ ${}^{\left(d\right)}$	$4.7\times 10^{-5}$							

${}^{\left(a\right)}$$N_{tr}$ is the number of assigned transitions. ${}^{\left(b\right)}$$N_{l}$ is the number of obtained upper-state energies. ${}^{\left(c\right)}$ Here, $m_{i}$ = $n_{i}/N_{l}\;\times$ 100% (*i* = 1, 2, 3); $n_{1}$, $n_{2}$, and $n_{3}$ are the numbers of upper-state energies for which the differences δ = $E^{\exp}-E^{\mathrm{calc}}$ satisfy the conditions δ≤0.5× 10${}^{-4}$ cm${}^{-1}$, 0.5 ×10${}^{-4}$ cm${}^{-1}$<δ≤1× 10${}^{-4}$ cm${}^{-1}$, and $\delta >1\times 10^{-4}$ cm${}^{-1}$. ${}^{\left(d\right)}$ in cm${}^{-1}$.

**Table 3:** Small part of the list of transitions assigned to the $\nu_{6}$ band of CH${}_{3}{}^{35}$Cl.

*J*	*K*	Γ	$J^{\prime }$	$K^{\prime }$	$\Gamma^{\prime }$	ν ${}^{\left(a\right)}$	$\delta_{\nu }$ ${}^{\left(b\right)}$	$S_{\nu }^{\exp}$(294.45) ${}^{\left(c\right)}$	$\Delta_{S}$ ${}^{\left(d\right)}$	$S_{\nu }^{\mathrm{calc}}$(294.45) ${}^{\left(e\right)}$	$\delta_{S}$ ${}^{\left(f\right)}$	$\Gamma_{0}$ ${}^{\left(g\right)}$	$\Delta_{2}$ ${}^{\left(g\right)}$	$\nu_{\mathrm{VC}}$ ${}^{\left(g\right)}$	*R* ${}^{\left(h\right)}$
**1**			**2**			**3**	**4**	**5**	**6**	**7**	**8**	**9**	**10**	**11**	**12**
5	1	*E*	4	2	*E*	1010.81799	5	0.231768 $\times \;10^{-2}$	0.5	0.2321 $\times \;10^{-2}$	−0.15	2.987 $\times \;10^{-4}$	−0.224	−0.422	1.3
13	2	$A_{2}$	12	3	$A_{1}$	1010.83845	1	0.108382 $\times \;10^{-1}$	0.2	0.5389 $\times \;10^{-2}$	0.56	3.525 $\times \;10^{-4}$	−0.150	−0.449	1.7
13	2	$A_{1}$	12	3	$A_{2}$	1010.83845	−1			0.5389 $\times \;10^{-2}$					
38	0	*E*	38	1	*E*	1010.88494	0	0.320599 $\times \;10^{-2}$	3.9	0.2939 $\times \;10^{-2}$	8.32	2.815 $\times \;10^{-4}$			1.0
37	0	*E*	37	1	*E*	1011.00984	0	0.366556 $\times \;10^{-2}$	0.4	0.3401 $\times \;10^{-2}$	7.22	3.206 $\times \;10^{-4}$			1.0
36	0	*E*	36	1	*E*	1011.13137	−1	0.422301 $\times \;10^{-2}$	0.6	0.3915 $\times \;10^{-2}$	7.30	2.999 $\times \;10^{-4}$	0.136		1.1
30	4	*E*	29	5	*E*	1011.15162	8	0.206013 $\times \;10^{-2}$	1.0	0.1975 $\times \;10^{-2}$	4.11	2.808 $\times \;10^{-4}$			1.0
24	3	*E*	25	2	*E*	1011.15634	−2	0.499552 $\times \;10^{-2}$	0.4	0.5107 $\times \;10^{-2}$	−2.23	3.976 $\times \;10^{-4}$			1.0
9	1	$A_{2}$	10	0	$A_{1}$	1011.20573	1	0.157995 $\times \;10^{-1}$	0.2	0.1596 $\times \;10^{-1}$	−0.99	4.175 $\times \;10^{-4}$			1.0
35	0	*E*	35	1	*E*	1011.24956	−3	0.476969 $\times \;10^{-2}$	0.3	0.4482 $\times \;10^{-2}$	6.03	2.332 $\times \;10^{-4}$	−0.750 $\times \;10^{-1}$	−0.315	1.2
48	6	*E*	47	7	*E*	1011.30392	−59			0.8773 $\times \;10^{-4}$					
39	5	$A_{2}$	38	6	$A_{1}$	1011.36055	12	0.109227 $\times \;10^{-2}$	0.4	0.5266 $\times \;10^{-3}$	3.57	2.970 $\times \;10^{-4}$			1.0
39	5	$A_{1}$	38	6	$A_{2}$	1011.36055	12			0.5266 $\times \;10^{-3}$					
34	0	*E*	34	1	*E*	1011.36443	−2	0.549711 $\times \;10^{-2}$	0.4	0.5104 $\times \;10^{-2}$	7.14	2.970 $\times \;10^{-4}$			1.0
31	4	$A_{2}$	32	3	$A_{1}$	1011.44531	−6	0.464102 $\times \;10^{-2}$	0.6	0.2429 $\times \;10^{-2}$	−4.69	3.126 $\times \;10^{-4}$	−0.159		1.1
31	4	$A_{1}$	32	3	$A_{2}$	1011.44531	−6			0.2429 $\times \;10^{-2}$					
33	0	*E*	33	1	*E*	1011.47598	0	0.614718 $\times \;10^{-2}$	0.2	0.5782 $\times \;10^{-2}$	5.94	2.449 $\times \;10^{-4}$	−0.129	−0.317	2.0
22	3	*E*	21	4	*E*	1011.50323	0	0.426391 $\times \;10^{-2}$	0.6	0.4292 $\times \;10^{-2}$	−0.67	4.391 $\times \;10^{-4}$			1.0
1	0	*E*	2	1	*E*	1011.53211	−1	0.336528 $\times \;10^{-2}$	0.5	0.3237 $\times \;10^{-2}$	3.82	4.895 $\times \;10^{-4}$			1.0
32	0	*E*	32	1	*E*	1011.58418	−1	0.703075 $\times \;10^{-2}$	0.4	0.6514 $\times \;10^{-2}$	7.35	3.079 $\times \;10^{-4}$			1.0
38	5	*E*	39	4	*E*	1011.64991	−9	0.916940 $\times \;10^{-3}$	2.7	0.8656 $\times \;10^{-3}$	5.60	2.000 $\times \;10^{-4}$			1.0
14	2	$A_{2}$	13	3	$A_{1}$	1011.67808	0	0.117754 $\times \;10^{-1}$	0.3	0.5676 $\times \;10^{-2}$	3.60	3.914 $\times \;10^{-4}$			1.0
14	2	$A_{1}$	13	3	$A_{2}$	1011.67808	2			0.5676 $\times \;10^{-2}$					
6	1	*E*	5	2	*E*	1011.68498	7	0.323333 $\times \;10^{-2}$	0.5	0.3157 $\times \;10^{-2}$	2.37	3.697 $\times \;10^{-4}$			1.0
31	0	*E*	31	1	*E*	1011.68904	−2	0.781218 $\times \;10^{-2}$	0.5	0.7299 $\times \;10^{-2}$	6.57	3.697 $\times \;10^{-4}$			1.0
16	2	*E*	17	1	*E*	1011.71876	−1	0.789033 $\times \;10^{-2}$	0.3	0.7925 $\times \;10^{-2}$	−0.44	4.412 $\times \;10^{-4}$	−0.549 $\times \;10^{-1}$	−0.266	1.1
45	6	*E*	46	5	*E*	1011.77417	24			0.2339 $\times \;10^{-3}$					
30	0	*E*	30	1	*E*	1011.79061	−1	0.854407 $\times \;10^{-2}$	0.5	0.8133 $\times \;10^{-2}$	4.81	3.123 $\times \;10^{-4}$			1.0
29	0	*E*	29	1	*E*	1011.88885	−1	0.938728 $\times \;10^{-2}$	0.5	0.9012 $\times \;10^{-2}$	4.00	3.487 $\times \;10^{-4}$			1.0

${}^{\left(a\right)}$ Experimental line position (in cm${}^{-1}$). ${}^{\left(b\right)}$ Differences $\delta_{\nu }$ between the experimental and calculated line position (in 10${}^{-5}$cm${}^{-1}$). ${}^{\left(c\right)}$ Line intensity determined from the fit of experimental line shape with a Hartmann–Tran (qSDRP) profile (in cm${}^{-2}$/atm${}^{-1}$). ${}^{\left(d\right)}$ Experimental error $\Delta_{S}$ (in percent) in the line intensity determination. ${}^{\left(e\right)}$ Calculated line intensity with parameters from Table 4 (in cm${}^{-2}$/atm${}^{-1}$). ${}^{\left(f\right)}$ Differences $\delta_{S}$ (in percent) between the experimental line strengths and such calculated with the parameters from Table 4. ${}^{\left(g\right)}$ Parameter obtained from the fit of the experimental line shape with the Hartmann–Tran (qSDRP) profile. Value is absent in the column when corresponding parameter is insufficient for the fit. In this case, such parameter was taken as zero. ${}^{\left(h\right)}$ Here, $R=d_{\mathrm{rms}}$(qSDVP)/$d_{\mathrm{rms}}$(qSDRP); $d_{\mathrm{rms}}$(qSDVP) and $d_{\mathrm{rms}}$(qSDRP) are obtained from the line shape fit.

**Table 4:** Effective dipole moment parameters of the $\nu_{6}$ band of CH${}_{3}{}^{35}$Cl (in Debye).

Operator	Parameter	Value
$R_{\sigma }^{E(0,A_{1})E}=k_{\sigma }^{E}$	$P^{E(0,A_{1})}\times 10$	0.55712(72)
$R_{\sigma }^{E(2,1A_{1})E}$	$P^{E(2,1A_{1})}\times 10^{3}$	0.1466(89)
$R_{\sigma }^{A_{2}\left(2,1E\right)E}$	$P^{A_{2}\left(2,1E\right)}\times 10^{3}$	−0.493(54)
$R_{\sigma }^{E(4,3A_{1})E}$	$P^{E(4,3A_{1})}\times 10^{5}$	−0.2249(25)
$R_{\sigma }^{A_{2}\left(4,1E\right)E}$	$P^{A_{2}\left(4,1E\right)}\times 10^{5}$	−0.3378(49)
$R_{\sigma }^{A_{2}\left(4,2E\right)E}$	$P^{A_{2}\left(4,2E\right)}\times 10^{5}$	−1.024(62)

### 3.2. Theoretical Background for the Effective Hamiltonian Used

The mathematical model of a symmetric top molecule which was discussed earlier in [70] and successfully applied by the authors to the study of high-resolution spectra of different symmetric top molecules (including AsH${}_{3}$ and AsD${}_{3}$ [71,72,73], PH${}_{3}$ and PD${}_{3}$ [74,75,76], NF${}_{3}$ [77], CH${}_{3}$D and CHD${}_{3}$ [78,79,80,81], NaBH${}_{4}$ [82], CHF${}_{3}$ [83,84]) is applied in the present study to the analysis of the ro–vibrational structure of the $\nu_{6}$ band of CH${}_{3}$Cl. This model uses the symmetry properties of a molecule (applying theorems and results of the theory of irreducible tensorial sets [85,86,87,88,89,90,91,92,93]) for the construction of the molecular Hamiltonian and of different matrix elements as adapted to the $C_{3v}$-type molecules. Having no possibility to discuss here the “effective Hamiltonian” theory in detail, we (to give the reader an impression about the problem) very briefly present the main aspects.

#### 3.2.1. Effective Rotational–Vibrational Hamiltonian

The use of the “effective Hamiltonians” model is one of the most efficient ways to solve the stationary Schrödinger equation

(5) $$\begin{array}{c} H\psi_{\alpha }\left(x,y\right)=E_{\alpha }\psi_{\alpha }\left(x,y\right) \end{array}$$

for a quantum mechanical system (such as, for example, a polyatomic molecule) which (1) depends on, at least, two different types of variables (sets of coordinates “*x*” and “*y*”; as an illustration, they can be vibrational and angular coordinates in vibrational–rotational problems; or coordinates electrons and nuclei in electron–nuclear problems) and (2) for which an influence of the second type variables “*y*” can be considered as a considerably smaller effect in comparison with the influence of the first type “*x*” variables. As a consequence, the Hamiltonian of a system can be presented in the following form:

(6) $$\begin{array}{c} H=H_{0}\left(x\right)+h\left(x,y\right)\equiv H_{0}\left(x\right)+\sum_{n=1}h^{\left(n\right)}\left(x,y\right), \end{array}$$

where $H_{0}\left(x\right)\gg h\left(x,y\right)$, and solutions of the Schrödinger equation with the zero-order operator $H_{0}\left(x\right)$

(7) $$\begin{array}{c} H_{0}\left(x\right)|i\left(x\right)\rangle =E_{i}^{0}|i\left(x\right)\rangle \end{array}$$

are known (in Equation (6), $h^{\left(n\right)}\left(x,y\right)$ denotes a “small” operator of the order *n* in comparison with the zero-order operator $H_{0}\left(x\right)$).

The problem (at the first view) does not differ from the corresponding traditional problems of quantum mechanics, which is usually solved by methods of perturbation theory [94]. There is, however, a very significant difference between the traditional quantum mechanical problems and the solution of the Schrödinger equation with a Hamiltonian in the form of Equation (6). It is connected with the fact that the “mall” perturbation operator “h(x)” in the traditional quantum mechanical problem depends on the same variables as the zero approximation operator “$H_{0}\left(x\right)$”, and, therefore, corrections to the energies of the zeroth approximation from the perturbation operator are obtained in the form of numbers. At the same time, the formal application of the traditional perturbation theory to the Hamiltonian (6) leads to the fact that the resulting corrections are obtained in the form not of numbers, but of operators depending on variables of the “*y*” type (and not only on the “*y*” coordinates themselves, but also from their derivatives “∂/∂y”, which generally do not commute with each other).

To solve such kind of problem, the Van Vleck transformation [95] has been applied in molecular physics for many years, and uses the well-known statement of quantum mechanics that any two Hermitian operators *H* and $\tilde{H}$, which are connected with each other by an unitary transformation

(8) $$\begin{array}{c} \tilde{H}=G^{+}HG \end{array}$$

(*G* is an arbitrary unitary operator), have absolutely identical sets of eigenvalues $E_{\alpha }$ and ${\tilde{E}}_{\alpha }$ and are connected with each other’s sets of eigenfunctions $\psi_{\alpha }\left(x,y\right)$ and ${\tilde{\psi }}_{\alpha }\left(x,y\right)$ (${\tilde{E}}_{\alpha }$ and ${\tilde{\psi }}_{\alpha }\left(x,y\right)$ are the eigenvalues and eigenfunctions of the Hamiltonian $\tilde{H}$):

(9) $$\begin{array}{c} \tilde{H}{\tilde{\psi }}_{\alpha }\left(x,y\right)={\tilde{E}}_{\alpha }{\tilde{\psi }}_{\alpha }\left(x,y\right). \end{array}$$

The main idea of transformation, Equation (8), is to replace a Schrödinger equation with a complicated Hamiltonian *H* by the Schrödinger equation with a considerably more simple one, $\tilde{H}$. The possibility of such a simplification is based on the fact that the above is true when using any unitary operator G(x,y), and it is only necessary to find among the infinite number of unitary operators one that will allow one to make the necessary simplification of the original Hamiltonian *H*. This approach is known in the vibrational–rotational theory of polyatomic molecules as the “operator perturbation theory”, which is used both in the form of the “contact transformations” [96,97,98,99,100], and in the form of the “matrix formulation of operator perturbation theory” [101,102,103] (the latter is especially efficient and simple for its implementation on a computer in the form of “systems for analytical calculations”). The result of this approach is the so-called “effective rotational operators” and “effective dipole moment operators”, which allow one to solve the problems of determining the rotational structure of a separate vibrational state and/or absolute line strengths in a separate vibrational–rotational band of a molecule with high accuracy.

The possibility of implementing the operator perturbation theory is based on the fact that (according to Equation (6)) the part of the initial Hamiltonian *H*, which depends on the coordinates of the “*y*”-type, is a small operator compared to the $H_{0}$ one. As a result, the operator *G* in Equation (8) can be used in the form

(10) $$\begin{array}{c} G=\exp\left(i\sum_{n}^{\infty }g_{n}\left(x,y\right)\right)=1+\left(\sum_{n}^{\infty }ig_{n}\left(x,y\right)\right)+\frac{1}{2}{\left(\sum_{n}^{\infty }ig_{n}\left(x,y\right)\right)}^{2}+\ldots , \end{array}$$

where $i=\sqrt{-1}$. Being substituted in Equation (8), the operator *G*, Equation (10), leads to the following relations (if one equates the terms of the same order in the right and left parts of the obtained result):

(11) $$\begin{array}{c} {\tilde{H}}_{0}\;\equiv H_{0}, \end{array}$$

(12) $$\begin{array}{c} {\tilde{h}}^{\left(1\right)}=h^{\left(1\right)}+\left(H_{0}ig_{1}-ig_{1}H_{0}\right), \end{array}$$

(13) $$\begin{array}{ccc} {\tilde{h}}^{\left(2\right)} & & =h^{\left(2\right)}+\left(h_{1}ig_{1}-ig_{1}h_{1}\right)+\left(H_{0}ig_{2}-ig_{2}H_{0}\right)\\ & & +\frac{1}{2}\left(H_{0}{\left(ig_{2}\right)}^{2}-2ig_{1}H_{0}ig_{1}+{\left(ig_{2}\right)}^{2}H_{0}+\ldots \right), \end{array}$$

etc. Equations (11)–(13), etc., can be considered as a set of equations for obtaining operators $g_{n}\left(n=1,2,\ldots \right)$ which would help to simplify a problem of determination of eigenvalues of the Hamiltonian, Equation (6). Here, we discuss, very briefly, a realization of this idea in the frame of the “matrix operator perturbation theory” and use one of the most general methods of quantum mechanics for solving a stationary Schrödinger equation, namely, the construction of a Hamiltonian matrix on a complete orthogonal set of basis functions in the space in which the Hamiltonian is defined. In the considered case of the vibrational–rotational problem, these are all possible combinations of functions $|v_{n}\rangle |Jkm\rangle =|v\rangle |r\rangle =|\varphi_{vr}\rangle$ (here, $|v_{n}\rangle$ are harmonic oscillator functions [61] and ∣Jkm〉 are spherical functions [104] which form complete orthogonal sets in the space of vibrational and rotational states, respectively).

Let us assume that the procedure of construction of the Hamiltonian matrix

(14) $$\begin{array}{c} \langle \varphi_{vr}|\tilde{H}|\varphi_{v^{\prime }r^{\prime }}\rangle =\langle r|\left(\langle v|\tilde{H}|v^{\prime }\rangle \right)|r^{\prime }\rangle \end{array}$$

is divided into two steps. At the first step, the matrix $\langle v|\tilde{H}|v^{\prime }\rangle$ is constructed, which has the form

(15) $$\begin{array}{c} \left(\begin{array}{ccccc} {\tilde{H}}_{11} & {\tilde{H}}_{12} & {\tilde{H}}_{13} & . & .\\ {\tilde{H}}_{21} & {\tilde{H}}_{22} & {\tilde{H}}_{23} & . & .\\ {\tilde{H}}_{31} & {\tilde{H}}_{32} & {\tilde{H}}_{33} & . & .\\ . & . & . & . & .\\ . & . & . & . & . \end{array}\right) \end{array}$$

(it is important that the matrix, Equation (15), is a matrix of the infinite dimension and each element ${\tilde{H}}_{nm}$ of this matrix is an operator dependent on the “*y*”-type variables). At the second step, for each element ${\tilde{H}}_{nm}$ of the matrix, Equation (15), it is necessary to construct a corresponding matrix on the basis of rotational functions ∣r〉. As a result, the matrix (already numeric) is obtained in the form of an infinite number of matrices of infinite dimension each. Evidently, it is impossible to diagonalize such a matrix. However, one can take advantage of the fact that each of the operator elements of the matrix, Equation (15), depends on an arbitrary yet unitary operator *G*, Equation (10) (or, in other words, is a function of a set of Hermitian operators $g_{n}$ satisfying the conditions (11)–(13), etc). Let us assume now that all operator matrix elements ${\tilde{H}}_{1n}\left(n\neq 1\right)$ and ${\tilde{H}}_{n1}\left(n\neq 1\right)$ in Equation (15) can be turned to zero. It means that the matrix, Equation (15), is transformed to the following matrix:

(16) $$\begin{array}{c} \left(\begin{array}{ccccc} {\tilde{H}}_{11} & 0 & 0 & 0 & .\\ 0 & {\tilde{H}}_{22} & {\tilde{H}}_{23} & . & .\\ 0 & {\tilde{H}}_{32} & {\tilde{H}}_{33} & . & .\\ 0 & . & . & . & .\\ . & . & . & . & . \end{array}\right), \end{array}$$

and, after construction of the final matrix on the rotational functions ∣r〉, the problem of determination of eigenvalues and eigenfunctions of the Hamiltonian, Equations (8) and (9) (or, finally, eigenvalues and eigenfunctions of the initial Hamiltonian, Equation (6)), is divided into two independent problems: (1) the first one is the determination of the rotational structure of the $|v_{1}\rangle$ vibrational state, and (2) the second one is the rotational structure of all the other vibrational states. In this case, if one is interested in the rotational structure of only one concrete $|v_{1}\rangle$ vibrational state, then consideration of the second problem can be omitted without a disadvantage to the result, but the first problem becomes already solvable. The resulting operator ${\tilde{H}}_{11}$ is called the “effective rotational Hamiltonian” of the $|v_{1}\rangle$ vibrational state. Obviously, if one is interested in a study of the rotational structure of another vibrational state, the effective rotational Hamiltonian of that other vibrational state can be determined similarly.

Having no possibility here to discuss in detail the procedure of constructing an effective rotational Hamiltonian, we note only a few important general points:

(1) The requirement ${\tilde{H}}_{1n}={\tilde{H}}_{n1}=0\left(n\neq 1\right)$ is equivalent to fulfilling the conditions (which follow from Equations (11)–(13), etc):
  (17) $$\begin{array}{c} \langle v_{1}|h_{1}|v_{n}\rangle +\left(E_{1}^{0}-E_{n}^{0}\right)\langle v_{1}|ig_{1}|v_{n}\rangle =0, \end{array}$$
  (18) $$\begin{array}{ccc} & & \langle v_{1}|h_{2}|v_{n}\rangle +\langle v_{1}|h_{1}ig_{1}-ig_{1}h_{1}|v_{n}\rangle +\left(E_{1}^{0}-E_{n}^{0}\right)\langle v_{1}|ig_{2}|v_{n}\rangle \\ & & +\frac{1}{2}\left(E_{1}^{0}+E_{n}^{0}\right)\langle v_{1}|{\left(ig_{1}\right)}^{2}|v_{n}\rangle -2\langle v_{1}|ig_{1}H_{0}ig_{1}|v_{n}\rangle =0, \end{array}$$
  etc. These relations can be considered as equations for determining operators ($ig_{n}$) (hence, ultimately, the unitary operator *G*, which transforms the operator matrix Equation (15) to the matrix in the form of Equation (16)).
(2) All the above said allows us to present the Hamiltonian $\tilde{H}$ in the following form:
  (19) $$\begin{array}{ccc} \tilde{H} & & =\sum_{n,m}|v_{n}\rangle \left(\langle v_{n}|\tilde{H}|v_{m}\rangle \right)\langle v_{m}|=\sum_{nm}|v_{n}\rangle {\tilde{H}}_{n,m}\langle v_{m}|\\ & & =|v_{1}\rangle {\tilde{H}}_{11}\langle v_{1}|+\sum_{n,m\neq 1}|v_{n}\rangle {\tilde{H}}_{nm}\langle v_{m}|. \end{array}$$
(3) As was discussed above, if one is interested in the rotational structure of the only one vibrational state $|v_{1}\rangle$, then the second term on the right side of Equation (19) is insignificant and can be omitted from the further consideration. As for the first term, it obviously has the form of a function of coordinates of the second “*y*”-type (for vibrational–rotational problems, they are the Euler angles φ,θ, and χ; in this case, the dependence of the effective Hamiltonian on the angular variables is manifested in the form of its dependence on the components $J_{x},J_{y}$, and $J_{z}$ of the angular momentum operator *J*.

An effective Hamiltonian $\tilde{H}$ can be derived from the same relations, Equations (11)–(13), etc., as the operators $\left(ig_{n}\right)$ in the following form:

(20) $$\begin{array}{c} {\tilde{H}}_{11}={\tilde{H}}_{11}^{\left(0\right)}+{\tilde{H}}_{11}^{\left(1\right)}+{\tilde{H}}_{11}^{\left(2\right)}+\ldots , \end{array}$$

where

(21) $$\begin{array}{c} {\tilde{H}}_{11}^{\left(0\right)}=H_{11}^{\left(0\right)}\equiv \langle v_{n}|H_{o}|v_{n}\rangle =E_{1}^{0}, \end{array}$$

(22) $$\begin{array}{c} {\tilde{H}}_{11}^{\left(1\right)}=\langle v_{n}|{\tilde{h}}^{\left(1\right)}|v_{n}\rangle =\langle v_{n}|h^{\left(1\right)}|v_{n}\rangle , \end{array}$$

(23) $$\begin{array}{ccc} {\tilde{H}}_{11}^{\left(2\right)}=\langle v_{n}|{\tilde{h}}^{\left(2\right)}|v_{n}\rangle & & =\langle v_{n}|h^{\left(2\right)}|v_{n}\rangle +\langle v_{n}|h^{\left(1\right)}ig_{1}-ig_{1}h^{\left(1\right)}|v_{n}\rangle +\\ & & E_{1}^{0}\langle 1|{\left(ig_{1}\right)}^{2}|n\rangle -2\langle v_{1}|ig_{1}H_{0}ig_{1}|v_{1}\rangle , \end{array}$$

etc.; the $\langle v_{1}|ig_{i}|v_{n}\rangle$ and $\langle v_{n}|ig_{i}|v_{1}\rangle$ operators are determined from the solution of the system of Equations (17) and (18), etc. It is easy to understand that the effective Hamiltonian, Equations (20)–(23), is obtained in this case in the form of a series expansion in terms of the powers of the operators $J_{\alpha }\left(\alpha =x,y,z\right)$:

(24) $$\begin{array}{c} {\tilde{H}}_{\left(11\right)}=\sum_{p+q+r=0}^{\infty }A_{pqr}\left(J_{x}^{p}J_{y}^{q}J_{z}^{r}+J_{z}^{r}J_{y}^{q}J_{x}^{p}\right). \end{array}$$

However, it should be noted that in the general case (especially when dealing with degenerate vibrational states), the above scheme is extremely cumbersome and complicated. Therefore, when solving practical problems related to the analysis of vibrational–rotational spectra of polyatomic molecules, the effective rotational operator is usually determined from the following considerations: the effective operator (being a rotational Hamiltonian in the form of Equation (24)) must be

(a) Hermitian;
(b) Totally symmetric (transformed in accordance with a symmetric irreducible representation of a molecule symmetry group);
(c) Invariant according to the time reversal operation.

For a molecule, which has some or other symmetry, three components $J_{x},J_{y}$, and $J_{z}$ (or their linear combinations) of the angular momentum operator are transformed in accordance with one or the other irreducible representation of a molecular symmetry group. This gives us the possibility to construct a correct effective Hamiltonian even without using the general formulae, Equations (17), (18), and (20)–(23). In particular, the symmetry group of the CH${}_{3}$Cl molecule (which is considered in the present study) is isomorphic to the C${}_{3v}$ point symmetry group. The C${}_{3v}$ group has three irreducible representations: symmetric, $A_{1}$, antisymmetric, $A_{2}$, and two-dimensional irreducible representation *E* (the $\left(v_{6}=1\right)$ vibrational state which is considered in this paper just as the state of *E*-type symmetry, and it is described by the doubly degenerate vibrational mode, $q_{6}^{E1}$ and $q_{6}^{E2}$). In this case, in accordance with the irreducible tensorial sets theory (we have no possibility to discuss here theorems and results of that theory and refer the reader to, e.g., Refs. [85,86,87,88,89,90,91,92], the effective Hamiltonian of the *E*-type vibrational state have to be written in the following form (see Equations (19) and (24)):

(25) $$\begin{array}{c} {\tilde{H}}^{\left(v_{1},E\right)}=\sum_{\Gamma }{\left[{\left(|v_{1},E\rangle ⊗\langle v_{1},E|\right)}^{\Gamma }⊗{\tilde{H}}_{\left(11\right)}^{\Gamma }\right]}^{A_{1}} \end{array}$$

(here, the operator ${\tilde{H}}^{\left(v_{1}\right)}$, Equation (25), is nothing other than the first term on the right hand side of Equation (19) being written in the symmetrized form). The sign ⊗ in Equation (25) denotes a tensorial product; $\Gamma =A_{1},A_{2},$ or *E*.

Omitting all intermediate calculations and explanations (which can be found, e.g., in Refs. [70,92]), we immediately present the result for the effective rotational operator in the most general form, which allows one to take into account all possible effects and interactions described by spectroscopic parameters, in the *E*-type vibrational states of axially symmetric molecules. In this case, because in the present study only the isolated $\nu_{6}$ band is considered, we mention here only the effective Hamiltonian of the isolated doubly degenerate vibrational state of the *E*-type symmetry in order to define the notations in the results. In the absence of interactions with the other vibrational states, the effective Hamiltonian of a doubly degenerate vibrational state can be written in the following form [70] (Ref. [105] should be mentioned also, in which some of the effects considered in the present paper, were discussed):

(26) $$\begin{array}{c} {\tilde{H}}^{\left(E\right)}\equiv H_{1}^{E}+H_{2}^{E}+H_{3}^{E}, \end{array}$$

where we have

(27) $$\begin{array}{ccc} H_{1}^{E} & & =(|E_{1}\rangle \langle E_{1}\left|+\right|E_{2}\rangle \langle E_{2}|)\{E^{e}+B^{e}\left(J_{x}^{2}+J_{y}^{2}\right)+C^{e}J_{z}^{2}-D_{J}^{e}J^{4}\\ & & -D_{JK}^{e}J^{2}J_{z}^{2}-D_{K}^{e}J_{z}^{4}+H_{J}^{e}J^{6}+H_{JK}^{e}J^{4}J_{z}^{2}+H_{KJ}^{e}J^{2}J_{z}^{4}+H_{K}^{e}J_{z}^{6}\\ & & +L_{J}^{e}J^{8}+L_{JJK}^{e}J^{6}J_{z}^{2}+L_{JK}^{e}J^{4}J_{z}^{4}+L_{KKJ}^{e}J^{2}J_{z}^{6}\\ & & +L_{K}^{e}J_{z}^{8}+P_{J}^{e}J^{10}+P_{JJK}^{e}J^{8}J_{z}^{2}+\ldots \\ & & +{\left[\left(\epsilon^{e}J_{z}+\epsilon_{J}^{e}J_{z}J^{2}+\epsilon_{K}^{e}J_{z}^{3}+\ldots \right),\left(J_{+}^{3}+J_{-}^{3}\right)\right]}_{+}+\ldots ,\} \end{array}$$

(28) $$\begin{array}{ccc} H_{2}^{E} & & =(|E_{1}\rangle \langle E_{2}\left|-\right|E_{2}\rangle \langle E_{1}|)\{2{\left(C\zeta \right)}^{e}J_{z}+\eta_{J}^{e}J_{z}J^{2}+\eta_{K}^{e}J_{z}^{3}+\eta_{JJ}^{e}J_{z}J^{4}\\ & & +\eta_{JK}^{e}J_{z}^{3}J^{2}+\eta_{KK}^{e}J_{z}^{5}+\eta_{JJJ}^{e}J_{z}J^{6}+\eta_{JJK}^{e}J_{z}^{3}J^{4}+\eta_{JKK}^{e}J_{z}^{5}J^{2}\\ & & +\eta_{KKK}^{e}J_{z}^{7}+\eta_{JJJJ}^{e}J_{z}J^{8}+\eta_{JJJK}^{e}J_{z}^{3}J^{6}+\ldots \}, \end{array}$$

(29) $$\begin{array}{ccc} H_{3}^{E} & & =(|E_{2}\rangle \langle E_{2}\left|-\right|E_{1}\rangle \langle E_{1}|)\{{\left[C^{e},\left(J_{+}^{2}+J_{-}^{2}\right)\right]}_{+}+{\left[F^{e},\left(J_{+}^{4}+J_{-}^{4}\right)\right]}_{+}\\ & & +(|E_{1}\rangle \langle E_{2}\left|+\right|E_{2}\rangle \langle E_{1}|){\left[iD^{e},\left(J_{+}^{2}-J_{-}^{2}\right)\right]}_{+}+{\left[iG^{e},\left(J_{+}^{4}-J_{-}^{4}\right)\right]}_{+}\}, \end{array}$$

and

(30) $$\begin{array}{ccc} C^{e} & = & \frac{1}{2}\gamma^{e}+\frac{1}{2}\gamma_{J}^{e}J^{2}+\gamma_{K}^{e}J_{z}^{2}+\frac{1}{2}\gamma_{JJ}^{e}J^{4}+\gamma_{JK}^{e}J^{2}J_{z}^{2}+\gamma_{KK}^{e}J_{z}^{4}+\ldots ,\\ D^{e} & = & \delta^{e}J_{z}+\delta_{J}^{e}J_{z}J^{2}+\delta_{K}^{e}J_{z}^{3}+\delta_{JJ}^{e}J_{z}J^{4}+\delta_{JK}^{e}J^{2}J_{z}^{3}+\delta_{KK}^{e}J_{z}^{5}+\ldots ,\\ F^{e} & = & \frac{1}{2}\kappa^{e}+\frac{1}{2}\kappa_{J}^{e}J^{2}+\kappa_{K}^{e}J_{z}^{2}+\frac{1}{2}\kappa_{JJ}^{e}J^{4}+\kappa_{JK}^{e}J^{2}J_{z}^{2}+\kappa_{KK}^{e}J_{z}^{4}+\ldots ,\\ G^{e} & = & \theta^{e}J_{z}+\theta_{J}^{e}J_{z}J^{2}+\theta_{K}^{e}J_{z}^{3}+\ldots \end{array}$$

In Equations (27)–(29) the $E^{e}$, $B^{e}$, $C^{e}$, $D_{J}^{e}$, $D_{JK}^{e}$, $D_{K}^{e}$, $H_{J}^{e}$, $H_{JK}^{e}$, $H_{KJ}^{e}$, $H_{K}^{e}$, $L_{J}^{e}$,…are the rotational and centrifugal distortion parameters of the *E*-type symmetry vibrational state, respectively. The operators $\left(J_{+}^{3}+J_{-}^{3}\right)$ connect rotational states |JK〉 and $|JK^{\prime }\rangle$ with different values of the quantum numbers *K*, namely, $\Delta K=K-K^{\prime }=\pm 3$, and the operators $J_{+}$ and $J_{-}$ have the form of $J_{\pm }=J_{x}\mp iJ_{y}$. The parameters $\epsilon_{J}^{e}$ and $\epsilon_{K}^{e}$ describe the *J* and *K* dependence of the main $\epsilon^{e}$ parameter. The expression ${\left[\ldots ,\ldots \right]}_{+}$ denotes an anticommutator. The operator Equation (28) is connected with the so-called k−l splittings (see, e.g., [62]) in a symmetric top molecule. In this case, the first $2{\left(C\zeta \right)}^{e}J_{z}$ term describes the main part of such splittings; other parameters describe centrifugal corrections of different order to the main parameter. The operator of Equation (29) describes different couplings between ro–vibrational states of different K-values. It is important that, in particular, operators from $H_{3}^{E}$:

(1) Provide $a_{1}/a_{2}$ splittings of ro–vibrational energies $E_{\left[J\;K,a_{1}\right]}$ and $E_{\left[J\;K,a_{2}\right]}$ for different values of the quantum number *K* (the operators $C^{e}$ and $D^{e}$ are responsible for the $a_{1}/a_{2}$ splittings for states with K=1, operators $F^{e}$ and $G^{e}$ are responsible for the $a_{1}/a_{2}$ splittings for states with K=2).
(2) Are responsible (the $J_{+}^{2}\pm J_{-}^{2}$ operators) for the borrowing of intensities from one Q—sub-band to the other.

#### 3.2.2. Symmetrized Ro–Vibrational Functions

In the most general form, symmetrized ro–vibrational functions of a symmetric top XY${}_{3}$ ($C_{3v}-$symmetry) molecule can be written in the following form [92]:

(31) $$\begin{array}{ccc} |v\gamma_{v},JK\gamma_{r},m\gamma \sigma \rangle & \equiv & (|v\gamma_{v}\rangle ⊗|JK\gamma_{r}{\rangle )}_{\sigma }^{\gamma }. \end{array}$$

Here, $|v\gamma_{v},JK\gamma_{r},m\gamma \sigma \rangle$, $|v\gamma_{v}\rangle$ and $|JK\gamma_{r}\rangle$ denote ro–vibrational, vibrational, and pure rotational wave functions, respectively; γ is a symmetry (irreducible representation of the $C_{3v}$ point group: γ = $a_{1}$, $a_{2}$, or *e*); σ refer to the line of the corresponding irreducible representation (σ=1 or 2 for γ=e and can be omitted for $\gamma =a_{1}$ or $a_{2}$); the index *m* distinguishes between ro–vibrational states of the same symmetry; the indices $\gamma_{v}$ and $\gamma_{r}$ indicate the symmetry of the vibrational and pure rotational states for which one has 0≤K≤J, and the symbol ⊗ denotes a direct tensorial product.

Symmetrized pure rotational functions $|JK\gamma_{r}\sigma_{r}\rangle$ can be constructed on the basis of well-known rotational functions [104]:

(32) $$\begin{array}{ccc} |Jkm\rangle & = & \exp\left(-ik\varphi \right)d_{km}^{j}\left(\vartheta \right)\exp\left(-im\psi \right) \end{array}$$

(in Equation (32), one has J≥k≥−J; since, for a free molecule, the index *m* in the functions of Equation (32) is nonessential, we omit it in the following in this paper) if one takes into account the following two circumstances:

(1) The functions |Jk〉 are also symmetrized functions, and any set of |Jk〉 functions (for *J* fixed and −J≤k≤J) is transformed in accordance with the irreducible representation $D^{\left(J\right)}$ of the SO(3) symmetry group (Ref. [104]).
(2) Because the C${}_{3v}$ symmetry group is a subgroup of the SO(3) group, any irreducible representation $D^{\left(J\right)}$ of the SO(3) group is divided into a set of irreducible representations ($\gamma =a_{1},a_{2},$ and *e*) of the C${}_{3v}$ group. In this case (in accordance with the general rules of the theory of group, see, e.g., [106]), one can construct superpositions of functions |Jk〉 which will be transformed already in accordance with irreducible representations of the C${}_{3v}$ group. Such pure rotational (symmetrized in the C${}_{3v}$ group) functions have the form
  (33) $$\begin{array}{ccc} \;\sqrt{2}|JK\gamma_{r}\sigma_{r}\rangle & = & C_{JK\gamma_{r}\sigma_{r}}^{l}\{|JK\rangle +{\left(-1\right)}^{l}{\left(-1\right)}^{J+K}|J-K\rangle \}. \end{array}$$
  Corresponding coefficients $C_{JK\gamma_{r}\sigma_{r}}^{l}$ in Equation (33) are simple numbers, and their nonzero values are presented in the last column of Table 5.

The symmetrized ro–vibrational $|v\gamma_{v},JK\gamma_{r},m\gamma \sigma \rangle$ functions, Equation (31), are the tensorial products of the symmetrized vibrational [61] and rotational, Equation (33), functions. In accordance with the irreducible tensorial sets theory, they can be written as

(34) $$\begin{array}{ccc} |v\gamma_{v},JK\gamma_{r},m\gamma \sigma \rangle & = & \sqrt{\left[\gamma \right]}\sum_{\sigma_{v}\sigma_{r}}\left(\begin{array}{ccc} \gamma & \gamma_{v} & \gamma_{r}\\ \sigma & \sigma_{v} & \sigma_{r} \end{array}\right)|v\gamma_{v}\sigma_{v}\rangle |JK\gamma_{r}\sigma_{r}\rangle , \end{array}$$

where [γ] denotes the dimension of the irreducible representation γ, namely, $\left[A_{1}\right]$ = $\left[A_{2}\right]$ = 1, and [E] = 2; $\left(\begin{array}{ccc} \gamma & \gamma_{v} & \gamma_{r}\\ \sigma & \sigma_{v} & \sigma_{r} \end{array}\right)$ are the so-called 3Γ−symbols of the point symmetry group (for the C${}_{3v}$ symmetry group, nonzero 3Γ−symbols are shown in Table 6).

The use of Equations (33) and (34) offers the possibility to present the symmetrized ro–vibrational functions of a molecule (of the $\left(v_{6}=1,E\right)$ vibrational state of CH${}_{3}$Cl, in our case) in a more traditional form:

(35) $$\begin{array}{ccc} \sqrt{2}|vE,JK,m\gamma \sigma \rangle & = & \sum_{\gamma_{r}\sigma }A_{JKm\gamma \sigma }^{\gamma_{r}\sigma_{r}}|vE_{1}\rangle |JK\gamma_{r}\sigma_{r}\rangle \\ & & +\sum_{\gamma_{r}\sigma_{r}}B_{JKm\gamma \sigma }^{\gamma_{r}\sigma_{r}}|vE_{2}\rangle |JK\gamma_{r}\sigma_{r}\rangle , \end{array}$$

where the coefficients $A_{JKm\gamma \sigma }^{\gamma_{r}\sigma_{r}}$ and $B_{JKm\gamma \sigma }^{\gamma_{r}\sigma_{r}}$ are presented in Table 7.

#### 3.2.3. Hamiltonian Matrix Elements

Any matrix element of the operators (27)–(30) on the functions (33) can be obtained if one considers the values of the basic matrix elements of the operators $J_{\alpha }$(α=x,y,z) on the pure rotational functions |Jk〉, Equation (32) (here and further, as mentioned above, the quantum number *m* is omitted in functions Equation (32)). To realize this, one should take into account the acting of the $J_{\alpha }\left(\alpha =x,y,z\right)$ operators (or, which is the same, of the $J_{z}$, and $J_{\pm }=J_{x}\mp iJ_{y}$ operators) on the |Jk〉 functions. Following Ref. [107], one can write

(36) $$\begin{array}{c} J_{z}|Jk\rangle =k|Jk\rangle \end{array}$$

and

(37) $$\begin{array}{c} J_{\pm }|Jk\rangle ={\left\{J\left(J+1\right)-k\left(k\pm 1\right)\right\}}^{1/2}|Jk\pm 1\rangle . \end{array}$$

From these relations, one can easily obtain the nonzero matrix elements:

(38) $$\begin{array}{c} \langle J^{\prime }k^{\prime }|J_{z}|Jk\rangle =k\delta_{J^{\prime }J}, \end{array}$$

(39) $$\begin{array}{c} \langle J^{\prime }k^{\prime }|J_{+}|Jk\rangle ={\left\{J\left(J+1\right)-k\left(k+1\right)\right\}}^{1/2}\delta_{J^{\prime }J}\delta_{k^{\prime }k+1}, \end{array}$$

and

(40) $$\begin{array}{c} \langle J^{\prime }k^{\prime }|J_{-}|Jk\rangle ={\left\{J\left(J+1\right)-k\left(k-1\right)\right\}}^{1/2}\delta_{J^{\prime }J}\delta_{k^{\prime }k-1}. \end{array}$$

### 3.3. Ro–Vibrational Analysis and Parameters of the Effective Hamiltonian

A set of the 5124 assigned transitions from Section 3.1 was used for the determination of the upper ro–vibrational energy values of the $\left(v_{6}=1,E\right)$ vibrational state of the CH${}_{3}{}^{35}$Cl molecule. The 2077 upper energy values were determined and are presented in Supplementary Data S2; see also the statistical information in Table 2). The upper energy values obtained were then used in a weighted fit procedure with the effective Hamiltonian; Equations (26)–(30). The result of the fit is shown in Table 8 (values in parentheses are 1σ statistical confidence intervals for corresponding parameters).

**Figure 5:**
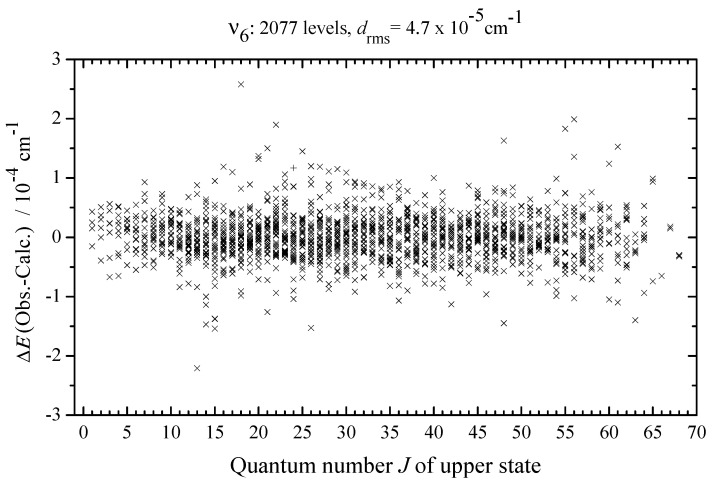
Residuals $E_{i}^{\mathrm{obs}}-E_{i}^{\mathrm{calc}}$ of effective Hamiltonian fit calculations of the $\nu_{6}\left(E\right)$ band of CH${}_{3}{}^{35}$Cl dependent on upper state quantum number *J*.

The set of 25 spectroscopic parameters obtained from the least squares adjustment reproduces the 2077 initial upper energy values with the $d_{\mathrm{rms}}=4.7\times 10^{-5}$ cm${}^{-1}$ (5124 experimental transition wavenumbers are reproduced with a $d_{\mathrm{rms}}=2.04\times 10^{-4}$ cm${}^{-1}$; see also statistical information in Table 2 and column 4 of Supplementary Data S1 and Table 3 where the values $\delta_{\nu }$ of differences between the experimental and calculated with the parameters from Table 8 transition wavenumbers are shown). For illustration of the quality of the results obtained, column 4 of Supplementary Data S2 presents differences δ between the experimental upper energy values and those calculated with the parameters from Table 8. Figure 5 illustrates differences ΔE (observed–calculated) for upper energy values as well as the fit statistics for the $\left(v_{6}=1\right)$ vibrational state of CH${}_{3}$Cl. We would like to note that the obtained results are considerably more extensive and precise in comparison with analogous preceding data known in the literature [6,43]. Highly accurate ro–vibrational functions, which were obtained during the fit procedure, are a good basis for the further precise analysis (see Section 3.6) of the absolute line strengths.

### 3.4. Line Strengths: Experimental Intensities of Ro–Vibrational Lines of the $\nu_{6}$ Band

Experimental line strengths of the $\nu_{6}$ fundamental were determined from the fit of their line shapes with a Hartmann–Tran profile [108,109,110] (for the $\Gamma_{0}$, $\Delta_{2}$, $\nu_{VC}$ parameters of a Hartmann–Tran profile, see Table 1 of [110], and line strengths were adjusted while the line positions $\nu_{0}$ were fixed to their values given in Supplementary Data S1 to this paper; the Doppler parameter $\Gamma_{D}$ was calculated in the standard way). The instrumental line shape function $S_{I}\left(\nu \right)$ was taken into account in accordance with a boxcar apodization line shape function [57,111]:

(41) $$\begin{array}{c} S_{I}\left(\nu \right)≃\left[\Omega_{\max}\frac{2\pi }{\nu_{0}\Omega_{\max}}\Pi \left(\frac{2\pi \nu }{\nu_{0}\Omega_{\max}}\right)\right]⊗2d_{\mathrm{MOPD}}sinc\left(2\nu d_{\mathrm{MOPD}}\right) \end{array}$$

and used in the relation, which is an analogue of Equation (17) from [57]:

(42) $$\begin{array}{c} \tau {\left(\tilde{\nu }\right)}^{\left(\exp\right)}=\tau \left(\tilde{\nu }\right)⊗S_{I}\left(\tilde{\nu }\right). \end{array}$$

In Equations (41)–(42), the sign ⊗ means a convolution; the $\Omega_{\max}$ is the so-called “solid angle”; Π is an external rectangular “boxcar” function with width $\left(\nu_{0}\Omega_{\max}\right)/\left(2\pi \right)$; and

(43) $$\begin{array}{c} sinc(\alpha )=\sin(\pi \alpha )/(\pi \alpha ) \end{array}$$

(for more details, see [57]). The value $\tau (\tilde{\nu })$ in Equation (42):

(44) $$\begin{array}{c} \tau \left(\tilde{\nu }\right)=S_{\nu }^{P}\cdot F\left(\tilde{\nu }-\nu \right)\cdot PL\equiv S_{\nu }^{N}\cdot \frac{F(\tilde{\nu }-\nu )}{k_{B}T})\cdot PL, \end{array}$$

is a factor of the exponent in the well-known Beer–Lambert–Bouguer law (see, e.g., Ref. [57]), *L* is the path length, *P* is the pressure of the sample gas, $k_{B}$ and *T* are the Boltzmann constant and temperature in K, and $F(\tilde{\nu }-\nu )$ is one of the line profile functions (see, e.g., [112,113,114,115,116,117,118,119,120,121,122,123,124,125,126]). On the other hand, the $\tau (\tilde{\nu })$ value, being measured by FTIR spectroscopy, is obtained as follows (see, e.g., [127]):

(45) $$\begin{array}{c} \tau {\left(\tilde{\nu }\right)}^{\left(\exp\right)}=\frac{1}{\mathrm{lg}\left(e\right)}\mathrm{lg}\left(\frac{I_{0}\left(\tilde{\nu }\right)}{I(\tilde{\nu })}\right). \end{array}$$

The intensities $S_{\nu }^{N}(T=294.45$ K) (see Equation (19)) of 2081 lines (about 2800 transitions) of the $\nu_{6}$ band were determined in accordance with the above discussion from the fit of their line shapes with a computer code which uses a Hartman–Tran profile (small amounts of H${}_{2}$O and CO${}_{2}$ and a natural abundance [48] of CH${}_{3}$Cl in the sample (see Table 9), were taken into account in accordance with the discussion of Ref. [128]). The results obtained are shown in column 5 of Supplementary Data S1 (see also Table 3).

**Figure 6:**
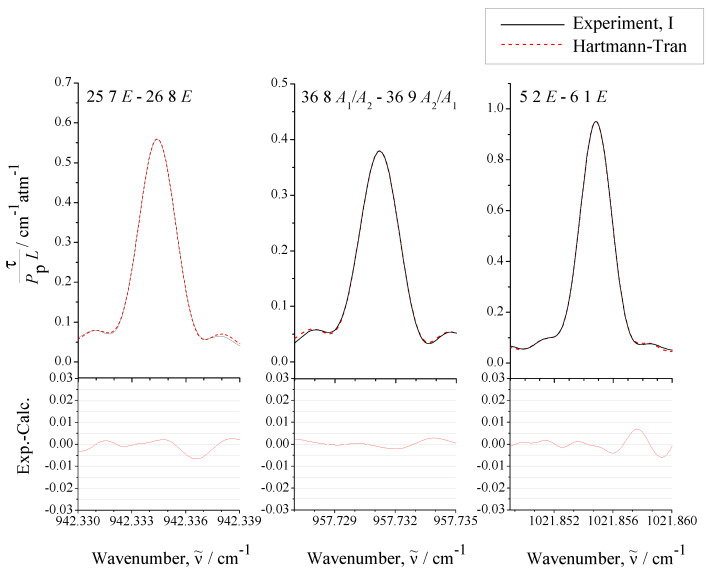
Some examples of the line shape analysis in the experimental spectrum I (for experimental conditions, see Section 2 and Table 1). The fit of the experimental line shapes was made with the qSDRP profile of individual lines. The bottom part of the figure shows the (exp.–calc.) residuals.

In fact, as will be seen from the discussion below, a reduced form of the Hartman–Tran profile, namely, the quadratic-speed-dependent Rautian, qSDRP, was used. For comparison, the same was carried out with the quadratic-speed-dependent Voigt (qSDVP) profile and the values $R=d_{\mathrm{rms}}$(qSDVP)/$d_{\mathrm{rms}}$(qSDRP), where $d_{\mathrm{rms}}$(qSDVP) and $d_{\mathrm{rms}}$(qSDRP) are 1σ statistical $d_{\mathrm{rms}}-$deviations of corresponding fits, are presented in column 12 of Supplementary Data S1 (the higher the *R* value, the more strongly the qSDRP profile improves the shape of the concrete line in comparison with the qSDVP profile). Columns 9, 10, and 11 of Supplementary Data S1 (see also Table 3) present the values of parameters $\Gamma_{0}$, $\Delta_{2}$ and $\nu_{\mathrm{VC}}$ which are obtained from the fit of line shapes with the qSDRP profile. When the parameter $\Delta_{2}$ or $\nu_{\mathrm{VC}}$ is absent in column 10 or 11, this implies that the corresponding $d_{\mathrm{rms}}-$value is larger than the value of the parameter itself, and such a parameter is not suitable for the use of the qSDRP profile. When both parameters $\Delta_{2}$ and $\nu_{\mathrm{VC}}$ are absent in column 10 and 11 (in this case, R=1.00 in column 12), it means that the use of the qSDRP profile for this concrete line has no advantages in comparison with the use of the quadratic-speed-dependent Voigt profile. Column 6 shows the total uncertainties $\Delta_{S}^{\exp}$ (given in percent) which were estimated in accordance with the following equation:

(46) $$\begin{array}{c} \Delta_{S}^{\exp}={\left\{\Delta {\left(L\right)}^{2}+\Delta {\left(T\right)}^{2}+\Delta {\left(TP\right)}^{2}+\Delta {\left(PP\right)}^{2}+\Delta {\left(\mathrm{stat}\right)}^{2}\right\}}^{1/2} \end{array}$$

(in Equation (46), Δ(L), Δ(T), Δ(TP), and Δ(PP) are uncertainties in optical path length, temperature, total pressure, and partial pressure of CH${}_{3}$Cl in the sample, and Δ(stat) is a statistical $d_{\mathrm{rms}}—$deviation of the fit of a particular line shape). To illustrate the quality of the analysis, Figure 6 shows three examples of line shape fits (with qSDRP profile) from which the line intensities were determined.

### 3.5. Line Strength Analysis: Improvement of the Model and Calculation Scheme

As was mentioned in the introduction, the theory of the absolute line strengths based on their symmetry properties was discussed earlier for different types of molecules (see, e.g., [1,5]). Here, we show that (based on the properties of the irreducible tensorial sets of the $C_{3v}$ point symmetry group instead of the analogous properties of the SO(3) symmetry group) it is possible to derive an efficient (but much simpler and clearer in real applications) model of the effective dipole moment operator for the axially symmetric $C_{3v}$-type molecule. In this case, we focus on the allowed transitions that correspond to the selection rule ΔK=±1 for the perpendicular *E*-type ro–vibrational band which is considered in the present paper (here, we analyze “allowed” transitions only and plan to make analogous line strength discussion of both the “forbidden” transitions and the parallel-type ro–vibrational bands in the nearest future). As will be seen from the further discussion, distribution of the result both to the forbidden transitions and to the parallel-type bands is simple and can be made by the reader without problem.

In general case, the absolute strength of the vibration–rotation line, due to transitions from the |i〉 to |f〉 state, is determined by the expression (see, e.g., [1]):

(47) $$\begin{array}{c} S_{\nu_{0}}=\frac{8\pi^{3}\tilde{\nu_{0}}}{4\pi \epsilon_{0}3hc}\left[1-\exp\left(-\frac{hc\nu_{0}}{k_{B}T}\right)\right]N\frac{g_{i}}{Z(T)}\exp\left(-\frac{E_{i}}{k_{B}T}\right)R_{i}^{f}, \end{array}$$

where $\tilde{\nu_{0}}=\left(E_{f}-E_{i}\right)/hc$ is the wavenumber of the transition, and $E_{f}$ and $E_{i}$ are the upper and the lower ro–vibrational energies of the transition; $g_{i}$ is the “nuclear spin” statistical weight (for XH${}_{3}$Y molecule, $g_{i}$ = 4 for all ro–vibrational states of the $A_{1}$, $A_{2}$, and *E* symmetry; both in $g_{i}$ and in $Z_{r}\left(T\right)$ we took into account only spin statistics of H nuclei, taking into account that the spins of X and Y nuclei are insufficient for calculation of the $g_{i}/Z\left(T\right)$ value in Equation (47)); *N* is the number of absorbing molecules per unit volume; Z(T) is the partition function. In the present study we used Z(294.45) = 14,353.37 for the CH${}_{3}{}^{35}$Cl molecule, which was calculated in accordance with Formula (4) from [129]:

(48) $$\begin{array}{c} Z\left(T\right)=a+bT+cT^{2}+dT^{3}. \end{array}$$

Necessary for calculation, values of parameters *a*, *b*, *c*, and *d* were taken from Table 1 of Ref. [129].

The term $R_{i}^{f}=\left|\langle f\right|\mu_{Z}^{\prime }{\left|i\rangle \right|}^{2}$ in Equation (47) is the matrix element of the operator

(49) $$\begin{array}{c} \mu_{Z}^{\prime }=G^{+}P_{Z}G \end{array}$$

applied to the functions |i〉 and |f〉 of the lower and upper ro–vibrational states. The operator $P_{Z}$ is the Z—component of the dipole moment of a molecule (see, e.g., [130,131]) and, depending on the instantaneous distances between the nuclei, it can be written with the approximation

(50) $$\begin{array}{c} P_{Z}=\sum_{\alpha }k_{Z\alpha }\left(\mu_{\alpha }^{e}+\sum_{\lambda }\mu_{\alpha }^{\lambda }q_{\lambda }+\sum_{\lambda ,\nu \geq \lambda }\mu_{\alpha }^{\lambda \nu }q_{\lambda }q_{\nu }+\ldots \right). \end{array}$$

Here, $k_{Z\alpha }$ are elements of the direction cosines matrix [104]; $\mu_{\alpha }^{e}$ are the components of the equilibrium (permanent) dipole moment of the molecule in the molecule-fixed coordinate system; $q_{\lambda }$ are dimensionless normal vibrational coordinates [61,62] of a molecule; and $\mu_{\alpha }^{\lambda }$, $\mu_{\alpha }^{\lambda \nu }$, …are the parameters which describe the dependence of dipole moment components $\mu_{\alpha }$ on the normal vibrational coordinates. The first terms $\mu_{\alpha }^{e}$ in Equation (50) are responsible for the pure rotational transitions; the second terms (which are proportional to the first order of vibrational coordinates) are responsible for the appearance of the fundamental band transitions in absorption (in more general case, of the bands which correspond to a change of only one vibrational quantum number by unit), etc. The operator *G* in Equation (49) is a unitary operator known from the theory of effective operators (see, e.g., Refs. [61,62,96,97,98,99,100,101,102]). If one takes the results of the mentioned articles into account, then it is possible to show that for an arbitrary polyatomic molecule, Equation (49) can be transformed to the following expression:

(51) $$\begin{array}{c} \mu_{Z}^{\prime }=\sum_{v}{|0\rangle }^{v}\mu_{Z}\langle v|, \end{array}$$

where the values ${}^{v}\mu_{Z}$ depend only on the operators $k_{Z\alpha }$ and $J_{\alpha }$ and do not depend on the vibrational operators; |0〉 and 〈v| are the vibrational function of the lower (in our case, of the ground) and upper vibrational states. The operators ${}^{v}\mu_{Z}$ have the following form:

(52) $$\begin{array}{c} {}^{v}\mu_{Z}=\sum_{j}{}^{v}\mu_{j}{}^{v}A_{j}, \end{array}$$

where ${}^{v}A_{j}$ are symmetrized rotational operators and ${}^{v}\mu_{j}$ are called the effective dipole moment parameters of a specific vibrational band, 〈v|←|0〉.

As was discussed in the above sections, the use of the symmetry properties is essential. This is true not only for the Hamiltonian and the energy structure analysis, but for the line strength analysis, as well.

#### 3.5.1. Effective Dipole Moment Operator for the E-Type Band

Taking Equations (49) and (50) and the symmetry properties of the operators $J_{\beta }$ and $k_{z\beta }$, β=x,y,z (see Section 3.5.2, Section 3.5.3, Section 3.5.4 and Section 3.5.5 below) into account, it is not difficult to show that the effective dipole moment operator $\mu_{Z}^{\prime }$, Equation (51), for the *E*-type band (the $\nu_{6}$ band which is discussed in the present paper is just one such type band) can be expressed in the following form:

(53) $${\left(\mu_{z}^{\prime }\right)}^{A_{2}}=|v_{gr}A_{1}\rangle \left[\sum_{\Gamma ,\Omega ,n,\tilde{\Gamma }}({\langle vE|⊗R^{\Gamma (\Omega ,n\tilde{\Gamma })E})}^{A_{2}}P_{v_{gr}A_{1},vE}^{\Gamma (\Omega ,n\tilde{\Gamma })E)}\right],$$

where, in accordance with general Equation (13),

(54) $${\left(\langle vE|⊗R^{\Gamma (\Omega ,n\tilde{\Gamma })E}\right)}^{A_{2}}=\frac{1}{\sqrt{2}}\left(\langle vE1|(R_{2}^{\Gamma (\Omega ,n\tilde{\Gamma })E}-\langle vE2|R_{1}^{\Gamma (\Omega ,n\tilde{\Gamma })E}\right).$$

In Equations (53) and (54), the $|v_{gr}A_{1}\rangle$ and 〈vE| are the symmetrized vibrational functions (as *v*, we denote here the couple of vibrational quantum numbers *v* and *l* (see, e.g., [61]); *E* is the symmetry of the function); $P_{v_{gr}A_{1},vE}^{\Gamma (\Omega ,n\tilde{\Gamma })E}$ are the parameters of the effective dipole moment operator for the *E*-type band; the symbol ⊗ denotes the direct product [88]. The $R_{\sigma }^{\Gamma (\Omega n\tilde{\Gamma })E}$ are operators which are symmetrized in the $C_{3v}$ point symmetry group and depend on the $J_{\beta }$ angular momentum and direct cosines $k_{Z\beta }$ operators:

(55) $$R_{\sigma }^{\Gamma (\Omega ,n\tilde{\Gamma })E}=\frac{1}{2}\left[{\left(k^{\Gamma }⊗R_{\sigma }^{\left(\Omega n\tilde{\Gamma }\right)}\right)}_{\sigma }^{E}+{\left(R_{\sigma }^{\left(\Omega n\tilde{\Gamma }\right)}⊗k^{\Gamma }\right)}_{\sigma }^{E}\right].$$

The values Γ and $\tilde{\Gamma }$ denote a symmetry of the direction of cosines and angular momentum operators; Ω is the power of the angular momentum operators; *n* distinguishes between different operators of the same values of Ω and symmetry $\tilde{\Gamma }$. The presentation of the operators $R_{\sigma }^{\Gamma (\Omega n\tilde{\Gamma })E}$ in the form of Equation (55) is caused by the requirement that they must be hermitian.

#### 3.5.2. Irreducible Rotational Operators of the SO(3) and $C_{3v}$ Symmetry First-Order Operators

In this paper, following Refs. [106,107], we choose the rotational operators $J_{\beta }$ in the following form:

(56) $$\begin{array}{c} J_{x}=i\left\{\frac{\sin\varphi }{\sin\theta }\frac{\partial }{\partial \psi }-\sin\varphi \cot\frac{\partial }{\partial \varphi }+\cos\varphi \frac{\partial }{\partial \theta }\right\}, \end{array}$$

(57) $$\begin{array}{c} J_{y}=i\left\{-\frac{\cos\varphi }{\sin\theta }\frac{\partial }{\partial \psi }+\cos\varphi \cot\frac{\partial }{\partial \varphi }+\sin\varphi \frac{\partial }{\partial \theta }\right\}, \end{array}$$

(58) $$\begin{array}{c} J_{z}=i\frac{\partial }{\partial \varphi }. \end{array}$$

It is important that

(1) The rotational operators, Equations (56)–(58), satisfy the commutation relations; see, e.g., [95]:
  (59) $$\begin{array}{c} {\left(J_{\alpha },J_{\beta }\right)}_{-}=J_{\alpha }J_{\beta }-J_{\beta }J_{\alpha }=-i\sum_{\gamma }\epsilon_{\alpha \beta \gamma }J_{\gamma }, \end{array}$$
  where $\epsilon_{\alpha \beta \gamma }$ is the totally antisymmetric tensor;
(2) They also satisfy the important transformation conditions [107]:
  (60) $$\begin{array}{c} \left(J_{x}\mp iJ_{y}\right)|Jk\rangle ={\left\{J(J+1)-k(k\pm 1)\right\}}^{1/2}|Jk\pm 1\rangle \end{array}$$
  and
  (61) $$\begin{array}{c} J_{z}|Jk\rangle =k|Jk\rangle ; \end{array}$$
(3) As a consequence, three operators $R_{m}^{1(1)}$ (m=0,±1) can be constructed:
  (62) $$\begin{array}{c} R_{0}^{1(1)}=J_{z}, \end{array}$$
  and
  (63) $$\begin{array}{c} R_{\pm 1}^{1(1)}=\mp \frac{1}{\sqrt{2}}\left(J_{x}\mp iJ_{y}\right), \end{array}$$
  which satisfy the conditions
  (64) $$\begin{array}{c} \langle \tilde{J}\tilde{k}|R_{m}^{1(1)}|Jk\rangle =\frac{1}{{\left(2\tilde{J}+1\right)}^{1/2}}C_{Jk,1m}^{\tilde{J}\tilde{k}}<\tilde{J}\left\|R^{1(1)}\right\|J> \end{array}$$
  and, as a further consequence, they are nothing other than the first-rank irreducible rotational operators of the SO(3) symmetry group. In Equation (42) $\tilde{J}=J,J\pm 1$, −J≤k≤J, and $\tilde{k}=k,k\pm 1$; the values $C_{Jk,1m}^{\tilde{J}\tilde{k}}$ and $\langle \tilde{J}\|R^{1,1}\|J\rangle$ are the well-known Clebsch–Gordan coefficients and the so-called reduced matrix elements [104]:
  (65) $$\begin{array}{c} \langle J\|R^{1(1)}\|J\rangle ={\left\{\left(2J+1\right)J\left(J+1\right)\right\}}^{1/2}. \end{array}$$
  Following the scheme of connection of irreducible tensorial operators of the SO(3) and $C_{3v}$ symmetry groups (see, e.g., [78]), one can obtain three first-order irreducible rotational operators that are symmetrized in accordance with irreducible representations of the $C_{3v}$ point symmetry group:
  (66) $$\begin{array}{c} R^{1(A_{2})}\equiv J_{z}=R_{0}^{1(1)}\in A_{2}, \end{array}$$
  (67) $$\begin{array}{c} R_{1}^{1(E)}\equiv J_{y}=-\frac{i}{\sqrt{2}}\left(R_{-1}^{1(1)}+R_{1}^{1(1)}\right)\in E_{1}, \end{array}$$
  and
  (68) $$\begin{array}{c} R_{2}^{1(E)}\equiv J_{x}=\frac{1}{\sqrt{2}}\left(R_{-1}^{1(1)}-R_{1}^{1(1)}\right)\in E_{2}. \end{array}$$
  Taking into account Equations (60)–(65), one can obtain
  (69) $$\begin{array}{c} \langle \tilde{J}\tilde{k}|R^{1(A_{2})}|Jk\rangle \equiv \langle \tilde{J}\tilde{k}|J_{z}|Jk\rangle =\delta_{J\tilde{J}}\delta_{k\tilde{k}}k, \end{array}$$
  (70) $$\begin{array}{ccc} & & \langle \tilde{J}k\pm 1|R_{2}^{1(E)}|Jk\rangle \equiv \langle \tilde{J}k\pm 1|J_{x}|Jk\rangle =\mp \langle \tilde{J}k\pm 1|iR_{1}^{1(E)}|Jk\rangle \\ & & \equiv \mp \langle \tilde{J}k\pm 1|iJ_{y}|Jk\rangle =\delta_{J\tilde{J}}{\left\{(J\pm k+1)(J\mp k)\right\}}^{1/2} \end{array}$$

#### 3.5.3. The $\lambda_{m}^{\left(1\right)}$ and $\lambda_{\sigma }^{\left(\Gamma \right)}$ Irreducible Direction Cosines Operators

In the analysis, it is suitable to use the direction cosines $k_{Z\beta }$ in the following form:

(71) $$\begin{array}{c} k_{Zz}=\cos\theta ,\;\;\;\;k_{Zx}=\sin\theta \sin\varphi ,\;\;\;\;k_{Zy}=-\sin\theta \cos\varphi . \end{array}$$

It is important that

(1) The direction cosines, which are obtained in such a way, satisfy the commutation relations
  (72) $$\begin{array}{c} {\left(J_{\alpha },k_{Z\beta }\right)}_{-}=-i\sum_{\gamma }\epsilon_{\alpha \beta \gamma }k_{Z\gamma }, \end{array}$$
  specific to the components of the rotational angular momentum in the molecular-fixed coordinate system (Ref. [95]);
(2) Their combinations
  (73) $$\begin{array}{c} \lambda_{0}^{1}=k_{Zz},\;\;\;\;\mathrm{and}\;\;\;\;\lambda_{\pm 1}^{1}=\mp \frac{1}{\sqrt{2}}\left(k_{Zx}\mp ik_{Zy}\right) \end{array}$$
  form the first rank irreducible tensor $\lambda_{m}^{\left(1\right)}$ of the SO(3) symmetry group;
(3) As a consequence, for the nonzero matrix elements $<\tilde{J}\tilde{k}|\lambda_{m}^{\left(1\right)}|Jk>$, the following relations (analogous to Equation (64)) are valid:
  (74) $$\begin{array}{c} \langle \tilde{J}\tilde{k}|\lambda_{m}^{\left(1\right)}|Jk\rangle =\frac{1}{{\left(2J+1\right)}^{1/2}}C_{Jk,1m}^{\tilde{J}\tilde{k}}\langle \tilde{J}\|\lambda^{\left(1\right)}\|J\rangle \end{array}$$
  with
  (75) $$\begin{array}{c} \langle J\|\lambda^{\left(1\right)}\|J\rangle =\left(2J+1\right), \end{array}$$
  (76) $$\begin{array}{c} \langle J\|\lambda^{\left(1\right)}\|J+1\rangle =-{\left\{(2J+1)(2J+3)\right\}}^{1/2}, \end{array}$$
  (77) $$\begin{array}{c} \langle J\|\lambda^{\left(1\right)}\|J-1\rangle ={\left\{(2J+1)(2J-1)\right\}}^{1/2}; \end{array}$$
(4) Analogously to Equations (66)–(68), it is possible to show that three irreducible operators $\lambda_{\sigma }^{\left(\Gamma \right)}$ of the $C_{3v}$ point symmetry group are
  (78) $$\begin{array}{c} \lambda^{\left(A_{2}\right)}\equiv k^{\left(A_{2}\right)}=k_{Zz},\;\;\;\;\;\lambda_{1}^{\left(E\right)}\equiv k_{1}^{\left(E\right)}=k_{Zy},\;\;\;\;\;\lambda_{2}^{\left(E\right)}\equiv k_{2}^{\left(E\right)}=k_{Zx}, \end{array}$$
  and the corresponding nonzero matrix elements of these operators on the functions
  ∣Jk> are
  (79) $$\begin{array}{c} \langle Jk|k_{Zz}|Jk\rangle =k{\left\{\frac{\left(2J+1\right)}{J(J+1)}\right\}}^{1/2}, \end{array}$$
  (80) $$\begin{array}{ccc} & & \langle Jk|k_{Zx}|Jk\pm 1\rangle =\pm \langle Jk|ik_{Zy}|Jk\pm 1\rangle \\ & & =\frac{1}{2}{\left\{\frac{\left(2J+1)(J\mp k)(J\pm k+1\right)}{J(J+1)}\right\}}^{1/2}; \end{array}$$
  (81) $$\begin{array}{c} \langle Jk|k_{Zz}|J+1k\rangle ={\left\{\frac{\left(J+k+1)(J-k+1\right)}{\left(J+1\right)}\right\}}^{1/2}, \end{array}$$
  (82) $$\begin{array}{ccc} & & \langle Jk|k_{Zx}|J+1k\pm 1\rangle =\pm \langle Jk|ik_{Zy}|J+1k\pm 1\rangle \\ & & =\mp \frac{1}{2}{\left\{\frac{\left(J\pm k+1)(J\pm k+2\right)}{\left(J+1\right)}\right\}}^{1/2}; \end{array}$$
  (83) $$\begin{array}{c} \langle Jk|k_{Zz}|J-1k\rangle ={\left\{\frac{\left(J+k)(J-k\right)}{J}\right\}}^{1/2}, \end{array}$$
  (84) $$\begin{array}{ccc} & & \langle Jk|k_{Zx}|J-1k\pm 1\rangle =\pm \langle Jk|ik_{Zy}|J-1k\pm 1\rangle \\ & & =\pm \frac{1}{2}{\left\{\frac{\left(J\mp k)(J\mp k-1\right)}{J}\right\}}^{1/2}. \end{array}$$

#### 3.5.4. Effective Dipole Moment Operator: The Main Part

Let us return now to the general expression of the effective dipole moment operator in the form of Equations (51)–(53) and consider the main part of this operator which depends on a pair of operators $R_{\sigma }^{\Gamma =E(\Omega =0,\tilde{\Gamma }=A_{1})E}$ (two components with σ=1 and 2). Evidently, its nonzero matrix elements, which just determine the main selection rules for the *E*-type perpendicular band, are determined by the formulas Equations (80), (82), and (84).

#### 3.5.5. Effective Dipole Moment Operator: First Order Corrections

Here, we discuss the first-order corrections to the main part of the effective dipole moment operator (they are sometimes called “centrifugal distortion corrections” analogously to the corresponding correction operators in the effective Hamiltonian). Taking into account the symmetry properties of the first-order rotational operators (see Equations (66)–(68)), one can constrain corresponding first-order corrections to the effective dipole moment operator (see Section 3.5.1). Following Equations (53) and (54), three such types of operators can be constructed: $R_{\sigma }^{E(1,A_{2})E}$, $R_{\sigma }^{E(1,E)E}$, and $R_{\sigma }^{A_{2}\left(1,E\right)E}$ (σ=1,2). In accordance with the general scheme of connection of the irreducible tensorial sets of the $C_{3v}$ point symmetry group (see Equation (34) and discussion there), it is possible to show that the $R_{1}^{E(1,A_{2})E}$ operator can be expressed as

(85) $$\begin{array}{ccc} & & R_{1}^{E(1,A_{2})E}=\frac{1}{2}{\left\{k^{E}⊗R^{1(A_{2})}\right\}}_{1}^{E}+\frac{1}{2}{\left\{R^{1(A_{2})}⊗k^{E}\right\}}_{1}^{E}\\ & & =\frac{1}{\sqrt{2}}\left(\begin{array}{ccc} E & E & A_{2}\\ 1 & 2 & \end{array}\right)k_{2}^{E}R^{1(A_{2})}+\frac{1}{\sqrt{2}}\left(\begin{array}{ccc} E & A_{2} & E\\ 1 & & 2 \end{array}\right)R^{1(A_{2})}k_{2}^{E}\\ & & =\frac{1}{2}\left(J_{z}k_{Zx}-k_{Zx}J_{z}\right)=\frac{i}{2}k_{Zy}, \end{array}$$

Analogously, one can obtain

(86) $$\begin{array}{c} R_{2}^{E(1A_{2})E}=\frac{1}{2}{\left\{k^{E}⊗R^{1(A_{2})}\right\}}_{2}^{E}+\frac{1}{2}{\left\{R^{1(A_{2})}⊗k^{E}\right\}}_{2}^{E}=\frac{i}{2}k_{Zx}. \end{array}$$

As is seen from a comparison of Equations (85) and (86) and corresponding to Equation (78), the operators $R_{1}^{E(1,A_{2})E}$ and $R_{2}^{E(1,A_{2})E}$ differ from the operators $k_{1}^{E}$ and $k_{2}^{E}$ only by the factor $\frac{i}{2}$. This means that the operator $R^{E(1\;A_{2})E}$ gives only a minor correction to the main effective dipole moment operator, and in a real calculation it can be omitted (in this case, one should take into account that the corresponding parameter $P_{\ldots ,\ldots }^{E(1,A_{2})E}$ (see Equation (53)), should be purely imaginary). Absolutely the same conclusion can be made after analogous consideration of the third pair of the abovementioned operators, namely, $R_{\sigma }^{A_{2}\left(1,E\right)E}$.

Let us consider now the second pair of operators, $R_{\sigma }^{E(1,E)E}$. The use of the scheme of Equation (85) gives the following result:

(87) $$\begin{array}{c} R_{1}^{E(1,E)E}=\frac{1}{\sqrt{2}}\left\{k_{Zx}J_{x}-k_{Zy}J_{y}\right\} \end{array}$$

and

(88) $$\begin{array}{c} R_{2}^{E(1,E)E}=\frac{1}{\sqrt{2}}\left\{k_{Zx}J_{y}+k_{Zy}J_{x}\right\}. \end{array}$$

If, now, one takes into account a discussion in Section 3.5.2 and Section 3.5.3 and considers matrix elements $<\tilde{J}\tilde{k}|R_{\sigma }^{E(1,E)E}|Jk>$ of the operators $R_{\sigma }^{E(1,E)E}$, then it is possible to see that these matrix elements differ from zero only when ΔJ=0,±1, and Δk=±2. This means that the operator $R^{E(1,E)E}$ can only contribute to the strengths of “forbidden” transitions with the value Δk=±2. As was mentioned in Section 3.5, we analyze here “allowed” transitions only and plan to offer analogous line strength discussion of both the “forbidden” transitions and the parallel type ro–vibrational bands in the nearest future and apply results to the analysis of the $\nu_{2}+\nu_{3}$ band of CH${}_{3}$Cl. The operator $R^{E(1,E)E}$ can be also omitted from the present consideration.

#### 3.5.6. Effective Dipole Moment Operator: Second-Order Corrections

Taking into account the symmetry properties of the first-order rotational operators (see Section 3.5.3), one can constrain symmetrized second-order rotational operators. In this case, two of them are the totally symmetric ones which are transformed in accordance with the A${}_{1}$ irreducible representation of the $C_{3v}$ symmetry group, and they are

(89) $$\begin{array}{c} R^{\left(2,1A_{1}\right)}=\sum_{\alpha =x,y,z}J_{\alpha }^{2}\equiv J^{2} \end{array}$$

and

(90) $$\begin{array}{c} R^{\left(2,2A_{1}\right)}=J_{z}^{2}. \end{array}$$

Two pairs of remaining second-order rotational operators are the σ=1,2 components of the two *E*-type operators:

(91) $$\begin{array}{c} R_{1}^{\left(2,1E\right)}=-{\left[J_{z},J_{x}\right]}_{+}\equiv -\left(J_{z}J_{x}+J_{x}J_{z}\right), \end{array}$$

(92) $$\begin{array}{c} R_{2}^{\left(2,1E\right)}={\left[J_{z},J_{y}\right]}_{+}\equiv \left(J_{z}J_{y}+J_{y}J_{z}\right), \end{array}$$

and

(93) $$\begin{array}{c} R_{1}^{\left(2,2E\right)}=J_{x}^{2}-J_{y}^{2}\equiv {\left(R_{1}^{1(1)}\right)}^{2}+{\left(R_{-1}^{1(1)}\right)}^{2}, \end{array}$$

(94) $$\begin{array}{c} R_{2}^{\left(2,2E\right)}=J_{x}J_{y}+J_{y}J_{x}\equiv i{\left(R_{1}^{1(1)}\right)}^{2}-i{\left(R_{-1}^{1(1)}\right)}^{2}. \end{array}$$

It is possible now to obtain all nonzero matrix elements of the operator, Equation (53), with the value Ω=2 which are caused by the effects of the second order. Taking into account the discussion of Section 3.5.2 and Section 3.5.3, it is possible to derive the following results:

(95) $$\begin{array}{c} \langle \tilde{J}\tilde{k}|R_{\sigma }^{E(2,1A_{1})E}|Jk\rangle =\frac{1}{2}\langle \tilde{J}\tilde{k}|k_{\sigma }^{E}|Jk\rangle \left\{J\left(J+1\right)+\tilde{J}\left(\tilde{J}+1\right)\right\}, \end{array}$$

(96) $$\begin{array}{c} \langle \tilde{J}\tilde{k}|R_{\sigma }^{E(2,2A_{1})E}|Jk\rangle =\frac{1}{2}\langle \tilde{J}\tilde{k}|k_{\sigma }^{E}|Jk\rangle \left(k^{2}+{\tilde{k}}^{2}\right), \end{array}$$

(97) $$\begin{array}{ccc} & \langle Jk\mp 1|R_{\sigma }^{E(2,2E)E}|Jk\rangle = & \\ - & \frac{1}{2}\langle Jk\mp 1|k_{\sigma }^{E}|Jk\rangle \left\{J(J+1)-k(k\mp 1)-1\right\}, \end{array}$$

and

(98) $$\begin{array}{ccc} & \langle J+\Delta Jk+\Delta k|R_{\sigma }^{E(2,2E)E}|Jk\rangle = & \\ & \frac{1}{2}\langle J+\Delta Jk+\Delta k|k_{\sigma }^{E}|Jk\rangle \left\{{\left(J-\Delta J\Delta kk\right)}^{2}+\left(J-\Delta J\Delta kk\right)+1\right\}. \end{array}$$

With regard to the $R_{\sigma }^{E(2,1E)E}$ operators, it is possible to show that they (analogously to the above-discussed $R_{\sigma }^{E(1,1E)E}$ ones) only affect the strengths of “forbidden” transitions with the value Δk=±2.

Another set of the second-order corrections to the effective dipole moment operator are two pairs of operators $R_{\sigma }^{A_{2}\left(2,1E\right)E}$ and $R_{\sigma }^{A_{2}\left(2,2E\right)E}$. The use of the operators $R_{\sigma }^{A_{2}\left(2,1E\right)E}$ for calculation of corresponding matrix elements gives the following result:

(99) $$\begin{array}{ccc} & & R_{1}^{A_{2}\left(2,1E\right)E}=\frac{1}{2}{\left\{k^{A_{2}}⊗R^{\left(2,1E\right)}\right\}}_{1}^{E}+\frac{1}{2}{\left\{R^{\left(2,1E\right)}⊗k^{A_{2}}\right\}}_{1}^{E}\\ & & =\frac{1}{\sqrt{2}}\left(\begin{array}{ccc} E & A_{2} & E\\ 1 & & 2 \end{array}\right)k^{A_{2}}R_{2}^{\left(2,1E\right)}\\ & & +\frac{1}{\sqrt{2}}\left(\begin{array}{ccc} E & E & A_{2}\\ 1 & 2 & \end{array}\right)R_{2}^{\left(2,1E\right)}k^{A_{2}}=-\frac{i}{2}\left(k_{Zx}J_{z}+J_{z}k_{Zx}\right) \end{array}$$

and

(100) $$\begin{array}{ccc} & & R_{2}^{A_{2}\left(2,1E\right)E}=\frac{1}{2}{\left\{k^{A_{2}}⊗R^{\left(2,1E\right)}\right\}}_{2}^{E}+\frac{1}{2}{\left\{R^{\left(2,1E\right)}⊗k^{A_{2}}\right\}}_{2}^{E}\\ & & =\frac{i}{2}\left(k_{Zy}J_{z}+J_{z}k_{Zy}\right). \end{array}$$

If one takes into account Equations (69), (70), (80), (82), and (84), then it is possible to show that for any matrix element $\langle \tilde{J}\tilde{k}|R_{\sigma }^{A_{2}\left(2,1E\right)E}|Jk\rangle$, the following relation is valid:

(101) $$\begin{array}{ccc} & & \langle J+\Delta Jk+\Delta k|R_{\sigma }^{A_{2}\left(2,1E\right)E}|Jk\rangle =\\ & & -\frac{1}{2}\left(1+2k\Delta k\right)\langle J+\Delta Jk+\Delta k|k_{\sigma }^{E}|Jk\rangle , \end{array}$$

With regard to the last pair of the second-order operators, $R_{\sigma }^{A_{2}\left(2,2E\right)E}$, it is possible to show that their matrix elements differ from zero only for Δk=±2, i.e., they are needed for the description of “forbidden” transitions only.

#### 3.5.7. Effective Dipole Moment Operator: Higher-Order Corrections

As one can see from the above discussion, extension of the consideration to the higher-order corrections to the effective dipole moment operator is a routine procedure and, for this reason, we do not discuss it here in detail, but only present the final result for the most important higher-order corrections. The obtained result is presented in Table 10. The second column of this table shows operators which are responsible for the different-order corrections to the effective dipole moment operator. Notations of the corresponding parameters are shown in the third column. The fourth column presents nonzero matrix elements of operators from the second column in the form of

(102) $$\begin{array}{c} \langle \tilde{J}\tilde{k}=k\pm 1|R_{\sigma }^{\Gamma (\Omega ,n\tilde{\Gamma })E}|Jk\rangle =\langle \tilde{J}\tilde{k}=k\pm 1|k_{\sigma }^{E}|Jk\rangle C^{\Gamma (\Omega ,n\tilde{\Gamma })E}. \end{array}$$

In this case, the fourth column of Table 10 just presents coefficients $C^{\Gamma (\Omega ,n\tilde{\Gamma })}$.

The results that are derived in the present section and presented in Table 10 are valid for any symmetric ($C_{3v}$ symmetry) top molecule. It is important that they are very simple for their use in real calculations and are very similar to the corresponding basic results for an asymmetric top molecule (see Ref. [1]). At the same time, the obtained results are considerably more simple in comparison with the cumbersome formulas which are derived traditionally on the basis of the irreducible tensorial formalism of the SO(3) group (see, e.g., [2,3,4,5]).

### 3.6. Line Strengths Analysis: Determination of Effective Dipole Moment Parameters and Discussion

The general results obtained in Section 3.5 deliver the basic input for the computer code SYMTOMLIST which was used then in the fit procedure with the goal to determine effective dipole moment parameters of the $\nu_{6}$ band of the CH${}_{3}$Cl molecule.

**Figure 7:**
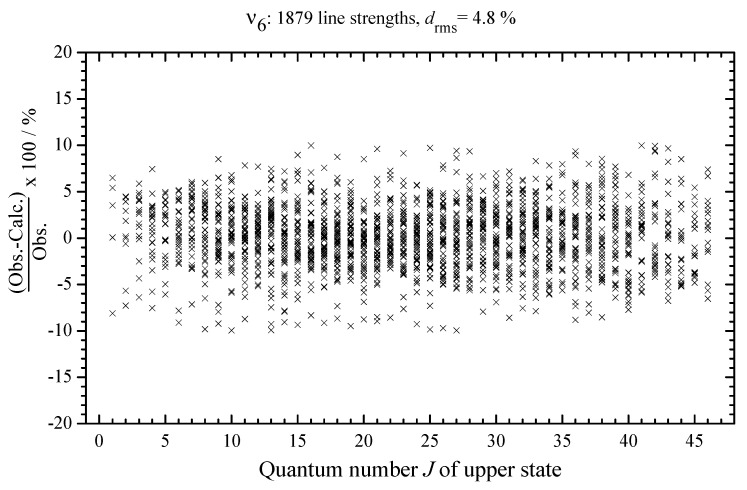
Some examples of the line shape analysis in the experimental spectrum I (for experimental conditions, see Section 2 and Table 1). The fit of the experimental line shapes was made with the qSDRP profile of individual lines. The bottom part of the figure shows the (exp.–calc.) residuals.

The 2804 experimental transition strengths discussed in Section 3.4 were used as the initial data in the fit procedure. The values of the effective dipole moment parameters $P^{E(\Omega ,n\tilde{\Gamma })}$ obtained from the fit are presented in Table 4 together with their 1σ statistical confidence intervals. These parameters give us the possibility to reproduce the strength of the 2804 initial experimental transitions with the $d_{\mathrm{rms}}=3.4\%$. To illustrate the quality of the fit, column 8 of Supplementary Data S1 and Table 3 presents values of differences (in percent)

(103) $$\begin{array}{c} \delta_{\nu }^{S}=100\times \left(\frac{{}^{\left(\exp\right)}S_{\nu_{i}}^{N}-{}^{\left(\mathrm{calc}\right)}S_{\nu_{i}}^{N}}{{}^{\left(\exp\right)}S_{\nu_{i}}^{N}}\right) \end{array}$$

between the experimental absolute line strengths and those calculated with the parameters from Table 4 (see also Figure 7, where the fit residuals for line strengths are shown as a function of the quantum number *J*). Also for illustration, the lower parts of Figure 1, Figure 2, Figure 3 and Figure 4 present simulated spectra which were constructed on the basis of effective Hamiltonian parameters from Table 8 and effective dipole moment parameters from Table 4. One can see a good agreement between upper and lower panels of Figure 1, Figure 2, Figure 3 and Figure 4. We would like to note that the presented analysis provides the first precise and, simultaneously, extensive data on experimental line strengths on the $\nu_{6}$ band of the CH${}_{3}{}^{35}$Cl molecule.

As one can see from the Supplementary Data, the experimental strengths of some assigned transitions were not measured (the corresponding information is absent in columns 5–6 and 8–12) and, as a consequence, they were not used in the fit of dipole moment parameters. However, we theoretically estimated strengths of all these transitions on the basis of the parameters obtained (see Table 8 and Table 4), which are shown in column 7 of Supplementary Data S1 and Table 3.

It can be interesting to estimate the integrated band intensity of the $\nu_{6}$ band on the basis of the obtained values of the effective dipole moment parameters. In our present study, such estimation was made as a direct sum of all possible individual line strengths calculated with the use of general Formula (25) and the derived parameters from Table 4 and Table 8. Such direct calculation gives the value 15.46 cm${}^{-2}\cdot$atm${}^{-1}$ which is close to the corresponding value (15.06±0.19) cm${}^{-2}\cdot$atm${}^{-1}$ from Ref. [6].

## 4. Conclusions

In the present paper, we derived a new, efficient, effective dipole moment operator taking into account fourth-order corrections to the main dipole moment parameter. This operator turned out to be very simple and clearer to handle in applications compared to the existing methods. The results obtained were used for the precise analysis of absolute strengths of 2804 individual transitions of CH${}_{3}{}^{35}$Cl in the region of its $\nu_{6}$ band on the basis of the Hartmann–Tran line profile. As the first step of the study, an analysis of line positions of the $\nu_{6}$ band and ro–vibrational energy values of the $\left(v_{6}=1\right)$ vibrational state was fulfilled on the basis of the high-resolution infrared spectra of CH${}_{3}$Cl specially recorded with a highly resolving Fourier transform infrared spectrometer. The 2077 ro–vibrational energy values of the $\left(v_{6}=1\right)$ vibrational state (corresponding to 5124 assigned transitions of the $\nu_{6}$ band) were used then in the weighted fit procedure and a set of 25 rotational, centrifugal, and *k*-*l*-splitting parameters was obtained which reproduced the 2077 initial upper ro–vibrational energy values with the $d_{\mathrm{rms}}=4.7\times 10^{-5}$ cm${}^{-1}$. The 2081 absolute line strengths (which correspond to 2804 ro–vibrational transitions) were experimentally measured and analyzed with the use of the Voigt and Hartmann–Tran spectral line profiles and the multispectrum fit analysis. Effective parameters of the electric dipole transition moment of the $\nu_{6}$ band were determined by the computer code SYMTOMLIST, created on the basis of a newly derived theoretical approach resulting in six effective dipole moment parameters reproducing the initial experimental strengths of 2804 initial experimental transitions with a relative $d_{\mathrm{rms}}=3.4\%$.

## Figures and Tables

**Table 1:** Experimental setup of the infrared spectra of CH${}_{3}$Cl.

Spectrum	Region /cm${}^{-1}$	Resolution /cm${}^{-1}$	No. of Scans	Source	Detector	Beam- Splitter	Aperture /mm	Opt. Path- Length/m	Temp. /K	Pressure /Pa	Calibr. Gas
I	500–1700	0.0025	1000	Globar	MCT	KBr	1.5	4	(294.45±0.3) K	50	H${}_{2}$O, CO${}_{2}$
II	500–5000	0.003	1860	Globar	MCT	KBr	1.0	24	(294.45±0.3) K	300	H${}_{2}$O, CO${}_{2}$

**Table 5:** Coefficients $C_{JK\gamma_{r}\sigma_{r}}^{l}$ of symmetrized rotational functions.

*J*	*K*	$\gamma_{r}\sigma_{r}$	*l*	Value
Even	0	$a_{1}$	0	${\left(-i\right)}^{J}\sqrt{2}$
Odd	0	$a_{2}$	1	${\left(-i\right)}^{J-1}\sqrt{2}$
Any	3, 6, 9, …	$a_{1}$	0	${\left(-1\right)}^{J}$
		$a_{2}$	1	${\left(-1\right)}^{J+1}$
	1, 4, 7, …	$e_{1}$	0	${\left(-1\right)}^{J}$
		$e_{2}$	1	${\left(-1\right)}^{J+1}$
	2, 5, 8, …	$e_{1}$	0	${\left(-1\right)}^{J}$
		$e_{2}$	1	${\left(-1\right)}^{J-1}$

**Table 6:** Nonzero 3Γ symbols of the $C_{3v}$ symmetry group ${}^{a}$.

$\gamma_{1}\sigma_{1}$	$\gamma_{2}\sigma_{2}$	$\gamma_{3}\sigma_{3}$	Value	$\gamma_{1}\sigma_{1}$	$\gamma_{2}\sigma_{2}$	$\gamma_{3}\sigma_{3}$	Value
$A_{1}$	$A_{1}$	$A_{1}$	1	$A_{2}$	$E_{1}$	$E_{2}$	$1/\sqrt{2}$
$A_{1}$	$A_{2}$	$A_{2}$	1	$E_{1}$	$E_{1}$	$E_{1}$	−1/2
$A_{1}$	$E_{1}$	$E_{1}$	$1/\sqrt{2}$	$E_{1}$	$E_{2}$	$E_{2}$	1/2
$A_{1}$	$E_{2}$	$E_{2}$	$1/\sqrt{2}$				

${}^{a}$ Other nonzero 3Γ symbols are connected with the values given in the table by the relations $\begin{array}{cccccc} & & \gamma_{1} & \gamma_{2} & \gamma_{3}\\ & ( & \sigma_{1} & \sigma_{2} & \sigma_{3} & ) \end{array}$ =$\begin{array}{cccccc} & & \gamma_{2} & \gamma_{3} & \gamma_{1}\\ & ( & \sigma_{2} & \sigma_{3} & \sigma_{1} & ) \end{array}$ = ${\left(-1\right)}^{\gamma_{1}+\gamma_{2}+\gamma_{3}}$$\begin{array}{cccccc} & & \gamma_{1} & \gamma_{3} & \gamma_{2}\\ & ( & \sigma_{1} & \sigma_{3} & \sigma_{2} & ) \end{array}$ where ${\left(-1\right)}^{A_{1}}={\left(-1\right)}^{E}=\;+1;\;{\left(-1\right)}^{A_{1}}=\;-1$.

**Table 7:** Coefficients $A_{JKm\gamma \sigma }^{\gamma_{r}\sigma_{r}}$ and $B_{JKm\gamma \sigma }^{\gamma_{r}\sigma_{r}}$ of symmetrized ro–vibrational functions.

*J*	*K*	mγσ	$\;\;\;\gamma_{r}\sigma_{r}\;\;\;$	$A_{JKm\gamma \sigma }^{\gamma_{r}\sigma }$	$B_{JKm\gamma \sigma }^{\gamma_{r}\sigma }$
any	k≠0,3,6,9,…	$a_{1}$	$e_{1}$	1	
			$e_{2}$		1
		$a_{2}$	$e_{1}$		−1
			$e_{2}$	1	
		$e_{1}$	$e_{1}$	−1	
			$e_{2}$		1
		$e_{2}$	$e_{1}$		1
any	*k* = 3, 6, 9,…	$1e_{1}$	$a_{1}$	1	
			$a_{2}$		1
		$1e_{2}$	$a_{1}$		1
			$a_{2}$	−1	
		$2e_{1}$	$a_{1}$	1	
			$a_{2}$		−1
		$2e_{2}$	$a_{1}$		1
			$a_{2}$	1	
even	*k* = 0	$e_{1}$	$a_{1}$	$\sqrt{2}$	
		$e_{2}$	$a_{1}$		$\sqrt{2}$
odd	*k* = 0	$e_{1}$	$a_{2}$		$\sqrt{2}$
		$e_{2}$	$a_{2}$	$-\sqrt{2}$	

**Table 8:** Spectroscopic parameters of the $\left(v_{6}=1\right)$ state of CH${}_{3}{}^{35}$Cl (in cm${}^{-1}$) ${}^{\left(a\right)}$.

Parameter	Value
*E*	1018.0707900(43)
*B*	0.4417686446(97)
*C*	5.23070591(17)
$D_{J}\;\times \;10^{6}$	0.6049990(56)
$D_{JK}\;\times \;10^{5}$	0.678610(25)
$D_{K}\;\times \;10^{4}$	0.85663560(15)
$H_{J}\;\times \;10^{12}$	−0.33657(93)
$H_{JK}\;\times \;10^{10}$	0.14360(66)
$H_{KJ}\;\times \;10^{9}$	0.1873(22)
$H_{K}\;\times \;10^{8}$	0.13300(49)
$L_{JK}\;\times \;10^{13}$	−0.1389(42)
$L_{KKJ}\;\times \;10^{12}$	−0.2198(49)
2Cζ	2.6202558(10)
$\eta_{J}\;\times \;10^{4}$	−0.156270(14)
$\eta_{K}\;\times \;10^{3}$	−0.112648(17)
$\eta_{JJ}\;\times \;10^{10}$	0.8931(92)
$\eta_{JK}\;\times \;10^{8}$	−0.2986(22)
$\eta_{KK}\;\times \;10^{7}$	0.12944(56)
$\eta_{JKK}\;\times \;10^{11}$	−0.8241(79)
$\gamma \;\times \;10^{3}$	−0.1205968(96)
$\gamma_{J}\;\times \;10^{9}$	0.5859(14)
$\gamma_{K}\;\times \;10^{6}$	−0.4166(42)
$\kappa \;\times \;10^{9}$	0.145(15)
$\kappa_{J}\;\times \;10^{12}$	−0.2327(92)
$\kappa_{JJ}\;\times \;10^{16}$	0.415(20)

${}^{\left(a\right)}$ Values in parentheses are 1σ standard errors.

**Table 9:** Estimated concentration of different species in the experimental sample (in percent).

Spectrum	CH${}_{3}$${}^{35}$Cl	CH${}_{3}$${}^{37}$Cl	H${}_{2}$O	CO${}_{2}$
I	74.21	23.73	2.05 ± 0.22	0.013 ± 0.001

**Table 10:** Effective dipole moment parameters of the *E*-type band of axially symmetric ($C_{3v}$) molecule.

	Operator, $R_{\sigma }^{\Gamma (\Omega ,n\tilde{\Gamma })E}$	Parameter, $P^{\Gamma (\Omega ,n\tilde{\Gamma })}$	$C^{\Gamma (\Omega ,n\tilde{\Gamma })}$ Coefficient, Equation (102)
1	$R_{\sigma }^{E(0,A_{1})E}=k_{\sigma }^{E}$	$P^{E(0,A_{1})}$	1
2	$R_{\sigma }^{E(2,1A_{1})E}$	$P^{E(2,1A_{1})}$	$\frac{1}{2}\left\{J\left(J+1\right)+\tilde{J}\left(\tilde{J}+1\right)\right\}$
3	$R_{\sigma }^{E(2,2A_{1})E}$	$P^{E(2,2A_{1})}$	$\frac{1}{2}\left(k^{2}+{\tilde{k}}^{2}\right)$
4	$R_{\sigma }^{E(2,2E)E}$	$P^{E(2,2E)}$	$-\frac{1}{2}\left\{J\left(J+1\right)-k\tilde{k}-1\right\}$, ΔJ=0,Δk=±1
			$\frac{1}{2}\left\{\left(J-\Delta J\Delta kk\right)\left(\tilde{J}-\Delta J\Delta k\tilde{k}+1\right)+1\right\}$, ΔJ=±1,Δk=±1
5	$R_{\sigma }^{A_{2}\left(2,1E\right)E}$	$P^{A_{2}\left(2,1E\right)}$	$-\frac{\Delta k}{2}\left(k+\tilde{k}\right)$
6	$R_{\sigma }^{E(4,1A_{1})E}$	$P^{E(4,1A_{1})}$	$\frac{1}{2}\left\{J^{2}{\left(J+1\right)}^{2}+{\tilde{J}}^{2}{\left(\tilde{J}+1\right)}^{2}\right\}$
7	$R_{\sigma }^{E(4,2A_{1})E}$	$P^{E(4,2A_{1})}$	$\frac{1}{2}\left\{J\left(J+1\right)k^{2}+\tilde{J}\left(\tilde{J}+1\right){\tilde{k}}^{2}\right\}$
8	$R_{\sigma }^{E(4,3A_{1})E}$	$P^{E(4,3A_{1})}$	$\frac{1}{2}\left\{k^{4}+{\tilde{k}}^{4}\right\}$
9	$R_{\sigma }^{A_{2}\left(4,1E\right)E}$ ${}^{\left(a\right)}$	$P^{A_{2}\left(4,1E\right)}$	$-\frac{\Delta k}{2}\left(k+\tilde{k}\right)\left\{J\left(J+1\right)+\tilde{J}\left(\tilde{J}+1\right)\right\}$
10	$R_{\sigma }^{A_{2}\left(4,2E\right)E}$ ${}^{\left(b\right)}$	$P^{A_{2}\left(4,2E\right)}$	$-\frac{\Delta k}{2}\left(k+\tilde{k}\right)\left(k^{2}+{\tilde{k}}^{2}\right)$

${}^{\left(a\right)}$$R_{\sigma }^{A_{2}\left(4,1E\right)E}=\frac{1}{2}{\left\{k^{A_{2}}⊗R^{\left(2,1E\right)}\right\}}_{\sigma }^{E}R^{\left(2,1A_{1}\right)}+\frac{1}{2}R^{\left(2,1A_{1}\right)}{\left\{R^{\left(2,1E\right)}⊗k^{A_{2}}\right\}}_{\sigma }^{E}$. ${}^{\left(b\right)}$$R_{\sigma }^{A_{2}\left(4,2E\right)E}=\frac{1}{2}{\left\{k^{A_{2}}⊗R^{\left(2,2E\right)}\right\}}_{\sigma }^{E}R^{\left(2,2A_{1}\right)}+\frac{1}{2}R^{\left(2,2A_{1}\right)}{\left\{R^{\left(2,1E\right)}⊗k^{A_{2}}\right\}}_{\sigma }^{E}$.

## Data Availability

Not applicable.

## Supplementary Materials

The following supporting information can be downloaded at: https://www.mdpi.com/article/10.3390/ijms241512122/s1.
