# Supplementary material for: Effective Dipole Moment Model for Axially Symmetric C3v Molecules: Application to the Precise Study of Absolute Line Strengths of the ν6 Fundamental of CH335Cl
Source: Int J Mol Sci. 2023 Jul 28;24(15):12122. doi: 10.3390/ijms241512122 (PMC10418978; doi:10.3390/ijms241512122)
Supplement: Supplementary file 1 [file ijms-24-12122-s001.zip › Supplementary Data S1.pdf]

Supplementary material 1

Line list of transitions of the (v_6=1) state of $CH_3{}^{35}Cl$.

Electronic supplementary materials to:

Effective dipole moment model for axially symmetric C_{3v} molecules: Application to the precise study of absolute line strengths of the ν_6 fundamental of CH_3Cl

O. N. Ulenikov[‡,a], E. S. Bekhtereva[a], O. V. Gromova[a], A. L. Fomchenko[a], Yu. B. Morzhikova[a], S. S. Sidko[a], C. Sydow[b], and S. Bauerecker[§,b]

[a] Research School of High–Energy Physics, National Research Tomsk Polytechnic University, Tomsk 634050, Russia;

[b] Institut für Physikalische und Theoretische Chemie, Technische Universität Braunschweig, D - 38106, Braunschweig, Germany.

[a)] Experimental line position (in cm^{-1}).
[b)] Differences $\delta_\nu{}^{b)}$ between the experimental and calculated line position (in $10^{-5}cm^{-1}$).
[c)] Line intensity determined from the fit of experimental line shape with a Hartmann-Tran (qSDRP) profile (in $cm^{-2}\cdot atm^{-1}$).
[d)] Experimental error Δ_S (in per cent) in the line intensity determination.
[e)] Calculated line intensity with parameters from Table 11 (in $cm^{-2}\cdot atm^{-1}$).
[f)] Differences δ_S (in per cent) between the experimental line strengths and such calculated with the parameters from Table 11.
[g)] Parameter obtained from the fit of the experimental line shape with the Hartmann–Tran (qSDRP) profile. Value is absent in the column when corresponding parameter is insufficient for the fit. In this case, such parameter was taken as zero.
[h)] Here $R = d_{\rm rms}(\text{qSDVP})/d_{\rm rms}(\text{qSDRP})$; $d_{\rm rms}$(qSDVP) and $d_{\rm rms}$(qSDRP) are obtained from the line shape fit.

List of transitions assigned to the ν_6 band of $CH_3{}^{35}Cl$

J	K_a	Γ	J'	K'_a	Γ'	$\nu^{a)}$	$\delta_\nu{}^{b)}$	$S_\nu^{\rm exp}(294.45)^{c)}$	$\Delta_S{}^{d)}$	$S_\nu^{\rm calc}(294.45)^{e)}$	$\delta_S{}^{f)}$	$\Gamma_0{}^{g)}$	$\Delta_2{}^{g)}$	$\nu_{\rm VC}{}^{g)}$	$R^{h)}$
	1			2		3	4	5	6	7	8	9	10	11	12
29	15	E	30	16	E	888.61548	-12			0.4995E-05					
41	13	E	42	14	E	888.72740	-48			0.6048E-05					
22	16	E	23	17	E	889.46544	-43			0.2944E-05					
28	15	E	29	16	E	889.59274	-41			0.5697E-05					
40	13	E	41	14	E	889.73922	-46			0.7239E-05					
46	12	E	47	13	E	889.76661	11			0.5313E-05					
52	11	A_1	53	12	A_2	889.76661	-6			0.3024E-05					
52	11	A_2	53	12	A_1	889.76661	-6			0.3024E-05					
21	16	E	22	17	E	890.42162	-41			0.3277E-05					
27	15	E	28	16	E	890.56777	12			0.6469E-05					
39	13	E	40	14	E	890.74829	-34			0.8625E-05					
45	12	E	46	13	E	890.79172	5			0.6494E-05					
51	11	A_2	52	12	A_1	890.80755	9			0.3793E-05					
51	11	A_1	52	12	A_2	890.80755	9			0.3793E-05					
20	16	E	21	17	E	891.37516	16			0.3633E-05					
26	15	E	27	16	E	891.53916	9			0.7314E-05					
38	13	E	39	14	E	891.75453	-20			0.1023E-04					
44	12	E	45	13	E	891.81409	0			0.7901E-05					
50	11	A_2	51	12	A_1	891.84553	-5			0.4736E-05					
50	11	A_1	51	12	A_2	891.84553	-5			0.4736E-05					
19	16	E	20	17	E	892.32476	-2			0.4011E-05					
25	15	E	26	16	E	892.50740	-1			0.8233E-05					
37	13	E	38	14	E	892.75773	-22			0.1208E-04					
43	12	E	44	13	E	892.83379	6			0.9569E-05					
49	11	A_1	50	12	A_2	892.88117	14			0.5886E-05					
49	11	A_2	50	12	A_1	892.88117	14			0.5886E-05					
18	16	E	19	17	E	893.27166	32			0.4413E-05					
24	15	E	25	16	E	893.47294	28			0.9228E-05					
36	13	E	37	14	E	893.75812	-16			0.1419E-04					
42	12	E	43	13	E	893.85045	-14			0.1154E-04					
48	11	A_2	49	12	A_1	893.91385	7			0.7282E-05					
48	11	A_1	49	12	A_2	893.91385	7			0.7282E-05					
17	16	E	18	17	E	894.21498	29			0.4838E-05					
23	15	E	24	16	E	894.43493	14			0.1030E-04					
35	13	E	36	14	E	894.75578	6			0.1661E-04					
41	12	E	42	13	E	894.86469	5			0.1384E-04					
47	11	A_1	48	12	A_2	894.94374	-9			0.8968E-05					
47	11	A_2	48	12	A_1	894.94374	-9			0.8968E-05					
16	16	E	17	17	E	895.15493	13			0.5287E-05					
22	15	E	23	16	E	895.39392	12			0.1145E-04					
28	14	A_2	29	15	A_1	895.59027	-56			0.1728E-04					
28	14	A_1	29	15	A_2	895.59027	-56			0.1728E-04					
34	13	E	35	14	E	895.75037	13			0.1934E-04					
40	12	E	41	13	E	895.87567	-22			0.1654E-04					
46	11	A_2	47	12	A_1	895.97124	8			0.1099E-04					
46	11	A_1	47	12	A_2	895.97124	8			0.1099E-04					
21	15	E	22	16	E	896.34984	16			0.1267E-04					
27	14	A_2	28	15	A_1	896.56451	-42			0.1956E-04					
27	14	A_1	28	15	A_2	896.56451	-42			0.1956E-04					
33	13	E	34	14	E	896.74196	11			0.2243E-04					
39	12	E	40	13	E	896.88418	-13			0.1967E-04					
45	11	A_1	46	12	A_2	896.99584	7			0.1342E-04					
45	11	A_2	46	12	A_1	896.99584	7			0.1342E-04					
51	10	E	52	11	E	897.07938	10			0.7198E-05					
20	15	E	21	16	E	897.30273	32			0.1398E-04					
26	14	A_1	27	15	A_2	897.53560	-40			0.2203E-04					
26	14	A_2	27	15	A_1	897.53560	-40			0.2203E-04					
32	13	E	33	14	E	897.73072	21			0.2588E-04					
38	12	E	39	13	E	897.88994	5			0.2328E-04					
44	11	A_1	45	12	A_2	898.01776	13			0.1630E-04					
44	11	A_2	45	12	A_1	898.01776	13			0.1630E-04					
50	10	E	51	11	E	898.11683	0			0.8976E-05					
19	15	E	20	16	E	898.25200	3			0.1535E-04					
25	14	A_2	26	15	A_1	898.50397	-4			0.2472E-04					
25	14	A_1	26	15	A_2	898.50397	-4			0.2472E-04					
31	13	E	32	14	E	898.71628	5			0.2974E-04					
37	12	E	38	13	E	898.89270	7			0.2744E-04					
43	11	A_1	44	12	A_2	899.03679	4			0.1971E-04					
43	11	A_2	44	12	A_1	899.03679	4			0.1971E-04					
49	10	E	50	11	E	899.15152	-20			0.1114E-04					
18	15	E	19	16	E	899.19860	24			0.1680E-04					
24	14	A_1	25	15	A_2	899.46892	-3			0.2761E-04					
24	14	A_2	25	15	A_1	899.46892	-3			0.2761E-04					
30	13	E	31	14	E	899.69911	13			0.3402E-04					
36	12	E	37	13	E	899.89256	6			0.3218E-04					
42	11	A_2	43	12	A_1	900.05315	6			0.2373E-04					
42	11	A_1	43	12	A_2	900.05315	6			0.2373E-04					
17	15	E	18	16	E	900.14159	3			0.1833E-04					
48	10	E	49	11	E	900.18412	18			0.1377E-04					
54	9	E	55	10	E	900.28794	11			0.6335E-05					
23	14	A_1	24	15	A_2	900.43072	-9			0.3071E-04					
23	14	A_2	24	15	A_1	900.43072	-9			0.3071E-04					

(cont.)

J	K_a	Γ	J'	K_a'	Γ'	$\nu^{a)}$	$\delta_\nu{}^{b)}$	$S_\nu^{\mathrm{exp}}(294.45)^{c)}$	$\Delta_S{}^{d)}$	$S_\nu^{\mathrm{calc}}(294.45)^{e)}$	$\delta_S{}^{f)}$	$\Gamma_0{}^{g)}$	$\Delta_2{}^{g)}$	$\nu_{\mathrm{VC}}{}^{g)}$	$R^{h)}$
	1			2		3	4	5	6	7	8	9	10	11	12
29	13	E	30	14	E	900.67894	18			0.3874E-04					
35	12	E	36	13	E	900.88961	11			0.3758E-04					
41	11	A_2	42	12	A_1	901.06677	11			0.2843E-04					
41	11	A_1	42	12	A_2	901.06677	11			0.2843E-04					
16	15	E	17	16	E	901.08147	-9			0.1993E-04					
47	10	E	48	11	E	901.21359	12			0.1693E-04					
53	9	E	54	10	E	901.33295	19			0.7999E-05					
22	14	A_2	23	15	A_1	901.38954	-4			0.3402E-04					
22	14	A_1	23	15	A_2	901.38954	-4			0.3402E-04					
28	13	E	29	14	E	901.65571	16			0.4392E-04					
34	12	E	35	13	E	901.88376	15			0.4369E-04					
15	15	E	16	16	E	902.01818	-16			0.2160E-04					
40	11	A_1	41	12	A_2	902.07757	13			0.3391E-04					
40	11	A_2	41	12	A_1	902.07757	13			0.3391E-04					
46	10	E	47	11	E	902.24033	2			0.2073E-04					
21	14	A_1	22	15	A_2	902.34528	4			0.3754E-04					
21	14	A_2	22	15	A_1	902.34528	4			0.3754E-04					
52	9	E	53	10	E	902.37507	-1			0.1006E-04					
58	8	A_2	59	9	A_1	902.48422	-3			0.3891E-05					
58	8	A_1	59	9	A_2	902.48422	-3			0.3891E-05					
27	13	E	28	14	E	902.62950	17			0.4958E-04					
33	12	E	34	13	E	902.87496	14			0.5055E-04					
39	11	A_1	40	12	A_2	903.08530	-12			0.4026E-04					
39	11	A_2	40	12	A_1	903.08530	-12			0.4026E-04					
45	10	E	46	11	E	903.26450	7			0.2527E-04					
20	14	A_1	21	15	A_2	903.29779	1			0.4125E-04					
20	14	A_2	21	15	A_1	903.29779	1			0.4125E-04					
51	9	E	52	10	E	903.41494	15			0.1258E-04					
57	8	A_2	58	9	A_1	903.53884	-16			0.4999E-05					
57	8	A_1	58	9	A_2	903.53884	-16			0.4999E-05					
26	13	E	27	14	E	903.60012	1			0.5572E-04					
32	12	E	33	13	E	903.86321	10			0.5823E-04					
38	11	A_1	39	12	A_2	904.09066	9			0.4759E-04					
38	11	A_2	39	12	A_1	904.09066	9			0.4759E-04					
19	14	A_2	20	15	A_1	904.24715	-4			0.4516E-04					
19	14	A_1	20	15	A_2	904.24715	-4			0.4516E-04					
44	10	E	45	11	E	904.28593	10			0.3066E-04					
50	9	E	51	10	E	904.45192	6			0.1567E-04					
25	13	E	26	14	E	904.56800	15			0.6234E-04					
56	8	A_1	57	9	A_2	904.59141	21			0.6393E-05					
56	8	A_2	57	9	A_1	904.59141	21			0.6393E-05					
31	12	E	32	13	E	904.84863	15			0.6678E-04					
37	11	A_1	38	12	A_2	905.09298	8			0.5599E-04					
37	11	A_2	38	12	A_1	905.09298	8			0.5599E-04					
18	14	A_1	19	15	A_2	905.19343	-2			0.4925E-04					
18	14	A_2	19	15	A_1	905.19343	-2			0.4925E-04					
43	10	E	44	11	E	905.30453	3			0.3702E-04					
49	9	E	50	10	E	905.48627	-1			0.1943E-04					
24	13	E	25	14	E	905.53265	9			0.6946E-04					
55	8	A_1	56	9	A_2	905.64051	-33			0.8138E-05					
55	8	A_2	56	9	A_1	905.64051	-33			0.8138E-05					
30	12	E	31	13	E	905.83101	10			0.7623E-04					
36	11	A_1	37	12	A_2	906.09247	8			0.6557E-04					
36	11	A_2	37	12	A_1	906.09247	8			0.6557E-04					
17	14	A_2	18	15	A_1	906.13646	-10			0.5352E-04					
17	14	A_1	18	15	A_2	906.13646	-10			0.5352E-04					
42	10	E	43	11	E	906.32042	0			0.4451E-04					
23	13	E	24	14	E	906.49430	10			0.7706E-04					
48	9	E	49	10	E	906.51810	6			0.2398E-04					
54	8	A_1	55	9	A_2	906.68792	1			0.1031E-04					
54	8	A_2	55	9	A_1	906.68792	1			0.1031E-04					
29	12	E	30	13	E	906.81052	13			0.8664E-04					
16	14	A_2	17	15	A_1	907.07633	-15			0.5798E-04					
16	14	A_1	17	15	A_2	907.07633	-15			0.5798E-04					
35	11	A_2	36	12	A_1	907.08913	11			0.7645E-04					
35	11	A_1	36	12	A_2	907.08913	11			0.7645E-04					
41	10	E	42	11	E	907.33360	2			0.5326E-04					
22	13	E	23	14	E	907.45287	9			0.8514E-04					
53	8	A_1	54	9	A_2	907.73244	4			0.1301E-04					
53	8	A_2	54	9	A_1	907.73244	4			0.1301E-04					
28	12	E	29	13	E	907.78702	12			0.9802E-04					
59	7	E	60	8	E	907.89207	44			0.4607E-05					
15	14	A_2	16	15	A_1	908.01304	-18			0.6261E-04					
15	14	A_1	16	15	A_2	908.01304	-18			0.6261E-04					
34	11	A_2	35	12	A_1	908.08288	9			0.8872E-04					
34	11	A_1	35	12	A_2	908.08288	9			0.8872E-04					
40	10	E	41	11	E	908.34402	5			0.6345E-04					
21	13	E	22	14	E	908.40828	0			0.9368E-04					
46	9	E	47	10	E	908.57364	9			0.3604E-04					
27	12	E	28	13	E	908.76052	9			0.1104E-03					
52	8	A_2	53	9	A_1	908.77436	7			0.1634E-04					
52	8	A_1	53	9	A_2	908.77436	7			0.1634E-04					

(cont.)

J	K_a	Γ	J'	K'_a	Γ'	$\nu^{a)}$	$\delta_\nu{}^{b)}$	$S_\nu^{\exp}(294.45)^{c)}$	$\Delta_S{}^{d)}$	$S_\nu^{\mathrm{calc}}(294.45)^{e)}$	$\delta_S{}^{f)}$	$\Gamma_0{}^{g)}$	$\Delta_2{}^{g)}$	$\nu_{\mathrm{VC}}{}^{g)}$	$R^{h)}$
	1			2		3	4	5	6	7	8	9	10	11	12
14	14	A_1	15	15	A_2	908.94651	-25			0.6742E-04					
14	14	A_2	15	15	A_1	908.94651	-25			0.6742E-04					
33	11	A_2	34	12	A_1	909.07375	8	.189225E-03	2.7	0.1025E-03	-8.32	4.880E-04			1.0
33	11	A_1	34	12	A_2	909.07375	8			0.1025E-03					
39	10	E	40	11	E	909.35161	5			0.7523E-04					
20	13	E	21	14	E	909.36063	-6			0.1027E-03					
45	9	E	46	10	E	909.59729	2			0.4388E-04					
26	12	E	27	13	E	909.73106	9			0.1238E-03					
51	8	A_2	52	9	A_1	909.81361	4			0.2043E-04					
51	8	A_1	52	9	A_2	909.81361	4			0.2043E-04					
57	7	E	58	8	E	910.00284	1			0.7625E-05					
32	11	A_2	33	12	A_1	910.06173	7	.218294E-03	2.9	0.1179E-03	-7.98	1.375E-04		-.847E+00	1.1
32	11	A_1	33	12	A_2	910.06173	7			0.1179E-03					
19	13	E	20	14	E	910.30994	-4			0.1121E-03					
38	10	E	39	11	E	910.35639	2			0.8880E-04					
44	9	E	45	10	E	910.61831	3			0.5317E-04					
25	12	E	26	13	E	910.69850	-1			0.1383E-03					
50	8	A_2	51	9	A_1	910.85050	27			0.2542E-04					
50	8	A_1	51	9	A_2	910.85050	27			0.2542E-04					
31	11	A_2	32	12	A_1	911.04684	9	.250136E-03	1.4	0.1349E-03	-7.88	4.599E-04	-.645E+00	.321E+01	1.2
31	11	A_1	32	12	A_2	911.04684	9			0.1349E-03					
56	7	E	57	8	E	911.05468	6			0.9743E-05					
18	13	E	19	14	E	911.25614	-2			0.1219E-03					
37	10	E	38	11	E	911.35838	2			0.1043E-03					
43	9	E	44	10	E	911.63658	1			0.6414E-04					
24	12	E	25	13	E	911.66305	3			0.1537E-03					
49	8	A_1	50	9	A_2	911.88423	-3			0.3149E-04					
49	8	A_2	50	9	A_1	911.88423	-3			0.3149E-04					
30	11	A_2	31	12	A_1	912.02898	7	.287560E-03	2.5	0.1538E-03	-6.94	2.447E-04	.268E+00		1.3
30	11	A_1	31	12	A_2	912.02898	7			0.1538E-03					
55	7	E	56	8	E	912.10398	11			0.1239E-04					
17	13	E	18	14	E	912.19910	-10			0.1321E-03					
36	10	E	37	11	E	912.35758	6			0.1220E-03					
23	12	E	24	13	E	912.62452	1			0.1701E-03					
42	9	E	43	10	E	912.65214	1			0.7702E-04					
48	8	A_1	49	9	A_2	912.91566	2			0.3883E-04					
48	8	A_2	49	9	A_1	912.91566	2			0.3883E-04					
29	11	A_2	30	12	A_1	913.00822	7	.370203E-03	3.0	0.1744E-03	5.76	8.392E-05			1.0
29	11	A_1	30	12	A_2	913.00822	7			0.1744E-03					
16	13	E	17	14	E	913.13898	-11			0.1426E-03					
54	7	E	55	8	E	913.15040	-15			0.1569E-04					
35	10	E	36	11	E	913.35386	1			0.1420E-03					
22	12	E	23	13	E	913.58295	1			0.1875E-03					
41	9	E	42	10	E	913.66495	1			0.9206E-04					
47	8	A_2	48	9	A_1	913.94436	-1			0.4766E-04					
47	8	A_1	48	9	A_2	913.94436	-1			0.4766E-04					
28	11	A_2	29	12	A_1	913.98448	5	.388979E-03	1.5	0.1970E-03	-1.29	3.686E-04	.344E+00	.223E+01	1.1
28	11	A_1	29	12	A_2	913.98448	5			0.1970E-03					
15	13	E	16	14	E	914.07565	-16			0.1535E-03					
53	7	E	54	8	E	914.19484	18			0.1978E-04					
34	10	E	35	11	E	914.34736	3			0.1646E-03					
21	12	E	22	13	E	914.53833	1			0.2059E-03					
40	9	E	41	10	E	914.67503	3			0.1095E-03					
31	15	E	31	16	E	914.84283	-19			0.3445E-05					
27	11	A_2	28	12	A_1	914.95780	4	.446083E-03	0.9	0.2215E-03	.69	2.013E-04	-.441E+00		1.3
27	11	A_1	28	12	A_2	914.95780	4			0.2215E-03					
46	8	A_1	47	9	A_2	914.97045	3			0.5822E-04					
46	8	A_2	47	9	A_1	914.97045	3			0.5822E-04					
14	13	E	15	14	E	915.00916	-21			0.1648E-03					
29	15	E	29	16	E	915.05424	2			0.4077E-05					
28	15	E	28	16	E	915.15462	2			0.4376E-05					
52	7	E	53	8	E	915.23614	-5			0.2482E-04					
27	15	E	27	16	E	915.25165	15			0.4652E-05					
33	10	E	34	11	E	915.33795	1			0.1899E-03					
25	15	E	25	16	E	915.43542	53			0.5082E-05					
58	6	E	59	7	E	915.47455	13			0.8518E-05					
20	12	E	21	13	E	915.49064	2			0.2250E-03					
24	15	E	24	16	E	915.52151	13			0.5209E-05					
23	15	E	23	16	E	915.60477	38			0.5254E-05					
39	9	E	40	10	E	915.68271	43			0.1297E-03					
21	15	E	21	16	E	915.75991	-10			0.5022E-05					
19	15	E	19	16	E	915.90191	14			0.4214E-05					
26	11	A_2	27	12	A_1	915.92819	8	.492470E-03	1.2	0.2479E-03	-.69	3.606E-04	-.411E+00	.268E+00	1.2
26	11	A_1	27	12	A_2	915.92819	8			0.2479E-03					
13	13	E	14	14	E	915.93948	-24			0.1764E-03					
18	15	E	18	16	E	915.96716	-29			0.3531E-05					
45	8	A_2	46	9	A_1	915.99379	-1			0.7081E-04					
45	8	A_1	46	9	A_2	915.99379	-1			0.7081E-04					
17	15	E	17	16	E	916.02925	-42			0.2623E-05					
51	7	E	52	8	E	916.27531	20			0.3100E-04					
32	10	E	33	11	E	916.32573	5			0.2180E-03					
19	12	E	20	13	E	916.43982	-2			0.2450E-03					

(cont.)

J	K_a	Γ	J'	K'_a	Γ'	$\nu^{a)}$	$\delta_\nu{}^{b)}$	$S_\nu^{\rm exp}(294.45)^{c)}$	$\Delta_S{}^{d)}$	$S_\nu^{\rm calc}(294.45)^{e)}$	$\delta_S{}^{f)}$	$\Gamma_0{}^{g)}$	$\Delta_2{}^{g)}$	$\nu_{\rm VC}{}^{g)}$	$R^{h)}$
1			2			3	4	5	6	7	8	9	10	11	12
38	9	E	39	10	E	916.68680	2			0.1529E-03					
25	11	A_2	26	12	A_1	916.89553	5	.516927E-03	1.2	0.2763E-03	-6.89	1.721E-04	-.128E+01		1.4
25	11	A_1	26	12	A_2	916.89553	5			0.2763E-03					
44	8	A_1	45	9	A_2	917.01450	2			0.8572E-04					
44	8	A_2	45	9	A_1	917.01450	2			0.8572E-04					
31	10	E	32	11	E	917.31074	21	.247671E-03	2.5	0.2492E-03	-.61	6.942E-04			1.0
50	7	E	51	8	E	917.31074	-69			0.3854E-04					
18	12	E	19	13	E	917.38592	-4			0.2658E-03					
56	6	E	57	7	E	917.58005	18			0.1395E-04					
37	9	E	38	10	E	917.68853	4			0.1794E-03					
24	11	A_2	25	12	A_1	917.85987	3	.607182E-03	1.0	0.3065E-03	-.97	2.735E-04	-.557E+00		1.4
24	11	A_1	25	12	A_2	917.85987	3			0.3065E-03					
43	8	A_2	44	9	A_1	918.03246	1			0.1033E-03					
43	8	A_1	44	9	A_2	918.03246	1			0.1033E-03					
30	10	E	31	11	E	918.29255	7	.293817E-03	4.8	0.2835E-03	3.50	2.128E-04			1.0
17	12	E	18	13	E	918.32892	-5			0.2872E-03					
49	7	E	50	8	E	918.34515	3			0.4770E-04					
55	6	E	56	7	E	918.62854	-24			0.1773E-04					
36	9	E	37	10	E	918.68742	4			0.2096E-03					
23	11	A_2	24	12	A_1	918.82124	4	.638351E-03	1.9	0.3386E-03	-6.08	3.950E-04	-.578E+00		1.3
23	11	A_1	24	12	A_2	918.82124	4			0.3386E-03					
42	8	A_2	43	9	A_1	919.04775	5	.266540E-03	1.6	0.1239E-03	7.03	2.480E-04	-.125E+01		2.1
42	8	A_1	43	9	A_2	919.04775	5			0.1239E-03					
16	12	E	17	13	E	919.26927	43			0.3093E-03					
48	7	E	49	8	E	919.37618	0			0.5876E-04					
54	6	E	55	7	E	919.67546	32			0.2243E-04					
35	9	E	36	10	E	919.68349	3			0.2436E-03					
22	11	A_2	23	12	A_1	919.77964	11	.784661E-03	1.2	0.3724E-03	5.08	4.778E-04	-.104E+00		1.3
22	11	A_1	23	12	A_2	919.77964	11			0.3724E-03					
41	8	A_2	42	9	A_1	920.06021	-1			0.1479E-03					
41	8	A_1	42	9	A_2	920.06021	-1			0.1479E-03					
15	12	E	16	13	E	920.20544	-14	.344599E-03	1.9	0.3319E-03	3.68	1.546E-04	-.151E+01		1.5
28	10	E	29	11	E	920.24762	1	.345733E-03	2.1	0.3620E-03	-4.72	2.022E-04			1.0
47	7	E	48	8	E	920.40454	-5			0.7206E-04					
34	9	E	35	10	E	920.67676	7			0.2819E-03					
53	6	E	54	7	E	920.71901	7			0.2825E-04					
21	11	A_2	22	12	A_1	920.73481	0	0.809794E-03	1.0	0.4079E-03	0.74	2.221E-04	-.428E+00	-.357E+00	1.4
21	11	A_1	22	12	A_2	920.73481	0			0.4079E-03					
59	5	A_2	60	6	A_1	921.00523	12			0.8954E-05					
59	5	A_1	60	6	A_2	921.00523	12			0.8954E-05					
40	8	A_2	41	9	A_1	921.07000	0	.337152E-03	3.3	0.1758E-03	-4.29	1.401E-04			1.0
40	8	A_1	41	9	A_2	921.07000	0			0.1758E-03					
14	12	E	15	13	E	921.13902	-14	.376616E-03	1.2	0.3552E-03	5.69	2.053E-04			1.0
28	14	A_1	28	15	A_2	921.16341	-47			0.1422E-04					
28	14	A_2	28	15	A_1	921.16341	-47			0.1422E-04					
27	10	E	28	11	E	921.22085	8	.415145E-03	1.1	0.4063E-03	2.12	3.241E-04		-.838E+00	1.2
27	14	A_2	27	15	A_1	921.25962	-38			0.1517E-04					
27	14	A_1	27	15	A_2	921.25962	-38			0.1517E-04					
46	7	E	47	8	E	921.43032	-3			0.8795E-04					
25	14	A_2	25	15	A_1	921.44167	-23			0.1677E-04					
25	14	A_1	25	15	A_2	921.44167	-23			0.1677E-04					
24	14	A_1	24	15	A_2	921.52765	-3			0.1733E-04					
24	14	A_2	24	15	A_1	921.52765	-3			0.1733E-04					
23	14	A_1	23	15	A_2	921.61041	38			0.1767E-04					
23	14	A_2	23	15	A_1	921.61041	38			0.1767E-04					
33	9	E	34	10	E	921.66709	1			0.3248E-03					
20	11	A_2	21	12	A_1	921.68712	7	.878167E-03	0.6	0.4449E-03	-1.32	3.782E-04	-.821E-01	-.461E+00	1.2
20	11	A_1	21	12	A_2	921.68712	7			0.4449E-03					
52	6	E	53	7	E	921.76020	4			0.3542E-04					
21	14	A_2	21	15	A_1	921.76440	0			0.1745E-04					
21	14	A_1	21	15	A_2	921.76440	0			0.1745E-04					
20	14	A_1	20	15	A_2	921.83651	9			0.1678E-04					
20	14	A_2	20	15	A_1	921.83651	9			0.1678E-04					
19	14	A_1	19	15	A_2	921.90520	19			0.1564E-04					
19	14	A_2	19	15	A_1	921.90520	19			0.1564E-04					
18	14	A_1	18	15	A_2	921.96996	-21			0.1395E-04					
18	14	A_2	18	15	A_1	921.96996	-21			0.1395E-04					
17	14	A_2	17	15	A_1	922.03187	-1			0.1164E-04					
17	14	A_1	17	15	A_2	922.03187	-1			0.1164E-04					
58	5	A_1	59	6	A_2	922.06164	31			0.1153E-04					
58	5	A_2	59	6	A_1	922.06164	31			0.1153E-04					
13	12	E	14	13	E	922.06942	-15	.405038E-03	2.0	0.3790E-03	6.42	2.379E-04	-.125E+01	-.191E+00	1.4
39	8	A_2	40	9	A_1	922.07702	1	.434997E-03	2.4	0.2080E-03	4.39	2.831E-04	-.133E+01	-.248E+00	1.4
39	8	A_1	40	9	A_2	922.07702	1			0.2080E-03					
15	14	A_1	15	15	A_2	922.14468	-34			0.4771E-05					
15	14	A_2	15	15	A_1	922.14468	-34			0.4771E-05					
26	10	E	27	11	E	922.19101	4	.465330E-03	1.5	0.4540E-03	2.43	1.960E-04			1.0
45	7	E	46	8	E	922.45343	0			0.1069E-03					
19	11	A_2	20	12	A_1	922.63621	-1	.100429E-02	0.9	0.4832E-03	3.77	3.309E-04	-.674E+00	-.234E+00	1.6
19	11	A_1	20	12	A_2	922.63621	-1			0.4832E-03					
32	9	E	33	10	E	922.65463	2			0.3724E-03					
51	6	E	52	7	E	922.79868	-10			0.4420E-04					

(cont.)

J	K_a	Γ	J'	K'_a	Γ'	$\nu^{a)}$	$\delta_\nu{}^{b)}$	$S_\nu^{\rm exp}(294.45)^{c)}$	$\Delta_S{}^{d)}$	$S_\nu^{\rm calc}(294.45)^{e)}$	$\delta_S{}^{f)}$	$\Gamma_0{}^{g)}$	$\Delta_2{}^{g)}$	$\nu_{\rm VC}{}^{g)}$	$R^{h)}$
1			2			3	4	5	6	7	8	9	10	11	12
12	12	E	13	13	E	922.99663	-18	.433811E-03	3.1	0.4036E-03	6.97	3.757E-04	-.607E+00		1.1
38	8	A_2	39	9	A_1	923.08125	-1	.457364E-03	10.4	0.2449E-03	-7.07	1.529E-04			1.0
38	8	A_1	39	9	A_2	923.08125	-1			0.2449E-03					
57	5	A_2	58	6	A_1	923.11512	8			0.1477E-04					
57	5	A_1	58	6	A_2	923.11512	8			0.1477E-04					
25	10	E	26	11	E	923.15824	4	.470968E-03	2.0	0.5050E-03	-7.22	2.248E-04			1.0
44	7	E	45	8	E	923.47384	1			0.1292E-03					
18	11	A_2	19	12	A_1	923.58228	-2	.104996E-02	1.2	0.5228E-03	.41	2.714E-04	-.801E+00	-.222E+00	1.3
18	11	A_1	19	12	A_2	923.58228	-2			0.5228E-03					
31	9	E	32	10	E	923.63928	2			0.4250E-03					
50	6	E	51	7	E	923.83478	-3			0.5490E-04					
37	8	A_2	38	9	A_1	924.08272	0	.543839E-03	1.4	0.2870E-03	-5.53	2.472E-04			1.0
37	8	A_1	38	9	A_2	924.08272	0			0.2870E-03					
24	10	E	25	11	E	924.12249	3	.546516E-03	1.5	0.5592E-03	-2.32	3.117E-04			1.0
56	5	A_2	57	6	A_1	924.16625	3			0.1885E-04					
56	5	A_1	57	6	A_2	924.16625	3			0.1885E-04					
62	4	E	63	5	E	924.47007	72			0.5255E-05					
43	7	E	44	8	E	924.49159	6			0.1556E-03					
17	11	A_2	18	12	A_1	924.52525	-5	.110948E-02	0.7	0.5635E-03	-1.57	3.486E-04	-.354E+00		1.1
17	11	A_1	18	12	A_2	924.52525	-5			0.5635E-03					
30	9	E	31	10	E	924.62105	2	.472879E-03	1.7	0.4828E-03	-2.10	1.858E-04			1.0
49	6	E	50	7	E	924.86821	-1			0.6789E-04					
36	8	A_2	37	9	A_1	925.08174	35			0.3347E-03					
36	8	A_1	37	9	A_2	925.08174	35			0.3347E-03					
55	5	A_2	56	6	A_1	925.21487	1			0.2394E-04					
55	5	A_1	56	6	A_2	925.21487	1			0.2394E-04					
16	11	A_2	17	12	A_1	925.46515	-3	.125062E-02	0.9	0.6050E-03	3.24	2.502E-04	-.851E+00		1.8
16	11	A_1	17	12	A_2	925.46515	-3			0.6050E-03					
42	7	E	43	8	E	925.50650	-2			0.1864E-03					
29	9	E	30	10	E	925.59995	6	.540235E-03	1.8	0.5460E-03	-1.06	5.708E-04	-.442E+00	-.198E+00	1.1
40	13	E	40	14	E	925.82656	-50			0.9806E-05					
48	6	E	49	7	E	925.89905	4			0.8356E-04					
39	13	E	39	14	E	925.96334	-31			0.1138E-04					
22	10	E	23	11	E	926.04201	5	.662828E-03	1.3	0.6766E-03	-2.08	3.015E-04			1.0
35	8	A_2	36	9	A_1	926.07727	3	.712265E-03	1.4	0.3887E-03	-9.14	1.928E-04			1.0
35	8	A_1	36	9	A_2	926.07727	3			0.3887E-03					
38	13	E	38	14	E	926.09626	-54			0.1313E-04					
37	13	E	37	14	E	926.22622	-28			0.1507E-04					
54	5	A_2	55	6	A_1	926.26099	3			0.3026E-04					
54	5	A_1	55	6	A_2	926.26099	3			0.3026E-04					
36	13	E	36	14	E	926.35258	-20			0.1718E-04					
15	11	A_2	16	12	A_1	926.40190	-4	.131638E-02	0.4	0.6475E-03	1.63	5.728E-04	.482E+00	.269E+01	1.1
15	11	A_1	16	12	A_2	926.40190	-4			0.6475E-03					
35	13	E	35	14	E	926.47546	-16			0.1948E-04					
41	7	E	42	8	E	926.51882	3			0.2223E-03					
28	9	E	29	10	E	926.57590	6	.572551E-03	1.2	0.6145E-03	-7.33	1.384E-04			1.0
60	4	E	61	5	E	926.59445	64			0.8896E-05					
34	13	E	34	14	E	926.59445	-58			0.2194E-04					
33	13	E	33	14	E	926.71089	-12			0.2457E-04					
32	13	E	32	14	E	926.82352	-4			0.2732E-04					
47	6	E	48	7	E	926.92721	6			0.1024E-03					
31	13	E	31	14	E	926.93225	-43			0.3018E-04					
21	10	E	22	11	E	926.99722	4	.714553E-03	1.5	0.7394E-03	-3.48	5.546E-04			1.0
30	13	E	30	14	E	927.03848	11			0.3310E-04					
34	8	A_2	35	9	A_1	927.07031	3	.850043E-03	0.8	0.4492E-03	-5.69	2.874E-04	.552E+00	.706E+00	1.1
34	8	A_1	35	9	A_2	927.07031	3			0.4492E-03					
29	13	E	29	14	E	927.14071	7			0.3604E-04					
28	13	E	28	14	E	927.23969	20			0.3893E-04					
53	5	A_1	54	6	A_2	927.30452	3			0.3808E-04					
53	5	A_2	54	6	A_1	927.30452	3			0.3808E-04					
27	13	E	27	14	E	927.33549	57			0.4170E-04					
14	11	A_2	15	12	A_1	927.33549	-8	.146883E-02	0.5	0.6906E-03	5.96	3.575E-04	.198E-01	-.332E+00	1.1
14	11	A_1	15	12	A_2	927.33549	-8			0.6906E-03					
26	13	E	26	14	E	927.42713	20			0.4428E-04					
25	13	E	25	14	E	927.51531	-21			0.4657E-04					
40	7	E	41	8	E	927.52836	4	.252855E-03	4.0	0.2639E-03	-4.37	3.581E-04			1.0
27	9	E	28	10	E	927.54894	7	.654266E-03	0.9	0.6886E-03	-5.24	3.240E-04			1.0
24	13	E	24	14	E	927.60086	17			0.4846E-04					
59	4	E	60	5	E	927.65233	1			0.1150E-04					
23	13	E	23	14	E	927.68251	7			0.4983E-04					
22	13	E	22	14	E	927.76079	0			0.5056E-04					
20	13	E	20	14	E	927.90712	-11			0.4949E-04					
20	10	E	21	11	E	927.94935	-2	.804244E-03	0.7	0.8046E-03	-.04	1.382E-04	.168E+01	.291E+00	1.2
19	13	E	19	14	E	927.97533	0			0.4737E-04					
18	13	E	18	14	E	928.03979	-23			0.4396E-04					
33	8	A_2	34	9	A_1	928.06052	4	.108118E-02	1.2	0.5167E-03	4.41	4.339E-04	.640E+00	.378E+00	1.2
33	8	A_1	34	9	A_2	928.06052	4			0.5167E-03					
17	13	E	17	14	E	928.10132	2			0.3907E-04					
16	13	E	16	14	E	928.15908	-9			0.3247E-04					
15	13	E	15	14	E	928.21352	-11			0.2395E-04					
13	11	A_2	14	12	A_1	928.26600	-4	.152701E-02	0.4	0.7346E-03	3.79	3.075E-04	.651E-01	-.390E+00	1.2
13	11	A_1	14	12	A_2	928.26600	-4			0.7346E-03					

(cont.)

J	K_a	Γ	J'	K'_a	Γ'	$\nu^{a)}$	$\delta_\nu{}^{b)}$	$S_\nu^{\rm exp}(294.45)^{c)}$	$\Delta_S{}^{d)}$	$S_\nu^{\rm calc}(294.45)^{e)}$	$\delta_S{}^{f)}$	$\Gamma_0{}^{g)}$	$\Delta_2{}^{g)}$	$\nu_{\rm VC}{}^{g)}$	$R^{h)}$
1			2			3	4	5	6	7	8	9	10	11	12
52	5	A_2	53	6	A_1	928.34551	5			0.4770E-04					
52	5	A_1	53	6	A_2	928.34551	5			0.4770E-04					
26	9	E	27	10	E	928.51898	3	.728541E-03	2.2	0.7680E-03	-5.41	2.943E-04	.420E+00		1.1
39	7	E	40	8	E	928.53516	5	.301795E-03	2.2	0.3118E-03	-3.32	2.845E-04			1.0
58	4	E	59	5	E	928.70856	24			0.1479E-04					
19	10	E	20	11	E	928.89853	3	.884679E-03	1.2	0.8719E-03	1.45	3.301E-04	-.167E+00	-.468E+00	1.1
45	6	E	46	7	E	928.97550	2			0.1515E-03					
32	8	A_2	33	9	A_1	929.04787	4	.111890E-02	0.4	0.5916E-03	-5.75	3.270E-04	.775E-01	.529E+00	1.3
32	8	A_1	33	9	A_2	929.04787	4			0.5916E-03					
12	11	A_2	13	12	A_1	929.19334	-2	.164842E-02	0.6	0.7794E-03	5.43	2.911E-04	.742E-01	-.234E+00	1.1
12	11	A_1	13	12	A_2	929.19334	-2			0.7794E-03					
51	5	A_2	52	6	A_1	929.38390	6			0.5947E-04					
51	5	A_1	52	6	A_2	929.38390	6			0.5947E-04					
25	9	E	26	10	E	929.48611	4	.799063E-03	0.7	0.8526E-03	-6.70	2.741E-04	-.122E+00	-.293E+00	1.1
38	7	E	39	8	E	929.53921	7	.340294E-03	2.3	0.3667E-03	-7.77	3.186E-04		-.112E+01	1.2
57	4	E	58	5	E	929.76182	0			0.1894E-04					
18	10	E	19	11	E	929.84460	3	.103319E-02	1.0	0.9409E-03	8.93	4.704E-04	.539E+00	.176E+00	1.2
44	6	E	45	7	E	929.99568	4			0.1831E-03					
31	8	A_2	32	9	A_1	930.03237	5	.131552E-02	1.0	0.6743E-03	-2.51	3.497E-04			1.0
31	8	A_1	32	9	A_2	930.03237	5			0.6743E-03					
11	11	A_2	12	12	A_1	930.11743	-6	.175755E-02	0.8	0.8255E-03	6.06	2.540E-04			1.0
11	11	A_1	12	12	A_2	930.11743	-6			0.8255E-03					
50	5	A_1	51	6	A_2	930.41966	4			0.7381E-04					
50	5	A_2	51	6	A_1	930.41966	4			0.7381E-04					
24	9	E	25	10	E	930.45028	5	.946427E-03	1.3	0.9423E-03	.43	3.703E-04	-.323E+00	-.188E+00	1.1
37	7	E	38	8	E	930.54047	7	.453452E-03	2.9	0.4293E-03	5.33	3.195E-04	-.559E+00		1.1
48	12	E	48	13	E	930.76852	-15			0.5815E-05					
17	10	E	18	11	E	930.78760	3	.104442E-02	1.2	0.1011E-02	3.17	2.846E-04	-.100E+01	-.197E+00	1.4
56	4	E	57	5	E	930.81274	-5			0.2414E-04					
47	12	E	47	13	E	930.93180	2			0.7043E-05					
30	8	A_2	31	9	A_1	931.01389	-4	.183218E-02	0.7	0.7648E-03	4.50	3.504E-04	-.520E+00	-.165E+00	1.3
30	8	A_1	31	9	A_2	931.01389	-4			0.7648E-03					
46	12	E	46	13	E	931.09111	-36			0.8486E-05					
45	12	E	45	13	E	931.24779	7			0.1017E-04					
44	12	E	44	13	E	931.40097	42			0.1213E-04					
23	9	E	24	10	E	931.41148	7	.997183E-03	0.8	0.1037E-02	-3.97	3.723E-04	-.292E+00	.510E+00	1.2
49	5	A_2	50	6	A_1	931.45284	4			0.9119E-04					
49	5	A_1	50	6	A_2	931.45284	4			0.9119E-04					
25	16	E	24	17	E	931.53323	26			0.1554E-06					
36	7	E	37	8	E	931.53891	4	.480814E-03	1.3	0.5002E-03	-4.02	2.512E-04	-.411E+00		1.2
43	12	E	43	13	E	931.54995	0			0.1439E-04					
42	12	E	42	13	E	931.69571	-21			0.1698E-04					
16	10	E	17	11	E	931.72748	1	.111691E-02	1.1	0.1083E-02	3.04	3.583E-04			1.0
41	12	E	41	13	E	931.83842	-5			0.1992E-04					
40	12	E	40	13	E	931.97769	9			0.2325E-04					
29	8	A_2	30	9	A_1	931.99273	7	.171710E-02	0.5	0.8635E-03	-.58	2.517E-04	.226E+00	-.131E+00	1.3
29	8	A_1	30	9	A_2	931.99273	7			0.8635E-03					
42	6	E	43	7	E	932.02791	3	.258155E-03	1.7	0.2635E-03	-2.08	3.513E-04	.106E+01	.592E+00	1.6
38	12	E	38	13	E	932.24560	0			0.3116E-04					
22	9	E	23	10	E	932.36965	5	.108060E-02	1.4	0.1136E-02	-5.08	3.023E-04	.424E+00		1.1
48	5	A_1	49	6	A_2	932.48341	5			0.1121E-03					
48	5	A_2	49	6	A_1	932.48341	5			0.1121E-03					
36	12	E	36	13	E	932.50010	15			0.4082E-04					
35	7	E	36	8	E	932.53460	6	.540429E-03	0.9	0.5800E-03	-7.32	3.271E-04	-.226E+00		1.1
35	12	E	35	13	E	932.62209	8			0.4631E-04					
15	10	E	16	11	E	932.66429	2	.111624E-02	1.1	0.1155E-02	-3.51	1.891E-04		-.704E+00	1.1
34	12	E	34	13	E	932.74075	10			0.5223E-04					
33	12	E	33	13	E	932.85600	11			0.5854E-04					
54	4	E	55	5	E	932.90708	-3			0.3869E-04					
28	8	A_2	29	9	A_1	932.96853	5	.197517E-02	0.6	0.9704E-03	-1.56	3.810E-04			1.0
28	8	A_1	29	9	A_2	932.96853	5			0.9704E-03					
32	12	E	32	13	E	932.96853	81			0.6520E-04					
41	6	E	42	7	E	933.04001	7	.343996E-03	3.2	0.3139E-03	8.73	3.439E-04	.951E+00	.341E+00	1.2
31	12	E	31	13	E	933.07626	11			0.7214E-04					
30	12	E	30	13	E	933.18130	13			0.7928E-04					
29	12	E	29	13	E	933.28302	22			0.8652E-04					
60	3	E	61	4	E	933.30011	11			0.1089E-04					
21	9	E	22	10	E	933.32483	6	.124285E-02	1.0	0.1238E-02	.38	2.300E-04	-.103E+00	-.400E+00	1.1
28	12	E	28	13	E	933.38106	4			0.9373E-04					
27	12	E	27	13	E	933.47584	0			0.1008E-03					
47	5	A_2	48	6	A_1	933.51132	4			0.1372E-03					
47	5	A_1	48	6	A_2	933.51132	4			0.1372E-03					
34	7	E	35	8	E	933.52747	7	.639358E-03	1.8	0.6694E-03	-4.70	2.092E-04	.627E+00	.418E+00	1.1
26	12	E	26	13	E	933.56734	7			0.1074E-03					
14	10	E	15	11	E	933.59794	-1	.131414E-02	0.6	0.1228E-02	6.52	3.497E-04	-.125E+00	-.877E-01	1.1
25	12	E	25	13	E	933.65503	-27			0.1135E-03					
24	12	E	24	13	E	933.73997	4			0.1188E-03					
23	12	E	23	13	E	933.82120	3			0.1231E-03					
22	12	E	22	13	E	933.89879	-23			0.1260E-03					
27	8	A_2	28	9	A_1	933.94146	7	.212687E-02	0.9	0.1085E-02	-2.07	2.756E-04	-.271E+00	-.363E+00	1.2
27	8	A_1	28	9	A_2	933.94146	7			0.1085E-02					
53	4	E	54	5	E	933.95049	5			0.4864E-04					

(cont.)

J	K_a	Γ	J'	K'_a	Γ'	$\nu^{a)}$	$\delta_\nu{}^{b)}$	$S_\nu^{\rm exp}(294.45)^{c)}$	$\Delta_S{}^{d)}$	$S_\nu^{\rm calc}(294.45)^{e)}$	$\delta_S{}^{f)}$	$\Gamma_0{}^{g)}$	$\Delta_2{}^{g)}$	$\nu_{\rm VC}{}^{g)}$	$R^{h)}$
1			2			3	4	5	6	7	8	9	10	11	12
21	12	E	21	13	E	933.97346	-1			0.1272E-03					
20	12	E	20	13	E	934.04476	22			0.1265E-03					
40	6	E	41	7	E	934.04930	3			0.3723E-03					
19	12	E	19	13	E	934.11229	8			0.1235E-03					
18	12	E	18	13	E	934.17642	-8			0.1177E-03					
17	12	E	17	13	E	934.23733	-6			0.1088E-03					
20	9	E	21	10	E	934.27700	7	.127591E-02	1.0	0.1344E-02	-5.35	2.312E-04		-.511E+00	1.1
16	12	E	16	13	E	934.29475	-15			0.9634E-04					
15	12	E	15	13	E	934.34883	-19			0.7981E-04					
59	3	E	60	4	E	934.35872	35			0.1406E-04					
14	12	E	14	13	E	934.39966	-10			0.5868E-04					
13	12	E	13	13	E	934.44682	-29			0.3232E-04					
33	7	E	34	8	E	934.51752	8	.719970E-03	1.9	0.7690E-03	-6.81	1.184E-04		-.989E+00	1.2
13	10	E	14	11	E	934.52856	7	.141607E-02	1.0	0.1302E-02	8.04	3.540E-04	.361E+00	-.237E+00	1.2
46	5	A_2	47	6	A_1	934.53659	3	.352643E-03	2.7	0.1672E-03	5.17	3.101E-04			1.2
46	5	A_1	47	6	A_2	934.53659	3			0.1672E-03					
26	8	A_2	27	9	A_1	934.91145	8	.226675E-02	0.6	0.1208E-02	-6.62	2.682E-04	.824E-01	-.323E+00	1.1
26	8	A_1	27	9	A_2	934.91145	8			0.1208E-02					
52	4	E	53	5	E	934.99124	3			0.6088E-04					
39	6	E	40	7	E	935.05592	6	.455010E-03	2.8	0.4394E-03	3.43	2.942E-04	-.178E+00		1.0
19	9	E	20	10	E	935.22611	6	.146362E-02	1.1	0.1453E-02	.72	4.126E-04		-.437E+00	1.0
58	3	E	59	4	E	935.41437	13			0.1807E-04					
12	10	E	13	11	E	935.45591	2	.143054E-02	0.3	0.1377E-02	3.77	3.134E-04	-.280E+00		2.2
32	7	E	33	8	E	935.50473	9	.875612E-03	0.8	0.8793E-03	-.42	2.913E-04	-.218E+00	-.158E+00	1.2
45	5	A_2	46	6	A_1	935.55925	7	.406831E-03	1.6	0.2028E-03	.32	2.125E-04		-.199E+00	1.0
45	5	A_1	46	6	A_2	935.55925	7			0.2028E-03					
56	11	A_2	56	12	A_1	935.56990	19			0.2244E-05					
56	11	A_1	56	12	A_2	935.56990	19			0.2244E-05					
55	11	A_1	55	12	A_2	935.75933	3			0.2827E-05					
55	11	A_2	55	12	A_1	935.75933	3			0.2827E-05					
25	8	A_2	26	9	A_1	935.87851	10	.251476E-02	0.7	0.1339E-02	-6.50	3.617E-04	-.275E+00		1.3
25	8	A_1	26	9	A_2	935.87851	10			0.1339E-02					
51	4	E	52	5	E	936.02931	-8			0.7584E-04					
38	6	E	39	7	E	936.05978	8	.519504E-03	1.0	0.5161E-03	.65	1.470E-04	-.361E+00	-.174E+00	1.3
53	11	A_2	53	12	A_1	936.12792	-25			0.4421E-05					
53	11	A_1	53	12	A_2	936.12792	-25			0.4421E-05					
18	9	E	19	10	E	936.17218	7	.149318E-02	0.4	0.1564E-02	-4.74	3.711E-04	-.680E-01	-.305E+00	1.1
52	11	A_1	52	12	A_2	936.30710	-36			0.5489E-05					
52	11	A_2	52	12	A_1	936.30710	-36			0.5489E-05					
11	10	E	12	11	E	936.38019	6	.134604E-02	0.6	0.1452E-02	-7.89	1.396E-04		-.565E+00	1.2
57	3	E	58	4	E	936.46775	15			0.2312E-04					
31	7	E	32	8	E	936.48906	7	.100242E-02	1.2	0.1001E-02	.19	4.133E-04			1.0
44	5	A_2	45	6	A_1	936.57922	8	.497807E-03	1.3	0.2447E-03	1.68	6.262E-05		-.754E+00	1.2
44	5	A_1	45	6	A_2	936.57922	8			0.2447E-03					
49	11	A_2	49	12	A_1	936.82477	-3			0.1020E-04					
49	11	A_1	49	12	A_2	936.82477	-3			0.1020E-04					
24	8	A_2	25	9	A_1	936.84257	7	.297152E-02	0.3	0.1477E-02	.58	2.930E-04	-.247E+00	-.467E+00	1.4
24	8	A_1	25	9	A_2	936.84257	7			0.1477E-02					
48	11	A_2	48	12	A_1	936.99046	6			0.1241E-04					
48	11	A_1	48	12	A_2	936.99046	6			0.1241E-04					
37	6	E	38	7	E	937.06087	9	.603382E-03	3.7	0.6035E-03	-.01	1.332E-04			1.0
50	4	E	51	5	E	937.06497	-1			0.9404E-04					
17	9	E	18	10	E	937.11521	9	.164025E-02	0.7	0.1676E-02	-2.21	2.944E-04	-.797E+00	-.312E+00	1.7
47	11	A_1	47	12	A_2	937.15285	26			0.1503E-04					
47	11	A_2	47	12	A_1	937.15285	26			0.1503E-04					
10	10	E	11	11	E	937.30122	3	.162436E-02	0.6	0.1530E-02	5.83	2.777E-04	-.519E+00	.168E+00	1.9
46	11	A_1	46	12	A_2	937.31151	14			0.1810E-04					
46	11	A_2	46	12	A_1	937.31151	14			0.1810E-04					
30	7	E	31	8	E	937.47063	16	.111382E-02	1.1	0.1133E-02	-1.74	4.564E-04	-.339E+00	.927E+00	1.1
56	3	E	57	4	E	937.51847	3			0.2944E-04					
43	5	A_2	44	6	A_1	937.59650	9	.573650E-03	1.9	0.2940E-03	-2.50	2.968E-04	-.440E+00	.667E+00	1.1
43	5	A_1	44	6	A_2	937.59650	9			0.2940E-03					
44	11	A_1	44	12	A_2	937.61870	1			0.2587E-04					
44	11	A_2	44	12	A_1	937.61870	1			0.2587E-04					
43	11	A_1	43	12	A_2	937.76725	1			0.3070E-04					
43	11	A_2	43	12	A_1	937.76725	1			0.3070E-04					
23	8	A_2	24	9	A_1	937.80371	9	.317741E-02	0.6	0.1622E-02	-2.08	3.008E-04	-.932E+00		1.2
23	8	A_1	24	9	A_2	937.80371	9			0.1622E-02					
42	11	A_1	42	12	A_2	937.91237	-1			0.3622E-04					
42	11	A_2	42	12	A_1	937.91237	-1			0.3622E-04					
16	9	E	17	10	E	938.05508	3	.208008E-02	0.8	0.1790E-02	9.86	6.122E-04	-.394E+00	.223E+01	1.2
41	11	A_1	41	12	A_2	938.05508	96			0.4252E-04					
41	11	A_2	41	12	A_1	938.05508	96			0.4252E-04					
36	6	E	37	7	E	938.05896	-13			0.7022E-03					
49	4	E	50	5	E	938.09806	9			0.1161E-03					
40	11	A_2	40	12	A_1	938.19252	6			0.4965E-04					
40	11	A_1	40	12	A_2	938.19252	6			0.4965E-04					
39	11	A_1	39	12	A_2	938.32744	3			0.5766E-04					
39	11	A_2	39	12	A_1	938.32744	3			0.5766E-04					
29	7	E	30	8	E	938.44917	9	.129789E-02	1.9	0.1277E-02	1.58	2.988E-04	.701E+00		1.1
38	11	A_2	38	12	A_1	938.45906	11			0.6660E-04					
38	11	A_1	38	12	A_2	938.45906	11			0.6660E-04					

(cont.)

J	K_a	Γ	J'	K'_a	Γ'	$\nu^{a)}$	$\delta_\nu{}^{b)}$	$S_\nu^{\rm exp}(294.45)^{c)}$	$\Delta_S{}^{d)}$	$S_\nu^{\rm calc}(294.45)^{e)}$	$\delta_S{}^{f)}$	$\Gamma_0{}^{g)}$	$\Delta_2{}^{g)}$	$\nu_{\rm VC}{}^{g)}$	$R^{h)}$
	1			2		3	4	5	6	7	8	9	10	11	12
55	3	E	56	4	E	938.56678	4			0.3732E-04					
37	11	A_2	37	12	A_1	938.58716	5			0.7649E-04					
37	11	A_1	37	12	A_2	938.58716	5			0.7649E-04					
42	5	A_2	43	6	A_1	938.61110	10	.690356E-03	2.0	0.3515E-03	-1.84	3.033E-04			1.0
42	5	A_1	43	6	A_2	938.61110	10			0.3515E-03					
36	11	A_2	36	12	A_1	938.71195	8	.182968E-03	3.0	0.8736E-04	.00	6.532E-04	-.109E+01	.602E+00	1.1
36	11	A_1	36	12	A_2	938.71195	8			0.8736E-04					
22	8	A_2	23	9	A_1	938.76185	10	.349954E-02	0.7	0.1773E-02	-1.30	5.445E-04	.425E-01	.223E+01	1.1
22	8	A_1	23	9	A_2	938.76185	10			0.1773E-02					
35	11	A_2	35	12	A_1	938.83329	6	.215654E-03	3.0	0.9920E-04	.00	4.396E-04	.141E+01		1.3
35	11	A_1	35	12	A_2	938.83329	6			0.9920E-04					
34	11	A_2	34	12	A_1	938.95125	4	.238878E-03	4.2	0.1120E-03	6.24	6.754E-04			1.0
34	11	A_1	34	12	A_2	938.95125	4			0.1120E-03					
15	9	E	16	10	E	938.99202	13	.198031E-02	1.6	0.1904E-02	3.87	4.370E-04	-.761E+00	-.234E+00	1.3
35	6	E	36	7	E	939.05478	18			0.8133E-03					
33	11	A_2	33	12	A_1	939.06584	4	.270264E-03	2.2	0.1257E-03	7.00	1.580E-04			1.0
33	11	A_1	33	12	A_2	939.06584	4			0.1257E-03					
48	4	E	49	5	E	939.12856	22			0.1426E-03					
32	11	A_2	32	12	A_1	939.17705	4	.280786E-03	2.7	0.1402E-03	.16	1.284E-04			1.0
32	11	A_1	32	12	A_2	939.17705	4			0.1402E-03					
31	11	A_2	31	12	A_1	939.28487	4	.306596E-03	3.3	0.1553E-03	-1.33	5.619E-04			1.0
31	11	A_1	31	12	A_2	939.28487	4			0.1553E-03					
30	11	A_2	30	12	A_1	939.38931	5	.322368E-03	2.1	0.1710E-03	-6.12	5.122E-04			1.0
30	11	A_1	30	12	A_2	939.38931	5			0.1710E-03					
28	7	E	29	8	E	939.42487	7	.140043E-02	0.9	0.1433E-02	-2.34	3.233E-04	-.835E+00	-.320E+00	1.6
29	11	A_2	29	12	A_1	939.49007	-25			0.1871E-03					
29	11	A_1	29	12	A_2	939.49007	-25			0.1871E-03					
28	11	A_2	28	12	A_1	939.58806	7	.412116E-03	1.3	0.2032E-03	1.40	2.497E-04	-.537E+00		1.1
28	11	A_1	28	12	A_2	939.58806	7			0.2032E-03					
54	3	E	55	4	E	939.61245	-5			0.4709E-04					
41	5	A_2	42	6	A_1	939.62297	10	.831558E-03	1.0	0.4184E-03	-.62	1.217E-04	-.450E+00		1.6
41	5	A_1	42	6	A_2	939.62297	10			0.4184E-03					
27	11	A_2	27	12	A_1	939.68232	4	.426604E-03	1.7	0.2191E-03	-2.70	2.216E-04	-.379E+00		1.1
27	11	A_1	27	12	A_2	939.68232	4			0.2191E-03					
21	8	A_2	22	9	A_1	939.71701	12	.376963E-02	0.6	0.1928E-02	-2.31	3.888E-04			1.0
21	8	A_1	22	9	A_2	939.71701	12			0.1928E-02					
26	11	A_2	26	12	A_1	939.77323	3	.487903E-03	2.2	0.2344E-03	3.93	2.909E-04	-.658E+00		1.3
26	11	A_1	26	12	A_2	939.77323	3			0.2344E-03					
25	11	A_2	25	12	A_1	939.86073	-1	.514786E-03	1.4	0.2487E-03	3.38	4.609E-04			1.0
25	11	A_1	25	12	A_2	939.86073	-1			0.2487E-03					
14	9	E	15	10	E	939.92575	13	.190597E-02	0.9	0.2017E-02	-5.84	3.122E-04	.122E+00	-.467E+00	1.2
24	11	A_2	24	12	A_1	939.94488	-2	.544073E-03	1.6	0.2616E-03	3.85	2.990E-04	.234E+00		1.1
24	11	A_1	24	12	A_2	939.94488	-2			0.2616E-03					
23	11	A_2	23	12	A_1	940.02566	-3			0.2725E-03					
23	11	A_1	23	12	A_2	940.02566	-3			0.2725E-03					
34	6	E	35	7	E	940.04740	9	.909591E-03	0.9	0.9374E-03	-3.06	3.067E-04	-.180E+00	.206E+00	1.1
22	11	A_2	22	12	A_1	940.10309	-1	.593698E-03	1.7	0.2810E-03	5.34	2.247E-04			1.0
22	11	A_1	22	12	A_2	940.10309	-1			0.2810E-03					
47	4	E	48	5	E	940.15616	7			0.1744E-03					
21	11	A_2	21	12	A_1	940.17713	-1	.590183E-03	1.4	0.2864E-03	2.95	5.216E-04	.890E+00	.598E+00	1.1
21	11	A_1	21	12	A_2	940.17713	-1			0.2864E-03					
20	11	A_2	20	12	A_1	940.24784	3	.592271E-03	1.4	0.2880E-03	2.75	3.942E-04	-.672E+00	-.353E+00	1.2
20	11	A_1	20	12	A_2	940.24784	3			0.2880E-03					
19	11	A_2	19	12	A_1	940.31505	-6	.622525E-03	1.8	0.2852E-03	8.38	4.440E-04			1.0
19	11	A_1	19	12	A_2	940.31505	-6			0.2852E-03					
18	11	A_2	18	12	A_1	940.37907	3	.595550E-03	1.3	0.2772E-03	6.91	2.140E-05		-.101E+01	1.4
18	11	A_1	18	12	A_2	940.37907	3			0.2772E-03					
27	7	E	28	8	E	940.39772	11	.154292E-02	0.5	0.1600E-02	-3.72	2.571E-04	-.376E-01	-.381E+00	1.2
17	11	A_2	17	12	A_1	940.43958	-1	.528993E-03	1.8	0.2632E-03	.48	2.685E-04			1.0
17	11	A_1	17	12	A_2	940.43958	-1			0.2632E-03					
16	11	A_2	16	12	A_1	940.49674	-4	.495686E-03	0.9	0.2424E-03	2.18	4.227E-04	.405E+00	-.196E+00	1.4
16	11	A_1	16	12	A_2	940.49674	-4			0.2424E-03					
15	11	A_2	15	12	A_1	940.55060	0	.449344E-03	1.2	0.2139E-03	4.79	4.670E-04	-.480E+00	.103E+01	1.3
15	11	A_1	15	12	A_2	940.55060	0			0.2139E-03					
14	11	A_2	14	12	A_1	940.60103	-3	.385046E-03	4.3	0.1767E-03	8.24	4.776E-04			1.0
14	11	A_1	14	12	A_2	940.60103	-3			0.1767E-03					
40	5	A_2	41	6	A_1	940.63213	10	.101527E-02	0.7	0.4955E-03	2.38	3.070E-04			1.0
40	5	A_1	41	6	A_2	940.63213	10			0.4955E-03					
13	11	A_2	13	12	A_1	940.64812	-3	.242922E-03	2.1	0.1295E-03	-6.65	1.894E-04			1.0
13	11	A_1	13	12	A_2	940.64812	-3			0.1295E-03					
53	3	E	54	4	E	940.65595	26			0.5916E-04					
20	8	A_2	21	9	A_1	940.66911	9	.401425E-02	0.6	0.2089E-02	-4.06	4.216E-04	.235E+00	.401E+00	1.1
20	8	A_1	21	9	A_2	940.66911	9			0.2089E-02					
12	11	A_1	12	12	A_2	940.69185	-2			0.7120E-04					
12	11	A_2	12	12	A_1	940.69185	-2			0.7120E-04					
13	9	E	14	10	E	940.85636	12	.197919E-02	0.6	0.2131E-02	-7.65	1.988E-04		-.671E+00	1.3
33	6	E	34	7	E	941.03730	9	.108228E-02	1.0	0.1075E-02	.64	2.741E-04	-.476E-01		.9
59	2	A_1	60	3	A_2	941.11783	29			0.1647E-04					
46	4	E	47	5	E	941.18123	4			0.2122E-03					
26	7	E	27	8	E	941.36762	11	.173595E-02	0.9	0.1778E-02	-2.45	2.890E-04		-.522E+00	1.1
19	8	A_2	20	9	A_1	941.61825	12	.439970E-02	0.4	0.2252E-02	-2.36	2.942E-04		-.604E+00	1.8
19	8	A_1	20	9	A_2	941.61825	12			0.2252E-02					

(cont.)

J	K_a	Γ	J'	K'_a	Γ'	$\nu^{a)}$	$\delta_\nu{}^{b)}$	$S_\nu^{\mathrm{exp}}(294.45)^{c)}$	$\Delta_S{}^{d)}$	$S_\nu^{\mathrm{calc}}(294.45)^{e)}$	$\delta_S{}^{f)}$	$\Gamma_0{}^{g)}$	$\Delta_2{}^{g)}$	$\nu_{\mathrm{VC}}{}^{g)}$	$R^{h)}$
	1			2		3	4	5	6	7	8	9	10	11	12
39	5	A_2	40	6	A_1	941.63852	7	.118603E-02	0.7	0.5842E-03	1.49	3.781E-04			1.0
39	5	A_1	40	6	A_2	941.63852	7			0.5842E-03					
52	3	E	53	4	E	941.69645	13			0.7396E-04					
12	9	E	13	10	E	941.78383	11	.225566E-02	0.7	0.2244E-02	.53	3.895E-04	.233E+00	.449E-01	1.1
56	10	E	56	11	E	941.86184	21			0.4377E-05					
23	14	A_2	22	15	A_1	941.90926	-12			0.2333E-05					
23	14	A_1	22	15	A_2	941.90926	-12			0.2333E-05					
32	6	E	33	7	E	942.02436	8	.126693E-02	0.8	0.1228E-02	3.09	3.460E-04	-.547E+00	.359E+00	1.2
58	2	A_1	59	3	A_2	942.18150	24			0.2115E-04					
45	4	E	46	5	E	942.20366	2			0.2571E-03					
54	10	E	54	11	E	942.23518	-30			0.6909E-05					
25	7	E	26	8	E	942.33456	8	.193480E-02	0.4	0.1967E-02	-1.67	3.919E-04	-.144E+00	.145E+00	1.2
53	10	E	53	11	E	942.41772	43			0.8618E-05					
18	8	A_2	19	9	A_1	942.56432	11	.479878E-02	0.3	0.2417E-02	-.74	3.684E-04	-.250E+00	-.230E+00	1.8
18	8	A_1	19	9	A_2	942.56432	11			0.2417E-02					
52	10	E	52	11	E	942.59573	5			0.1070E-04					
38	5	A_2	39	6	A_1	942.64225	11	.134225E-02	0.5	0.6854E-03	-2.13	3.173E-04	-.462E+00	.266E+00	1.6
38	5	A_1	39	6	A_2	942.64225	11			0.6854E-03					
11	9	E	12	10	E	942.70832	26			0.2357E-02					
51	3	E	52	4	E	942.73448	11			0.9205E-04					
51	10	E	51	11	E	942.77091	25			0.1321E-04					
31	6	E	32	7	E	943.00860	9	.139777E-02	1.0	0.1395E-02	.19	2.300E-04			1.0
44	4	E	45	5	E	943.22353	10	.333536E-03	2.2	0.3099E-03	7.07	5.143E-04	-.815E+00	.169E+01	1.3
57	2	A_1	58	3	A_2	943.22739	38			0.2703E-04					
57	2	A_2	58	3	A_1	943.23449	2			0.2703E-04					
48	10	E	48	11	E	943.27521	1			0.2418E-04					
24	7	E	25	8	E	943.29866	16	.204301E-02	0.9	0.2165E-02	-5.99	3.802E-04			1.0
47	10	E	47	11	E	943.43656	-1			0.2928E-04					
25	14	A_2	24	15	A_1	943.50099	36			0.2676E-05					
25	14	A_1	24	15	A_2	943.50099	36			0.2676E-05					
17	8	A_2	18	9	A_1	943.50736	13	.515328E-02	0.3	0.2584E-02	-.27	2.921E-04	-.978E-02	-.506E+00	1.3
17	8	A_1	18	9	A_2	943.50736	13			0.2584E-02					
46	10	E	46	11	E	943.59462	6			0.3528E-04					
10	9	E	11	10	E	943.62936	12	.240307E-02	0.6	0.2471E-02	-2.83	2.778E-04	-.184E+00	-.404E+00	1.3
37	5	A_2	38	6	A_1	943.64320	14	.156793E-02	1.1	0.8004E-03	-2.10	1.486E-04		-.706E+00	1.1
37	5	A_1	38	6	A_2	943.64320	14			0.8004E-03					
45	10	E	45	11	E	943.74921	6			0.4229E-04					
50	3	E	51	4	E	943.76996	14			0.1140E-03					
44	10	E	44	11	E	943.90046	12			0.5044E-04					
18	13	E	17	14	E	943.94170	-42			0.3271E-05					
30	6	E	31	7	E	943.98997	9	.161190E-02	0.9	0.1578E-02	2.13	2.965E-04	-.360E+00	-.247E+00	1.2
43	10	E	43	11	E	944.04816	1			0.5985E-04					
42	10	E	42	11	E	944.19268	11			0.7065E-04					
43	4	E	44	5	E	944.24066	12	.373283E-03	2.1	0.3719E-03	.36	1.773E-04	-.351E+00		1.1
23	7	E	24	8	E	944.25972	15	.224948E-02	0.6	0.2372E-02	-5.47	4.011E-04	-.697E-01	.481E+00	1.1
56	2	A_2	57	3	A_1	944.27810	10			0.3438E-04					
56	2	A_1	57	3	A_2	944.28524	12			0.3438E-04					
41	10	E	41	11	E	944.33356	-5			0.8297E-04					
16	8	A_2	17	9	A_1	944.44731	12	.546535E-02	0.4	0.2750E-02	-.63	3.477E-04	.111E+00	-.223E+00	1.1
16	8	A_1	17	9	A_2	944.44731	12			0.2750E-02					
40	10	E	40	11	E	944.47127	0			0.9691E-04					
9	9	E	10	10	E	944.54737	13	.254830E-02	0.4	0.2589E-02	-1.59	2.825E-04	-.134E+00	-.374E+00	1.4
39	10	E	39	11	E	944.60554	0			0.1126E-03					
36	5	A_2	37	6	A_1	944.64135	13	.189208E-02	0.7	0.9302E-03	1.67	2.852E-04	-.256E+00	-.136E+00	1.3
36	5	A_1	37	6	A_2	944.64135	13			0.9302E-03					
38	10	E	38	11	E	944.73639	-4			0.1301E-03					
62	1	E	63	2	E	944.75463	6			0.8636E-05					
49	3	E	50	4	E	944.80279	11			0.1406E-03					
37	10	E	37	11	E	944.86403	8			0.1496E-03					
29	6	E	30	7	E	944.96850	12	.182864E-02	0.9	0.1775E-02	2.91	2.149E-04		-.574E+00	1.2
36	10	E	36	11	E	944.98813	4			0.1709E-03					
35	10	E	35	11	E	945.10883	-2			0.1943E-03					
22	7	E	23	8	E	945.21782	15	.248624E-02	1.0	0.2587E-02	-4.06	1.916E-04		-.952E+00	1.3
34	10	E	34	11	E	945.22612	-13			0.2196E-03					
42	4	E	43	5	E	945.25506	9	.451710E-03	2.1	0.4443E-03	1.65	4.160E-05			1.0
55	2	A_1	56	3	A_2	945.32649	2			0.4354E-04					
55	2	A_2	56	3	A_1	945.33309	-13			0.4354E-04					
33	10	E	33	11	E	945.34031	4			0.2467E-03					
15	8	A_2	16	9	A_1	945.38419	12	.596650E-02	0.6	0.2915E-02	2.29	3.482E-04	-.752E+00		1.2
15	8	A_1	16	9	A_2	945.38419	12			0.2915E-02					
32	10	E	32	11	E	945.45094	1			0.2755E-03					
31	10	E	31	11	E	945.55820	-1			0.3057E-03					
20	13	E	19	14	E	945.57256	-1			0.5274E-05					
35	5	A_2	36	6	A_1	945.63671	12	.220756E-02	0.8	0.1076E-02	2.53	1.602E-04		-.737E+00	1.7
35	5	A_1	36	6	A_2	945.63671	12			0.1076E-02					
30	10	E	30	11	E	945.66213	0			0.3372E-03					
29	10	E	29	11	E	945.76268	0			0.3696E-03					
48	3	E	49	4	E	945.83311	19			0.1725E-03					
28	10	E	28	11	E	945.85986	-2	.403329E-03	2.4	0.4023E-03	.26	3.009E-04	.655E+00		1.3
28	6	E	29	7	E	945.94386	-15			0.1989E-02					
27	10	E	27	11	E	945.95370	0			0.4348E-03					
26	10	E	26	11	E	946.04415	-2	.486106E-03	0.7	0.4666E-03	4.01	3.571E-04	-.381E+00	-.379E+00	1.4

(cont.)

J	K_a	Γ	J'	K_a'	Γ'	$\nu^{a)}$	$\delta_\nu{}^{b)}$	$S_\nu^{\rm exp}(294.45)^{c)}$	$\Delta_S{}^{d)}$	$S_\nu^{\rm calc}(294.45)^{e)}$	$\delta_S{}^{f)}$	$\Gamma_0{}^{g)}$	$\Delta_2{}^{g)}$	$\nu_{\rm VC}{}^{g)}$	$R^{h)}$
1			2			3	4	5	6	7	8	9	10	11	12
25	10	E	25	11	E	946.13129	1	.478472E-03	1.5	0.4969E-03	-3.85	4.619E-04			1.0
21	7	E	22	8	E	946.17292	14	.271144E-02	1.0	0.2808E-02	-3.56	1.942E-04		-.676E+00	1.3
24	10	E	24	11	E	946.21506	4	.516799E-03	2.6	0.5249E-03	-1.56	1.778E-04		-.935E+00	1.1
41	4	E	42	5	E	946.26691	22	.547245E-03	1.4	0.5281E-03	3.50	2.167E-04		-.712E+00	1.1
23	10	E	23	11	E	946.29540	-1	.535352E-03	1.2	0.5496E-03	-2.65	4.308E-04	-.173E+00	-.144E+00	1.1
14	8	A_2	15	9	A_1	946.31800	14	.608850E-02	0.5	0.3078E-02	-1.11	3.302E-04	-.203E+00	-.471E+00	1.3
14	8	A_1	15	9	A_2	946.31800	14			0.3078E-02					
22	10	E	22	11	E	946.37244	0	.555074E-03	0.8	0.5700E-03	-2.69	1.154E-04			1.0
54	2	A_2	55	3	A_1	946.37244	5			0.5488E-04					
21	10	E	21	11	E	946.44612	0	.569680E-03	1.0	0.5851E-03	-2.71	2.705E-04		-.583E+00	1.1
20	10	E	20	11	E	946.51645	1	.578573E-03	1.3	0.5937E-03	-2.62	3.701E-04		-.886E+00	1.1
19	10	E	19	11	E	946.58341	0	.578801E-03	1.3	0.5946E-03	-2.72	1.455E-04		-.809E+00	1.2
34	5	A_2	35	6	A_1	946.62931	13	.248699E-02	0.4	0.1238E-02	.41	2.644E-04		.130E+00	1.1
34	5	A_1	35	6	A_2	946.62931	13			0.1238E-02					
18	10	E	18	11	E	946.64702	0	.570780E-03	1.4	0.5863E-03	-2.73	4.415E-04			1.0
17	10	E	17	11	E	946.70729	1	.553068E-03	1.5	0.5676E-03	-2.64	2.416E-04			1.0
16	10	E	16	11	E	946.76422	3	.525554E-03	1.2	0.5370E-03	-2.18	3.811E-04			1.0
15	10	E	15	11	E	946.81773	-2	.485362E-03	2.2	0.4928E-03	-1.54	8.032E-05	-.168E+01		1.5
47	3	E	48	4	E	946.86069	16			0.2107E-03					
14	10	E	14	11	E	946.86791	-5	.424998E-03	2.2	0.4335E-03	-1.99	3.961E-04	-.165E+01		1.8
27	6	E	28	7	E	946.91682	8	.241083E-02	6.4	0.2216E-02	-6.74	7.437E-05			1.0
13	10	E	13	11	E	946.91682	201			0.3570E-03					
12	10	E	12	11	E	946.95833	1			0.2611E-03					
11	10	E	11	11	E	946.99849	1			0.1433E-03					
20	7	E	21	8	E	947.12503	14	.289649E-02	0.4	0.3034E-02	-4.73	3.642E-04	-.417E+00	-.376E+00	1.6
22	13	E	21	14	E	947.18791	-30			0.6888E-05					
13	8	A_2	14	9	A_1	947.24869	15	.639745E-02	0.3	0.3238E-02	-1.24	3.703E-04	-.262E+00	-.268E+00	1.8
13	8	A_1	14	9	A_2	947.24869	15			0.3238E-02					
40	4	E	41	5	E	947.27580	10	.649838E-03	1.9	0.6248E-03	3.85	9.453E-06		-.862E+00	1.1
53	2	A_1	54	3	A_2	947.41608	31			0.6885E-04					
53	2	A_2	54	3	A_1	947.42161	-9			0.6885E-04					
15	12	E	14	13	E	947.61022	60			0.3303E-05					
33	5	A_2	34	6	A_1	947.61907	12	.284958E-02	0.5	0.1419E-02	.43	3.233E-04			1.0
33	5	A_1	34	6	A_2	947.61907	12			0.1419E-02					
26	6	E	27	7	E	947.88659	3	.264599E-02	0.5	0.2458E-02	-2.60	3.313E-04		-.791E+00	1.5
59	1	E	60	2	E	947.93832	12			0.1870E-04					
23	13	E	22	14	E	947.99021	-23			0.7489E-05					
57	9	E	57	10	E	948.02410	0			0.6218E-05					
19	7	E	20	8	E	948.07405	5	.325599E-02	0.7	0.3262E-02	-.18	2.833E-04		-.633E+00	1.3
12	8	A_2	13	9	A_1	948.17616	5	.673929E-02	0.4	0.3396E-02	-.78	2.699E-04		-.613E+00	1.6
12	8	A_1	13	9	A_2	948.17616	5			0.3396E-02					
56	9	E	56	10	E	948.21505	-27			0.7869E-05					
39	4	E	40	5	E	948.28208	10	.800709E-03	1.0	0.7357E-03	8.12	1.072E-04		-.717E+00	1.4
55	9	E	55	10	E	948.40320	8			0.9910E-05					
52	2	A_2	53	3	A_1	948.45690	33			0.8599E-04					
52	2	A_1	53	3	A_2	948.46198	-9			0.8599E-04					
32	5	A_2	33	6	A_1	948.60596	6	.316558E-02	0.6	0.1617E-02	-2.18	1.939E-04		-.726E+00	1.5
32	5	A_1	33	6	A_2	948.60596	6			0.1617E-02					
24	13	E	23	14	E	948.78906	13			0.7942E-05					
25	6	E	26	7	E	948.85351	4	.259568E-02	0.7	0.2714E-02	-4.55	2.629E-04		-.638E+00	1.5
45	3	E	46	4	E	948.90795	13	.312838E-03	2.3	0.3100E-03	.89	2.053E-04			1.0
52	9	E	52	10	E	948.94620	5			0.1923E-04					
58	1	E	59	2	E	948.99460	24			0.2396E-04					
18	7	E	19	8	E	949.02013	5	.344810E-02	0.4	0.3492E-02	-1.26	3.492E-04	-.162E-01	-.433E+00	1.2
11	8	A_2	12	9	A_1	949.10061	7	.715433E-02	0.3	0.3551E-02	.73	3.471E-04			1.0
11	8	A_1	12	9	A_2	949.10061	7			0.3551E-02					
51	9	E	51	10	E	949.12002	-35			0.2376E-04					
38	4	E	39	5	E	949.28554	1	.854971E-03	1.1	0.8621E-03	-.83	2.697E-04			1.0
50	9	E	50	10	E	949.29042	-78			0.2920E-04					
49	9	E	49	10	E	949.45876	12			0.3572E-04					
64	0	E	65	1	E	949.46799	0			0.5843E-05					
51	2	A_1	52	3	A_2	949.49515	36			0.1069E-03					
51	2	A_2	52	3	A_1	949.49982	-4			0.1069E-03					
31	5	A_2	32	6	A_1	949.59007	5	.361911E-02	0.5	0.1835E-02	-1.40	3.264E-04	-.128E+00		1.1
31	5	A_1	32	6	A_2	949.59007	5			0.1835E-02					
48	9	E	48	10	E	949.62269	0			0.4348E-04					
47	9	E	47	10	E	949.78331	-5			0.5266E-04					
24	6	E	25	7	E	949.81752	8	.283043E-02	0.3	0.2981E-02	-5.33	3.350E-04		-.555E+00	1.2
44	3	E	45	4	E	949.92761	12			0.3734E-03					
46	9	E	46	10	E	949.94067	1			0.6346E-04					
17	7	E	18	8	E	949.96317	5	.359402E-02	0.3	0.3721E-02	-3.52	3.541E-04	-.614E-01	-.385E+00	1.3
10	8	A_2	11	9	A_1	950.02190	7	.772797E-02	0.3	0.3705E-02	4.11	2.605E-04		-.500E+00	1.3
10	8	A_1	11	9	A_2	950.02190	7			0.3705E-02					
57	1	E	58	2	E	950.04814	16			0.3058E-04					
45	9	E	45	10	E	950.09455	-2			0.7609E-04					
44	9	E	44	10	E	950.24510	-1			0.9077E-04					
37	4	E	38	5	E	950.28642	10	.102426E-02	1.1	0.1005E-02	1.84	3.142E-04			1.0
26	13	E	25	14	E	950.37433	-26			0.8408E-05					
43	9	E	43	10	E	950.39228	1			0.1077E-03					
50	2	A_2	51	3	A_1	950.53111	69			0.1322E-03					
50	2	A_1	51	3	A_2	950.53561	55			0.1322E-03					
50	2	A_1	51	3	A_2	950.53559	53			0.1322E-03					

(cont.)

J	K_a	Γ	J'	K'_a	Γ'	$\nu^{a)}$	$\delta_\nu{}^{b)}$	$S_\nu^{\rm exp}(294.45)^{c)}$	$\Delta_S{}^{d)}$	$S_\nu^{\rm calc}(294.45)^{e)}$	$\delta_S{}^{f)}$	$\Gamma_0{}^{g)}$	$\Delta_2{}^{g)}$	$\nu_{\rm VC}{}^{g)}$	$R^{h)}$
	1			2		3	4	5	6	7	8	9	10	11	12
42	9	E	42	10	E	950.53559	-47			0.1272E-03					
30	5	A_2	31	6	A_1	950.57136	6	.409472E-02	0.8	0.2072E-02	-1.18	3.506E-04	-.201E+00		1.1
30	5	A_1	31	6	A_2	950.57136	6			0.2072E-02					
41	9	E	41	10	E	950.67648	0			0.1495E-03					
23	6	E	24	7	E	950.77854	8	.337784E-02	0.3	0.3259E-02	3.51	3.616E-04			1.0
40	9	E	40	10	E	950.81353	0			0.1747E-03					
16	7	E	17	8	E	950.90318	8	.372059E-02	0.5	0.3947E-02	-6.46	3.640E-04	-.159E+00	-.280E+00	1.2
19	12	E	18	13	E	950.90318	43			0.1376E-04					
9	8	A_2	10	9	A_1	950.94003	8	.744989E-02	0.5	0.3860E-02	-3.63	2.822E-04	-.142E+00		1.1
9	8	A_1	10	9	A_2	950.94003	8			0.3860E-02					
43	3	E	44	4	E	950.94408	-39			0.4476E-03					
38	9	E	38	10	E	951.07745	-9			0.2348E-03					
56	1	E	57	2	E	951.09906	1			0.3882E-04					
27	13	E	26	14	E	951.16155	-19			0.8438E-05					
37	9	E	37	10	E	951.20440	-10			0.2700E-03					
36	4	E	37	5	E	951.28447	12	.120065E-02	0.9	0.1167E-02	2.81	1.934E-04	-.229E+00	-.108E+00	1.2
36	9	E	36	10	E	951.32802	-8	.300122E-03	3.0	0.3089E-03	-2.92	3.973E-04			1.0
35	9	E	35	10	E	951.44824	-10	.362267E-03	3.8	0.3514E-03	3.01	3.990E-04			1.0
29	5	A_2	30	6	A_1	951.54977	6	.456467E-02	0.4	0.2327E-02	-1.97	3.593E-04			1.0
29	5	A_1	30	6	A_2	951.54977	6			0.2327E-02					
34	9	E	34	10	E	951.56463	-59			0.3974E-03					
33	9	E	33	10	E	951.67866	-8	.453005E-03	1.1	0.4470E-03	1.34	2.637E-04	.135E+00	.450E+00	1.1
22	6	E	23	7	E	951.73660	8	.351601E-02	0.4	0.3546E-02	-.85	3.919E-04			1.0
32	9	E	32	10	E	951.78885	-6	.503019E-03	2.2	0.4997E-03	.65	2.222E-04			1.0
15	7	E	16	8	E	951.84012	9	.395962E-02	1.0	0.4169E-02	-5.29	3.829E-04	.280E+00		1.1
8	8	A_2	9	9	A_1	951.85498	8	.777550E-02	0.4	0.4020E-02	-3.41	2.826E-04	.100E+00		1.1
8	8	A_1	9	9	A_2	951.85498	8			0.4020E-02					
31	9	E	31	10	E	951.89568	-5	.558149E-03	9.8	0.5554E-03	.49	6.668E-04			1.0
28	13	E	27	14	E	951.94511	3			0.8349E-05					
42	3	E	43	4	E	951.95882	5	.539456E-03	2.4	0.5340E-03	1.02	1.767E-04			1.0
30	9	E	30	10	E	951.99914	-6	.620868E-03	1.2	0.6136E-03	1.17	3.421E-04	.636E+00		1.1
29	9	E	29	10	E	952.09922	-9	.679238E-03	1.0	0.6736E-03	.83	2.716E-04	.350E+00		1.2
55	1	E	56	2	E	952.14757	1			0.4910E-04					
28	9	E	28	10	E	952.19602	-6	.730155E-03	1.4	0.7347E-03	-.62	5.376E-04			1.0
35	4	E	36	5	E	952.27972	11	.137531E-02	1.8	0.1348E-02	2.00	3.493E-04			1.0
27	9	E	27	10	E	952.28942	-8	.782615E-03	1.7	0.7959E-03	-1.70	4.479E-04			1.0
26	9	E	26	10	E	952.37950	-7	.848507E-03	1.2	0.8564E-03	-.93	5.279E-04	.510E+00	.299E+00	1.2
25	9	E	25	10	E	952.46604	-26	.897622E-03	4.6	0.9147E-03	-1.90	3.733E-04			1.0
28	5	A_2	29	6	A_1	952.52533	8	.509770E-02	0.5	0.2602E-02	-2.08	3.572E-04			1.0
28	5	A_1	29	6	A_2	952.52533	8			0.2602E-02					
24	9	E	24	10	E	952.54963	-5	.962843E-03	0.8	0.9696E-03	-.70	3.409E-04			1.0
48	2	A_2	49	3	A_1	952.59404	23			0.1996E-03					
48	2	A_1	49	3	A_2	952.59764	1			0.1996E-03					
23	9	E	23	10	E	952.62962	-10	.100798E-02	1.1	0.1019E-02	-1.13	3.101E-04			1.0
61	0	E	62	1	E	952.67191	14			0.1286E-04					
21	6	E	22	7	E	952.69170	9	.386749E-02	0.8	0.3839E-02	.74	3.812E-04			1.0
22	9	E	22	10	E	952.70637	-4	.107869E-02	13.6	0.1063E-02	1.50	1.628E-03			1.0
29	13	E	28	14	E	952.72442	-17			0.8157E-05					
14	7	E	15	8	E	952.77393	6	.423940E-02	0.8	0.4386E-02	-3.45	4.329E-04			1.0
21	9	E	21	10	E	952.77955	-22	.110493E-02	3.3	0.1097E-02	.70	4.414E-04			1.0
20	9	E	20	10	E	952.84974	-4	.111951E-02	2.1	0.1121E-02	-.16	3.707E-04			1.0
19	9	E	19	10	E	952.91642	-4	.112552E-02	1.3	0.1133E-02	-.65	4.071E-04		-.572E+00	1.1
41	3	E	42	4	E	952.97042	4	.660539E-03	2.0	0.6340E-03	4.02	3.474E-04			1.0
18	9	E	18	10	E	952.97976	-3	.112193E-02	1.2	0.1130E-02	-.70	4.176E-04			1.0
17	9	E	17	10	E	953.03976	-3	.109914E-02	1.3	0.1110E-02	-.95	4.347E-04			1.0
16	9	E	16	10	E	953.09641	-4	.106268E-02	1.0	0.1070E-02	-.69	4.364E-04	-.156E+00		1.1
15	9	E	15	10	E	953.14981	4	.987611E-03	0.7	0.1009E-02	-2.12	2.347E-04			1.0
54	1	E	55	2	E	953.19386	36			0.6177E-04					
14	9	E	14	10	E	953.19978	2	.920685E-03	0.9	0.9224E-03	-.19	3.718E-04			1.0
13	9	E	13	10	E	953.24641	0	.802121E-03	1.1	0.8088E-03	-.83	2.526E-04	-.281E+00		1.2
34	4	E	35	5	E	953.27215	7	.150498E-02	1.0	0.1549E-02	-2.94	3.124E-04			1.0
12	9	E	12	10	E	953.28972	-1	.674317E-03	1.5	0.6643E-03	1.49	4.215E-04			1.0
11	9	E	11	10	E	953.32974	2	.476860E-03	3.1	0.4850E-03	-1.70	2.191E-04			1.0
10	9	E	10	10	E	953.36633	-4			0.2658E-03					
27	5	A_2	28	6	A_1	953.49798	7	.562726E-02	0.4	0.2895E-02	-2.88	3.230E-04	.123E+00	-.182E+00	1.1
27	5	A_1	28	6	A_2	953.49798	7			0.2895E-02					
47	2	A_1	48	3	A_2	953.62151	-5			0.2436E-03					
47	2	A_2	48	3	A_1	953.62521	22			0.2436E-03					
20	6	E	21	7	E	953.64379	8	.411232E-02	0.6	0.4136E-02	-.58	3.787E-04	-.937E+00		1.3
13	7	E	14	8	E	953.70466	4	.440285E-02	0.5	0.4595E-02	-4.37	3.342E-04	-.611E+00		1.8
60	0	E	61	1	E	953.73445	-7			0.1658E-04					
40	3	E	41	4	E	953.97928	1	.752331E-03	1.1	0.7491E-03	.43	2.915E-04	-.736E+00	-.144E+00	1.9
23	12	E	22	13	E	954.13714	-11			0.2090E-04					
53	1	E	54	2	E	954.23692	7			0.7740E-04					
58	8	A_1	58	9	A_2	954.24605	0			0.8169E-05					
58	8	A_2	58	9	A_1	954.24605	0			0.8169E-05					
33	4	E	34	5	E	954.26177	2	.170378E-02	1.3	0.1772E-02	-4.00	3.008E-04	-.475E+00		1.4
31	13	E	30	14	E	954.27205	-2			0.7525E-05					
65	1	A_2	66	0	A_1	954.38769	-26			0.1280E-04					
26	5	A_2	27	6	A_1	954.46768	1	.632384E-02	0.3	0.3205E-02	-1.35	3.096E-04	-.296E+00	-.409E+00	1.9
26	5	A_1	27	6	A_2	954.46768	1			0.3205E-02					
19	6	E	20	7	E	954.59284	3	.440154E-02	0.5	0.4435E-02	-.77	3.798E-04	-.310E+00		1.4

(cont.)

J	K_a	Γ	J'	K'_a	Γ'	$\nu^{a)}$	$\delta_\nu{}^{b)}$	$S_\nu^{\rm exp}(294.45)^{c)}$	$\Delta_S{}^{d)}$	$S_\nu^{\rm calc}(294.45)^{e)}$	$\delta_S{}^{f)}$	$\Gamma_0{}^{g)}$	$\Delta_2{}^{g)}$	$\nu_{\rm VC}{}^{g)}$	$R^{h)}$
1			2			3	4	5	6	7	8	9	10	11	12
12	7	E	13	8	E	954.63229	3	.455186E-02	0.2	0.4797E-02	-5.38	3.068E-04	-.223E+00	-.420E+00	2.1
46	2	A_2	47	3	A_1	954.64649	-16			0.2957E-03					
46	2	A_1	47	3	A_2	954.64993	21			0.2957E-03					
59	0	E	60	1	E	954.79463	-4			0.2129E-04					
55	8	A_1	55	9	A_2	954.81755	4			0.1656E-04					
55	8	A_2	55	9	A_1	954.81755	4			0.1656E-04					
24	12	E	23	13	E	954.93656	-4			0.2176E-04					
39	3	E	40	4	E	954.98542	-2	.886789E-03	0.8	0.8809E-03	.66	2.572E-04	-.359E+00	.475E+00	1.4
54	8	A_2	54	9	A_1	955.00095	-26			0.2075E-04					
54	8	A_1	54	9	A_2	955.00095	-26			0.2075E-04					
53	8	A_2	53	9	A_1	955.18221	68			0.2588E-04					
53	8	A_1	53	9	A_2	955.18221	68			0.2588E-04					
32	4	E	33	5	E	955.24864	4			0.2017E-02					
52	1	E	53	2	E	955.27755	-7			0.9649E-04					
52	8	A_2	52	9	A_1	955.35835	-12			0.3213E-04					
52	8	A_1	52	9	A_2	955.35835	-12			0.3213E-04					
25	5	A_2	26	6	A_1	955.43453	2	.694315E-02	0.5	0.3530E-02	-1.69	2.931E-04	.946E-01	-.470E+00	1.2
25	5	A_1	26	6	A_2	955.43453	2			0.3530E-02					
17	11	A_1	16	12	A_2	955.47096	-24			0.2647E-04					
17	11	A_2	16	12	A_1	955.47096	-24			0.2647E-04					
64	1	A_1	65	0	A_2	955.48655	-17			0.1662E-04					
51	8	A_1	51	9	A_2	955.53205	3			0.3970E-04					
51	8	A_2	51	9	A_1	955.53205	3			0.3970E-04					
18	6	E	19	7	E	955.53894	5	.470863E-02	0.7	0.4733E-02	-.52	3.215E-04	.219E+00	-.337E+00	1.2
11	7	E	12	8	E	955.55681	3	.471626E-02	0.5	0.4991E-02	-5.83	3.302E-04	-.130E-01	-.271E+00	1.1
45	2	A_1	46	3	A_2	955.66901	-7			0.3574E-03					
45	2	A_2	46	3	A_1	955.67196	16			0.3574E-03					
50	8	A_1	50	9	A_2	955.70217	-3			0.4880E-04					
50	8	A_2	50	9	A_1	955.70217	-3			0.4880E-04					
25	12	E	24	13	E	955.73228	8			0.2224E-04					
58	0	E	59	1	E	955.85234	13			0.2720E-04					
49	8	A_1	49	9	A_2	955.86888	-12			0.5970E-04					
49	8	A_2	49	9	A_1	955.86888	-12			0.5970E-04					
38	3	E	39	4	E	955.98887	0	.102102E-02	0.6	0.1031E-02	-.97	2.697E-04			1.0
48	8	A_1	48	9	A_2	956.03229	-14			0.7268E-04					
48	8	A_2	48	9	A_1	956.03229	-14			0.7268E-04					
47	8	A_2	47	9	A_1	956.19236	-13			0.8805E-04					
47	8	A_1	47	9	A_2	956.19236	-13			0.8805E-04					
31	4	E	32	5	E	956.23257	-5	.224330E-02	0.5	0.2285E-02	-1.84	3.096E-04	.128E+00	-.183E+00	1.1
18	11	A_2	17	12	A_1	956.29305	-28			0.3253E-04					
18	11	A_1	17	12	A_2	956.29305	-28			0.3253E-04					
51	1	E	52	2	E	956.31581	3			0.1198E-03					
46	8	A_2	46	9	A_1	956.34900	-18			0.1061E-03					
46	8	A_1	46	9	A_2	956.34900	-18			0.1061E-03					
24	5	A_2	25	6	A_1	956.39845	2	.774610E-02	0.2	0.3869E-02	.09	3.129E-04	-.130E+00	-.433E+00	1.5
24	5	A_1	25	6	A_2	956.39845	2			0.3869E-02					
10	7	E	11	8	E	956.47823	7	.503129E-02	0.5	0.5179E-02	-2.94	3.345E-04			1.0
17	6	E	18	7	E	956.48197	2	.521666E-02	0.5	0.5027E-02	3.64	3.345E-04			1.0
45	8	A_2	45	9	A_1	956.50227	-24			0.1273E-03					
45	8	A_1	45	9	A_2	956.50227	-24			0.1273E-03					
26	12	E	25	13	E	956.52371	-35			0.2239E-04					
34	13	E	33	14	E	956.56435	16			0.6196E-05					
63	1	A_2	64	0	A_1	956.58268	-1			0.2149E-04					
44	8	A_1	44	9	A_2	956.65234	-13			0.1519E-03					
44	8	A_2	44	9	A_1	956.65234	-13			0.1519E-03					
44	2	A_2	45	3	A_1	956.68860	-24			0.4299E-03					
43	8	A_2	43	9	A_1	956.79898	-9			0.1804E-03					
43	8	A_1	43	9	A_2	956.79898	-9			0.1804E-03					
57	0	E	58	1	E	956.90748	34			0.3459E-04					
42	8	A_2	42	9	A_1	956.94222	-9			0.2131E-03					
42	8	A_1	42	9	A_2	956.94222	-9			0.2131E-03					
37	3	E	38	4	E	956.98969	13	.123771E-02	1.1	0.1201E-02	3.00	2.452E-04			1.0
41	8	A_2	41	9	A_1	957.08223	4			0.2504E-03					
41	8	A_1	41	9	A_2	957.08223	4			0.2504E-03					
19	11	A_2	18	12	A_1	957.11138	-46			0.3803E-04					
19	11	A_1	18	12	A_2	957.11138	-46			0.3803E-04					
30	4	E	31	5	E	957.21382	1	.242653E-02	0.9	0.2575E-02	-6.11	3.292E-04			1.0
40	8	A_2	40	9	A_1	957.21849	-22	.585964E-03	3.7	0.2928E-03	.07	1.384E-04			1.0
40	8	A_1	40	9	A_2	957.21849	-22			0.2928E-03					
35	13	E	34	14	E	957.32113	72			0.5707E-05					
50	1	E	51	2	E	957.35179	46			0.1479E-03					
50	1	E	51	2	E	957.35167	34			0.1479E-03					
39	8	A_1	39	9	A_2	957.35179	-10			0.3405E-03					
39	8	A_2	39	9	A_1	957.35179	-10			0.3405E-03					
39	8	A_1	39	9	A_2	957.35167	-22			0.3405E-03					
39	8	A_2	39	9	A_1	957.35167	-22			0.3405E-03					
23	5	A_2	24	6	A_1	957.35945	3	.823090E-02	0.4	0.4220E-02	-2.55	3.596E-04			1.0
23	5	A_1	24	6	A_2	957.35945	3			0.4220E-02					
9	7	E	10	8	E	957.39644	5	.511906E-02	0.4	0.5363E-02	-4.77	3.577E-04			1.0
16	6	E	17	7	E	957.42201	5	.527180E-02	0.2	0.5314E-02	-.81	4.118E-04	-.164E+00		1.4
38	8	A_2	38	9	A_1	957.48161	-10	.814126E-03	0.7	0.3940E-03	3.21	1.039E-04		-.848E+00	1.7
38	8	A_1	38	9	A_2	957.48161	-10			0.3940E-03					

(cont.)

J	K_a	Γ	J'	K'_a	Γ'	$\nu^{a)}$	$\delta_\nu{}^{b)}$	$S_\nu^{\rm exp}(294.45)^{c)}$	$\Delta_S{}^{d)}$	$S_\nu^{\rm calc}(294.45)^{e)}$	$\delta_S{}^{f)}$	$\Gamma_0{}^{g)}$	$\Delta_2{}^{g)}$	$\nu_{\rm VC}{}^{g)}$	$R^{h)}$
1			2			3	4	5	6	7	8	9	10	11	12
12	10	E	11	11	E	957.57697	30			0.6427E-05					
37	8	A_2	37	9	A_1	957.60807	-11	.933762E-03	0.8	0.4534E-03	2.88	1.578E-04		-.848E+00	1.7
37	8	A_1	37	9	A_2	957.60807	-11			0.4534E-03					
62	1	A_1	63	0	A_2	957.67600	17			0.2767E-04					
43	2	A_1	44	3	A_2	957.70581	-10			0.5146E-03					
36	8	A_2	36	9	A_1	957.73122	-8	.107831E-02	0.6	0.5190E-03	3.74	2.881E-04	-.153E+00	.504E+00	1.3
36	8	A_1	36	9	A_2	957.73122	-8			0.5190E-03					
35	8	A_2	35	9	A_1	957.85091	-17	.125151E-02	0.9	0.5908E-03	5.58	2.985E-04			1.0
35	8	A_1	35	9	A_2	957.85091	-17			0.5908E-03					
20	11	A_2	19	12	A_1	957.92667	-3			0.4280E-04					
20	11	A_1	19	12	A_2	957.92667	-3			0.4280E-04					
56	0	E	57	1	E	957.95947	2			0.4380E-04					
34	8	A_2	34	9	A_1	957.96740	-11	.143241E-02	0.9	0.6689E-03	6.61	3.122E-04			1.0
34	8	A_1	34	9	A_2	957.96740	-11			0.6689E-03					
36	3	E	37	4	E	957.98749	0	.137142E-02	0.8	0.1391E-02	-1.46	3.014E-04	.116E+00		1.1
33	8	A_2	33	9	A_1	958.08046	-14	.156949E-02	0.4	0.7530E-03	4.05	2.529E-04	-.134E+00	-.908E-01	1.4
33	8	A_1	33	9	A_2	958.08046	-14			0.7530E-03					
28	12	E	27	13	E	958.09593	-50			0.2178E-04					
29	4	E	30	5	E	958.19164	-50			0.2888E-02					
31	8	A_2	31	9	A_1	958.29669	-6	.197633E-02	1.0	0.9379E-03	5.09	2.910E-04	-.400E+00	-.360E+00	1.4
31	8	A_1	31	9	A_2	958.29669	-6			0.9379E-03					
8	7	E	9	8	E	958.31149	3	.524211E-02	1.2	0.5549E-02	-5.85	3.418E-04			1.0
22	5	A_2	23	6	A_1	958.31755	10	.930439E-02	0.7	0.4580E-02	1.55	5.414E-04			1.0
22	5	A_1	23	6	A_2	958.31755	10			0.4580E-02					
15	6	E	16	7	E	958.35897	5	.544280E-02	0.7	0.5592E-02	-2.74	4.099E-04	-.239E+00		1.2
49	1	E	50	2	E	958.38444	18			0.1819E-03					
30	8	A_2	30	9	A_1	958.40004	22			0.1037E-02					
30	8	A_1	30	9	A_2	958.40004	22			0.1037E-02					
29	8	A_2	29	9	A_1	958.49946	-9	.227430E-02	0.4	0.1141E-02	-.31	2.614E-04	-.974E-01	-.397E+00	1.1
29	8	A_1	29	9	A_2	958.49946	-9			0.1141E-02					
28	8	A_2	28	9	A_1	958.59591	-3	.264629E-02	0.2	0.1246E-02	5.81	3.836E-04	-.108E+00	.147E+00	1.1
28	8	A_1	28	9	A_2	958.59591	-3			0.1246E-02					
27	8	A_2	27	9	A_1	958.68900	0	.276591E-02	0.5	0.1353E-02	2.17	3.349E-04			1.0
27	8	A_1	27	9	A_2	958.68900	0			0.1353E-02					
42	2	A_1	43	3	A_2	958.72241	31			0.6131E-03					
21	11	A_1	20	12	A_2	958.73763	-27			0.4674E-04					
21	11	A_2	20	12	A_1	958.73763	-27			0.4674E-04					
26	8	A_2	26	9	A_1	958.77869	-3	.304558E-02	0.7	0.1459E-02	4.20	3.485E-04			1.0
26	8	A_1	26	9	A_2	958.77869	-3			0.1459E-02					
25	8	A_2	25	9	A_1	958.86510	-1	.307905E-02	0.6	0.1562E-02	-1.48	2.948E-04	-.203E+00	-.209E+00	1.1
25	8	A_1	25	9	A_2	958.86510	-1			0.1562E-02					
24	8	A_2	24	9	A_1	958.94822	5	.329521E-02	1.1	0.1661E-02	-.81	3.113E-04			1.0
24	8	A_1	24	9	A_2	958.94822	5			0.1661E-02					
35	3	E	36	4	E	958.98261	-4			0.1605E-02					
55	0	E	56	1	E	959.00918	5			0.5521E-04					
23	8	A_2	23	9	A_1	959.02793	3	.346274E-02	0.4	0.1752E-02	-1.22	4.151E-04			1.0
23	8	A_1	23	9	A_2	959.02793	3			0.1752E-02					
22	8	A_2	22	9	A_1	959.10443	13			0.1834E-02					
22	8	A_1	22	9	A_2	959.10443	13			0.1834E-02					
28	4	E	29	5	E	959.16756	-5	.310509E-02	0.9	0.3222E-02	-3.77	6.279E-04			1.0
21	8	A_2	21	9	A_1	959.17739	2	.371183E-02	1.0	0.1903E-02	-2.54	4.038E-04			1.0
21	8	A_1	21	9	A_2	959.17739	2			0.1903E-02					
7	7	E	8	8	E	959.22347	12	.553062E-02	0.6	0.5744E-02	-3.86	3.458E-04			1.0
20	8	A_2	20	9	A_1	959.24712	0	.389621E-02	0.4	0.1956E-02	-.42	3.940E-04			1.0
20	8	A_1	20	9	A_2	959.24712	0			0.1956E-02					
21	5	A_2	22	6	A_1	959.27261	10	.955103E-02	0.1	0.4946E-02	-3.56	3.721E-04	-.112E+00	-.254E+00	1.8
21	5	A_1	22	6	A_2	959.27261	10			0.4946E-02					
14	6	E	15	7	E	959.29292	11	.583889E-02	0.3	0.5858E-02	-.33	3.837E-04	-.139E+00		1.2
19	8	A_2	19	9	A_1	959.31357	4	.394211E-02	0.3	0.1990E-02	-.98	3.500E-04	-.192E+00	-.437E+00	1.6
19	8	A_1	19	9	A_2	959.31357	4			0.1990E-02					
18	8	A_2	18	9	A_1	959.37666	4	.396426E-02	0.3	0.2002E-02	-1.02	3.566E-04	-.143E+00	-.199E+00	1.4
18	8	A_1	18	9	A_2	959.37666	4			0.2002E-02					
48	1	E	49	2	E	959.41463	7			0.2226E-03					
17	8	A_2	17	9	A_1	959.43643	4	.384351E-02	0.3	0.1988E-02	-3.46	3.104E-04	-.299E+00	-.427E+00	2.1
17	8	A_1	17	9	A_2	959.43643	4			0.1988E-02					
16	8	A_2	16	9	A_1	959.49288	5	.391930E-02	0.4	0.1945E-02	.75	4.074E-04	-.151E+00	.811E-01	1.2
16	8	A_1	16	9	A_2	959.49288	5			0.1945E-02					
22	11	A_1	21	12	A_2	959.54600	58	.375640E-02	0.2	0.4979E-04	-.80	3.481E-04	-.124E+00	-.330E+00	1.5
22	11	A_2	21	12	A_1	959.54600	58			0.4979E-04					
15	8	A_1	15	9	A_2	959.54600	5			0.1868E-02					
15	8	A_2	15	9	A_1	959.54600	5			0.1868E-02					
14	8	A_2	14	9	A_1	959.59581	7	.343924E-02	0.4	0.1754E-02	-2.03	3.543E-04	-.132E+00		1.2
14	8	A_1	14	9	A_2	959.59581	7			0.1754E-02					
13	8	A_2	13	9	A_1	959.64231	9	.310250E-02	0.4	0.1599E-02	-3.10	3.462E-04	-.126E+00		1.1
13	8	A_1	13	9	A_2	959.64231	9			0.1599E-02					
12	8	A_2	12	9	A_1	959.68546	9	.279964E-02	0.5	0.1398E-02	.11	4.150E-04			1.0
12	8	A_1	12	9	A_2	959.68546	9			0.1398E-02					
11	8	A_2	11	9	A_1	959.72528	9	.229305E-02	0.5	0.1146E-02	.07	3.856E-04			1.0
11	8	A_1	11	9	A_2	959.72528	9			0.1146E-02					
41	2	A_1	42	3	A_2	959.73249	56			0.7269E-03					
10	8	A_2	10	9	A_1	959.76176	6	.169809E-02	0.7	0.8351E-03	1.64	3.399E-04	.185E+00		1.2
10	8	A_1	10	9	A_2	959.76176	6			0.8351E-03					

(cont.)

J	K_a	Γ	J'	K'_a	Γ'	$\nu^{a)}$	$\delta_\nu{}^{b)}$	$S_\nu^{\mathrm{exp}}(294.45)^{c)}$	$\Delta_S{}^{d)}$	$S_\nu^{\mathrm{calc}}(294.45)^{e)}$	$\delta_S{}^{f)}$	$\Gamma_0{}^{g)}$	$\Delta_2{}^{g)}$	$\nu_{\mathrm{VC}}{}^{g)}$	$R^{h)}$
	1			2		3	4	5	6	7	8	9	10	11	12
9	8	A_2	9	9	A_1	959.79501	12	.900656E-03	1.2	0.4575E-03	-1.58	2.971E-04	-.421E+00		1.3
9	8	A_1	9	9	A_2	959.79501	12			0.4575E-03					
60	1	A_1	61	0	A_2	959.85382	23			0.4526E-04					
34	3	E	35	4	E	959.97514	12	.179420E-02	0.5	0.1842E-02	-2.65	2.126E-04	-.207E+00		1.2
54	0	E	55	1	E	960.05652	35			0.6926E-04					
27	4	E	28	5	E	960.14031	12	.340065E-02	0.3	0.3578E-02	-5.21	3.361E-04	-.532E-01	-.230E+00	1.1
13	6	E	14	7	E	960.22435	74	.156739E-01	0.2	0.6110E-02	-6.79	4.693E-04	-.684E-01	-.776E+00	1.8
20	5	A_1	21	6	A_2	960.22435	-24			0.5314E-02					
20	5	A_2	21	6	A_1	960.22435	-24			0.5314E-02					
23	11	A_2	22	12	A_1	960.34919	-7			0.5196E-04					
23	11	A_1	22	12	A_2	960.34919	-7			0.5196E-04					
31	12	E	30	13	E	960.42607	-34			0.1919E-04					
47	1	E	48	2	E	960.44229	8			0.2712E-03					
40	2	A_2	41	3	A_1	960.74165	78	.169691E-02	2.9	0.8578E-03	-1.10	1.236E-03			1.0
40	2	A_1	41	3	A_2	960.74165	-56			0.8578E-03					
57	7	E	57	8	E	960.91685	26			0.1621E-04					
59	1	A_2	60	0	A_1	960.93829	10			0.5750E-04					
33	3	E	34	4	E	960.96471	12	.203788E-02	0.9	0.2103E-02	-3.21	3.464E-04			1.0
53	0	E	54	1	E	961.10058	1			0.8651E-04					
26	4	E	27	5	E	961.10998	9	.372761E-02	0.6	0.3952E-02	-6.03	3.073E-04			1.0
12	6	E	13	7	E	961.15143	11	.629317E-02	0.3	0.6347E-02	-.86	3.802E-04	-.283E+00	-.192E+00	1.6
19	5	A_2	20	6	A_1	961.17380	12	.107998E-01	0.2	0.5680E-02	-5.20	3.584E-04	-.189E+00	-.395E+00	1.6
19	5	A_1	20	6	A_2	961.17380	12			0.5680E-02					
46	1	E	47	2	E	961.46723	2			0.3287E-03					
53	7	E	53	8	E	961.65560	-17			0.4042E-04					
39	2	A_1	40	3	A_2	961.74772	65			0.1007E-02					
39	2	A_2	40	3	A_1	961.74772	-48			0.1007E-02					
52	7	E	52	8	E	961.83193	-19			0.5019E-04					
25	11	A_1	24	12	A_2	961.94550	-29			0.5376E-04					
25	11	A_2	24	12	A_1	961.94550	-29			0.5376E-04					
32	3	E	33	4	E	961.95146	10	.240786E-02	1.0	0.2390E-02	.74	1.638E-04		-.578E+00	1.1
33	12	E	32	13	E	961.96031	-19			0.1682E-04					
51	7	E	51	8	E	962.00507	-3			0.6201E-04					
58	1	A_1	59	0	A_2	962.01975	-16			0.7273E-04					
11	6	E	12	7	E	962.07620	29	.102308E-01	0.2	0.6568E-02	-6.67	3.969E-04		-.543E+00	1.5
25	4	E	26	5	E	962.07620	-48			0.4344E-02					
18	5	A_2	19	6	A_1	962.11976	-1	.114465E-01	0.2	0.6042E-02	-5.57	3.494E-04	-.994E-01	-.469E+00	1.6
18	5	A_1	19	6	A_2	962.11976	-1			0.6042E-02					
52	0	E	53	1	E	962.14221	-11			0.1075E-03					
50	7	E	50	8	E	962.17446	-26			0.7625E-04					
49	7	E	49	8	E	962.34080	-17			0.9330E-04					
45	1	E	46	2	E	962.48954	0			0.3967E-03					
48	7	E	48	8	E	962.50362	-23			0.1136E-03					
18	10	E	17	11	E	962.56685	12			0.8267E-04					
47	7	E	47	8	E	962.66316	-22			0.1377E-03					
34	12	E	33	13	E	962.72153	-19			0.1555E-04					
26	11	A_2	25	12	A_1	962.73835	-11			0.5350E-04					
26	11	A_1	25	12	A_2	962.73835	-11			0.5350E-04					
38	2	A_2	39	3	A_1	962.75084	32	.237684E-02	1.6	0.1177E-02	.96	8.727E-04			1.0
38	2	A_1	39	3	A_2	962.75084	-63		1.6	0.1177E-02		8.727E-04			1.0
31	3	E	32	4	E	962.93526	-4	.276751E-02	0.5	0.2702E-02	2.36	4.473E-04			1.0
45	7	E	45	8	E	962.97218	-18			0.1991E-03					
10	6	E	11	7	E	962.99739	1	.672918E-02	0.3	0.6775E-02	-.68	3.562E-04	-.272E+00		1.3
24	4	E	25	5	E	963.04052	-3	.455156E-02	0.4	0.4751E-02	-4.38	3.549E-04	-.585E-01	-.308E+00	1.2
17	5	A_2	18	6	A_1	963.06283	-1	.120594E-01	0.2	0.6395E-02	-6.06	3.564E-04	-.227E+00	-.296E+00	1.9
17	5	A_1	18	6	A_2	963.06283	-1			0.6395E-02					
57	1	A_2	58	0	A_1	963.09866	-8			0.9160E-04					
44	7	E	44	8	E	963.12164	-18			0.2377E-03					
51	0	E	52	1	E	963.18138	-2			0.1331E-03					
43	7	E	43	8	E	963.26754	-39			0.2824E-03					
19	10	E	18	11	E	963.38613	27			0.9317E-04					
42	7	E	42	8	E	963.41051	-18			0.3337E-03					
35	12	E	34	13	E	963.47881	-24			0.1426E-04					
44	1	E	45	2	E	963.50911	-9	.500197E-03	2.3	0.4763E-03	4.77	9.870E-05	-.114E+01		1.4
27	11	A_1	26	12	A_2	963.52763	26			0.5259E-04					
27	11	A_2	26	12	A_1	963.52763	26			0.5259E-04					
41	7	E	41	8	E	963.54990	-20	.415532E-03	1.0	0.3923E-03	5.58	1.269E-04	-.665E+00		1.6
40	7	E	40	8	E	963.68602	-15	.502050E-03	3.7	0.4589E-03	8.59	4.170E-04			1.0
37	2	A_1	38	3	A_2	963.75156	34		1.4	0.1369E-02		8.504E-04			1.0
37	2	A_2	38	3	A_1	963.75156	-45	.278024E-02	1.4	0.1369E-02	1.55	8.504E-04			1.0
39	7	E	39	8	E	963.81873	-17	.573909E-03	1.8	0.5340E-03	6.95	3.458E-04			1.0
30	3	E	31	4	E	963.91592	-48	.954697E-02	0.4	0.3040E-02	-4.84	4.333E-04			1.0
9	6	E	10	7	E	963.91592	21			0.6969E-02					
38	7	E	38	8	E	963.94814	-14	.664237E-03	2.0	0.6182E-03	6.93	2.930E-04			1.0
16	5	A_2	17	6	A_1	964.00255	-32	.179932E-01	0.5	0.6735E-02	-3.59	6.563E-04	-.489E+00		1.2
16	5	A_1	17	6	A_2	964.00255	-32			0.6735E-02					
37	7	E	37	8	E	964.07416	-17	.734585E-03	1.5	0.7119E-03	3.09	2.681E-04			1.0
56	1	A_1	57	0	A_2	964.17463	-3			0.1148E-03					
36	7	E	36	8	E	964.19691	-12	.835393E-03	2.2	0.8154E-03	2.40	2.072E-04	-.586E+00		1.1
20	10	E	19	11	E	964.20096	-39			0.1019E-03					
50	0	E	51	1	E	964.21772	-9			0.1639E-03					
36	12	E	35	13	E	964.23166	-80			0.1298E-04					

(cont.)

J	K_a	Γ	J'	K_a'	Γ'	$\nu^{a)}$	$\delta_\nu{}^{b)}$	$S_\nu^{\rm exp}(294.45)^{c)}$	$\Delta_S{}^{d)}$	$S_\nu^{\rm calc}(294.45)^{e)}$	$\delta_S{}^{f)}$	$\Gamma_0{}^{g)}$	$\Delta_2{}^{g)}$	$\nu_{\rm VC}{}^{g)}$	$R^{h)}$
	1			2		3	4	5	6	7	8	9	10	11	12
35	7	E	35	8	E	964.31627	-14	.988106E-03	1.6	0.9289E-03	6.00	2.217E-04	-.107E+01	.453E+00	1.2
34	7	E	34	8	E	964.43226	-18			0.1052E-02					
43	1	E	44	2	E	964.52610	-7	.593079E-03	2.3	0.5693E-03	4.01	2.653E-04			1.0
33	7	E	33	8	E	964.54501	-14	.112458E-02	0.9	0.1186E-02	-5.44	2.231E-04			1.0
32	7	E	32	8	E	964.65440	-12	.127729E-02	1.5	0.1329E-02	-4.01	4.216E-04			1.0
36	2	A_2	37	3	A_1	964.74943	28	.325111E-02	1.1	0.1584E-02	2.58	6.304E-04			1.0
36	2	A_1	37	3	A_2	964.74943	-36			0.1584E-02					
31	7	E	31	8	E	964.76049	-8	.142779E-02	2.1	0.1480E-02	-3.66	3.044E-04			1.0
8	6	E	9	7	E	964.83089	0	.727141E-02	0.3	0.7155E-02	1.60	3.195E-04			1.0
30	7	E	30	8	E	964.86320	-8	.159542E-02	1.8	0.1639E-02	-2.74	3.496E-04			1.0
29	3	E	30	4	E	964.89467	1	.326233E-02	0.6	0.3403E-02	-4.31	3.325E-04			1.0
15	5	A_2	16	6	A_1	964.93984	-1	.136387E-01	0.3	0.7058E-02	-3.50	3.586E-04	.113E+00	-.261E+00	1.2
15	5	A_1	16	6	A_2	964.93984	-1			0.7058E-02					
22	4	E	23	5	E	964.95948	-1	.547645E-02	3.3	0.5594E-02	-2.15	2.836E-04			1.0
29	7	E	29	8	E	964.96248	-19			0.1805E-02					
37	12	E	36	13	E	964.98216	23			0.1173E-04					
21	10	E	20	11	E	965.01306	-14			0.1088E-03					
28	7	E	28	8	E	965.05862	-12	.203231E-02	0.6	0.1975E-02	2.84	3.442E-04	-.363E+00		1.2
29	11	A_2	28	12	A_1	965.09404	16			0.4914E-04					
29	11	A_1	28	12	A_2	965.09404	16			0.4914E-04					
27	7	E	27	8	E	965.15136	-12	.215744E-02	0.5	0.2147E-02	.47	3.013E-04	-.512E-01	-.319E+00	1.1
26	7	E	26	8	E	965.24111	22			0.2320E-02					
49	0	E	50	1	E	965.25152	-3			0.2010E-03					
25	7	E	25	8	E	965.32689	-10	.261108E-02	0.5	0.2490E-02	4.64	4.025E-04			1.0
24	7	E	24	8	E	965.40969	-8	.267921E-02	0.5	0.2654E-02	.95	3.684E-04			1.0
23	7	E	23	8	E	965.48916	-6	.287196E-02	0.4	0.2808E-02	2.22	3.271E-04	-.331E+00	-.293E+00	1.1
42	1	E	43	2	E	965.54040	-5	.729237E-03	0.7	0.6771E-03	7.16	4.878E-04	.209E-01	.188E+01	1.3
22	7	E	22	8	E	965.56511	-25			0.2949E-02					
21	7	E	21	8	E	965.63812	-6	.308182E-02	0.4	0.3072E-02	.32	3.960E-04			1.0
62	2	E	63	1	E	965.66086	-13			0.1159E-04					
20	7	E	20	8	E	965.70762	-6	.314687E-02	0.6	0.3173E-02	-.82	3.685E-04			1.0
38	12	E	37	13	E	965.72704	-42			0.1053E-04					
7	6	E	8	7	E	965.74318	28			0.7344E-02					
19	7	E	19	8	E	965.77382	-5	.320877E-02	1.0	0.3247E-02	-1.18	3.049E-04	-.848E+00	-.240E+00	1.6
22	10	E	21	11	E	965.82173	35			0.1138E-03					
18	7	E	18	8	E	965.83668	-6	.326575E-02	0.9	0.3289E-02	-.70	4.052E-04			1.0
28	3	E	29	4	E	965.87018	12		0.3	0.3790E-02		3.992E-04			1.0
14	5	A_2	15	6	A_1	965.87378	0	.143469E-01	0.3	0.7360E-02	-2.61	3.992E-04			1.0
14	5	A_1	15	6	A_2	965.87378	0			0.7360E-02					
17	7	E	17	8	E	965.89630	0	.323186E-02	0.5	0.3294E-02	-1.91	3.380E-04	-.269E+00	-.307E+00	1.3
21	4	E	22	5	E	965.91450	-3	.575385E-02	0.3	0.6024E-02	-4.69	3.891E-04			1.0
16	7	E	16	8	E	965.95252	-3	.319140E-02	0.5	0.3257E-02	-2.05	3.496E-04	-.188E+00	-.292E+00	1.1
15	7	E	15	8	E	966.00547	-2	.311095E-02	0.4	0.3172E-02	-1.98	3.267E-04	-.640E-01	-.367E+00	1.2
14	7	E	14	8	E	966.05511	0	.299287E-02	0.7	0.3036E-02	-1.43	3.804E-04	-.226E+00		1.1
13	7	E	13	8	E	966.10138	-4	.283793E-02	0.6	0.2840E-02	-.09	4.044E-04	-.336E+00		1.4
12	7	E	12	8	E	966.14441	-1	.256272E-02	0.5	0.2581E-02	-.71	4.006E-04	-.289E+00	-.126E+00	1.3
11	7	E	11	8	E	966.18409	-3	.223527E-02	0.5	0.2250E-02	-.66	3.767E-04			1.0
10	7	E	10	8	E	966.22049	-1	.181799E-02	0.4	0.1840E-02	-1.21	3.539E-04			1.0
9	7	E	9	8	E	966.25359	2	.132760E-02	1.3	0.1340E-02	-.91	3.531E-04			1.0
48	0	E	49	1	E	966.28305	45			0.2452E-03					
8	7	E	8	8	E	966.28308	-25			0.7340E-03					
54	1	A_1	55	0	A_2	966.31768	-5			0.1781E-03					
15	9	E	14	10	E	966.42383	-8			0.1155E-03					
62	6	E	62	7	E	966.45673	59			0.6809E-05					
39	12	E	38	13	E	966.46890	-12			0.9385E-05					
41	1	E	42	2	E	966.55199	-2	.827592E-03	1.8	0.8014E-03	3.16	2.612E-04			1.0
23	10	E	22	11	E	966.62540	-50			0.1169E-03					
31	11	A_1	30	12	A_2	966.64532	14			0.4415E-04					
31	11	A_2	30	12	A_1	966.64532	14			0.4415E-04					
6	6	E	7	7	E	966.65174	2	.783896E-02	0.5	0.7551E-02	3.67	2.690E-04	-.317E+00		1.3
61	6	E	61	7	E	966.66287	48			0.8825E-05					
61	2	E	62	1	E	966.71829	2			0.1489E-04					
34	2	A_2	35	3	A_1	966.73683	18	.424930E-02	0.6	0.2089E-02	1.67	4.767E-04	-.204E+00		1.1
34	2	A_1	35	3	A_2	966.73683	-23			0.2089E-02					
13	5	A_2	14	6	A_1	966.80468	5	.149146E-01	0.2	0.7639E-02	-2.44	5.242E-04	-.713E-01	-.145E+00	1.1
13	5	A_1	14	6	A_2	966.80468	5			0.7639E-02					
27	3	E	28	4	E	966.84258	0	.401844E-02	0.3	0.4199E-02	-4.49	3.359E-04	-.208E+00		1.1
60	6	E	60	7	E	966.86636	111			0.1138E-04					
20	4	E	21	5	E	966.86657	-2	.616392E-02	0.3	0.6453E-02	-4.68	4.022E-04	-.186E+00		1.1
40	12	E	39	13	E	967.20648	-12			0.8309E-05					
16	9	E	15	10	E	967.25422	-11			0.1421E-03					
47	0	E	48	1	E	967.31096	0			0.2978E-03					
53	1	A_2	54	0	A_1	967.38486	0			0.2204E-03					
32	11	A_1	31	12	A_2	967.41453	-56			0.4130E-04					
32	11	A_2	31	12	A_1	967.41453	-56			0.4130E-04					
24	10	E	23	11	E	967.42647	-25			0.1183E-03					
40	1	E	41	2	E	967.56082	-4	.101765E-02	0.7	0.9440E-03	7.24	3.128E-04			1.0
33	2	A_2	34	3	A_1	967.72632	-20	.479032E-02	1.0	0.2382E-02	.57	4.019E-04			1.0
33	2	A_1	34	3	A_2	967.72632	12			0.2382E-02					
12	5	A_2	13	6	A_1	967.73239	0	.152526E-01	0.3	0.7893E-02	-3.49	3.804E-04			1.0
12	5	A_1	13	6	A_2	967.73239	0			0.7893E-02					
26	3	E	27	4	E	967.81223	1	.440656E-02	1.2	0.4628E-02	-5.03	4.531E-04			1.0

(cont.)

J	K_a	Γ	J'	K'_a	Γ'	$\nu^{a)}$	$\delta_\nu{}^{b)}$	$S_\nu^{\rm exp}(294.45)^{c)}$	$\Delta_S{}^{d)}$	$S_\nu^{\rm calc}(294.45)^{e)}$	$\delta_S{}^{f)}$	$\Gamma_0{}^{g)}$	$\Delta_2{}^{g)}$	$\nu_{\rm VC}{}^{g)}$	$R^{h)}$
	1			2		3	4	5	6	7	8	9	10	11	12
19	4	E	20	5	E	967.81570	3	.657779E-02	1.2	0.6876E-02	-4.53	4.531E-04			1.0
54	6	E	54	7	E	968.01102	-38			0.4750E-04					
17	9	E	16	10	E	968.08115	-4			0.1664E-03					
33	11	A_1	32	12	A_2	968.18095	-20			0.3831E-04					
33	11	A_2	32	12	A_1	968.18095	-20			0.3831E-04					
53	6	E	53	7	E	968.19049	-13			0.5927E-04					
25	10	E	24	11	E	968.22368	-16			0.1181E-03					
46	0	E	47	1	E	968.33658	-3			0.3601E-03					
52	6	E	52	7	E	968.36630	-18			0.7360E-04					
52	1	A_1	53	0	A_2	968.44901	-1			0.2714E-03					
51	6	E	51	7	E	968.53889	-8			0.9096E-04					
39	1	E	40	2	E	968.56692	-6	.115899E-02	1.0	0.1107E-02	4.53	3.036E-04			1.0
11	5	A_2	12	6	A_1	968.65706	1	.153658E-01	0.3	0.8119E-02	-5.67	3.218E-04	-.308E+00	-.395E+00	1.9
11	5	A_1	12	6	A_2	968.65706	1			0.8119E-02					
32	2	A_2	33	3	A_1	968.71306	13	.532616E-02	0.6	0.2701E-02	-1.44	4.329E-04	-.185E+00		1.1
32	2	A_1	33	3	A_2	968.71306	-12			0.2701E-02					
18	4	E	19	5	E	968.76175	0	.693495E-02	0.3	0.7288E-02	-5.09	4.290E-04			1.0
25	3	E	26	4	E	968.77897	1	.495151E-02	0.6	0.5075E-02	-2.50	4.112E-04	-.216E+00		1.2
49	6	E	49	7	E	968.87351	-37			0.1369E-03					
18	9	E	17	10	E	968.90459	13			0.1878E-03					
34	11	A_2	33	12	A_1	968.94316	-18			0.3526E-04					
34	11	A_1	33	12	A_2	968.94316	-18			0.3526E-04					
26	10	E	25	11	E	969.01712	-12			0.1164E-03					
48	6	E	48	7	E	969.03649	19			0.1668E-03					
47	6	E	47	7	E	969.19522	-15			0.2021E-03					
46	6	E	46	7	E	969.35096	-12			0.2438E-03					
45	0	E	46	1	E	969.35952	-3	.440667E-03	1.8	0.4332E-03	1.69	3.074E-04			1.0
43	12	E	42	13	E	969.39499	-29			0.5552E-05					
45	6	E	45	7	E	969.50324	-22			0.2926E-03					
51	1	A_2	52	0	A_1	969.51026	5			0.3328E-03					
38	1	E	39	2	E	969.57024	-11			0.1291E-02					
10	5	A_2	11	6	A_1	969.57861	2	.156182E-01	1.1	0.8318E-02	-6.51	3.307E-04	.322E+00		1.1
10	5	A_1	11	6	A_2	969.57861	2			0.8318E-02					
44	6	E	44	7	E	969.65231	-17			0.3494E-03					
31	2	A_2	32	3	A_1	969.69692	-10	.597870E-02	0.5	0.3049E-02	-1.98	4.178E-04			1.0
31	2	A_1	32	3	A_2	969.69692	7			0.3049E-02					
17	4	E	18	5	E	969.70483	1	.719955E-02	0.5	0.7684E-02	-6.73	4.336E-04			1.0
24	3	E	25	4	E	969.74277	-1	.529546E-02	0.8	0.5536E-02	-4.54	3.536E-04	-.383E+00		1.1
43	6	E	43	7	E	969.79802	-15			0.4151E-03					
27	10	E	26	11	E	969.80660	-30			0.1135E-03					
58	2	E	59	1	E	969.87608	-52			0.3075E-04					
42	6	E	42	7	E	969.94037	-14	.517273E-03	0.6	0.4908E-03	5.12	6.979E-04	-.117E+00	.530E+01	1.6
41	6	E	41	7	E	970.07941	-11	.629242E-03	1.9	0.5773E-03	8.26	1.052E-04	.143E+01		1.3
40	6	E	40	7	E	970.21507	-12	.712966E-03	2.1	0.6756E-03	5.25	3.113E-04			1.0
12	8	A_2	11	9	A_1	970.31023	21			0.1126E-03					
12	8	A_1	11	9	A_2	970.31023	21			0.1126E-03					
39	6	E	39	7	E	970.34741	-11	.759234E-03	1.0	0.7865E-03	-3.59	5.314E-04			1.0
44	0	E	45	1	E	970.37978	1	.543390E-03	2.5	0.5187E-03	4.54	2.282E-04			1.0
36	11	A_1	35	12	A_2	970.45596	-12			0.2922E-04					
36	11	A_2	35	12	A_1	970.45596	-12			0.2922E-04					
38	6	E	38	7	E	970.47653	1	.996476E-03	1.8	0.9109E-03	8.58	6.244E-04			1.0
9	5	A_2	10	6	A_1	970.49700	0	.159359E-01	0.8	0.8491E-02	-6.57	3.454E-04	-.678E+00	-.184E+00	1.3
9	5	A_1	10	6	A_2	970.49700	0			0.8491E-02					
20	9	E	19	10	E	970.54014	-5			0.2201E-03					
64	3	E	65	2	E	970.55062	-29			0.6660E-05					
37	1	E	38	2	E	970.57097	0	.152216E-02	1.5	0.1498E-02	1.58	5.645E-04			1.0
37	6	E	37	7	E	970.60209	-11	.101333E-02	2.4	0.1050E-02	-3.57	1.789E-04			1.0
16	4	E	17	5	E	970.64487	1	.769207E-02	0.3	0.8059E-02	-4.77	4.141E-04			1.0
30	2	A_2	31	3	A_1	970.67797	5	.678892E-02	0.4	0.3423E-02	-.83	4.144E-04			1.0
30	2	A_1	31	3	A_2	970.67797	-7			0.3423E-02					
23	3	E	24	4	E	970.70369	1	.571097E-02	1.0	0.6007E-02	-5.18	6.268E-04			1.0
36	6	E	36	7	E	970.72445	-9	.124364E-02	2.3	0.1203E-02	3.28	3.238E-04			1.0
35	6	E	35	7	E	970.84343	-13	.145280E-02	0.7	0.1371E-02	5.62	2.729E-04	-.476E+00	-.123E+00	1.4
57	2	E	58	1	E	970.92485	4			0.3881E-04					
34	6	E	34	7	E	970.95913	-12	.164694E-02	1.0	0.1555E-02	5.60	3.744E-04	-.651E+00	-.231E+00	1.4
33	6	E	33	7	E	971.07154	-8	.185301E-02	0.8	0.1753E-02	5.39	2.697E-04	-.133E+00		1.1
13	8	A_1	12	9	A_2	971.15152	5			0.1645E-03					
13	8	A_2	12	9	A_1	971.15152	5			0.1645E-03					
32	6	E	32	7	E	971.18054	-13	.203974E-02	0.4	0.1966E-02	3.62	3.134E-04			1.0
37	11	A_2	36	12	A_1	971.20653	-5			0.2632E-04					
37	11	A_1	36	12	A_2	971.20653	-5			0.2632E-04					
31	6	E	31	7	E	971.28630	-9	.228139E-02	0.9	0.2192E-02	3.91	3.748E-04			1.0
21	9	E	20	10	E	971.35230	-32			0.2306E-03					
29	10	E	28	11	E	971.37490	-5			0.1045E-03					
30	6	E	30	7	E	971.38879	-1	.259494E-02	0.8	0.2430E-02	6.34	3.560E-04			1.0
43	0	E	44	1	E	971.39741	14	.655777E-03	4.7	0.6182E-03	5.73	4.495E-04			1.0
8	5	A_2	9	6	A_1	971.41227	1	.172931E-01	0.2	0.8645E-02	.02	3.314E-04			1.0
8	5	A_1	9	6	A_2	971.41227	1			0.8645E-02					
29	6	E	29	7	E	971.48784	-5	.276450E-02	0.7	0.2679E-02	3.10	3.639E-04	-.235E+00		1.3
36	1	E	37	2	E	971.56884	2	.172399E-02	4.3	0.1730E-02	-.36	3.192E-04			1.0
28	6	E	28	7	E	971.58205	-162	.120085E-01	0.6	0.2935E-02	5.55	7.715E-04			1.0
15	4	E	16	5	E	971.58205	18			0.8408E-02					

(cont.)

J	K_a	Γ	J'	K_a'	Γ'	$\nu^{a)}$	$\delta_\nu{}^{b)}$	$S_\nu^{\mathrm{exp}}(294.45)^{c)}$	$\Delta_S{}^{d)}$	$S_\nu^{\mathrm{calc}}(294.45)^{e)}$	$\delta_S{}^{f)}$	$\Gamma_0{}^{g)}$	$\Delta_2{}^{g)}$	$\nu_{\mathrm{VC}}{}^{g)}$	$R^{h)}$
1			2			3	4	5	6	7	8	9	10	11	12
49	1	A_2	50	0	A_1	971.62362	1			0.4935E-03					
29	2	A_2	30	3	A_1	971.65615	-6			0.3823E-02					
29	2	A_1	30	3	A_2	971.65615	2			0.3823E-02					
22	3	E	23	4	E	971.66167	3			0.6483E-02					
27	6	E	27	7	E	971.67610	-3	.309270E-02	0.8	0.3196E-02	-3.35	2.801E-04	-.542E+00	-.308E+00	1.4
26	6	E	26	7	E	971.76523	-5	.351819E-02	0.5	0.3459E-02	1.68	3.794E-04			1.0
25	6	E	25	7	E	971.85113	2	.383499E-02	2.1	0.3719E-02	3.02	2.545E-04			1.0
56	2	E	57	1	E	971.97026	-45			0.4877E-04					
14	8	A_2	13	9	A_1	971.98966	25			0.2167E-03					
14	8	A_1	13	9	A_2	971.98966	25			0.2167E-03					
23	6	E	23	7	E	972.01298	13			0.4214E-02					
22	6	E	22	7	E	972.08871	-5	.440342E-02	0.4	0.4437E-02	-.77	3.815E-04	-.152E+00		1.1
22	9	E	21	10	E	972.16131	-10	.479228E-02	0.6	0.2375E-03	-1.73	3.518E-04			1.0
21	6	E	21	7	E	972.16131	-5			0.4638E-02					
20	6	E	20	7	E	972.23062	-3	.473936E-02	0.4	0.4808E-02	-1.46	3.947E-04			1.0
47	12	E	46	13	E	972.25695	19			0.2982E-05					
19	6	E	19	7	E	972.29664	0	.500490E-02	0.5	0.4943E-02	1.24	4.145E-04	.159E+00	-.235E+00	1.2
7	5	A_2	8	6	A_1	972.32439	2	.176202E-01	0.2	0.8787E-02	.26	3.105E-04			1.0
7	5	A_1	8	6	A_2	972.32439	2			0.8787E-02					
18	6	E	18	7	E	972.35924	-8	.509280E-02	0.4	0.5034E-02	1.15	4.341E-04	-.111E+00		1.1
42	0	E	43	1	E	972.41210	8	.789096E-03	3.6	0.7332E-03	7.09	7.422E-05			1.0
17	6	E	17	7	E	972.41868	-2	.506992E-02	0.7	0.5076E-02	-.12	3.840E-04			1.0
16	6	E	16	7	E	972.47475	-2	.505345E-02	0.6	0.5061E-02	-.15	3.961E-04			1.0
14	4	E	15	5	E	972.51583	1	.818999E-02	0.5	0.8725E-02	-6.53	4.102E-04			1.0
15	6	E	15	7	E	972.52756	1	.490045E-02	1.0	0.4982E-02	-1.67	3.765E-04			1.0
35	1	E	36	2	E	972.56388	-2	.199622E-02	1.3	0.1988E-02	.40	2.783E-04			1.0
14	6	E	14	7	E	972.57703	1	.481236E-02	0.6	0.4833E-02	-.43	3.801E-04			1.0
21	3	E	22	4	E	972.61671	6	.677066E-02	0.4	0.6959E-02	-2.79	3.858E-04			1.0
13	6	E	13	7	E	972.62314	-5	.469578E-02	0.6	0.4606E-02	1.91	3.931E-04			1.0
28	2	A_2	29	3	A_1	972.63152	3	.828270E-02	0.4	0.4248E-02	-2.58	4.180E-04			1.0
28	2	A_1	29	3	A_2	972.63152	-2			0.4248E-02					
12	6	E	12	7	E	972.66607	1	.434287E-02	0.5	0.4294E-02	1.13	4.451E-04			1.0
62	3	E	63	2	E	972.67575	11			0.1109E-04					
48	1	A_1	49	0	A_2	972.67575	-5			0.5968E-03					
11	6	E	11	7	E	972.70565	2	.379421E-02	0.5	0.3889E-02	-2.49	2.676E-04	-.336E+00	-.396E+00	2.0
10	6	E	10	7	E	972.74191	1	.332321E-02	0.8	0.3382E-02	-1.75	3.190E-04	-.493E+00	-.265E+00	1.6
9	6	E	9	7	E	972.77479	-8	.278765E-02	0.6	0.2760E-02	.99	4.234E-04	-.458E+00	-.500E+00	1.7
8	6	E	8	7	E	972.80457	3	.198958E-02	0.4	0.2009E-02	-.96	2.997E-04	-.454E+00	-.320E+00	2.4
15	8	A_2	14	9	A_1	972.82405	22	.545066E-03	5.1	0.2667E-03	2.16	1.255E-04			1.0
15	8	A_1	14	9	A_2	972.82405	22			0.2667E-03					
7	6	E	7	7	E	972.83078	-14			0.1102E-02					
63	5	A_2	63	6	A_1	972.84648	77			0.7212E-05					
63	5	A_1	63	6	A_2	972.84648	77			0.7212E-05					
31	10	E	30	11	E	972.92745	-42			0.9286E-04					
23	9	E	22	10	E	972.96639	-14			0.2409E-03					
55	2	E	56	1	E	973.01427	-2			0.6101E-04					
6	5	A_2	7	6	A_1	973.23332	1	.178009E-01	0.2	0.8934E-02	-.38	2.723E-04	-.106E+00	-.157E+00	1.3
6	5	A_1	7	6	A_2	973.23332	1			0.8934E-02					
41	0	E	42	1	E	973.42397	-6			0.8654E-03					
13	4	E	14	5	E	973.44672	2	.904072E-02	0.3	0.9006E-02	.38	3.460E-04	-.225E+00	-.294E+00	1.4
60	5	A_1	60	6	A_2	973.46414	106			0.1571E-04					
60	5	A_2	60	6	A_1	973.46414	106			0.1571E-04					
34	1	E	35	2	E	973.55623	4	.228571E-02	2.4	0.2273E-02	.54	3.035E-04			1.0
20	3	E	21	4	E	973.56849	-20			0.7430E-02					
27	2	A_2	28	3	A_1	973.60400	1	.928031E-02	0.2	0.4696E-02	-1.21	3.606E-04	-.455E-01	-.257E+00	1.2
27	2	A_1	28	3	A_2	973.60400	3			0.4696E-02					
16	8	A_1	15	9	A_2	973.65478	7			0.3125E-03					
16	8	A_2	15	9	A_1	973.65478	7			0.3125E-03					
61	3	E	62	2	E	973.73462	7			0.1423E-04					
24	9	E	23	10	E	973.76805	7			0.2411E-03					
57	5	A_1	57	6	A_2	974.05031	31			0.3280E-04					
57	5	A_2	57	6	A_1	974.05031	31			0.3280E-04					
54	2	E	55	1	E	974.05541	-11			0.7598E-04					
5	5	A_2	6	6	A_1	974.13908	3	.182698E-01	0.2	0.9113E-02	.23	2.353E-04	-.236E+00	-.372E+00	2.3
5	5	A_1	6	6	A_2	974.13908	3			0.9113E-02					
41	11	A_1	40	12	A_2	974.16885	-30			0.1621E-04					
41	11	A_2	40	12	A_1	974.16885	-30			0.1621E-04					
9	7	E	8	8	E	974.22547	3			0.4397E-04					
56	5	A_1	56	6	A_2	974.23869	-20			0.4153E-04					
56	5	A_2	56	6	A_1	974.23869	-20			0.4153E-04					
12	4	E	13	5	E	974.37453	3	.920490E-02	0.2	0.9249E-02	-.48	3.334E-04	-.109E+00	-.511E+00	1.8
55	5	A_2	55	6	A_1	974.42423	-18			0.5233E-04					
55	5	A_1	55	6	A_2	974.42423	-18			0.5233E-04					
40	0	E	41	1	E	974.43335	7			0.1016E-02					
33	10	E	32	11	E	974.46542	-10			0.7983E-04					
17	8	A_2	16	9	A_1	974.48187	-16			0.3529E-03					
17	8	A_1	16	9	A_2	974.48187	-16			0.3529E-03					
19	3	E	20	4	E	974.51774	-1			0.7890E-02					
33	1	E	34	2	E	974.54566	-2	.254317E-02	0.6	0.2586E-02	-1.70	2.597E-04			1.0
25	9	E	24	10	E	974.56529	-45			0.2383E-03					
26	2	A_2	27	3	A_1	974.57356	-1	.103265E-01	0.4	0.5164E-02	-.01	2.903E-04		-.608E+00	1.4
26	2	A_1	27	3	A_2	974.57356	-1			0.5164E-02					

(cont.)

J	K_a	Γ	J'	K'_a	Γ'	$\nu^{a)}$	$\delta_\nu{}^{b)}$	$S_\nu^{\rm exp}(294.45)^{c)}$	$\Delta_S{}^{d)}$	$S_\nu^{\rm calc}(294.45)^{e)}$	$\delta_S{}^{f)}$	$\Gamma_0{}^{g)}$	$\Delta_2{}^{g)}$	$\nu_{\rm VC}{}^{g)}$	$R^{h)}$
	1			2		3	4	5	6	7	8	9	10	11	12
54	5	A_2	54	6	A_1	974.60640	-17			0.6563E-04					
54	5	A_1	54	6	A_2	974.60640	-17			0.6563E-04					
46	1	A_1	47	0	A_2	974.77106	0			0.8610E-03					
53	5	A_2	53	6	A_1	974.78515	-21			0.8192E-04					
53	5	A_1	53	6	A_2	974.78515	-21			0.8192E-04					
42	11	A_1	41	12	A_2	974.89914	-70			0.1414E-04					
42	11	A_2	41	12	A_1	974.89914	-70			0.1414E-04					
52	5	A_2	52	6	A_1	974.96062	-18			0.1018E-03					
52	5	A_1	52	6	A_2	974.96062	-18			0.1018E-03					
10	7	E	9	8	E	975.07705	-62			0.1143E-03					
51	5	A_1	51	6	A_2	975.13274	-14			0.1258E-03					
51	5	A_2	51	6	A_1	975.13274	-14			0.1258E-03					
34	10	E	33	11	E	975.22845	-13			0.7319E-04					
11	4	E	12	5	E	975.29924	3	.933201E-02	0.1	0.9450E-02	-1.27	4.469E-04			1.0
18	8	A_2	17	9	A_1	975.30573	-6	.790158E-03	1.7	0.3871E-03	2.02	3.643E-04			1.0
18	8	A_1	17	9	A_2	975.30573	-6			0.3871E-03					
26	9	E	25	10	E	975.35956	-23			0.2329E-03					
39	0	E	40	1	E	975.43977	0			0.1188E-02					
18	3	E	19	4	E	975.46382	0	.805854E-02	0.6	0.8331E-02	-3.38	4.861E-04			1.0
32	1	E	33	2	E	975.53233	-3	.288277E-02	1.2	0.2927E-02	-1.54	2.565E-04			1.0
25	2	A_2	26	3	A_1	975.54026	1	.111225E-01	0.3	0.5648E-02	-1.56	3.894E-04			1.0
25	2	A_1	26	3	A_2	975.54026	-1			0.5648E-02					
48	5	A_2	48	6	A_1	975.62839	-61			0.2308E-03					
48	5	A_1	48	6	A_2	975.62839	-61			0.2308E-03					
47	5	A_2	47	6	A_1	975.78756	-11			0.2798E-03					
47	5	A_1	47	6	A_2	975.78756	-11			0.2798E-03					
45	1	A_2	46	0	A_1	975.81414	4	.106146E-02	1.0	0.1027E-02	3.24	2.782E-04			1.0
59	3	E	60	2	E	975.84516	-22			0.2308E-04					
11	7	E	10	8	E	975.92630	-15			0.1992E-03					
46	5	A_2	46	6	A_1	975.94294	-7			0.3376E-03					
46	5	A_1	46	6	A_2	975.94294	-7			0.3376E-03					
35	10	E	34	11	E	975.98759	-19			0.6661E-04					
45	5	A_2	45	6	A_1	976.09490	-10	.899215E-03	0.8	0.4053E-03	9.86	2.428E-04			1.0
45	5	A_1	45	6	A_2	976.09490	-10			0.4053E-03					
19	8	A_2	18	9	A_1	976.12595	0			0.4146E-03					
19	8	A_1	18	9	A_2	976.12595	0			0.4146E-03					
52	2	E	53	1	E	976.13041	-48			0.1163E-03					
27	9	E	26	10	E	976.15000	-12			0.2253E-03					
10	4	E	11	5	E	976.22093	12	.946999E-02	0.3	0.9609E-02	-1.47	3.744E-04			1.0
44	5	A_2	44	6	A_1	976.24366	0	.106425E-02	1.6	0.4841E-03	9.02	2.943E-04			1.0
44	5	A_1	44	6	A_2	976.24366	0			0.4841E-03					
44	11	A_2	43	12	A_1	976.34861	-56			0.1057E-04					
44	11	A_1	43	12	A_2	976.34861	-56			0.1057E-04					
43	5	A_2	43	6	A_1	976.38901	3	.126971E-02	1.1	0.5755E-03	9.36	2.868E-04	.264E+00		1.2
43	5	A_1	43	6	A_2	976.38901	3			0.5755E-03					
17	3	E	18	4	E	976.40699	11	.862848E-02	0.5	0.8747E-02	-1.38	3.815E-04	.186E+00	-.301E+00	1.3
38	0	E	39	1	E	976.44358	11	.139580E-02	1.2	0.1382E-02	1.00	2.827E-04			1.0
24	2	A_2	25	3	A_1	976.50416	11	.122374E-01	0.2	0.6144E-02	-.41	3.829E-04			1.0
24	2	A_1	25	3	A_2	976.50416	14			0.6144E-02					
31	1	E	32	2	E	976.51630	9	.323190E-02	0.9	0.3296E-02	-1.99	2.956E-04			1.0
42	5	A_2	42	6	A_1	976.53094	-3	.147344E-02	0.5	0.6806E-03	7.62	3.155E-04			1.0
42	5	A_1	42	6	A_2	976.53094	-3			0.6806E-03					
41	5	A_2	41	6	A_1	976.66960	-2	.173604E-02	0.4	0.8008E-03	7.74	2.481E-04			1.0
41	5	A_1	41	6	A_2	976.66960	-2			0.8008E-03					
36	10	E	35	11	E	976.74297	-12			0.6021E-04					
12	7	E	11	8	E	976.77182	6			0.2903E-03					
40	5	A_2	40	6	A_1	976.80493	-2	.196219E-02	1.2	0.9376E-03	4.44	2.391E-04			1.0
40	5	A_1	40	6	A_2	976.80493	-2			0.9376E-03					
44	1	A_1	45	0	A_2	976.85420	12	.127788E-02	0.9	0.1219E-02	4.58	2.561E-04			1.0
28	9	E	27	10	E	976.93696	25	.256434E-02	0.5	0.2159E-03	6.41	3.228E-04			1.0
39	5	A_2	39	6	A_1	976.93696	1			0.1092E-02					
39	5	A_1	39	6	A_2	976.93696	1			0.1092E-02					
20	8	A_2	19	9	A_1	976.94255	3	.869373E-03	1.9	0.4353E-03	-.15	2.738E-04			1.0
20	8	A_1	19	9	A_2	976.94255	3			0.4353E-03					
38	5	A_2	38	6	A_1	977.06562	0	.279786E-02	0.6	0.1265E-02	9.54	2.885E-04			1.0
38	5	A_1	38	6	A_2	977.06562	0			0.1265E-02					
9	4	E	10	5	E	977.13940	12	.948782E-02	0.6	0.9727E-02	-2.52	3.526E-04			1.0
51	2	E	52	1	E	977.16499	0			0.1428E-03					
37	5	A_2	37	6	A_1	977.19059	-38			0.1459E-02					
37	5	A_1	37	6	A_2	977.19059	-38			0.1459E-02					
36	5	A_2	36	6	A_1	977.31303	3	.345130E-02	0.8	0.1673E-02	3.07	2.642E-04	-.147E+00		1.1
36	5	A_1	36	6	A_2	977.31303	3			0.1673E-02					
16	3	E	17	4	E	977.34706	13	.856891E-02	0.2	0.9133E-02	-6.58	4.193E-04	-.170E+00		1.2
35	5	A_2	35	6	A_1	977.43172	1	.413521E-02	0.5	0.1908E-02	7.72	3.164E-04			1.0
35	5	A_1	35	6	A_2	977.43172	1			0.1908E-02					
37	0	E	38	1	E	977.44449	9	.160341E-02	1.2	0.1599E-02	.25	2.679E-04			1.0
23	2	A_2	24	3	A_1	977.46500	12	.129620E-01	0.2	0.6647E-02	-2.56	3.334E-04		-.490E+00	1.6
23	2	A_1	24	3	A_2	977.46500	9			0.6647E-02					
30	1	E	31	2	E	977.49729	6			0.3692E-02					
34	5	A_2	34	6	A_1	977.54713	3	.450660E-02	0.4	0.2165E-02	3.94	2.891E-04	-.269E+00		1.4
34	5	A_1	34	6	A_2	977.54713	3			0.2165E-02					
64	4	A_2	65	3	A_1	977.63675	-4			0.5900E-05					

(cont.)

J	K_a	Γ	J'	K'_a	Γ'	$\nu^{a)}$	$\delta_\nu{}^{b)}$	$S_\nu^{\rm exp}(294.45)^{c)}$	$\Delta_S{}^{d)}$	$S_\nu^{\rm calc}(294.45)^{e)}$	$\delta_S{}^{f)}$	$\Gamma_0{}^{g)}$	$\Delta_2{}^{g)}$	$\nu_{\rm VC}{}^{g)}$	$R^{h)}$
1			2			3	4	5	6	7	8	9	10	11	12
64	4	A_1	65	3	A_2	977.63675	-4			0.5900E-05					
33	5	A_2	33	6	A_1	977.65922	4	.501635E-02	0.4	0.2443E-02	2.62	2.937E-04	-.153E+00		1.2
33	5	A_1	33	6	A_2	977.65922	4			0.2443E-02					
29	9	E	28	10	E	977.71903	-52			0.2050E-03					
21	8	A_2	20	9	A_1	977.75553	6	.925762E-03	2.1	0.4493E-03	2.94	4.330E-04			1.0
21	8	A_1	20	9	A_2	977.75553	6			0.4493E-03					
32	5	A_2	32	6	A_1	977.76797	3	.561198E-02	0.3	0.2741E-02	2.31	2.978E-04			1.0
32	5	A_1	32	6	A_2	977.76797	3			0.2741E-02					
46	11	A_2	45	12	A_1	977.78273	40			0.7716E-05					
46	11	A_1	45	12	A_2	977.78273	40			0.7716E-05					
31	5	A_1	31	6	A_2	977.87347	8			0.3059E-02					
31	5	A_2	31	6	A_1	977.87347	8			0.3059E-02					
43	1	A_2	44	0	A_1	977.89103	7	.147286E-02	3.0	0.1441E-02	2.17	1.245E-04			1.0
30	5	A_2	30	6	A_1	977.97558	5	.668427E-02	0.4	0.3395E-02	-1.57	2.506E-04	-.314E+00	-.371E+00	1.6
30	5	A_1	30	6	A_2	977.97558	5			0.3395E-02					
8	4	E	9	5	E	978.05475	13			0.9807E-02					
29	5	A_2	29	6	A_1	978.07443	7	.732654E-02	0.2	0.3745E-02	-2.24	2.632E-04	-.244E+00	-.371E+00	2.5
29	5	A_1	29	6	A_2	978.07443	7			0.3745E-02					
28	5	A_2	28	6	A_1	978.16990	2	.844286E-02	0.5	0.4108E-02	2.68	3.135E-04	-.324E+00	-.454E+00	1.7
28	5	A_1	28	6	A_2	978.16990	2			0.4108E-02					
50	2	E	51	1	E	978.19650	-18			0.1747E-03					
38	10	E	37	11	E	978.24205	3			0.4822E-04					
27	5	A_1	27	6	A_2	978.26218	8			0.4479E-02					
27	5	A_2	27	6	A_1	978.26218	8			0.4479E-02					
15	3	E	16	4	E	978.28406	12	.876015E-02	0.3	0.9481E-02	-8.22	3.758E-04	-.211E+00	-.228E+00	1.5
26	5	A_2	26	6	A_1	978.35107	6	.957201E-02	0.2	0.4854E-02	-1.43	3.377E-04	-.163E+00		1.2
26	5	A_1	26	6	A_2	978.35107	6			0.4854E-02					
22	2	A_2	23	3	A_1	978.42291	8	.139213E-01	0.3	0.7152E-02	-2.74	3.874E-04			1.0
22	2	A_1	23	3	A_2	978.42291	11			0.7152E-02					
25	5	A_2	25	6	A_1	978.43668	6	.102274E-01	0.3	0.5228E-02	-2.23	3.579E-04			1.0
25	5	A_1	25	6	A_2	978.43668	6			0.5228E-02					
36	0	E	37	1	E	978.44271	18	.187224E-02	1.7	0.1842E-02	1.63	3.024E-04			1.0
29	1	E	30	2	E	978.47553	13	.406575E-02	0.7	0.4114E-02	-1.19	3.701E-04	-.197E+00		1.1
30	9	E	29	10	E	978.49846	-16			0.1930E-03					
24	5	A_2	24	6	A_1	978.51896	4	.107713E-01	0.3	0.5594E-02	-3.86	3.551E-04			1.0
24	5	A_1	24	6	A_2	978.51896	4			0.5594E-02					
23	5	A_1	23	6	A_2	978.59818	26			0.5945E-02					
23	5	A_2	23	6	A_1	978.59818	26			0.5945E-02					
22	5	A_2	22	6	A_1	978.67369	6	.121561E-01	0.4	0.6275E-02	-3.25	3.610E-04			1.0
22	5	A_1	22	6	A_2	978.67369	6			0.6275E-02					
21	5	A_2	21	6	A_1	978.74610	7	.129450E-01	0.2	0.6576E-02	-1.60	3.872E-04			1.0
21	5	A_1	21	6	A_2	978.74610	7			0.6576E-02					
20	5	A_2	20	6	A_1	978.81504	-10	.132956E-01	0.3	0.6839E-02	-2.88	3.542E-04	-.190E+00	-.376E+00	1.4
20	5	A_1	20	6	A_2	978.81504	-10			0.6839E-02					
19	5	A_2	19	6	A_1	978.88085	-9	.138658E-01	0.2	0.7056E-02	-1.78	4.023E-04			1.0
19	5	A_1	19	6	A_2	978.88085	-9			0.7056E-02					
42	1	A_1	43	0	A_2	978.92462	-13	.171091E-02	1.7	0.1695E-02	.94	3.424E-04			1.0
18	5	A_2	18	6	A_1	978.94336	-10	.139040E-01	0.2	0.7218E-02	-3.83	3.851E-04			1.0
18	5	A_1	18	6	A_2	978.94336	-10			0.7218E-02					
7	4	E	8	5	E	978.96675	-6	.971160E-02	2.0	0.9857E-02	-1.50	1.568E-04			1.0
39	10	E	38	11	E	978.98560	0			0.4274E-04					
56	3	E	57	2	E	978.99388	-8			0.4614E-04					
17	5	A_2	17	6	A_1	979.00257	-10	.142709E-01	0.4	0.7317E-02	-2.54	3.346E-04	-.247E+00	-.388E+00	1.5
17	5	A_1	17	6	A_2	979.00257	-10			0.7317E-02					
16	5	A_2	16	6	A_1	979.05851	-9	.142257E-01	0.3	0.7342E-02	-3.22	3.398E-04	-.195E+00	-.488E+00	1.5
16	5	A_1	16	6	A_2	979.05851	-9			0.7342E-02					
15	5	A_1	15	6	A_2	979.11114	-8		0.2	0.7286E-02		3.545E-04	-.179E+00	-.455E+00	2.0
15	5	A_2	15	6	A_1	979.11114	-8	.140400E-01	0.2	0.7286E-02	-3.79	3.545E-04	-.179E+00	-.455E+00	2.0
14	5	A_2	14	6	A_1	979.16048	-8	.137861E-01	0.2	0.7140E-02	-3.58	3.358E-04	-.198E+00	-.492E+00	1.9
14	5	A_1	14	6	A_2	979.16048	-8			0.7140E-02					
13	5	A_2	13	6	A_1	979.20652	-8	.133023E-01	0.3	0.6895E-02	-3.67	3.638E-04			1.0
13	5	A_1	13	6	A_2	979.20652	-8			0.6895E-02					
14	3	E	15	4	E	979.21785	-5	.893603E-02	0.4	0.9785E-02	-9.50	3.997E-04			1.0
12	5	A_2	12	6	A_1	979.24928	-8	.132608E-01	1.5	0.6544E-02	1.31	2.579E-04			1.0
12	5	A_1	12	6	A_2	979.24928	-8			0.6544E-02					
31	9	E	30	10	E	979.27383	-7			0.1802E-03					
11	5	A_1	11	6	A_2	979.28872	-10			0.6077E-02					
11	5	A_2	11	6	A_1	979.28872	-10			0.6077E-02					
10	5	A_2	10	6	A_1	979.32491	-8	.105820E-01	0.6	0.5486E-02	-3.68	2.969E-04	-.206E+00	-.412E+00	1.3
10	5	A_1	10	6	A_2	979.32491	-8			0.5486E-02					
9	5	A_2	9	6	A_1	979.35780	-7	.938350E-02	0.4	0.4757E-02	-1.40	3.556E-04	-.333E+00		1.4
9	5	A_1	9	6	A_2	979.35780	-7			0.4757E-02					
23	8	A_2	22	9	A_1	979.37023	-22	.943639E-03	7.9	0.4581E-03	2.91	1.642E-04			1.0
23	8	A_1	22	9	A_2	979.37023	-22			0.4581E-03					
21	2	A_2	22	3	A_1	979.37773	-4	.148891E-01	0.4	0.7651E-02	-2.78	3.921E-04			1.0
21	2	A_1	22	3	A_2	979.37773	-8			0.7651E-02					
8	5	A_2	8	6	A_1	979.38737	-9	.751945E-02	0.8	0.3878E-02	-3.13	3.570E-04			1.0
8	5	A_1	8	6	A_2	979.38737	-9			0.3878E-02					
7	5	A_2	7	6	A_1	979.41371	-5	.557986E-02	0.5	0.2822E-02	-1.16	3.841E-04	-.122E+00	.263E+00	1.1
7	5	A_1	7	6	A_2	979.41371	-5			0.2822E-02					
6	5	A_2	6	6	A_1	979.43712	34	.544342E-02	0.7	0.1553E-02	4.17	5.834E-04			1.0
6	5	A_1	6	6	A_2	979.43712	34			0.1553E-02					

(cont.)

J	K_a	Γ	J'	K_a'	Γ'	$\nu^{a)}$	$\delta_\nu{}^{b)}$	$S_\nu^{\rm exp}(294.45)^{c)}$	$\Delta_S{}^{d)}$	$S_\nu^{\rm calc}(294.45)^{e)}$	$\delta_S{}^{f)}$	$\Gamma_0{}^{g)}$	$\Delta_2{}^{g)}$	$\nu_{\rm VC}{}^{g)}$	$R^{h)}$
	1			2		3	4	5	6	7	8	9	10	11	12
28	1	E	29	2	E	979.45056	-15			0.4560E-02					
40	10	E	39	11	E	979.72507	-17			0.3765E-04					
6	4	E	7	5	E	979.87576	-7	.973011E-02	0.3	0.9892E-02	-1.67	3.552E-04			1.0
8	6	E	7	7	E	979.89325	69			0.7458E-04					
49	11	A_1	48	12	A_2	979.90144	2			0.4617E-05					
49	11	A_2	48	12	A_1	979.90144	2			0.4617E-05					
41	1	A_2	42	0	A_1	979.95529	-13	.197318E-02	0.6	0.1984E-02	-.55	2.384E-04			1.0
55	3	E	56	2	E	980.03848	-23			0.5760E-04					
32	9	E	31	10	E	980.04513	-25			0.1670E-03					
16	7	E	15	8	E	980.11793	-8			0.6214E-03					
13	3	E	14	4	E	980.14873	-7	.928481E-02	0.4	0.1004E-01	-8.13	3.762E-04	-.143E+00	-.182E+00	1.1
24	8	A_2	23	9	A_1	980.17228	-18			0.4540E-03					
24	8	A_1	23	9	A_2	980.17228	-18			0.4540E-03					
48	2	E	49	1	E	980.25284	9			0.2577E-03					
20	2	A_2	21	3	A_1	980.32972	-10	.159856E-01	0.2	0.8140E-02	-1.84	3.849E-04	-.587E-01	-.137E+00	1.1
20	2	A_1	21	3	A_2	980.32972	-6			0.8140E-02					
27	1	E	28	2	E	980.42307	-8	.492410E-02	0.4	0.5028E-02	-2.11	3.510E-04			1.0
34	0	E	35	1	E	980.43021	-16	.244456E-02	0.8	0.2406E-02	1.58	3.460E-04			1.0
50	11	A_2	49	12	A_1	980.59945	-9			0.3849E-05					
50	11	A_1	49	12	A_2	980.59945	-9			0.3849E-05					
57	4	E	57	5	E	980.70504	-8			0.4282E-04					
9	6	E	8	7	E	980.74789	-63			0.1923E-03					
5	4	E	6	5	E	980.78160	-8	.976019E-02	0.2	0.9936E-02	-1.80	2.409E-04		-.502E+00	1.6
33	9	E	32	10	E	980.81291	-14			0.1536E-03					
61	4	A_1	62	3	A_2	980.82101	-64			0.1257E-04					
61	4	A_2	62	3	A_1	980.82101	-64			0.1257E-04					
17	7	E	16	8	E	980.94579	4	.692061E-03	1.4	0.6822E-03	1.43	5.437E-04	.122E+01		1.2
25	8	A_2	24	9	A_1	980.97062	-17	.925743E-03	1.6	0.4449E-03	3.88	3.897E-04			1.0
25	8	A_1	24	9	A_2	980.97062	-17			0.4449E-03					
40	1	A_1	41	0	A_2	980.98284	-12	.226783E-02	0.9	0.2312E-02	-1.93	2.428E-04			1.0
12	3	E	13	4	E	981.07655	-8	.968161E-02	0.2	0.1024E-01	-5.78	3.997E-04	-.672E-01		1.1
42	10	E	41	11	E	981.19238	-23			0.2869E-04					
54	4	E	54	5	E	981.26058	-6			0.8576E-04					
47	2	E	48	1	E	981.27777	66			0.3108E-03					
19	2	A_2	20	3	A_1	981.27874	-8	.169031E-01	0.3	0.8610E-02	-1.87	4.546E-04			1.0
19	2	A_1	20	3	A_2	981.27874	-12			0.8610E-02					
26	1	E	27	2	E	981.39261	-9	.531832E-02	0.4	0.5513E-02	-3.67	3.475E-04	-.169E+00	-.194E+00	1.2
33	0	E	34	1	E	981.41998	-7	.269252E-02	0.4	0.2729E-02	-1.35	2.607E-04	-.332E+00	-.349E+00	1.9
34	9	E	33	10	E	981.57651	-37			0.1403E-03					
10	6	E	9	7	E	981.60085	-20			0.3331E-03					
52	4	E	52	5	E	981.61402	-17			0.1330E-03					
4	4	E	5	5	E	981.68426	-7	.984339E-02	0.5	0.1003E-01	-1.94	2.583E-04	-.404E+00		1.4
26	8	A_1	25	9	A_2	981.76523	-20			0.4316E-03					
26	8	A_2	25	9	A_1	981.76523	-20			0.4316E-03					
18	7	E	17	8	E	981.76979	-14			0.7316E-03					
51	4	E	51	5	E	981.78557	-36			0.1645E-03					
60	4	A_2	61	3	A_1	981.87839	-20			0.1603E-04					
60	4	A_1	61	3	A_2	981.87839	-20			0.1603E-04					
43	10	E	42	11	E	981.92005	-26			0.2482E-04					
50	4	E	50	5	E	981.95421	-11			0.2024E-03					
11	3	E	12	4	E	982.00129	-9	.982341E-02	0.2	0.1039E-01	-5.72	3.949E-04			1.0
39	1	A_2	40	0	A_1	982.00726	-9	.261983E-02	0.9	0.2680E-02	-2.30	3.733E-04			1.0
49	4	E	49	5	E	982.11939	3			0.2479E-03					
18	2	A_2	19	3	A_1	982.22480	-11	.177704E-01	0.3	0.9053E-02	-1.89	3.952E-04			1.0
18	2	A_1	19	3	A_2	982.22480	-7			0.9053E-02					
48	4	E	48	5	E	982.28083	-23			0.3021E-03					
46	2	E	47	1	E	982.29886	-12			0.3731E-03					
35	9	E	34	10	E	982.33662	-24			0.1273E-03					
25	1	E	26	2	E	982.35927	-9	.577952E-02	0.3	0.6012E-02	-4.03	3.637E-04	-.138E+00		1.1
32	0	E	33	1	E	982.40680	-11	.305691E-02	0.4	0.3079E-02	-.73	2.933E-04	-.177E+00	-.255E+00	1.5
47	4	E	47	5	E	982.43927	-14			0.3664E-03					
11	6	E	10	7	E	982.45006	-8	.523541E-03	1.8	0.4837E-03	7.61	1.119E-04			1.0
27	8	A_2	26	9	A_1	982.55614	-21	.863546E-03	1.4	0.4147E-03	3.95	2.854E-04	-.630E+00		1.2
27	8	A_1	26	9	A_2	982.55614	-21			0.4147E-03					
19	7	E	18	8	E	982.59045	-9	.789247E-03	2.9	0.7693E-03	2.53	2.049E-04			1.0
46	4	E	46	5	E	982.59418	-25	.486082E-03	2.9	0.4422E-03	9.03	2.049E-04			1.0
44	10	E	43	11	E	982.64378	-22			0.2135E-04					
53	11	A_2	52	12	A_1	982.66887	-9			0.2159E-05					
53	11	A_1	52	12	A_2	982.66887	-9			0.2159E-05					
45	4	E	45	5	E	982.74598	-13	.571755E-03	3.5	0.5311E-03	7.12	2.349E-04			1.0
44	4	E	44	5	E	982.89432	-13			0.6346E-03					
10	3	E	11	4	E	982.92295	-6	.101130E-01	0.7	0.1047E-01	-3.53	4.043E-04	.294E+00	.274E+00	1.1
38	1	A_1	39	0	A_2	983.02848	-11	.303643E-02	0.4	0.3093E-02	-1.85	3.342E-04			1.0
43	4	E	43	5	E	983.03935	-12	.737329E-03	1.3	0.7546E-03	-2.34	3.042E-04			1.0
36	9	E	35	10	E	983.09282	-16			0.1147E-03					
52	3	E	53	2	E	983.15861	16			0.1091E-03					
17	2	A_2	18	3	A_1	983.16785	-7	.189192E-01	0.2	0.9462E-02	-.03	3.851E-04	-.254E+00	-.261E+00	2.3
17	2	A_1	18	3	A_2	983.16785	-10			0.9462E-02					
42	4	E	42	5	E	983.18094	-22			0.8927E-03					
12	6	E	11	7	E	983.29561	-16	.663525E-03	1.1	0.6345E-03	4.38	5.790E-04			1.0
41	4	E	41	5	E	983.31927	-25			0.1051E-02					
24	1	E	25	2	E	983.32301	-10	.619312E-02	0.4	0.6520E-02	-5.28	3.826E-04			1.0

(cont.)

J	K_a	Γ	J'	K'_a	Γ'	$\nu^{a)}$	$\delta_\nu{}^{b)}$	$S_\nu^{\rm exp}(294.45)^{c)}$	$\Delta_S{}^{d)}$	$S_\nu^{\rm calc}(294.45)^{e)}$	$\delta_S{}^{f)}$	$\Gamma_0{}^{g)}$	$\Delta_2{}^{g)}$	$\nu_{\rm VC}{}^{g)}$	$R^{h)}$
1			2			3	4	5	6	7	8	9	10	11	12
28	8	A_2	27	9	A_1	983.34329	-26	.823946E-03	1.7	0.3950E-03	4.12	6.939E-04	-.624E-01	.502E+01	1.1
28	8	A_1	27	9	A_2	983.34329	-26			0.3950E-03					
45	10	E	44	11	E	983.36337	-28			0.1827E-04					
31	0	E	32	1	E	983.39081	-10	.353594E-02	0.6	0.3457E-02	2.25	3.423E-04			1.0
20	7	E	19	8	E	983.40722	-34	.774525E-03	1.8	0.7953E-03	-2.68	8.404E-04			1.0
40	4	E	40	5	E	983.45486	31			0.1231E-02					
39	4	E	39	5	E	983.58624	-2	.151386E-02	0.9	0.1434E-02	5.26	2.205E-04	-.662E-01		1.0
38	4	E	38	5	E	983.71469	3	.173929E-02	0.5	0.1663E-02	4.41	3.374E-04	-.879E-01	-.823E-01	1.0
9	3	E	10	4	E	983.84149	-5	.129171E-01	0.4	0.1050E-01	3.90	5.534E-04	-.107E+01	-.302E+00	2.9
36	4	E	36	5	E	983.96149	0	.236746E-02	0.5	0.2200E-02	7.08	4.967E-04			1.0
58	4	A_1	59	3	A_2	983.98551	13			0.2573E-04					
58	4	A_2	59	3	A_1	983.98551	14			0.2573E-04					
37	1	A_2	38	0	A_1	984.04668	3	.350621E-02	0.7	0.3551E-02	-1.29	2.939E-04	-.831E+00	.164E+00	1.4
35	4	E	35	5	E	984.08000	6	.273988E-02	0.6	0.2511E-02	7.80	5.971E-04			1.0
46	10	E	45	11	E	984.08000	73			0.1554E-04					
16	2	A_2	17	3	A_1	984.10803	4	.192823E-01	0.4	0.9830E-02	-1.96	3.814E-04	-.589E+00	-.207E+00	1.6
16	2	A_1	17	3	A_2	984.10803	7			0.9830E-02					
29	8	A_2	28	9	A_1	984.12692	-9	.768753E-03	2.1	0.3730E-03	2.95	3.081E-04			1.0
29	8	A_1	28	9	A_2	984.12692	-9			0.3730E-03					
13	6	E	12	7	E	984.13790	-2	.731352E-03	1.7	0.7787E-03	-6.48	4.188E-04			1.0
34	4	E	34	5	E	984.19508	0	.294301E-02	0.5	0.2850E-02	3.16	3.857E-04			1.0
21	7	E	20	8	E	984.22099	2	.858269E-03	1.1	0.8101E-03	5.61	4.987E-04			1.0
23	1	E	24	2	E	984.28399	6	.671427E-02	0.3	0.7031E-02	-4.72	3.439E-04	.479E-01	-.343E+00	1.1
33	4	E	33	5	E	984.30696	6	.331256E-02	0.5	0.3218E-02	2.85	2.569E-04	-.466E-01	-.326E+00	1.1
44	2	E	45	1	E	984.33523	1			0.5302E-03					
30	0	E	31	1	E	984.37211	5	.385149E-02	0.5	0.3860E-02	-.21	3.247E-04			1.0
32	4	E	32	5	E	984.41548	6	.360048E-02	1.3	0.3614E-02	-.37	1.911E-04			1.0
31	4	E	31	5	E	984.52066	3	.397447E-02	0.6	0.4036E-02	-1.54	2.428E-04	.109E+00	-.257E+00	1.1
38	9	E	37	10	E	984.59336	-19			0.9133E-04					
30	4	E	30	5	E	984.62223	-31			0.4482E-02					
29	4	E	29	5	E	984.72118	4	.492311E-02	0.6	0.4949E-02	-.53	3.027E-04		-.191E+00	1.0
8	3	E	9	4	E	984.75702	9	.101922E-01	0.2	0.1046E-01	-2.67	3.807E-04	-.190E+00		1.4
28	4	E	28	5	E	984.81647	3	.561014E-02	0.3	0.5434E-02	3.14	3.447E-04			1.0
27	4	E	27	5	E	984.90841	-4	.681858E-02	0.5	0.5931E-02	2.76	4.428E-04	-.535E+00	-.510E+00	2.0
30	8	A_2	29	9	A_1	984.90841	170			0.3495E-03					
30	8	A_1	29	9	A_2	984.90841	170			0.3495E-03					
14	6	E	13	7	E	984.97669	10	.905969E-03	1.3	0.9117E-03	-.63	3.191E-04			1.0
26	4	E	26	5	E	984.99717	2	.653047E-02	0.9	0.6435E-02	1.46	3.177E-04			1.0
22	7	E	21	8	E	985.03071	-5			0.8144E-03					
15	2	A_2	16	3	A_1	985.04507	10	.201664E-01	0.2	0.1015E-01	-.65	3.734E-04	-.120E+00	-.394E+00	1.8
15	2	A_1	16	3	A_2	985.04507	8			0.1015E-01					
36	1	A_1	37	0	A_2	985.06161	8	.394219E-02	1.0	0.4058E-02	-2.94	2.082E-04			1.0
25	4	E	25	5	E	985.08259	3	.687054E-02	0.5	0.6939E-02	-1.00	3.249E-04	-.353E+00	-.156E+00	1.5
24	4	E	24	5	E	985.16473	6	.730889E-02	0.2	0.7435E-02	-1.73	3.636E-04			1.0
22	4	E	22	5	E	985.31907	6	.866643E-02	1.9	0.8371E-02	3.41	2.346E-04			1.0
39	9	E	38	10	E	985.33832	34			0.8074E-04					
29	0	E	30	1	E	985.35030	-5	.494530E-02	0.5	0.4287E-02	.62	3.880E-04	-.215E+00		1.3
43	2	E	44	1	E	985.35030	74			0.6276E-03					
21	4	E	21	5	E	985.39131	7	.852451E-02	0.3	0.8791E-02	-3.13	4.116E-04			1.0
20	4	E	20	5	E	985.46013	-5			0.9166E-02					
19	4	E	19	5	E	985.52575	-9			0.9484E-02					
18	4	E	18	5	E	985.58813	-7	.940714E-02	0.4	0.9735E-02	-3.48	4.052E-04			1.0
7	5	A_2	6	6	A_1	985.61688	-36			0.1209E-03					
7	5	A_1	6	6	A_2	985.61688	-36			0.1209E-03					
17	4	E	17	5	E	985.64719	-8	.953856E-02	0.5	0.9908E-02	-3.87	3.830E-04			1.0
31	8	A_2	30	9	A_1	985.68242	-22			0.3249E-03					
31	8	A_1	30	9	A_2	985.68242	-22			0.3249E-03					
16	4	E	16	5	E	985.70300	-6	.963974E-02	0.2	0.9991E-02	-3.65	3.478E-04	-.134E+00	-.430E+00	1.8
15	4	E	15	5	E	985.75552	-4	.943478E-02	1.6	0.9975E-02	-5.73	3.600E-04			1.0
14	4	E	14	5	E	985.80473	-5	.994469E-02	0.4	0.9850E-02	.96	4.572E-04			1.0
15	6	E	14	7	E	985.81152	-23	.109548E-02	3.7	0.1030E-02	5.99	3.350E-04			1.0
23	7	E	22	8	E	985.83661	-30	.772397E-03	4.3	0.8090E-03	-4.74	3.866E-04			1.0
13	4	E	13	5	E	985.85064	-7	.937736E-02	0.4	0.9605E-02	-2.42	4.154E-04			1.0
12	4	E	12	5	E	985.89328	-8	.895981E-02	0.8	0.9232E-02	-3.03	3.569E-04			1.0
11	4	E	11	5	E	985.93266	-7	.863883E-02	0.3	0.8722E-02	-.96	4.241E-04	.297E-01	.731E-01	1.0
10	4	E	10	5	E	985.96878	-3	.780161E-02	0.4	0.8066E-02	-3.39	3.583E-04			1.0
14	2	A_2	15	3	A_1	985.97885	-11	.208782E-01	0.2	0.1041E-01	.27	4.161E-04			1.0
14	2	A_1	15	3	A_2	985.97885	-9			0.1041E-01					
9	4	E	9	5	E	986.00156	-5	.700451E-02	0.2	0.7255E-02	-3.58	3.169E-04	-.160E+00	-.290E+00	1.9
8	4	E	8	5	E	986.03107	-6	.594866E-02	0.4	0.6275E-02	-5.49	2.870E-04	-.320E-01	-.360E+00	1.2
7	4	E	7	5	E	986.05732	-5	.493666E-02	0.5	0.5108E-02	-3.47	3.680E-04	-.115E+00		1.1
35	1	A_2	36	0	A_1	986.07311	-9	.446430E-02	0.6	0.4614E-02	-3.35	2.844E-04			1.0
6	4	E	6	5	E	986.08024	-9	.366933E-02	0.7	0.3722E-02	-1.43	4.279E-04			1.0
5	4	E	5	5	E	986.09991	-9	.199071E-02	2.2	0.2059E-02	-3.41	3.882E-04			1.0
21	1	E	22	2	E	986.19671	-6	.769606E-02	0.2	0.8036E-02	-4.41	3.776E-04	-.138E+00	-.127E+00	1.1
63	3	E	63	4	E	986.21753	45			0.1160E-04					
49	3	E	50	2	E	986.25612	3			0.1982E-03					
28	0	E	29	1	E	986.32567	-9	.459267E-02	1.0	0.4736E-02	-3.12	2.251E-04		-.527E+00	1.1
42	2	E	43	1	E	986.36120	-14			0.7393E-03					
32	8	A_2	31	9	A_1	986.45451	-27	.633098E-03	1.7	0.2999E-03	5.26	6.486E-04			1.0
32	8	A_1	31	9	A_2	986.45451	-27			0.2999E-03					
8	5	A_2	7	6	A_1	986.47683	-3	.613624E-03	2.4	0.3082E-03	-.46	5.469E-04			1.0

(cont.)

J	K_a	Γ	J'	K'_a	Γ'	$\nu^{a)}$	$\delta_\nu{}^{b)}$	$S_\nu^{\rm exp}(294.45)^{c)}$	$\Delta_S{}^{d)}$	$S_\nu^{\rm calc}(294.45)^{e)}$	$\delta_S{}^{f)}$	$\Gamma_0{}^{g)}$	$\Delta_2{}^{g)}$	$\nu_{\rm VC}{}^{g)}$	$R^{h)}$
1			2			3	4	5	6	7	8	9	10	11	12
8	5	A_1	7	6	A_2	986.47683	-3			0.3082E-03					
6	3	E	7	4	E	986.57821	-7	.936326E-02	0.3	0.1026E-01	-9.57	2.982E-04		-.317E+00	1.1
24	7	E	23	8	E	986.63932	-10			0.7949E-03					
16	6	E	15	7	E	986.64329	-11			0.1131E-02					
41	9	E	40	10	E	986.81486	-15			0.6198E-04					
60	3	E	60	4	E	986.83281	18			0.2533E-04					
13	2	A_2	14	3	A_1	986.90979	-6	.213711E-01	0.3	0.1061E-01	.71	3.666E-04	-.317E-01	-.474E+00	1.2
13	2	A_1	14	3	A_2	986.90979	-7			0.1061E-01					
34	1	A_1	35	0	A_2	987.08155	-11	.497547E-02	0.3	0.5220E-02	-4.92	2.881E-04			1.0
55	4	A_1	56	3	A_2	987.12755	-4			0.5058E-04					
55	4	A_2	56	3	A_1	987.12755	-5			0.5058E-04					
20	1	E	21	2	E	987.14870	-6	.807667E-02	0.3	0.8514E-02	-5.42	3.526E-04		-.582E+00	1.5
33	8	A_2	32	9	A_1	987.22279	-33	.582024E-03	2.7	0.2748E-03	5.56	6.917E-05			1.0
33	8	A_1	32	9	A_2	987.22279	-33			0.2748E-03					
48	3	E	49	2	E	987.28357	-9			0.2397E-03					
27	0	E	28	1	E	987.29817	-11	.538071E-02	0.4	0.5203E-02	3.30	3.688E-04			1.0
9	5	A_2	8	6	A_1	987.33301	-6	.104203E-02	2.5	0.5297E-03	-1.66	7.606E-04	-.923E-01	.328E+01	1.1
9	5	A_1	8	6	A_2	987.33301	-6			0.5297E-03					
41	2	E	42	1	E	987.37044	-12			0.8666E-03					
57	3	E	57	4	E	987.41753	-21			0.5300E-04					
25	7	E	24	8	E	987.43809	-16	.724540E-03	1.5	0.7732E-03	-6.72	2.827E-04	-.233E+00		1.1
17	6	E	16	7	E	987.47151	0	.117629E-02	3.0	0.1214E-02	-3.21	4.523E-04			1.0
5	3	E	6	4	E	987.48413	-7	.948207E-02	0.4	0.1012E-01	-6.68	3.217E-04			1.0
42	9	E	41	10	E	987.54750	-10			0.5383E-04					
56	3	E	56	4	E	987.60589	-14			0.6714E-04					
12	2	A_2	13	3	A_1	987.83763	-7	.217168E-01	0.2	0.1074E-01	1.08	4.177E-04	-.124E+00	.281E+00	1.2
12	2	A_1	13	3	A_2	987.83763	-6			0.1074E-01					
54	3	E	54	4	E	987.97224	-27			0.1062E-03					
34	8	A_2	33	9	A_1	987.98738	-25			0.2502E-03					
34	8	A_1	33	9	A_2	987.98738	-25			0.2502E-03					
33	1	A_2	34	0	A_1	988.08677	-11	.564422E-02	0.5	0.5876E-02	-4.11	3.613E-04			1.0
19	1	E	20	2	E	988.09771	-6	.846092E-02	0.4	0.8967E-02	-5.98	4.141E-04			1.0
53	3	E	53	4	E	988.15059	-12			0.1327E-03					
54	4	A_1	55	3	A_2	988.17013	-3			0.6279E-04					
54	4	A_2	55	3	A_1	988.17013	-3			0.6279E-04					
10	5	A_2	9	6	A_1	988.18586	0	.154666E-02	0.9	0.7646E-03	1.13	3.325E-04		-.977E+00	1.6
10	5	A_1	9	6	A_2	988.18586	0			0.7646E-03					
26	7	E	25	8	E	988.23306	-34	.697041E-03	1.2	0.7451E-03	-6.90	4.477E-04	-.325E+00	-.731E+00	1.2
26	0	E	27	1	E	988.26782	-9	.560346E-02	0.7	0.5685E-02	-1.46	3.175E-04			1.0
18	6	E	17	7	E	988.29598	-9	.122895E-02	0.7	0.1278E-02	-4.00	3.793E-04	-.225E+00	-.497E+00	1.4
47	3	E	48	2	E	988.30875	4			0.2884E-03					
52	3	E	52	4	E	988.32542	-13			0.1649E-03					
40	2	E	41	1	E	988.37726	6	.106595E-02	2.1	0.1011E-02	5.16	4.232E-04			1.0
4	3	E	5	4	E	988.38688	-5	.103232E-01	0.2	0.9987E-02	3.26	4.176E-04			1.0
51	3	E	51	4	E	988.49674	-30			0.2040E-03					
50	3	E	50	4	E	988.66505	-13			0.2512E-03					
35	8	A_1	34	9	A_2	988.74815	-16			0.2262E-03					
35	8	A_2	34	9	A_1	988.74815	-16			0.2262E-03					
11	2	A_2	12	3	A_1	988.76239	-6	.218358E-01	0.3	0.1080E-01	1.09	3.512E-04	-.246E+00	-.198E+00	1.4
11	2	A_1	12	3	A_2	988.76239	-7			0.1080E-01					
49	3	E	49	4	E	988.82988	-9			0.3077E-03					
48	3	E	48	4	E	988.99130	-12			0.3751E-03					
44	9	E	43	10	E	989.00061	-19			0.3992E-04					
27	7	E	26	8	E	989.02478	-8	.683539E-03	5.2	0.7117E-03	-4.12	4.757E-04			1.0
11	5	A_2	10	6	A_1	989.03512	-10	.196397E-02	1.8	0.9990E-03	-1.73	3.332E-04			1.0
11	5	A_1	10	6	A_2	989.03512	-10			0.9990E-03					
18	1	E	19	2	E	989.04374	-6	.883902E-02	0.5	0.9385E-02	-6.17	3.949E-04			1.0
32	1	A_1	33	0	A_2	989.08877	-10	.630750E-02	0.4	0.6581E-02	-4.33	3.129E-04			1.0
19	6	E	18	7	E	989.11699	-7	.129337E-02	0.8	0.1323E-02	-2.32	5.110E-04	-.852E+00	.541E+00	1.7
47	3	E	47	4	E	989.14947	-6			0.4551E-03					
53	4	A_1	54	3	A_2	989.21016	-11			0.7760E-04					
53	4	A_2	54	3	A_1	989.21016	-11			0.7760E-04					
25	0	E	26	1	E	989.23454	-8	.587905E-02	0.3	0.6177E-02	-5.06	3.825E-04			1.0
3	3	E	4	4	E	989.28640	-7	.937477E-02	0.3	0.9941E-02	-6.04	2.598E-04	-.385E-01	-.380E+00	1.4
46	3	E	46	4	E	989.30418	-12			0.5494E-03					
46	3	E	47	2	E	989.33110	-13			0.3455E-03					
39	2	E	40	1	E	989.38107	-17	.113060E-02	1.4	0.1174E-02	-3.81	2.301E-04	.237E+00		1.1
45	3	E	45	4	E	989.45563	-10			0.6601E-03					
36	8	A_2	35	9	A_1	989.50483	-31	.429338E-03	4.4	0.2032E-03	5.34	1.403E-04			1.0
36	8	A_1	35	9	A_2	989.50483	-31			0.2032E-03					
44	3	E	44	4	E	989.60377	-6			0.7891E-03					
10	2	A_2	11	3	A_1	989.68405	-8	.229955E-01	0.2	0.1078E-01	6.23	3.938E-04			1.0
10	2	A_1	11	3	A_2	989.68405	-7			0.1078E-01					
45	9	E	44	10	E	989.72113	-26			0.3409E-04					
43	3	E	43	4	E	989.74854	-7			0.9386E-03					
28	7	E	27	8	E	989.81246	-14	.675232E-03	2.2	0.6742E-03	.15	2.338E-04			1.0
12	5	A_2	11	6	A_1	989.88106	-7	.233450E-02	1.6	0.1223E-02	-4.77	4.144E-04			1.0
12	5	A_1	11	6	A_2	989.88106	-7			0.1223E-02					
42	3	E	42	4	E	989.88994	-11	.119974E-02	1.0	0.1111E-02	7.40	2.777E-04			1.0
20	6	E	19	7	E	989.93425	-23	.131591E-02	0.9	0.1351E-02	-2.63	5.774E-04			1.0
17	1	E	18	2	E	989.98675	-8	.952081E-02	0.2	0.9759E-02	-2.50	4.117E-04			1.0
41	3	E	41	4	E	990.02815	-3	.143692E-02	1.0	0.1308E-02	8.96	1.898E-04		-.705E+00	1.3

(cont.)

J	K_a	Γ	J'	K'_a	Γ'	$\nu^{a)}$	$\delta_\nu{}^{b)}$	$S_\nu^{\rm exp}(294.45)^{c)}$	$\Delta_S{}^{d)}$	$S_\nu^{\rm calc}(294.45)^{e)}$	$\delta_S{}^{f)}$	$\Gamma_0{}^{g)}$	$\Delta_2{}^{g)}$	$\nu_{\rm VC}{}^{g)}$	$R^{h)}$
	1			2		3	4	5	6	7	8	9	10	11	12
31	1	A_2	32	0	A_1	990.08753	-6	.697780E-02	0.5	0.7332E-02	-5.08	2.931E-04	.132E+00	-.160E+00	1.1
40	3	E	40	4	E	990.16289	-9	.160498E-02	1.0	0.1533E-02	4.49	6.050E-05			1.0
24	0	E	25	1	E	990.19837	-4	.641382E-02	0.2	0.6672E-02	-4.03	3.292E-04	-.121E+00	-.429E+00	1.4
37	8	A_2	36	9	A_1	990.25787	-23	.381363E-03	3.2	0.1814E-03	4.86	3.090E-04			1.0
37	8	A_1	36	9	A_2	990.25787	-23			0.1814E-03					
39	3	E	39	4	E	990.29434	-12	.190858E-02	1.2	0.1787E-02	6.37	3.035E-04			1.0
45	3	E	46	2	E	990.35121	0	.416209E-03	2.3	0.4119E-03	1.04	3.059E-04			1.0
38	2	E	39	1	E	990.38264	-3	.132574E-02	1.1	0.1356E-02	-2.27	2.575E-04			1.0
38	3	E	38	4	E	990.42251	-12	.221769E-02	0.5	0.2072E-02	6.55	2.309E-04			1.0
46	9	E	45	10	E	990.43793	-2			0.2895E-04					
37	3	E	37	4	E	990.54740	-8	.254001E-02	0.4	0.2391E-02	5.86	2.205E-04	-.737E-01	-.337E+00	1.2
29	7	E	28	8	E	990.59617	-44			0.6336E-03					
9	2	A_2	10	3	A_1	990.60264	-3	.219424E-01	0.2	0.1069E-01	2.59	3.767E-04			1.0
9	2	A_1	10	3	A_2	990.60264	-4			0.1069E-01					
36	3	E	36	4	E	990.66896	-6	.292317E-02	0.3	0.2745E-02	6.10	2.917E-04	-.704E-01	-.269E+00	1.2
13	5	A_2	12	6	A_1	990.72354	-3	.284856E-02	0.4	0.1429E-02	-.36	4.368E-04	-.272E+00		1.4
13	5	A_1	12	6	A_2	990.72354	-3			0.1429E-02					
35	3	E	35	4	E	990.78719	-6	.328142E-02	1.0	0.3134E-02	4.49	2.524E-04			1.0
34	3	E	34	4	E	990.90209	-8	.380867E-02	0.4	0.3560E-02	6.54	3.076E-04			1.0
16	1	E	17	2	E	990.92681	-4	.970182E-02	0.2	0.1008E-01	-3.91	3.744E-04	-.344E-01	-.471E+00	1.4
38	8	A_2	37	9	A_1	991.00715	-2	.342439E-03	6.7	0.1610E-03	5.98	2.681E-04			1.0
38	8	A_1	37	9	A_2	991.00715	-2			0.1610E-03					
33	3	E	33	4	E	991.01372	-6	.426811E-02	0.6	0.4021E-02	5.78	3.300E-04			1.0
30	1	A_1	31	0	A_2	991.08298	-6	.783055E-02	1.7	0.8127E-02	-3.78	1.518E-04			1.0
32	3	E	32	4	E	991.12206	-4	.479849E-02	0.5	0.4518E-02	5.84	3.127E-04	-.334E+00	-.225E+00	1.8
47	9	E	46	10	E	991.15066	19			0.2445E-04					
23	0	E	24	1	E	991.15896	-32			0.7166E-02					
31	3	E	31	4	E	991.22703	-8	.521165E-02	0.4	0.5049E-02	3.11	2.746E-04	-.164E+00	-.324E+00	1.3
51	4	A_1	52	3	A_2	991.28247	-64			0.1169E-03					
51	4	A_2	52	3	A_1	991.28247	-64			0.1169E-03					
30	3	E	30	4	E	991.32873	-9	.561453E-02	0.5	0.5612E-02	.05	2.037E-04		-.662E+00	1.8
44	3	E	45	2	E	991.36856	-7			0.4887E-03					
30	7	E	29	8	E	991.37681	-6	.615752E-03	1.3	0.5910E-03	4.02	2.892E-04			1.0
37	2	E	38	1	E	991.38133	-13	.154643E-02	0.4	0.1558E-02	-.78	3.313E-04			1.0
6	4	E	5	5	E	991.39873	8			0.1889E-03					
29	3	E	29	4	E	991.42745	22			0.6201E-02					
8	2	A_2	9	3	A_1	991.51809	-2	.212389E-01	1.3	0.1052E-01	.97	2.124E-04			1.0
8	2	A_1	9	3	A_2	991.51809	-1			0.1052E-01					
28	3	E	28	4	E	991.52224	-10	.667883E-02	1.3	0.6814E-02	-2.02	2.124E-04			1.0
22	6	E	21	7	E	991.55855	4	.137926E-02	0.7	0.1354E-02	1.80	3.685E-04			1.0
14	5	A_2	13	6	A_1	991.56248	-6	.311819E-02	0.7	0.1613E-02	-3.48	3.685E-04			1.0
14	5	A_1	13	6	A_2	991.56248	-6			0.1613E-02					
27	3	E	27	4	E	991.61411	-5	.733234E-02	0.3	0.7444E-02	-1.52	2.849E-04	-.130E+00	-.422E+00	1.6
26	3	E	26	4	E	991.70258	-11	.789053E-02	0.7	0.8084E-02	-2.45	4.505E-04	-.844E+00	-.313E+00	2.0
39	8	A_1	38	9	A_2	991.75206	-28			0.1420E-03					
39	8	A_2	38	9	A_1	991.75206	-28			0.1420E-03					
25	3	E	25	4	E	991.78788	-5	.859426E-02	0.3	0.8726E-02	-1.53	3.305E-04			1.0
15	1	E	16	2	E	991.86381	-3	.999204E-02	0.4	0.1034E-01	-3.52	3.865E-04			1.0
24	3	E	24	4	E	991.86985	-2	.942381E-02	0.4	0.9361E-02	.66	3.781E-04			1.0
23	3	E	23	4	E	991.94846	-7	.101346E-01	0.3	0.9979E-02	1.53	3.659E-04			1.0
22	3	E	22	4	E	992.02385	-5	.103216E-01	0.3	0.1057E-01	-2.39	3.173E-04	-.807E-01	-.502E+00	1.3
29	1	A_2	30	0	A_1	992.07517	-3	.858006E-02	0.3	0.8959E-02	-4.42	4.345E-04	.184E+00	.308E+00	1.4
21	3	E	21	4	E	992.09592	-6	.111133E-01	0.4	0.1112E-01	-.04	3.876E-04			1.0
22	0	E	23	1	E	992.11712	-8	.749613E-02	0.6	0.7650E-02	-2.06	4.197E-04	-.131E+00	.271E+00	1.1
31	7	E	30	8	E	992.15321	-17	.561258E-03	6.0	0.5472E-03	2.50	2.579E-04			1.0
20	3	E	20	4	E	992.16474	-4	.113360E-01	0.2	0.1161E-01	-2.45	3.556E-04	-.137E+00	-.308E+00	1.6
19	3	E	19	4	E	992.23024	-6	.116804E-01	0.4	0.1204E-01	-3.12	4.047E-04			1.0
7	4	E	6	5	E	992.26176	-10			0.4738E-03					
18	3	E	18	4	E	992.29249	-4	.119217E-01	0.4	0.1240E-01	-3.98	3.181E-04		-.548E+00	1.3
50	4	A_1	51	3	A_2	992.31566	-15			0.1425E-03					
50	4	A_2	51	3	A_1	992.31566	-15			0.1425E-03					
17	3	E	17	4	E	992.35143	-5	.120870E-01	0.4	0.1266E-01	-4.70	4.283E-04			1.0
23	6	E	22	7	E	992.36469	-40			0.1334E-02					
36	2	E	37	1	E	992.37750	-11			0.1782E-02					
43	3	E	44	2	E	992.38338	-9			0.5771E-03					
15	5	A_2	14	6	A_1	992.39797	-4	.352736E-02	2.2	0.1772E-02	-.46	3.423E-04			1.0
15	5	A_1	14	6	A_2	992.39797	-4		2.2	0.1772E-02		3.423E-04			1.0
16	3	E	16	4	E	992.40711	-4	.124034E-01	0.7	0.1281E-01	-3.28	3.980E-04			1.0
7	2	A_2	8	3	A_1	992.43036	-4	.217180E-01	0.2	0.1027E-01	5.39	3.370E-04	-.422E-01	-.111E+00	1.1
7	2	A_1	8	3	A_2	992.43036	-4			0.1027E-01					
15	3	E	15	4	E	992.45950	-4	.124269E-01	0.2	0.1285E-01	-3.38	3.461E-04	-.117E+00	-.450E+00	1.6
40	8	A_1	39	9	A_2	992.49350	-9			0.1245E-03					
40	8	A_2	39	9	A_1	992.49350	-9			0.1245E-03					
14	3	E	14	4	E	992.50860	-5	.126038E-01	0.2	0.1276E-01	-1.21	3.996E-04			1.0
13	3	E	13	4	E	992.55445	-4	.121711E-01	0.6	0.1253E-01	-2.92	3.696E-04			1.0
12	3	E	12	4	E	992.59699	-5	.118075E-01	0.4	0.1215E-01	-2.89	3.743E-04			1.0
11	3	E	11	4	E	992.63628	-4	.111869E-01	0.3	0.1162E-01	-3.83	3.261E-04		-.532E+00	1.5
10	3	E	10	4	E	992.67231	-2	.109051E-01	0.4	0.1092E-01	-.12	3.943E-04	.100E+00	-.274E+00	1.2
9	3	E	9	4	E	992.70502	-4	.974796E-02	0.4	0.1005E-01	-3.09	3.323E-04	-.139E+00	-.470E+00	1.4
8	3	E	8	4	E	992.73450	-1	.906950E-02	0.4	0.9001E-02	.76	3.810E-04	-.958E-01	-.989E-01	1.1
6	3	E	6	4	E	992.78355	-5	.618550E-02	0.6	0.6310E-02	-2.00	3.468E-04			1.0
14	1	E	15	2	E	992.79774	-6	.991192E-02	0.4	0.1054E-01	-6.32	3.729E-04			1.0

J	K_a	Γ	J'	K'_a	Γ'	$\nu^{a)}$	$\delta_\nu{}^{b)}$	$S_\nu^{\exp}(294.45)^{c)}$	$\Delta_S{}^{d)}$	$S_\nu^{\mathrm{calc}}(294.45)^{e)}$	$\delta_S{}^{f)}$	$\Gamma_0{}^{g)}$	$\Delta_2{}^{g)}$	$\nu_{\mathrm{VC}}{}^{g)}$	$R^{h)}$
1			2			3	4	5	6	7	8	9	10	11	12
5	3	E	5	4	E	992.80326	2	.441329E-02	1.1	0.4607E-02	-4.38	3.389E-04			1.0
4	3	E	4	4	E	992.81960	0	.261970E-02	0.7	0.2570E-02	1.91	4.792E-04	-.258E+00		1.2
32	7	E	31	8	E	992.92592	-18	.526942E-03	1.2	0.5032E-03	4.51	1.746E-04	-.173E+00		1.0
28	1	A_1	29	0	A_2	993.06396	-10	.930782E-02	0.4	0.9825E-02	-5.55	3.856E-04			1.0
21	0	E	22	1	E	993.07213	-3	.776866E-02	0.5	0.8118E-02	-4.49	4.232E-04			1.0
8	4	E	7	5	E	993.12202	33			0.8050E-03					
24	6	E	23	7	E	993.16777	-26			0.1301E-02					
16	5	A_2	15	6	A_1	993.22998	1	.40263E-02	0.3	0.1902E-02	0.13	4.127E-04			1.0
16	5	A_1	15	6	A_2	993.22998	1			0.1902E-02					
41	8	A_2	40	9	A_1	993.22998	-93			0.1085E-03					
41	8	A_1	40	9	A_2	993.22998	-93			0.1085E-03					
50	9	E	49	10	E	993.26359	4			0.1427E-04					
6	2	A_2	7	3	A_1	993.33950	-4	.205239E-01	0.8	0.9966E-02	2.88	3.014E-04			1.0
6	2	A_1	7	3	A_2	993.33950	-4			0.9966E-02					
49	4	A_2	50	3	A_1	993.34558	-42			0.1729E-03					
49	4	A_1	50	3	A_2	993.34558	-42			0.1729E-03					
35	2	E	36	1	E	993.37098	-11	.201667E-02	1.5	0.2028E-02	-.57	3.280E-04			1.0
42	3	E	43	2	E	993.39567	-6			0.6782E-03					
33	7	E	32	8	E	993.69491	-12			0.4595E-03					
13	1	E	14	2	E	993.72867	-3	.102211E-01	0.2	0.1066E-01	-4.28	3.593E-04	-.286E-01	-.420E+00	1.6
25	6	E	24	7	E	993.96727	-4			0.1257E-02					
9	4	E	8	5	E	993.97809	-3			0.1153E-02					
58	2	A_1	58	3	A_2	993.99852	2			0.4886E-04					
20	0	E	21	1	E	994.02412	-4	.848234E-02	0.3	0.8560E-02	-.91	4.290E-04	.689E-01	-.166E+00	1.1
27	1	A_2	28	0	A_1	994.04955	-5	.103290E-01	0.4	0.1071E-01	-3.73	3.618E-04			1.0
17	5	A_2	16	6	A_1	994.05839	-1	.411629E-02	0.9	0.2005E-02	2.61	5.516E-04			1.0
17	5	A_1	16	6	A_2	994.05839	-1			0.2005E-02					
57	2	A_1	57	3	A_2	994.18298	51			0.6224E-04					
57	2	A_2	57	3	A_1	994.18985	-8			0.6224E-04					
5	2	A_2	6	3	A_1	994.24547	-5	.212501E-01	0.2	0.9607E-02	9.58	3.222E-04	.689E-01	-.359E+00	1.3
5	2	A_1	6	3	A_2	994.24547	-5			0.9607E-02					
55	5	E	56	4	E	994.26963	-14			0.4229E-04					
34	2	E	35	1	E	994.36182	-7	.236828E-02	0.6	0.2296E-02	3.05	2.208E-04		-.405E+00	1.2
48	4	A_1	49	3	A_2	994.37356	-12			0.2087E-03					
48	4	A_2	49	3	A_1	994.37356	-12			0.2087E-03					
56	2	A_1	56	3	A_2	994.37805	11			0.7889E-04					
41	3	E	42	2	E	994.40526	-12	.772225E-03	1.4	0.7931E-03	-2.71	1.579E-04	-.209E+00		1.1
34	7	E	33	8	E	994.46011	-4			0.4169E-03					
55	2	A_1	55	3	A_2	994.55558	-24			0.9953E-04					
12	1	E	13	2	E	994.65650	-4	.102262E-01	0.3	0.1070E-01	-4.60	3.872E-04		-.228E+00	1.0
43	8	A_1	42	9	A_2	994.69356	-11			0.8107E-04					
43	8	A_2	42	9	A_1	994.69356	-11			0.8107E-04					
54	2	A_2	54	3	A_1	994.73727	-19			0.1250E-03					
54	2	A_1	54	3	A_2	994.74380	-1			0.1250E-03					
26	6	E	25	7	E	994.76280	-12	.117585E-02	1.2	0.1204E-02	-2.40	3.474E-04	-.286E+00		1.2
10	4	E	9	5	E	994.83111	-3	.147846E-02	0.6	0.1497E-02	-1.28	3.125E-04	.327E-01	-.289E+00	1.1
18	5	A_2	17	6	A_1	994.88326	-3	.413309E-02	0.2	0.2078E-02	-.56	4.000E-04	-.134E+00	-.398E-01	1.2
18	5	A_1	17	6	A_2	994.88326	-3			0.2078E-02					
53	2	A_1	53	3	A_2	994.91588	13			0.1562E-03					
53	2	A_2	53	3	A_1	994.92142	-26			0.1562E-03					
19	0	E	20	1	E	994.97313	-5	.861013E-02	0.3	0.8968E-02	-4.15	3.607E-04	-.217E+00	-.306E+00	1.5
26	1	A_1	27	0	A_2	995.03175	-6	.111030E-01	0.3	0.1162E-01	-4.66	3.780E-04			1.0
52	2	A_2	52	3	A_1	995.09073	5			0.1942E-03					
52	2	A_1	52	3	A_2	995.09587	-30			0.1942E-03					
4	2	A_2	5	3	A_1	995.14828	-3	.183413E-01	0.1	0.9220E-02	-.54	2.830E-04	-.545E-01	-.483E+00	2.0
4	2	A_1	5	3	A_2	995.14828	-3			0.9220E-02					
35	7	E	34	8	E	995.22139	-5			0.3759E-03					
51	2	A_1	51	3	A_2	995.26228	3			0.2404E-03					
51	2	A_2	51	3	A_1	995.26711	-20			0.2404E-03					
54	5	E	55	4	E	995.31227	-6			0.5245E-04					
33	2	E	34	1	E	995.34991	-7	.263548E-02	0.4	0.2586E-02	1.89	2.853E-04	-.232E+00	-.442E+00	1.8
47	4	A_1	48	3	A_2	995.39869	-14			0.2509E-03					
47	4	A_2	48	3	A_1	995.39869	-14			0.2509E-03					
40	3	E	41	2	E	995.41228	-13			0.9230E-03					
44	8	A_1	43	9	A_2	995.41893	-15			0.6949E-04					
44	8	A_2	43	9	A_1	995.41893	-15			0.6949E-04					
50	2	A_2	50	3	A_1	995.43044	-2			0.2960E-03					
50	2	A_1	50	3	A_2	995.43494	-16			0.2960E-03					
27	6	E	26	7	E	995.55477	-8	.113625E-02	1.4	0.1144E-02	-.68	3.279E-04			1.0
11	1	E	12	2	E	995.58127	-4	.103401E-01	0.4	0.1065E-01	-2.99	3.639E-04	.218E-01	-.472E+00	1.3
49	2	A_1	49	3	A_2	995.59524	-8			0.3628E-03					
49	2	A_2	49	3	A_1	995.59944	-10			0.3629E-03					
11	4	E	10	5	E	995.68069	-4	.181446E-02	2.1	0.1826E-02	-.62	3.258E-04			1.0
19	5	A_2	18	6	A_1	995.70458	-5	.416643E-02	0.4	0.2124E-02	-1.95	4.129E-04	-.259E+00	-.357E-01	1.5
19	5	A_1	18	6	A_2	995.70458	-5			0.2124E-02					
48	2	A_2	48	3	A_1	995.75683	1			0.4426E-03					
48	2	A_1	48	3	A_2	995.76111	47			0.4426E-03					
47	2	A_1	47	3	A_2	995.91526	28			0.5372E-03					
47	2	A_2	47	3	A_1	995.91847	6			0.5372E-03					
18	0	E	19	1	E	995.91911	-10	.959691E-02	0.2	0.9333E-02	2.75	4.421E-04			1.0
36	7	E	35	8	E	995.97878	-12			0.3368E-03					
25	1	A_2	26	0	A_1	996.01036	-32	.130299E-01	0.6	0.1253E-01	3.83	6.367E-04	-.405E+00		1.3

(cont.)

J	K_a	Γ	J'	K'_a	Γ'	$\nu^{a)}$	$\delta_\nu{}^{b)}$	$S_\nu^{\mathrm{exp}}(294.45)^{c)}$	$\Delta_S{}^{d)}$	$S_\nu^{\mathrm{calc}}(294.45)^{e)}$	$\delta_S{}^{f)}$	$\Gamma_0{}^{g)}$	$\Delta_2{}^{g)}$	$\nu_{\mathrm{VC}}{}^{g)}$	$R^{h)}$
1			2			3	4	5	6	7	8	9	10	11	12
3	2	A_2	4	3	A_1	996.04788	-4	.177172E-01	0.2	0.8849E-02	.11	2.892E-04		-.453E+00	1.7
3	2	A_1	4	3	A_2	996.04788	-4			0.8849E-02					
46	2	A_2	46	3	A_1	996.06957	-21			0.6489E-03					
46	2	A_1	46	3	A_2	996.07290	6			0.6489E-03					
45	8	A_2	44	9	A_1	996.14032	-17			0.5924E-04					
45	8	A_1	44	9	A_2	996.14032	-17			0.5924E-04					
45	2	A_1	45	3	A_2	996.22109	-14			0.7799E-03					
45	2	A_2	45	3	A_1	996.22401	6			0.7799E-03					
32	2	E	33	1	E	996.33532	-5	.292960E-02	0.5	0.2897E-02	1.12	3.286E-04			1.0
28	6	E	27	7	E	996.34297	-9	.106009E-02	1.1	0.1078E-02	-1.72	3.669E-04			1.0
53	5	E	54	4	E	996.35203	-39			0.6477E-04					
44	2	A_2	44	3	A_1	996.36907	-26			0.9327E-03					
44	2	A_1	44	3	A_2	996.37188	15			0.9327E-03					
39	3	E	40	2	E	996.41673	-8	.112898E-02	0.8	0.1069E-02	5.32	1.964E-04			1.0
46	4	A_2	47	3	A_1	996.42128	-15	.592147E-03	1.8	0.3001E-03	-1.37	3.816E-04			1.0
46	4	A_1	47	3	A_2	996.42128	-15			0.3001E-03					
10	1	E	11	2	E	996.50293	-5	.103210E-01	0.3	0.1051E-01	-1.86	4.953E-04	-.486E+00	.501E+00	1.2
43	2	A_2	43	3	A_1	996.51614	-6			0.1110E-02					
20	5	A_1	19	6	A_2	996.52239	0			0.2143E-02					
20	5	A_2	19	6	A_1	996.52239	0			0.2143E-02					
12	4	E	11	5	E	996.52671	-17			0.2128E-02					
42	2	A_2	42	3	A_1	996.65562	10			0.1314E-02					
37	7	E	36	8	E	996.73227	-22			0.2999E-03					
41	2	A_1	41	3	A_2	996.79435	74			0.1549E-02					
17	0	E	18	1	E	996.86220	-4	.939113E-02	0.5	0.9647E-02	-2.72	4.175E-04	-.498E+00	-.214E+00	1.2
40	2	A_2	40	3	A_1	996.92907	71			0.1815E-02					
40	2	A_1	40	3	A_2	996.92907	-63			0.1815E-02					
2	2	A_2	3	3	A_1	996.94427	-4	.172617E-01	0.2	0.8589E-02	.48	2.740E-04		-.548E+00	1.8
2	2	A_1	3	3	A_2	996.94427	-4			0.8589E-02					
24	1	A_1	25	0	A_2	996.98613	-5	.127475E-01	0.3	0.1344E-01	-5.40	3.329E-04		-.470E+00	1.5
39	2	A_1	39	3	A_2	997.06024	46			0.2117E-02					
39	2	A_2	39	3	A_1	997.06024	-68			0.2117E-02					
29	6	E	28	7	E	997.12739	-17	.982866E-03	0.6	0.1009E-02	-2.65	3.477E-04	-.235E+00	-.138E+00	1.4
38	2	A_2	38	3	A_1	997.18826	39	.528984E-02	0.6	0.2457E-02	7.12	7.854E-04			1.0
38	2	A_1	38	3	A_2	997.18826	-57			0.2457E-02					
5	3	E	4	4	E	997.23619	29			0.2876E-03					
37	2	A_2	37	3	A_1	997.31295	-48	.601107E-02	0.4	0.2836E-02	5.64	8.123E-04			1.0
37	2	A_1	37	3	A_2	997.31295	31			0.2836E-02					
31	2	E	32	1	E	997.31793	-9			0.3228E-02					
21	5	A_2	20	6	A_1	997.33652	-5	.418278E-02	0.7	0.2138E-02	-2.24	3.943E-04	-.824E+00	-.946E-01	2.0
21	5	A_1	20	6	A_2	997.33652	-5			0.2138E-02					
52	5	E	53	4	E	997.39034	29			0.7960E-04					
9	1	E	10	2	E	997.42153	-1	.102947E-01	1.2	0.1028E-01	.10	6.660E-04			1.0
36	2	A_2	36	3	A_1	997.43432	23	.689993E-02	1.9	0.3257E-02	5.60	5.631E-04			1.0
36	2	A_1	36	3	A_2	997.43432	-42			0.3257E-02					
45	4	A_2	46	3	A_1	997.44127	-20			0.3573E-03					
45	4	A_1	46	3	A_2	997.44127	-20			0.3573E-03					
38	7	E	37	8	E	997.48187	-34			0.2654E-03					
35	2	A_2	35	3	A_1	997.55241	-33	.777126E-02	0.8	0.3720E-02	4.25	5.703E-04			1.0
35	2	A_1	35	3	A_2	997.55241	19			0.3720E-02					
47	8	A_2	46	9	A_1	997.57128	3			0.4237E-04					
47	8	A_1	46	9	A_2	997.57128	3			0.4237E-04					
34	2	A_2	34	3	A_1	997.66717	14	.882073E-02	0.4	0.4228E-02	4.14	3.678E-04		-.877E+00	1.8
34	2	A_1	34	3	A_2	997.66717	-28			0.4228E-02					
33	2	A_1	33	3	A_2	997.77833	-21			0.4779E-02					
33	2	A_2	33	3	A_1	997.77833	-53			0.4779E-02					
16	0	E	17	1	E	997.80222	-3	.943699E-02	0.4	0.9900E-02	-4.91	3.627E-04	-.151E+00	-.401E+00	1.3
32	2	A_2	32	3	A_1	997.88677	4	.111172E-01	0.3	0.5373E-02	3.34	3.308E-04		-.829E+00	1.6
32	2	A_1	32	3	A_2	997.88677	-20			0.5373E-02					
30	6	E	29	7	E	997.90819	-12	.941875E-03	1.0	0.9372E-03	.49	1.599E-04		-.775E+00	1.4
23	1	A_2	24	0	A_1	997.95825	-6	.139668E-01	2.1	0.1432E-01	-2.53	1.937E-04			1.0
31	2	A_2	31	3	A_1	997.99165	-14	.123277E-01	0.3	0.6008E-02	2.54	3.721E-04	-.230E+00	-.454E+00	1.9
31	2	A_1	31	3	A_2	997.99165	4			0.6008E-02					
30	2	A_2	30	3	A_1	998.09319	0	.136497E-01	0.3	0.6680E-02	2.12	4.004E-04			1.0
30	2	A_1	30	3	A_2	998.09319	-13			0.6680E-02					
6	3	E	5	4	E	998.10261	-3	.674130E-03	6.3	0.7042E-03	-4.46	6.924E-04			1.0
22	5	A_2	21	6	A_1	998.14709	-6	.415317E-02	0.5	0.2111E-02	-1.65	4.178E-04			1.0
22	5	A_1	21	6	A_2	998.14709	-6			0.2111E-02					
29	2	A_2	29	3	A_1	998.19147	-9	.150381E-01	0.3	0.7387E-02	1.75	3.365E-04	-.448E-01	-.389E+00	1.5
29	2	A_1	29	3	A_2	998.19147	-1			0.7387E-02					
14	4	E	13	5	E	998.20886	7	.274100E-02	0.7	0.2632E-02	3.98	4.012E-04			1.0
39	7	E	38	8	E	998.22779	-25			0.2336E-03					
48	8	A_2	47	9	A_1	998.28054	-1			0.3555E-04					
48	8	A_1	47	9	A_2	998.28054	-1			0.3555E-04					
28	2	A_2	28	3	A_1	998.28643	-3	.166041E-01	1.7	0.8123E-02	2.16	2.794E-04			1.0
28	2	A_1	28	3	A_2	998.28643	-7			0.8123E-02					
30	2	E	31	1	E	998.29790	-2	.351994E-02	1.1	0.3577E-02	-1.63	4.278E-04			1.0
8	1	E	9	2	E	998.33696	-3	.973439E-02	0.2	0.9965E-02	-2.37	3.614E-04	-.179E+00	-.306E+00	1.5
27	2	A_2	27	3	A_1	998.37810	-6	.176807E-01	0.4	0.8880E-02	-.45	3.671E-04			1.0
27	2	A_1	27	3	A_2	998.37810	-5			0.8880E-02					
37	3	E	38	2	E	998.41756	-7			0.1412E-02					
51	5	E	52	4	E	998.42502	-18			0.9738E-04					

(cont.)

J	K_a	Γ	J'	K'_a	Γ'	$\nu^{a)}$	$\delta_\nu{}^{b)}$	$S_\nu^{\rm exp}(294.45)^{c)}$	$\Delta_S{}^{d)}$	$S_\nu^{\rm calc}(294.45)^{e)}$	$\delta_S{}^{f)}$	$\Gamma_0{}^{g)}$	$\Delta_2{}^{g)}$	$\nu_{\rm VC}{}^{g)}$	$R^{h)}$
	1			2		3	4	5	6	7	8	9	10	11	12
44	4	A_2	45	3	A_1	998.45900	6	.849419E-03	7.2	0.4233E-03	.32	1.831E-04			1.0
44	4	A_1	45	3	A_2	998.45900	5			0.4233E-03					
26	2	A_2	26	3	A_1	998.46648	-6	.191889E-01	0.3	0.9651E-02	-.59	3.622E-04			1.0
26	2	A_1	26	3	A_2	998.46648	-6			0.9651E-02					
25	2	A_2	25	3	A_1	998.55159	-3	.205691E-01	0.2	0.1043E-01	-1.39	3.532E-04	-.118E+00	-.178E+00	2.2
25	2	A_1	25	3	A_2	998.55159	-5			0.1043E-01					
24	2	A_2	24	3	A_1	998.63342	-4	.225855E-01	0.2	0.1120E-01	.85	4.145E-04			1.0
24	2	A_1	24	3	A_2	998.63342	-1			0.1120E-01					
31	6	E	30	7	E	998.68561	29			0.8646E-03					
23	2	A_2	23	3	A_1	998.71190	-5	.233266E-01	0.2	0.1195E-01	-2.44	3.616E-04	-.749E-01	-.127E+00	1.3
23	2	A_1	23	3	A_2	998.71190	-8			0.1195E-01					
15	0	E	16	1	E	998.73975	51			0.1008E-01					
22	2	A_2	22	3	A_1	998.78716	-6	.247108E-01	0.1	0.1267E-01	-2.54	4.028E-04			1.0
22	2	A_1	22	3	A_2	998.78716	-3			0.1267E-01					
21	2	A_2	21	3	A_1	998.85912	-2	.254887E-01	0.2	0.1334E-01	-4.71	3.602E-04	-.837E-01	-.404E+00	1.3
21	2	A_1	21	3	A_2	998.85912	-6			0.1334E-01					
22	1	A_1	23	0	A_2	998.92736	31			0.1517E-01					
20	2	A_1	20	3	A_2	998.92736	-46			0.1396E-01					
20	2	A_2	20	3	A_1	998.92736	-50			0.1396E-01					
23	5	A_2	22	6	A_1	998.95400	-11	.405769E-02	1.0	0.2063E-02	-1.71	3.784E-04			1.0
23	5	A_1	22	6	A_2	998.95400	-11			0.2063E-02					
40	7	E	39	8	E	998.96933	-63			0.2044E-03					
19	2	A_2	19	3	A_1	998.99320	-2	.290552E-01	0.2	0.1450E-01	.17	3.932E-04			1.0
19	2	A_1	19	3	A_2	998.99320	-6			0.1450E-01					
15	4	E	14	5	E	999.04452	-1	.274443E-02	2.4	0.2826E-02	-2.96	5.453E-04			1.0
18	2	A_2	18	3	A_1	999.05532	-6	.299621E-01	0.2	0.1496E-01	.17	3.935E-04			1.0
18	2	A_1	18	3	A_2	999.05532	-2			0.1496E-01					
17	2	A_2	17	3	A_1	999.11415	-4	.296526E-01	0.1	0.1530E-01	-3.22	3.993E-04			1.0
17	2	A_1	17	3	A_2	999.11415	-7			0.1530E-01					
16	2	A_2	16	3	A_1	999.16972	-7	.301658E-01	0.2	0.1553E-01	-2.98	4.072E-04			1.0
16	2	A_1	16	3	A_2	999.16972	-4			0.1553E-01					
15	2	A_2	15	3	A_1	999.22202	-4	.311615E-01	0.1	0.1563E-01	-.30	3.891E-04	.201E-01	-.191E+00	1.2
15	2	A_1	15	3	A_2	999.22202	-6			0.1563E-01					
7	1	E	8	2	E	999.24926	-5	.909580E-02	0.3	0.9555E-02	-5.05	3.095E-04	-.105E+00	-.444E+00	1.5
14	2	A_2	14	3	A_1	999.27106	-4	.305141E-01	2.3	0.1558E-01	-2.11	5.437E-04			1.0
14	2	A_1	14	3	A_2	999.27106	-2			0.1558E-01					
29	2	E	30	1	E	999.27495	-11			0.3943E-02					
13	2	A_2	13	3	A_1	999.31680	-3	.301950E-01	0.7	0.1537E-01	-1.83	3.780E-04	-.103E+01	.374E+00	1.4
13	2	A_1	13	3	A_2	999.31680	-4			0.1537E-01					
12	2	A_2	12	3	A_1	999.35927	-5	.298048E-01	0.1	0.1500E-01	-.68	3.848E-04			1.0
12	2	A_1	12	3	A_2	999.35927	-4			0.1500E-01					
11	2	A_2	11	3	A_1	999.39848	-4	.271743E-01	0.1	0.1446E-01	-6.43	3.465E-04	-.640E-01	-.211E+00	1.3
11	2	A_1	11	3	A_2	999.39848	-4			0.1446E-01					
36	3	E	37	2	E	999.41394	-10	.172993E-02	0.9	0.1611E-02	6.89	1.391E-04	-.138E+00	-.155E+00	1.2
10	2	A_2	10	3	A_1	999.43441	-5	.255818E-01	0.1	0.1374E-01	-7.41	3.612E-04	.291E-01	-.230E+00	1.1
10	2	A_1	10	3	A_2	999.43441	-4			0.1374E-01					
50	5	E	51	4	E	999.45836	51			0.1186E-03					
32	6	E	31	7	E	999.45836	-20			0.7923E-03					
9	2	A_2	9	3	A_1	999.46709	-3	.248214E-01	0.2	0.1283E-01	-3.40	3.631E-04			1.0
9	2	A_1	9	3	A_2	999.46709	-4			0.1283E-01					
43	4	A_2	44	3	A_1	999.47364	-19	.105201E-02	5.6	0.4992E-03	5.10	3.015E-04			1.0
43	4	A_1	44	3	A_2	999.47364	-19			0.4992E-03					
8	2	A_2	8	3	A_1	999.49649	-3	.222496E-01	0.3	0.1174E-01	-5.54	3.391E-04			1.0
8	2	A_1	8	3	A_2	999.49649	-3			0.1174E-01					
7	2	A_2	7	3	A_1	999.52261	-4	.199209E-01	0.3	0.1046E-01	-5.01	3.514E-04			1.0
7	2	A_1	7	3	A_2	999.52261	-4			0.1046E-01					
6	2	A_2	6	3	A_1	999.54547	-5	.172833E-01	0.3	0.8980E-02	-3.91	3.551E-04			1.0
6	2	A_1	6	3	A_2	999.54547	-5			0.8980E-02					
5	2	A_2	5	3	A_1	999.56509	-2	.138964E-01	0.3	0.7285E-02	-4.85	2.699E-04		-.601E+00	1.9
5	2	A_1	5	3	A_2	999.56509	-2			0.7285E-02					
4	2	A_2	4	3	A_1	999.58140	-4	.102959E-01	0.5	0.5333E-02	-3.60	3.397E-04	.491E-01	-.262E+00	1.1
4	2	A_1	4	3	A_2	999.58140	-4			0.5333E-02					
3	2	A_2	3	3	A_1	999.59444	-7	.610267E-02	0.3	0.3017E-02	1.14	4.427E-04	-.188E+00	-.508E+00	1.5
3	2	A_1	3	3	A_2	999.59444	-7			0.3017E-02					
14	0	E	15	1	E	999.67315	-4	.987562E-02	0.5	0.1019E-01	-3.20	3.850E-04			1.0
50	8	A_1	49	9	A_2	999.68647	-47			0.2463E-04					
50	8	A_2	49	9	A_1	999.68647	-47			0.2463E-04					
41	7	E	40	8	E	999.70776	-19			0.1778E-03					
24	5	A_2	23	6	A_1	999.75737	-6	.404426E-02	1.1	0.1999E-02	1.15	2.679E-04		-.496E+00	1.1
24	5	A_1	23	6	A_2	999.75737	-6			0.1999E-02					
8	3	E	7	4	E	999.82596	-6	.169261E-02	1.8	0.1666E-02	1.55	5.057E-04			1.0
16	4	E	15	5	E	999.87670	-6	.289916E-02	0.6	0.2978E-02	-2.73	4.834E-04	-.605E+00	-.458E+00	2.7
21	1	A_2	22	0	A_1	999.89233	-6	.150533E-01	0.2	0.1597E-01	-6.08	3.793E-04	-.305E+00	-.233E+00	2.5
6	1	E	7	2	E	1000.15849	2	.896278E-02	0.2	0.9061E-02	-1.10	3.558E-04	-.156E+00		1.2
33	6	E	32	7	E	1000.22786	-15	.734875E-03	1.6	0.7213E-03	1.85	2.253E-04			1.0
28	2	E	29	1	E	1000.24937	-4	.410847E-02	0.5	0.4322E-02	-5.20	3.505E-04	-.256E+00	-.212E+00	1.1
51	8	A_2	50	9	A_1	1000.38376	-23			0.2035E-04					
51	8	A_1	50	9	A_2	1000.38376	-23			0.2035E-04					
35	3	E	36	2	E	1000.40771	-3	.187024E-02	3.7	0.1828E-02	2.27	3.589E-04			1.0
42	7	E	41	8	E	1000.44181	-20			0.1538E-03					
42	4	A_2	43	3	A_1	1000.48612	1	.118033E-02	1.1	0.5857E-03	.75	5.811E-04			1.0
42	4	A_1	43	3	A_2	1000.48612	1			0.5857E-03					

(cont.)

J	K_a	Γ	J'	K'_a	Γ'	ν[a)]	δ_ν[b)]	$S_\nu^{\rm exp}(294.45)$[c)]	Δ_S[d)]	$S_\nu^{\rm calc}(294.45)$[e)]	δ_S[f)]	Γ_0[g)]	Δ_2[g)]	$\nu_{\rm VC}$[g)]	R[h)]
	1			2		3	4	5	6	7	8	9	10	11	12
25	5	A_2	24	6	A_1	1000.55708	-2	.398912E-02	0.4	0.1920E-02	3.74	4.048E-04			1.0
25	5	A_1	24	6	A_2	1000.55708	-2			0.1920E-02					
13	0	E	14	1	E	1000.60409	0	.103007E-01	0.4	0.1022E-01	.83	4.233E-04			1.0
9	3	E	8	4	E	1000.68262	-2	.211763E-02	0.6	0.2147E-02	-1.40	3.945E-04			1.0
17	4	E	16	5	E	1000.70548	1	.292080E-02	0.8	0.3090E-02	-5.81	6.044E-04	-.841E+00	.549E+00	1.6
58	1	E	58	2	E	1000.81583	36			0.5420E-04					
20	1	A_1	21	0	A_2	1000.85430	-1	.156781E-01	0.2	0.1670E-01	-6.53	3.927E-04	-.754E-01	-.141E+00	1.2
34	6	E	33	7	E	1000.99353	-13	.695924E-03	11.4	0.6525E-03	6.25	3.826E-04			1.0
57	1	E	57	2	E	1001.00728	4			0.6908E-04					
5	1	E	6	2	E	1001.06450	2	.815829E-02	0.2	0.8491E-02	-4.07	2.840E-04	-.192E+00	-.424E+00	2.4
43	7	E	42	8	E	1001.17187	-23			0.1323E-03					
56	1	E	56	2	E	1001.19561	0			0.8769E-04					
27	2	E	28	1	E	1001.22094	-3	.444841E-02	0.3	0.4710E-02	-5.89	3.144E-04	-.952E-01	-.353E+00	1.3
26	5	A_2	25	6	A_1	1001.35307	-4	.374202E-02	0.3	0.1829E-02	2.23	4.122E-04	-.244E+00	-.645E-01	1.9
26	5	A_1	25	6	A_2	1001.35307	-4			0.1829E-02					
55	1	E	55	2	E	1001.38051	-8			0.1107E-03					
34	3	E	35	2	E	1001.39871	-3	.200919E-02	0.9	0.2063E-02	-2.70	3.845E-04			1.0
55	6	E	56	5	E	1001.46199	25			0.3378E-04					
41	4	A_2	42	3	A_1	1001.49566	-12	.137132E-02	0.9	0.6839E-03	.26	2.512E-04	.537E-01	-.336E+00	1.1
41	4	A_1	42	3	A_2	1001.49566	-12			0.6839E-03					
48	5	E	49	4	E	1001.51572	10			0.1733E-03					
12	0	E	13	1	E	1001.53171	-21			0.1015E-01					
10	3	E	9	4	E	1001.53581	-4	.273375E-02	0.4	0.2603E-02	4.77	7.852E-04			1.0
54	1	E	54	2	E	1001.56197	-21			0.1392E-03					
53	1	E	53	2	E	1001.74059	21			0.1740E-03					
35	6	E	34	7	E	1001.75534	-16	.558676E-03	1.9	0.5866E-03	-5.00	1.421E-04		-.953E+00	1.2
53	8	A_2	52	9	A_1	1001.76567	-4			0.1367E-04					
53	8	A_1	52	9	A_2	1001.76567	-4			0.1367E-04					
19	1	A_2	20	0	A_1	1001.81279	-1	.164724E-01	0.2	0.1735E-01	-5.34	3.578E-04	-.102E+00	-.443E+00	2.0
44	7	E	43	8	E	1001.89803	-20			0.1133E-03					
52	1	E	52	2	E	1001.91528	8			0.2167E-03					
4	1	E	5	2	E	1001.96732	0	.755106E-02	0.4	0.7856E-02	-4.04	2.820E-04		-.384E+00	1.2
51	1	E	51	2	E	1002.08658	-7			0.2684E-03					
27	5	A_2	26	6	A_1	1002.14541	-3	.355591E-02	0.5	0.1729E-02	2.73	3.699E-04			1.0
27	5	A_1	26	6	A_2	1002.14541	-3			0.1729E-02					
26	2	E	27	1	E	1002.18970	-1	.486129E-02	0.3	0.5104E-02	-4.98	3.091E-04	-.169E+00	-.250E+00	1.6
50	1	E	50	2	E	1002.25468	-5			0.3309E-03					
19	4	E	18	5	E	1002.35228	0	.310619E-02	0.5	0.3196E-02	-2.90	3.800E-04	-.574E-01	-.347E+00	1.1
11	3	E	10	4	E	1002.38619	55	.555331E-02	0.5	0.3023E-02	3.85	6.908E-04	-.639E-01	-.860E+00	1.4
49	1	E	49	2	E	1002.41940	-4			0.4058E-03					
11	0	E	12	1	E	1002.45669	1	.959563E-02	0.4	0.9987E-02	-4.08	3.469E-04	.105E+00	-.447E+00	1.3
40	4	A_2	41	3	A_1	1002.50276	-5	.162034E-02	2.4	0.7946E-03	1.92	2.804E-04			1.0
40	4	A_1	41	3	A_2	1002.50276	-5			0.7946E-03					
36	6	E	35	7	E	1002.51334	-16	.519786E-03	2.2	0.5242E-03	-.85	3.062E-04			1.0
48	1	E	48	2	E	1002.58075	-4			0.4955E-03					
45	7	E	44	8	E	1002.61959	-77			0.9640E-04					
47	1	E	47	2	E	1002.73875	-4			0.6018E-03					
18	1	A_1	19	0	A_2	1002.76784	0	.170252E-01	0.4	0.1790E-01	-5.15	3.859E-04	-.611E+00		1.3
3	1	E	4	2	E	1002.86698	2	.704912E-02	0.4	0.7179E-02	-1.85	3.178E-04	-.263E+00	-.308E+00	1.4
46	1	E	46	2	E	1002.89339	-4	.788948E-03	2.2	0.7276E-03	7.77	1.694E-04	-.201E+00		1.1
28	5	A_2	27	6	A_1	1002.93402	-5	.335397E-02	0.5	0.1623E-02	3.22	3.283E-04		-.585E+00	1.4
28	5	A_1	27	6	A_2	1002.93402	-5			0.1623E-02					
45	1	E	45	2	E	1003.04467	-5	.943786E-03	0.8	0.8751E-03	7.28	3.128E-04	.746E-01	-.415E+00	1.1
4	2	A_2	3	3	A_1	1003.12800	-3	.871751E-03	1.6	0.4333E-03	.60	5.533E-04			1.0
4	2	A_1	3	3	A_2	1003.12800	-3			0.4333E-03					
25	2	E	26	1	E	1003.15561	-1	.545622E-02	0.6	0.5497E-02	-.75	4.134E-04			1.0
20	4	E	19	5	E	1003.17070	35			0.3194E-02					
44	1	E	44	2	E	1003.19262	-4	.108577E-02	0.6	0.1048E-02	3.52	1.027E-04		-.612E+00	1.3
12	3	E	11	4	E	1003.23217	18			0.3397E-02					
37	6	E	36	7	E	1003.26751	-14	.453606E-03	2.8	0.4656E-03	-2.65	1.553E-04	-.156E+00		1.0
43	1	E	43	2	E	1003.33734	8	.145784E-02	0.9	0.1247E-02	8.84	3.228E-04			1.0
46	7	E	45	8	E	1003.33734	-114			0.8161E-04					
32	3	E	33	2	E	1003.37245	-10	.263710E-02	0.9	0.2588E-02	1.86	1.703E-04			1.0
10	0	E	11	1	E	1003.37837	2	.992377E-02	0.2	0.9728E-02	1.98	4.115E-04			1.0
42	1	E	42	2	E	1003.47852	-1	.159137E-02	1.1	0.1478E-02	7.11	2.567E-04			1.0
39	4	A_1	40	3	A_2	1003.50729	9			0.9186E-03					
39	4	A_2	40	3	A_1	1003.50729	9			0.9186E-03					
53	6	E	54	5	E	1003.54408	-9			0.5165E-04					
46	5	E	47	4	E	1003.56315	-9			0.2487E-03					
41	1	E	41	2	E	1003.61675	29			0.1743E-02					
17	1	A_2	18	0	A_1	1003.71934	-8	.206472E-01	0.1	0.1834E-01	-3.45	4.026E-04	-.124E+00	-.357E+00	2.5
29	5	A_1	28	6	A_2	1003.71934	35			0.1512E-02					
29	5	A_2	28	6	A_1	1003.71934	35			0.1512E-02					
40	1	E	40	2	E	1003.75104	-2	.218717E-02	1.5	0.2044E-02	6.52	2.360E-04			1.0
2	1	E	3	2	E	1003.76340	-1	.639347E-02	0.7	0.6503E-02	-1.71	3.819E-04			1.0
39	1	E	39	2	E	1003.88229	-4	.246182E-02	0.9	0.2386E-02	3.08	3.026E-04			1.0
21	4	E	20	5	E	1003.98491	7	.332894E-02	0.7	0.3160E-02	5.08	4.171E-04			1.0
5	2	A_2	4	3	A_1	1003.99824	0	.208604E-02	1.6	0.1023E-02	1.94	4.529E-04			1.0
5	2	A_1	4	3	A_2	1003.99824	0			0.1023E-02					
38	1	E	38	2	E	1004.01026	-2	.283291E-02	0.7	0.2771E-02	2.20	3.217E-04			1.0
38	6	E	37	7	E	1004.01776	-18	.390432E-03	3.3	0.4112E-03	-5.33	5.402E-04			1.0
47	7	E	46	8	E	1004.05229	-29			0.6873E-04					

(cont.)

J	K_a	Γ	J'	K'_a	Γ'	$\nu^{a)}$	$\delta_\nu{}^{b)}$	$S_\nu^{\rm exp}(294.45)^{c)}$	$\Delta_S{}^{d)}$	$S_\nu^{\rm calc}(294.45)^{e)}$	$\delta_S{}^{f)}$	$\Gamma_0{}^{g)}$	$\Delta_2{}^{g)}$	$\nu_{\rm VC}{}^{g)}$	$R^{h)}$
	1			2		3	4	5	6	7	8	9	10	11	12
13	3	E	12	4	E	1004.07492	3	.379958E-02	0.3	0.3722E-02	2.05	3.624E-04	-.702E-01	-.474E+00	1.5
24	2	E	25	1	E	1004.11890	22			0.5884E-02					
37	1	E	37	2	E	1004.13489	-1	.336577E-02	1.0	0.3200E-02	4.92	3.459E-04			1.0
36	1	E	36	2	E	1004.25620	-1	.385286E-02	0.4	0.3678E-02	4.54	3.184E-04			1.0
9	0	E	10	1	E	1004.29692	1	.904039E-02	0.1	0.9368E-02	-3.62	3.226E-04	-.127E+00	-.430E+00	1.9
31	3	E	32	2	E	1004.35527	-5	.277984E-02	0.7	0.2875E-02	-3.43	3.789E-04			1.0
35	1	E	35	2	E	1004.37420	0	.428993E-02	0.2	0.4204E-02	2.00	2.726E-04			1.0
34	1	E	34	2	E	1004.48885	-4	.474761E-02	0.5	0.4781E-02	-.70	3.044E-04			1.0
30	5	A_2	29	6	A_1	1004.50016	-2	.283323E-02	0.9	0.1400E-02	1.20	3.208E-04			1.0
30	5	A_1	29	6	A_2	1004.50016	-2			0.1400E-02					
38	4	A_2	39	3	A_1	1004.50891	-2	.228693E-02	1.6	0.1057E-02	7.60	2.964E-04			1.0
38	4	A_1	39	3	A_2	1004.50891	-2		1.6	0.1057E-02		2.964E-04			1.0
45	5	E	46	4	E	1004.58295	-26			0.2957E-03					
33	1	E	33	2	E	1004.60025	-1	.568283E-02	0.2	0.5407E-02	4.85	3.909E-04	-.182E+00	.213E+00	1.4
1	1	E	2	2	E	1004.65662	-1	.605004E-02	0.7	0.5938E-02	1.86	3.833E-04			1.0
16	1	A_1	17	0	A_2	1004.66754	1	.179278E-01	0.3	0.1864E-01	-3.95	4.215E-04			1.0
32	1	E	32	2	E	1004.70833	0	.592968E-02	0.3	0.6083E-02	-2.59	3.245E-04			1.0
48	7	E	47	8	E	1004.76249	-15			0.5759E-04					
22	4	E	21	5	E	1004.79575	2	.310747E-02	0.9	0.3096E-02	.37	4.130E-04			1.0
31	1	E	31	2	E	1004.81307	-2	.692630E-02	0.5	0.6806E-02	1.73	3.430E-04			1.0
6	2	A_2	5	3	A_1	1004.86512	1	.345761E-02	0.9	0.1669E-02	3.44	4.964E-04			1.0
6	2	A_1	5	3	A_2	1004.86512	1			0.1669E-02					
14	3	E	13	4	E	1004.91449	16	.118033E-01	0.3	0.3993E-02	2.01	3.451E-04	-.171E+00	-.151E+00	1.4
30	1	E	30	2	E	1004.91449	-7			0.7574E-02					
29	1	E	29	2	E	1005.01272	0	.864150E-02	0.3	0.8381E-02	3.02	2.975E-04		-.361E+00	1.2
23	2	E	24	1	E	1005.07888	-1	.583298E-02	1.0	0.6260E-02	-7.32	4.500E-04			1.0
28	1	E	28	2	E	1005.10759	0	.938409E-02	0.5	0.9222E-02	1.73	3.204E-04			1.0
27	1	E	27	2	E	1005.19906	-11	.110323E-01	0.4	0.1009E-01	8.55	4.428E-04			1.0
8	0	E	9	1	E	1005.21234	-2	.879452E-02	0.6	0.8907E-02	-1.28	4.208E-04			1.0
26	1	E	26	2	E	1005.28745	-1	.110853E-01	1.0	0.1097E-01	1.01	3.248E-04			1.0
30	3	E	31	2	E	1005.33527	-6	.308492E-02	1.1	0.3176E-02	-2.96	2.442E-04			1.0
25	1	E	25	2	E	1005.37244	-1	.120283E-01	0.2	0.1186E-01	1.36	3.398E-04			1.0
24	1	E	24	2	E	1005.45415	-1	.132305E-01	0.3	0.1275E-01	3.62	3.691E-04			1.0
49	7	E	48	8	E	1005.46863	-1			0.4800E-04					
37	4	A_1	38	3	A_2	1005.50780	-19	.275159E-02	1.0	0.1209E-02	.66	4.566E-04			1.0
37	4	A_2	38	3	A_1	1005.50780	-19			0.1209E-02					
40	6	E	39	7	E	1005.50780	96			0.3153E-03					
23	1	E	23	2	E	1005.53261	2	.140906E-01	0.3	0.1362E-01	3.35	4.005E-04			1.0
58	7	A_1	59	6	A_2	1005.55966	-6			0.1321E-04					
58	7	A_2	59	6	A_1	1005.55966	-6			0.1321E-04					
23	4	E	22	5	E	1005.60303	2			0.3007E-02					
22	1	E	22	2	E	1005.60777	3	.142739E-01	0.3	0.1445E-01	-1.26	4.075E-04			1.0
15	1	A_2	16	0	A_1	1005.61211	-3	.182691E-01	0.3	0.1879E-01	-2.84	4.075E-04			1.0
51	6	E	52	5	E	1005.61726	54			0.7752E-04					
21	1	E	21	2	E	1005.67981	21			0.1524E-01					
7	2	A_2	6	3	A_1	1005.72865	2	.477271E-02	2.9	0.2325E-02	2.56	5.008E-04			1.0
7	2	A_1	6	3	A_2	1005.72865	2			0.2325E-02					
20	1	E	20	2	E	1005.74829	10	.188446E-01	0.6	0.1596E-01	-7.04	7.217E-04	.145E+01	.373E+00	2.5
19	1	E	19	2	E	1005.81348	-1	.163758E-01	0.4	0.1660E-01	-1.39	4.127E-04			1.0
18	1	E	18	2	E	1005.87549	-4			0.1715E-01					
17	1	E	17	2	E	1005.93428	0	.172036E-01	0.4	0.1757E-01	-2.15	4.053E-04			1.0
16	1	E	16	2	E	1005.98975	-2	.176566E-01	0.1	0.1787E-01	-1.20	4.088E-04			1.0
22	2	E	23	1	E	1006.03617	-4	.650149E-02	1.2	0.6616E-02	-1.76	4.592E-04			1.0
15	1	E	15	2	E	1006.04201	2	.186056E-01	0.6	0.1802E-01	3.15	4.676E-04			1.0
32	5	A_2	31	6	A_1	1006.05124	-7	.234784E-02	2.4	0.1175E-02	-.12	3.393E-04			1.0
32	5	A_1	31	6	A_2	1006.05124	-7			0.1175E-02					
14	1	E	14	2	E	1006.09093	0	.173904E-01	0.2	0.1801E-01	-3.57	3.553E-04	-.195E+00	-.420E+00	1.9
7	0	E	8	1	E	1006.12471	3	.839079E-02	0.5	0.8348E-02	.51	3.728E-04			1.0
13	1	E	13	2	E	1006.13662	1	.177713E-01	0.3	0.1783E-01	-.34	4.365E-04			1.0
50	7	E	49	8	E	1006.17015	-41			0.3980E-04					
12	1	E	12	2	E	1006.17901	-1	.170035E-01	0.1	0.1747E-01	-2.75	3.865E-04	-.711E-01	-.614E-01	1.3
11	1	E	11	2	E	1006.21817	1	.166706E-01	0.1	0.1692E-01	-1.52	3.791E-04	-.623E-01	-.142E+00	1.7
41	6	E	40	7	E	1006.24570	27			0.2738E-03					
10	1	E	10	2	E	1006.25404	0	.153652E-01	0.4	0.1619E-01	-5.34	2.955E-04		-.660E+00	1.5
9	1	E	9	2	E	1006.28665	0	.144863E-01	0.3	0.1525E-01	-5.30	3.181E-04	-.763E-01	-.499E+00	2.9
29	3	E	30	2	E	1006.31262	7	.350137E-02	0.9	0.3489E-02	.34	3.462E-04			1.0
8	1	E	8	2	E	1006.31602	2	.135787E-01	0.9	0.1413E-01	-4.07	3.462E-04			1.0
7	1	E	7	2	E	1006.34208	-1	.130896E-01	0.3	0.1282E-01	2.07	3.835E-04	-.168E+00	-.143E+00	1.5
6	1	E	6	2	E	1006.36493	2	.113255E-01	0.3	0.1132E-01	.02	3.576E-04	-.980E-01	-.112E+00	1.3
5	1	E	5	2	E	1006.38451	3	.963203E-02	0.4	0.9646E-02	-.15	3.867E-04			1.0
4	1	E	4	2	E	1006.40081	3	.782340E-02	1.1	0.7784E-02	.51	3.588E-04			1.0
24	4	E	23	5	E	1006.40669	3			0.2895E-02					
3	1	E	3	2	E	1006.41381	-1	.576134E-02	1.1	0.5707E-02	.94	4.719E-04			1.0
2	1	E	2	2	E	1006.42360	0	.334752E-02	2.2	0.3306E-02	1.26	5.368E-04			1.0
36	4	A_2	37	3	A_1	1006.50429	-7	.264700E-02	0.7	0.1376E-02	-4.00	2.978E-04			1.0
36	4	A_1	37	3	A_2	1006.50429	-7			0.1376E-02					
14	1	A_1	15	0	A_2	1006.55326	1	.183252E-01	0.2	0.1878E-01	-2.47	3.806E-04	-.420E-01	-.418E+00	1.3
16	3	E	15	4	E	1006.58272	-3	.433695E-02	0.9	0.4368E-02	-.73	3.545E-04			1.0
8	2	A_2	7	3	A_1	1006.58879	1	.595517E-02	0.6	0.2962E-02	.52	3.732E-04			1.0
8	2	A_1	7	3	A_2	1006.58879	2			0.2962E-02					
43	5	E	44	4	E	1006.61563	24			0.4120E-03					
50	6	E	51	5	E	1006.64921	-3			0.9431E-04					

(cont.)

J	K_a	Γ	J'	K_a'	Γ'	$\nu^{a)}$	$\delta_\nu{}^{b)}$	$S_\nu^{\rm exp}(294.45)^{c)}$	$\Delta_S{}^{d)}$	$S_\nu^{\rm calc}(294.45)^{e)}$	$\delta_S{}^{f)}$	$\Gamma_0{}^{g)}$	$\Delta_2{}^{g)}$	$\nu_{\rm VC}{}^{g)}$	$R^{h)}$
1			2			3	4	5	6	7	8	9	10	11	12
63	0	E	63	1	E	1006.65800	-14			0.1485E-04					
33	5	A_2	32	6	A_1	1006.82114	-8	.200825E-02	0.8	0.1067E-02	-6.24	3.496E-04			1.0
33	5	A_1	32	6	A_2	1006.82114	-8			0.1067E-02					
51	7	E	50	8	E	1006.86844	5			0.3284E-04					
42	6	E	41	7	E	1006.97981	-28			0.2365E-03					
42	6	E	41	7	E	1006.98013	4			0.2365E-03					
21	2	E	22	1	E	1006.99062	-1	.675240E-02	0.8	0.6946E-02	-2.87	2.308E-04		-.608E+00	1.2
6	0	E	7	1	E	1007.03386	0	.772322E-02	0.3	0.7694E-02	.38	3.805E-04			1.0
61	0	E	61	1	E	1007.07604	41			0.2540E-04					
25	4	E	24	5	E	1007.20665	-3	.283580E-02	7.6	0.2766E-02	2.46	2.428E-04			1.0
60	0	E	60	1	E	1007.27894	-19			0.3297E-04					
28	3	E	29	2	E	1007.28694	-4	.367738E-02	0.9	0.3811E-02	-3.64	3.508E-04	-.220E+00		1.2
17	3	E	16	4	E	1007.41172	2			0.4474E-02					
9	2	A_2	8	3	A_1	1007.44556	2	.714292E-02	0.4	0.3562E-02	.26	3.918E-04			1.0
9	2	A_1	8	3	A_2	1007.44556	2			0.3562E-02					
59	0	E	59	1	E	1007.47920	7			0.4259E-04					
13	1	A_2	14	0	A_1	1007.49085	1	.181178E-01	0.2	0.1859E-01	-2.62	4.290E-04			1.0
35	4	A_2	36	3	A_1	1007.49789	-14	.313175E-02	1.2	0.1559E-02	.45	3.715E-04			1.0
35	4	A_1	36	3	A_2	1007.49789	-14			0.1559E-02					
52	7	E	51	8	E	1007.56212	1			0.2696E-04					
34	5	A_2	33	6	A_1	1007.58727	-6	.189848E-02	5.6	0.9624E-03	-1.38	3.149E-04			1.0
34	5	A_1	33	6	A_2	1007.58727	-6			0.9624E-03					
42	5	E	43	4	E	1007.62729	-29			0.4828E-03					
58	0	E	58	1	E	1007.67568	4			0.5475E-04					
49	6	E	50	5	E	1007.67956	30			0.1142E-03					
43	6	E	42	7	E	1007.71037	-43			0.2031E-03					
57	0	E	57	1	E	1007.86871	3			0.7003E-04					
5	0	E	6	1	E	1007.94033	45			0.6949E-02					
26	4	E	25	5	E	1008.00299	-4	.261490E-02	0.5	0.2623E-02	-.29	3.842E-04			1.0
56	0	E	56	1	E	1008.05806	-20			0.8915E-04					
18	3	E	17	4	E	1008.23717	5	.449044E-02	0.7	0.4528E-02	-.83	4.282E-04			1.0
55	0	E	55	1	E	1008.24389	-47			0.1129E-03					
27	3	E	28	2	E	1008.25852	-6	.409763E-02	0.4	0.4138E-02	-.99	4.049E-04	-.117E+00		1.1
10	2	A_2	9	3	A_1	1008.29891	0	.843473E-02	0.5	0.4113E-02	2.48	3.883E-04	-.140E+00		1.0
10	2	A_1	9	3	A_2	1008.29891	1			0.4113E-02					
35	5	A_2	34	6	A_1	1008.34956	-7	.169651E-02	13.4	0.8630E-03	-1.74	5.786E-04			1.0
35	5	A_1	34	6	A_2	1008.34956	-7			0.8630E-03					
12	1	A_1	13	0	A_2	1008.42490	0	.178319E-01	0.2	0.1822E-01	-2.20	4.009E-04	-.133E+00	-.252E+00	1.5
44	6	E	43	7	E	1008.43716	-38			0.1735E-03					
34	4	A_2	35	3	A_1	1008.48893	-5	.337862E-02	0.4	0.1756E-02	-3.95	3.084E-04	-.223E+00	-.390E+00	1.5
34	4	A_1	35	3	A_2	1008.48893	-5			0.1756E-02					
53	0	E	53	1	E	1008.60613	-9			0.1786E-03					
41	5	E	42	4	E	1008.63724	9			0.5629E-03					
48	6	E	49	5	E	1008.70657	-18			0.1376E-03					
52	0	E	52	1	E	1008.78208	9			0.2229E-03					
27	4	E	26	5	E	1008.79569	-2	.262888E-02	0.6	0.2468E-02	6.10	3.611E-04			1.0
4	0	E	5	1	E	1008.84275	2	.593576E-02	0.7	0.6122E-02	-3.14	2.688E-04	-.218E+00	-.391E+00	1.2
19	2	E	20	1	E	1008.89073	0	.749292E-02	0.4	0.7495E-02	-.03	4.643E-04			1.0
51	0	E	51	1	E	1008.95457	25			0.2769E-03					
19	3	E	18	4	E	1009.05900	0	.448763E-02	0.8	0.4532E-02	-.99	4.300E-04	-.220E+00		1.1
3	1	E	2	2	E	1009.07401	0			0.6635E-03					
36	5	A_2	35	6	A_1	1009.10804	-7	.146122E-02	0.5	0.7693E-03	-5.30	1.984E-04	-.316E+00	-.303E+00	2.1
36	5	A_1	35	6	A_2	1009.10804	-7			0.7693E-03					
50	0	E	50	1	E	1009.12320	-2			0.3422E-03					
11	2	A_2	10	3	A_1	1009.14860	-25			0.4604E-02					
11	2	A_1	10	3	A_2	1009.14860	-26			0.4604E-02					
26	3	E	27	2	E	1009.22734	-2	.431570E-02	0.8	0.4467E-02	-3.50	3.872E-04	-.161E+00		1.1
49	0	E	49	1	E	1009.28867	-3			0.4209E-03					
11	1	A_2	12	0	A_1	1009.35541	0	.173563E-01	0.1	0.1766E-01	-1.77	3.513E-04	-.911E-01	-.371E+00	1.2
48	0	E	48	1	E	1009.45063	-14			0.5150E-03					
33	4	A_2	34	3	A_1	1009.47721	2	.399029E-02	0.5	0.1968E-02	1.39	3.328E-04	-.241E+00		1.2
33	4	A_1	34	3	A_2	1009.47721	2			0.1968E-02					
28	4	E	27	5	E	1009.58469	-1	.238830E-02	0.4	0.2307E-02	3.40	3.912E-04			1.0
47	0	E	47	1	E	1009.60941	-2			0.6271E-03					
40	5	E	41	4	E	1009.64395	-14	.715478E-03	2.0	0.6531E-03	8.72	2.555E-04			1.0
47	6	E	48	5	E	1009.73173	2			0.1650E-03					
3	0	E	4	1	E	1009.74241	2	.534844E-02	0.5	0.5220E-02	2.40	3.912E-04			1.0
46	0	E	46	1	E	1009.76467	-1	.681859E-03	1.3	0.7598E-03	-11.43	3.447E-04	.197E+00		1.1
18	2	E	19	1	E	1009.83638	0	.762136E-02	0.4	0.7699E-02	-1.02	4.255E-04	-.964E-01		1.1
37	5	A_1	36	6	A_2	1009.86268	-7			0.6819E-03					
37	5	A_2	36	6	A_1	1009.86268	-7			0.6819E-03					
20	3	E	19	4	E	1009.87734	1	.448945E-02	0.4	0.4492E-02	-2.82	3.953E-04	.887E-01	-.356E+00	1.1
46	6	E	45	7	E	1009.87734	-172			0.1247E-03					
45	0	E	45	1	E	1009.91656	2	.999236E-03	1.4	0.9159E-03	8.34	2.899E-04	-.282E+00		1.1
4	1	E	3	2	E	1009.94769	6	.145417E-02	0.9	0.1475E-02	-1.42	4.347E-04			1.0
12	2	A_2	11	3	A_1	1009.99538	0	.104046E-01	0.3	0.5031E-02	3.29	4.214E-04			1.0
12	2	A_1	11	3	A_2	1009.99538	1			0.5031E-02					
44	0	E	44	1	E	1010.06488	-13	.121343E-02	2.4	0.1099E-02	9.46	2.635E-04			1.0
25	3	E	26	2	E	1010.19326	-3	.467079E-02	0.5	0.4791E-02	-2.58	3.491E-04			1.0
43	0	E	43	1	E	1010.21006	-4	.145604E-02	0.4	0.1311E-02	9.96	2.927E-04			1.0
10	1	A_1	11	0	A_2	1010.28238	3	.169280E-01	0.3	0.1691E-01	.11	5.116E-04			1.0
42	0	E	42	1	E	1010.35181	1			0.1557E-02					

(cont.)

J	K_a	Γ	J'	K'_a	Γ'	$\nu^{a)}$	$\delta_\nu{}^{b)}$	$S_\nu^{\rm exp}(294.45)^{c)}$	$\Delta_S{}^{d)}$	$S_\nu^{\rm calc}(294.45)^{e)}$	$\delta_S{}^{f)}$	$\Gamma_0{}^{g)}$	$\Delta_2{}^{g)}$	$\nu_{\rm VC}{}^{g)}$	$R^{h)}$
	1			2		3	4	5	6	7	8	9	10	11	12
29	4	E	28	5	E	1010.36995	-3	.219279E-02	0.5	0.2142E-02	2.32	4.054E-04			1.0
32	4	A_2	33	3	A_1	1010.46262	-4	.431181E-02	0.4	0.2192E-02	-1.69	2.879E-04	-.353E+00	-.221E+00	1.7
32	4	A_1	33	3	A_2	1010.46262	-4			0.2192E-02					
41	0	E	41	1	E	1010.49016	3	.198431E-02	0.5	0.1839E-02	7.33	2.465E-04	-.341E+00		1.5
47	6	E	46	7	E	1010.59360	-20			0.1049E-03					
38	5	A_2	37	6	A_1	1010.61346	-7	.124875E-02	1.4	0.6009E-03	3.76	2.900E-04			1.0
38	5	A_1	37	6	A_2	1010.61346	-7			0.6009E-03					
40	0	E	40	1	E	1010.62475	-35			0.2161E-02					
2	0	E	3	1	E	1010.63890	4	.435566E-02	0.4	0.4255E-02	2.32	4.296E-04			1.0
39	5	E	40	4	E	1010.64835	-3	.813530E-03	2.3	0.7538E-03	7.34	1.915E-04			1.0
21	3	E	20	4	E	1010.69208	0	.443902E-02	0.5	0.4410E-02	.65	4.016E-04			1.0
39	0	E	39	1	E	1010.75668	-2	.279646E-02	3.8	0.2527E-02	9.64	1.087E-04			1.0
17	2	E	18	1	E	1010.77903	-3	.766731E-02	0.4	0.7845E-02	-2.31	4.336E-04			1.0
5	1	E	4	2	E	1010.81799	5	.231768E-02	0.5	0.2321E-02	-.15	2.987E-04	-.224E+00	-.422E+00	1.3
13	2	A_2	12	3	A_1	1010.83845	1	.108382E-01	0.2	0.5389E-02	.56	3.525E-04	-.150E+00	-.449E+00	1.7
13	2	A_1	12	3	A_2	1010.83845	-1			0.5389E-02					
38	0	E	38	1	E	1010.88494	0	.320599E-02	3.9	0.2939E-02	8.32	2.815E-04			1.0
37	0	E	37	1	E	1011.00984	0	.366556E-02	0.4	0.3401E-02	7.22	3.206E-04			1.0
36	0	E	36	1	E	1011.13137	-1	.422301E-02	0.6	0.3915E-02	7.30	2.999E-04	.136E+00		1.1
30	4	E	29	5	E	1011.15162	8	.206013E-02	1.0	0.1975E-02	4.11	2.808E-04			1.0
24	3	E	25	2	E	1011.15634	-2	.499552E-02	0.4	0.5107E-02	-2.23	3.976E-04			1.0
9	1	A_2	10	0	A_1	1011.20573	1	.157995E-01	0.2	0.1596E-01	-.99	4.175E-04			1.0
35	0	E	35	1	E	1011.24956	-3	.476969E-02	0.3	0.4482E-02	6.03	2.332E-04	-.750E-01	-.315E+00	1.2
48	6	E	47	7	E	1011.30392	-59			0.8773E-04					
39	5	A_2	38	6	A_1	1011.36055	12	.109227E-02	0.4	0.5266E-03	3.57	2.970E-04			1.0
39	5	A_1	38	6	A_2	1011.36055	12			0.5266E-03					
34	0	E	34	1	E	1011.36443	-2	.549711E-02	0.4	0.5104E-02	7.14	2.970E-04			1.0
31	4	A_2	32	3	A_1	1011.44531	-6	.464102E-02	0.6	0.2429E-02	-4.69	3.126E-04	-.159E+00		1.1
31	4	A_1	32	3	A_2	1011.44531	-6			0.2429E-02					
33	0	E	33	1	E	1011.47598	0	.614718E-02	0.2	0.5782E-02	5.94	2.449E-04	-.129E+00	-.317E+00	2.0
22	3	E	21	4	E	1011.50323	0	.426391E-02	0.6	0.4292E-02	-.67	4.391E-04			1.0
1	0	E	2	1	E	1011.53211	-1	.336528E-02	0.5	0.3237E-02	3.82	4.895E-04			1.0
32	0	E	32	1	E	1011.58418	-1	.703075E-02	0.4	0.6514E-02	7.35	3.079E-04			1.0
38	5	E	39	4	E	1011.64991	-9	.916940E-03	2.7	0.8656E-03	5.60	2.000E-04			1.0
14	2	A_2	13	3	A_1	1011.67808	0	.117754E-01	0.3	0.5676E-02	3.60	3.914E-04			1.0
14	2	A_1	13	3	A_2	1011.67808	2			0.5676E-02					
6	1	E	5	2	E	1011.68498	7	.323333E-02	0.5	0.3157E-02	2.37	3.697E-04			1.0
31	0	E	31	1	E	1011.68904	-2	.781218E-02	0.5	0.7299E-02	6.57	3.697E-04			1.0
16	2	E	17	1	E	1011.71876	-1	.789033E-02	0.3	0.7925E-02	-.44	4.412E-04	-.549E-01	-.266E+00	1.1
45	6	E	46	5	E	1011.77417	24			0.2339E-03					
30	0	E	30	1	E	1011.79061	-1	.854407E-02	0.5	0.8133E-02	4.81	3.123E-04			1.0
29	0	E	29	1	E	1011.88885	-1	.938728E-02	0.5	0.9012E-02	4.00	3.487E-04			1.0
31	4	E	30	5	E	1011.92937	1	.180529E-02	1.4	0.1810E-02	-.28	3.587E-04			1.0
28	0	E	28	1	E	1011.98378	-1	.103528E-01	0.3	0.9928E-02	4.10	3.298E-04			1.0
49	6	E	48	7	E	1012.01167	50			0.7303E-04					
27	0	E	27	1	E	1012.07540	-2	.113670E-01	0.3	0.1088E-01	4.32	3.385E-04	-.460E-01		1.0
23	3	E	24	2	E	1012.11658	2	.543566E-02	2.1	0.5407E-02	.52	2.513E-04			1.0
8	1	A_1	9	0	A_2	1012.12549	-1	.148480E-01	1.0	0.1481E-01	.26	5.645E-04			1.0
26	0	E	26	1	E	1012.16373	0	.121391E-01	0.3	0.1184E-01	2.43	3.368E-04			1.0
25	0	E	25	1	E	1012.24876	1	.132844E-01	0.3	0.1282E-01	3.49	3.874E-04			1.0
23	3	E	22	4	E	1012.31077	-2	.412168E-02	0.7	0.4144E-02	-.53	4.361E-04			1.0
24	0	E	24	1	E	1012.33046	-1	.140661E-01	0.3	0.1380E-01	1.92	3.575E-04			1.0
23	0	E	23	1	E	1012.40886	-3	.155432E-01	0.4	0.1475E-01	5.09	4.009E-04			1.0
22	0	E	22	1	E	1012.48402	0	.156463E-01	0.3	0.1568E-01	-.18	3.981E-04			1.0
15	2	A_2	14	3	A_1	1012.51422	2	.120447E-01	0.3	0.5891E-02	2.18	4.247E-04			1.0
15	2	A_1	14	3	A_2	1012.51422	0			0.5891E-02					
7	1	E	6	2	E	1012.54857	4	.409399E-02	1.8	0.3957E-02	3.35	3.694E-04			1.0
21	0	E	21	1	E	1012.55592	5	.181626E-01	0.4	0.1655E-01	8.89	4.316E-04			1.0
20	0	E	20	1	E	1012.62436	-6			0.1735E-01					
37	5	E	38	4	E	1012.64895	0	.100346E-02	3.7	0.9888E-03	1.46	2.232E-04			1.0
15	2	E	16	1	E	1012.65548	-1	.769755E-02	0.4	0.7933E-02	-3.06	4.206E-04			1.0
19	0	E	19	1	E	1012.68971	1	.183362E-01	0.2	0.1807E-01	1.45	4.151E-04			1.0
18	0	E	18	1	E	1012.75169	0	.185118E-01	0.2	0.1869E-01	-.94	4.063E-04			1.0
44	6	E	45	5	E	1012.79120	2			0.2765E-03					
17	0	E	17	1	E	1012.81040	-1	.189665E-01	0.3	0.1918E-01	-1.11	4.040E-04			1.0
41	5	A_2	40	6	A_1	1012.84258	4	.828447E-03	0.8	0.3978E-03	3.96	3.595E-04			1.0
41	5	A_1	40	6	A_2	1012.84258	4			0.3978E-03					
51	7	A_2	52	6	A_1	1012.85586	34			0.5900E-04					
51	7	A_1	52	6	A_2	1012.85586	34			0.5900E-04					
16	0	E	16	1	E	1012.86580	-5			0.1953E-01					
15	0	E	15	1	E	1012.91802	0	.192935E-01	0.4	0.1972E-01	-2.22	4.036E-04			1.0
14	0	E	14	1	E	1012.96692	0	.193372E-01	0.2	0.1975E-01	-2.12	3.423E-04	-.959E-01	-.523E+00	1.7
13	0	E	13	1	E	1013.01256	1	.190503E-01	0.2	0.1959E-01	-2.82	3.742E-04	-.147E+00	-.179E+00	1.7
7	1	A_2	8	0	A_1	1013.04167	0	.133670E-01	0.2	0.1347E-01	-.79	3.899E-04			1.0
12	0	E	12	1	E	1013.05491	-1	.192027E-01	0.2	0.1924E-01	-.18	4.049E-04			1.0
22	3	E	23	2	E	1013.07384	-3	.556807E-02	0.6	0.5687E-02	-2.13	4.343E-04			1.0
11	0	E	11	1	E	1013.09403	1	.182774E-01	0.2	0.1869E-01	-2.24	3.672E-04	-.227E+00	-.281E+00	1.7
24	3	E	23	4	E	1013.11472	0	.393990E-02	1.5	0.3969E-02	-.74	3.611E-04			1.0
10	0	E	10	1	E	1013.12986	1	.173616E-01	0.4	0.1793E-01	-3.29	3.705E-04			1.0
9	0	E	9	1	E	1013.16243	1	.166659E-01	0.1	0.1698E-01	-1.87	3.615E-04	-.957E-01	-.149E+00	1.7
8	0	E	8	1	E	1013.19175	2	.158588E-01	0.3	0.1582E-01	.23	3.925E-04	-.188E+00	.832E-01	1.2
7	0	E	7	1	E	1013.21778	0	.151619E-01	0.3	0.1448E-01	4.53	4.101E-04	-.470E+00		2.4

(cont.)

J	K_a	Γ	J'	K'_a	Γ'	$\nu^{a)}$	$\delta_\nu{}^{b)}$	$S_\nu^{\rm exp}(294.45)^{c)}$	$\Delta_S{}^{d)}$	$S_\nu^{\rm calc}(294.45)^{e)}$	$\delta_S{}^{f)}$	$\Gamma_0{}^{g)}$	$\Delta_2{}^{g)}$	$\nu_{\rm VC}{}^{g)}$	$R^{h)}$
	1			2		3	4	5	6	7	8	9	10	11	12
6	0	E	6	1	E	1013.24059	1	.126589E-01	0.2	0.1295E-01	-2.29	3.193E-04	-.153E+00	-.290E+00	2.1
5	0	E	5	1	E	1013.26013	2	.110490E-01	0.2	0.1126E-01	-1.90	3.007E-04	-.157E+00	-.464E+00	2.5
4	0	E	4	1	E	1013.27643	4	.937230E-02	0.5	0.9423E-02	-.54	4.751E-04			1.0
3	0	E	3	1	E	1013.28940	-1	.716085E-02	0.5	0.7463E-02	-4.22	4.034E-04			1.0
2	0	E	2	1	E	1013.29920	3	.541553E-02	0.7	0.5403E-02	.22	4.248E-04			1.0
1	0	E	1	1	E	1013.30571	2	.304315E-02	1.2	0.3272E-02	-7.51	7.584E-04			1.0
16	2	A_2	15	3	A_1	1013.34686	-1	.123541E-01	0.3	0.6036E-02	2.28	3.799E-04			1.0
16	2	A_1	15	3	A_2	1013.34686	1			0.6036E-02					
29	4	A_2	30	3	A_1	1013.40242	-3	.553475E-02	0.5	0.2932E-02	-5.95	3.083E-04			1.0
29	4	A_1	30	3	A_2	1013.40242	-3			0.2932E-02					
8	1	E	7	2	E	1013.40880	2	.483342E-02	0.6	0.4705E-02	2.66	4.197E-04			1.0
33	4	E	32	5	E	1013.47369	-3	.145879E-02	1.0	0.1492E-02	-2.30	3.126E-04			1.0
42	5	A_2	41	6	A_1	1013.57774	2			0.3430E-03					
42	5	A_1	41	6	A_2	1013.57774	2			0.3430E-03					
14	2	E	15	1	E	1013.58919	-1	.756483E-02	0.3	0.7862E-02	-3.93	4.256E-04			1.0
36	5	E	37	4	E	1013.64517	-3			0.1124E-02					
43	6	E	44	5	E	1013.80575	-9			0.3252E-03					
50	7	A_2	51	6	A_1	1013.88750	-39			0.7170E-04					
50	7	A_1	51	6	A_2	1013.88750	-39			0.7170E-04					
57	8	E	58	7	E	1013.89447	-20			0.1212E-04					
25	3	E	24	4	E	1013.91501	-1			0.3773E-02					
6	1	A_1	7	0	A_2	1013.95424	2	.115991E-01	0.4	0.1196E-01	-3.08	3.558E-04			1.0
21	3	E	22	2	E	1014.02824	-3	.571983E-02	0.4	0.5938E-02	-3.81	3.077E-04		-.547E+00	1.4
52	6	E	51	7	E	1014.10669	2			0.4088E-04					
17	2	A_2	16	3	A_1	1014.17599	0	.120683E-01	0.6	0.6114E-02	-1.33	3.727E-04	-.717E-01		1.0
17	2	A_1	16	3	A_2	1014.17599	-3			0.6114E-02					
34	4	E	33	5	E	1014.24021	-2			0.1343E-02					
9	1	E	8	2	E	1014.26568	2	.549613E-02	0.4	0.5388E-02	1.96	3.913E-04			1.0
43	5	A_2	42	6	A_1	1014.30879	-16	.633795E-03	1.0	0.2941E-03	7.18	2.337E-04			1.0
43	5	A_1	42	6	A_2	1014.30879	-16			0.2941E-03					
28	4	A_2	29	3	A_1	1014.37676	-4	.603108E-02	0.9	0.3193E-02	-5.88	2.777E-04			1.0
28	4	A_1	29	3	A_2	1014.37676	-4			0.3193E-02					
13	2	E	14	1	E	1014.51985	-4	.784649E-02	0.5	0.7708E-02	1.76	4.182E-04			1.0
35	5	E	36	4	E	1014.63863	-12	.125067E-02	0.6	0.1270E-02	-1.54	2.463E-04	.423E-01	-.278E+00	1.1
26	3	E	25	4	E	1014.71165	-1	.352746E-02	0.9	0.3562E-02	-.98	5.080E-04			1.0
62	1	A_2	62	0	A_1	1014.80037	3			0.7761E-04					
42	6	E	43	5	E	1014.81758	-30			0.3805E-03					
5	1	A_2	6	0	A_1	1014.86315	1	.104711E-01	0.3	0.1027E-01	1.90	4.047E-04	.598E-01	-.243E+00	1.1
49	7	A_2	50	6	A_1	1014.91742	-31			0.8673E-04					
49	7	A_1	50	6	A_2	1014.91742	-31			0.8673E-04					
20	3	E	21	2	E	1014.97972	-3	.590533E-02	0.3	0.6154E-02	-4.22	3.552E-04	.936E-01	-.326E+00	1.3
18	2	A_2	17	3	A_1	1015.00172	8	.141026E-01	0.2	0.6128E-02	4.57	4.207E-04		-.650E+00	1.7
18	2	A_1	17	3	A_2	1015.00172	11			0.6128E-02					
44	5	A_1	43	6	A_2	1015.03610	-12			0.2509E-03					
44	5	A_2	43	6	A_1	1015.03610	-12			0.2509E-03					
2	0	E	1	1	E	1015.07259	-15	.106842E-02	2.0	0.1093E-02	-2.30	6.534E-04			1.0
10	1	E	9	2	E	1015.11914	-1	.608493E-02	0.4	0.5999E-02	1.42	2.925E-04		-.724E+00	1.6
60	1	A_2	60	0	A_1	1015.14520	-14			0.1257E-03					
59	1	A_1	59	0	A_2	1015.31350	-2			0.1589E-03					
27	4	A_2	28	3	A_1	1015.34827	-5	.654518E-02	0.4	0.3456E-02	-5.59	2.788E-04	-.164E+00	-.356E+00	1.2
27	4	A_1	28	3	A_2	1015.34827	-5			0.3456E-02					
12	2	E	13	1	E	1015.44755	1	.740535E-02	0.6	0.7467E-02	-.83	4.265E-04			1.0
27	3	E	26	4	E	1015.50462	-1	.336875E-02	0.7	0.3339E-02	.88	2.326E-04		-.604E+00	1.3
34	5	E	35	4	E	1015.62946	-12	.141695E-02	1.7	0.1428E-02	-.75	1.854E-04			1.0
57	1	A_1	57	0	A_2	1015.64115	-6			0.2505E-03					
36	4	E	35	5	E	1015.76186	4			0.1069E-02					
4	1	A_1	5	0	A_2	1015.76843	2	.835856E-02	0.5	0.8437E-02	-.94	3.709E-04			1.0
56	1	A_2	56	0	A_1	1015.80100	25			0.3125E-03					
19	2	A_2	18	3	A_1	1015.82372	3	.119445E-01	0.4	0.6083E-02	-1.86	4.834E-04	.903E-01	.251E+00	1.1
19	2	A_1	18	3	A_2	1015.82372	-1			0.6083E-02					
19	3	E	20	2	E	1015.92828	-2	.628814E-02	0.7	0.6329E-02	-.65	4.668E-04	-.212E+00		1.2
3	0	E	2	1	E	1015.94981	9	.224231E-02	1.6	0.2169E-02	3.27	5.674E-04			1.0
55	1	A_1	55	0	A_2	1015.95732	-9			0.3881E-03					
11	1	E	10	2	E	1015.96923	1	.668577E-02	0.3	0.6529E-02	2.34	3.798E-04	-.213E+00		1.2
54	1	A_2	54	0	A_1	1016.11121	0			0.4799E-03					
55	6	E	54	7	E	1016.16606	98			0.2188E-04					
53	1	A_1	53	0	A_2	1016.26211	-5			0.5907E-03					
28	3	E	27	4	E	1016.29394	2	.313782E-02	0.4	0.3109E-02	.90	3.058E-04			1.0
26	4	A_2	27	3	A_1	1016.31699	-2	.735441E-02	0.5	0.3717E-02	-1.07	4.047E-04	-.321E+00	-.113E+00	1.4
26	4	A_1	27	3	A_2	1016.31699	-2			0.3717E-02					
11	2	E	12	1	E	1016.37225	11			0.7135E-02					
52	1	A_2	52	0	A_1	1016.41020	-4			0.7238E-03					
46	5	A_2	45	6	A_1	1016.47866	-17			0.1798E-03					
46	5	A_1	45	6	A_2	1016.47866	-17			0.1798E-03					
37	4	E	36	5	E	1016.51707	20			0.9451E-03					
51	1	A_1	51	0	A_2	1016.55543	-3			0.8829E-03					
33	5	E	34	4	E	1016.61755	-12	.160930E-02	0.8	0.1596E-02	.82	3.558E-04			1.0
20	2	A_2	19	3	A_1	1016.64227	1	.117275E-01	0.3	0.5984E-02	-2.05	4.482E-04			1.0
20	2	A_1	19	3	A_2	1016.64227	5			0.5984E-02					
3	1	A_2	4	0	A_1	1016.67005	4	.644261E-02	0.3	0.6469E-02	-.41	3.179E-04	-.699E-01	-.434E+00	1.7
50	1	A_2	50	0	A_1	1016.69788	4			0.1072E-02					
12	1	E	11	2	E	1016.81588	1	.684343E-02	0.6	0.6976E-02	-1.93	4.993E-04			1.0

(cont.)

J	K_a	Γ	J'	K'_a	Γ'	$\nu^{a)}$	$\delta_\nu{}^{b)}$	$S_\nu^{\rm exp}(294.45)^{c)}$	$\Delta_S{}^{d)}$	$S_\nu^{\rm calc}(294.45)^{e)}$	$\delta_S{}^{f)}$	$\Gamma_0{}^{g)}$	$\Delta_2{}^{g)}$	$\nu_{\rm VC}{}^{g)}$	$R^{h)}$
	1			2		3	4	5	6	7	8	9	10	11	12
4	0	E	3	1	E	1016.82337	-3	.325914E-02	1.4	0.3214E-02	1.37	5.054E-04			1.0
40	6	E	41	5	E	1016.83408	-1			0.5132E-03					
49	1	A_1	49	0	A_2	1016.83736	-1			0.1296E-02					
18	3	E	19	2	E	1016.87391	1	.634270E-02	0.5	0.6455E-02	-1.77	3.649E-04	-.839E-01	-.297E+00	1.1
47	7	A_2	48	6	A_1	1016.96983	0			0.1251E-03					
47	7	A_1	48	6	A_2	1016.96983	0			0.1251E-03					
48	1	A_2	48	0	A_1	1016.97405	-1			0.1560E-02					
54	8	E	55	7	E	1017.02781	-45			0.2328E-04					
29	3	E	28	4	E	1017.07954	3	.289861E-02	0.5	0.2877E-02	.75	3.402E-04			1.0
47	1	A_1	47	0	A_2	1017.10789	-2			0.1868E-02					
47	5	A_1	46	6	A_2	1017.19389	-23			0.1510E-03					
47	5	A_2	46	6	A_1	1017.19389	-23			0.1510E-03					
46	1	A_2	46	0	A_1	1017.23888	-4	.224061E-02	0.8	0.2228E-02	.57	2.398E-04			1.0
38	4	E	37	5	E	1017.26807	0	.867094E-03	1.4	0.8313E-03	4.13	2.767E-04			1.0
25	4	A_1	26	3	A_2	1017.28276	-9			0.3972E-02					
25	4	A_2	26	3	A_1	1017.28276	-9			0.3972E-02					
10	2	E	11	1	E	1017.29372	5	.668299E-02	0.4	0.6712E-02	-.43	4.153E-04			1.0
45	1	A_1	45	0	A_2	1017.36709	-1	.278856E-02	0.9	0.2644E-02	5.18	1.626E-04	-.137E+00		1.1
21	2	A_2	20	3	A_1	1017.45721	3	.115435E-01	0.4	0.5837E-02	-1.13	3.520E-04	.106E+00	-.505E+00	1.3
21	2	A_1	20	3	A_2	1017.45721	-1			0.5837E-02					
44	1	A_2	44	0	A_1	1017.49240	-5	.323843E-02	0.5	0.3124E-02	3.55	1.504E-04		-.366E+00	1.2
2	1	A_1	3	0	A_2	1017.56797	3	.432565E-02	0.4	0.4391E-02	-1.50	4.075E-04	-.133E+00	-.279E+00	1.2
32	5	E	33	4	E	1017.60296	-6	.177193E-02	1.1	0.1774E-02	-.13	3.618E-04			1.0
43	1	A_1	43	0	A_2	1017.61493	-4	.364889E-02	0.6	0.3673E-02	-.66	2.497E-04			1.0
13	1	E	12	2	E	1017.65912	4	.744173E-02	0.2	0.7336E-02	1.42	3.900E-04	-.173E+00	-.150E+00	1.5
5	0	E	4	1	E	1017.69376	-1	.424007E-02	0.3	0.4216E-02	.58	4.018E-04	-.288E+00	-.403E+00	1.9
42	1	A_2	42	0	A_1	1017.73501	35			0.4299E-02					
17	3	E	18	2	E	1017.81655	2	.642514E-02	0.3	0.6525E-02	-1.56	3.635E-04	-.231E+00	-.410E+00	2.0
39	6	E	40	5	E	1017.83826	3	.622988E-03	4.0	0.5914E-03	5.08	3.956E-04			1.0
41	1	A_1	41	0	A_2	1017.85153	-1	.494786E-02	0.4	0.5007E-02	-1.20	2.657E-04			1.0
30	3	E	29	4	E	1017.86135	-3	.261580E-02	0.6	0.2645E-02	-1.12	4.393E-04			1.0
48	5	A_2	47	6	A_1	1017.90518	-21			0.1262E-03					
48	5	A_1	47	6	A_2	1017.90518	-21			0.1262E-03					
40	1	A_2	40	0	A_1	1017.96557	-3	.571614E-02	0.5	0.5805E-02	-1.55	2.017E-04	-.323E+00	-.306E+00	2.0
46	7	A_2	47	6	A_1	1017.99237	31			0.1491E-03					
46	7	A_1	47	6	A_2	1017.99237	31			0.1491E-03					
39	4	E	38	5	E	1018.01530	-9	.699450E-03	1.8	0.7271E-03	-3.96	2.905E-04	.258E+00		1.1
53	8	E	54	7	E	1018.06787	-2			0.2868E-04					
39	1	A_1	39	0	A_2	1018.07682	-2	.681564E-02	0.4	0.6698E-02	1.73	2.908E-04			1.0
38	1	A_2	38	0	A_1	1018.18524	-2	.757715E-02	0.4	0.7691E-02	-1.50	2.342E-04	-.366E+00	-.262E+00	2.3
9	2	E	10	1	E	1018.21212	0	.638397E-02	0.5	0.6198E-02	2.91	4.046E-04	-.358E+00	-.222E+00	1.8
24	4	A_2	25	3	A_1	1018.24586	4	.815666E-02	0.9	0.4216E-02	-3.37	3.130E-04			1.0
24	4	A_1	25	3	A_2	1018.24586	4			0.4216E-02					
22	2	A_2	21	3	A_1	1018.26859	-1	.111356E-01	0.4	0.5648E-02	-1.44	3.789E-04		-.390E+00	1.2
22	2	A_1	21	3	A_2	1018.26859	3			0.5648E-02					
37	1	A_1	37	0	A_2	1018.29088	0	.869125E-02	0.7	0.8788E-02	-1.11	2.921E-04			1.0
36	1	A_2	36	0	A_1	1018.39368	-1	.978358E-02	0.2	0.9993E-02	-2.14	2.575E-04	-.931E-01	-.287E+00	1.5
1	1	A_2	2	0	A_1	1018.46220	3	.219025E-02	0.8	0.2225E-02	-1.61	6.119E-04	-.224E+00		1.1
35	1	A_1	35	0	A_2	1018.49365	-4	.111579E-01	0.3	0.1131E-01	-1.34	2.938E-04			1.0
14	1	E	13	2	E	1018.49886	2	.746205E-02	0.6	0.7609E-02	-1.97	4.333E-04			1.0
6	0	E	5	1	E	1018.56085	4	.516293E-02	0.5	0.5161E-02	.05	3.842E-04			1.0
31	5	E	32	4	E	1018.58554	-7	.198352E-02	2.5	0.1961E-02	1.13	5.416E-05			1.0
34	1	A_2	34	0	A_1	1018.59091	2	.122351E-01	0.3	0.1273E-01	-4.05	2.746E-04			1.0
49	5	A_2	48	6	A_1	1018.61241	-20			0.1049E-03					
49	5	A_1	48	6	A_2	1018.61241	-20			0.1049E-03					
31	3	E	30	4	E	1018.63952	0	.243559E-02	0.4	0.2417E-02	.77	3.750E-04	.621E-01	.227E+00	1.1
33	1	A_1	33	0	A_2	1018.68531	2	.139567E-01	0.3	0.1426E-01	-2.19	3.026E-04			1.0
40	4	E	39	5	E	1018.75834	-49			0.6326E-03					
32	1	A_2	32	0	A_1	1018.77687	-2	.155057E-01	0.3	0.1590E-01	-2.52	3.000E-04			1.0
38	6	E	39	5	E	1018.83929	-41			0.6779E-03					
31	1	A_1	31	0	A_2	1018.86569	0	.172495E-01	0.3	0.1763E-01	-2.19	3.125E-04	-.248E+00		1.7
30	1	A_2	30	0	A_1	1018.95169	-1	.187365E-01	0.2	0.1944E-01	-3.78	2.797E-04	-.265E-01	-.396E+00	2.3
45	7	A_1	46	6	A_2	1019.01162	-9			0.1769E-03					
45	7	A_2	46	6	A_1	1019.01162	-9			0.1769E-03					
29	1	A_1	29	0	A_2	1019.03492	1	.208474E-01	0.2	0.2134E-01	-2.35	3.364E-04			1.0
23	2	A_2	22	3	A_1	1019.07637	3	.106689E-01	0.3	0.5423E-02	-1.67	3.870E-04	-.801E-01	-.153E+00	1.1
23	2	A_1	22	3	A_2	1019.07637	0			0.5423E-02					
28	1	A_2	28	0	A_1	1019.11524	-10			0.2329E-01					
8	2	E	9	1	E	1019.12748	1	.549266E-02	0.8	0.5597E-02	-1.90	3.171E-04			1.0
27	1	A_1	27	0	A_2	1019.19298	1	.243482E-01	0.3	0.2528E-01	-3.83	3.458E-04			1.0
23	4	A_2	24	3	A_1	1019.20588	-5	.853883E-02	0.9	0.4444E-02	-4.09	3.974E-04			1.0
23	4	A_1	24	3	A_2	1019.20588	-5			0.4444E-02					
26	1	A_2	26	0	A_1	1019.26781	-1	.262475E-01	0.2	0.2729E-01	-3.99	3.574E-04			1.0
50	5	A_2	49	6	A_1	1019.31565	-12			0.8679E-04					
50	5	A_1	49	6	A_2	1019.31565	-12			0.8679E-04					
15	1	E	14	2	E	1019.33521	9	.791417E-02	1.2	0.7797E-02	1.48	4.172E-04			1.0
25	1	A_1	25	0	A_2	1019.33988	0	.283325E-01	0.2	0.2930E-01	-3.42	3.455E-04			1.0
24	1	A_2	24	0	A_1	1019.40914	-1	.304255E-01	0.1	0.3128E-01	-2.80	3.752E-04			1.0
32	3	E	31	4	E	1019.41377	-14			0.2195E-02					
7	0	E	6	1	E	1019.42453	3	.611561E-02	0.7	0.6038E-02	1.27	4.286E-04			1.0
23	1	A_1	23	0	A_2	1019.47565	0	.317889E-01	0.1	0.3319E-01	-4.40	3.346E-04	-.594E-01	-.334E+00	1.9
41	4	E	40	5	E	1019.49817	-20			0.5474E-03					

(cont.)

J	K_a	Γ	J'	K'_a	Γ'	$\nu^{a)}$	$\delta_\nu{}^{b)}$	$S_\nu^{\rm exp}(294.45)^{c)}$	$\Delta_S{}^{d)}$	$S_\nu^{\rm calc}(294.45)^{e)}$	$\delta_S{}^{f)}$	$\Gamma_0{}^{g)}$	$\Delta_2{}^{g)}$	$\nu_{\rm VC}{}^{g)}$	$R^{h)}$
1			2			3	4	5	6	7	8	9	10	11	12
22	1	A_2	22	0	A_1	1019.53905	-31			0.3501E-01					
30	5	E	31	4	E	1019.56543	1	.213239E-02	1.9	0.2155E-02	-1.06	3.110E-04			1.0
21	1	A_1	21	0	A_2	1019.60028	-1	.356256E-01	0.6	0.3670E-01	-3.01	2.991E-04	.176E-01	-.472E+00	1.2
20	1	A_2	20	0	A_1	1019.65846	1	.365027E-01	0.2	0.3823E-01	-4.72	3.979E-04			1.0
15	3	E	16	2	E	1019.69286	1	.637162E-02	0.6	0.6476E-02	-1.63	4.434E-04			1.0
19	1	A_1	19	0	A_2	1019.71401	19	.395450E-01	0.2	0.3956E-01	-.03	5.127E-04	.221E+00	-.490E+00	2.0
18	1	A_2	18	0	A_1	1019.76645	3	.401106E-01	0.2	0.4066E-01	-1.36	4.153E-04	-.106E+00		1.1
17	1	A_1	17	0	A_2	1019.81627	3	.415461E-01	0.2	0.4149E-01	.14	3.980E-04			1.0
16	1	A_2	16	0	A_1	1019.86325	-5	.417674E-01	0.7	0.4202E-01	-.60	4.883E-04			1.0
24	2	A_2	23	3	A_1	1019.88052	-1	.102448E-01	0.2	0.5170E-02	-.93	4.002E-04	.105E+00	-.143E+00	1.5
24	2	A_1	23	3	A_2	1019.88052	2			0.5170E-02					
15	1	A_1	15	0	A_2	1019.90758	1	.403133E-01	0.1	0.4223E-01	-4.74	4.082E-04			1.0
14	1	A_2	14	0	A_1	1019.94908	0	.399817E-01	0.1	0.4208E-01	-5.24	3.944E-04	-.470E-01	-.223E+00	1.3
13	1	A_1	13	0	A_2	1019.98782	1	.394403E-01	0.1	0.4156E-01	-5.36	3.911E-04	-.225E+00	-.147E+00	2.4
12	1	A_2	12	0	A_1	1020.02380	2	.385353E-01	0.3	0.4064E-01	-5.47	4.331E-04			1.0
7	2	E	8	1	E	1020.03972	1	.487882E-02	1.6	0.4914E-02	-.72	3.453E-04			1.0
11	1	A_1	11	0	A_2	1020.05699	2	.376166E-01	0.3	0.3933E-01	-4.56	3.823E-04			1.0
10	1	A_2	10	0	A_1	1020.08742	2	.358272E-01	0.1	0.3762E-01	-4.99	3.689E-04	-.157E+00	-.337E+00	2.4
9	1	A_1	9	0	A_2	1020.11508	2	.345286E-01	0.2	0.3550E-01	-2.81	3.800E-04			1.0
51	8	E	52	7	E	1020.13996	25			0.4289E-04					
8	1	A_2	8	0	A_1	1020.13996	1	.316774E-01	0.1	0.3299E-01	-4.14	3.423E-04	-.165E+00	-.366E+00	2.7
7	1	A_1	7	0	A_2	1020.16225	18	.388468E-01	0.3	0.3011E-01	-1.44	5.233E-04			1.0
16	1	E	15	2	E	1020.16790	-2	.781285E-02	1.7	0.7902E-02	-1.14	3.195E-04			1.0
6	1	A_2	6	0	A_1	1020.18143	0	.264980E-01	9.8	0.2687E-01	-1.41	4.934E-04			1.0
5	1	A_1	5	0	A_2	1020.19804	2	.237776E-01	0.4	0.2332E-01	1.92	3.589E-04			1.0
4	1	A_2	4	0	A_1	1020.21187	2	.196615E-01	0.3	0.1949E-01	.89	3.778E-04			1.0
3	1	A_1	3	0	A_2	1020.22295	4	.158255E-01	0.4	0.1541E-01	2.60	4.142E-04			1.0
2	1	A_2	2	0	A_1	1020.23125	5	.114172E-01	0.6	0.1115E-01	2.34	5.319E-04			1.0
1	1	A_1	1	0	A_2	1020.23680	7	.709695E-02	-1.0	0.6747E-02	4.93	6.796E-04			1.0
8	0	E	7	1	E	1020.28488	5	.693580E-02	0.3	0.6838E-02	1.41	4.024E-04	-.850E-01		1.1
29	5	E	30	4	E	1020.54239	-4	.228758E-02	0.7	0.2354E-02	-2.89	3.326E-04	-.145E+00		1.1
14	3	E	15	2	E	1020.62634	-16			0.6345E-02					
25	2	A_2	24	3	A_1	1020.68104	1	.964111E-02	0.5	0.4894E-02	-1.52	5.117E-04			1.0
25	2	A_1	24	3	A_2	1020.68104	-1			0.4894E-02					
63	2	E	63	1	E	1020.72107	-7			0.3268E-04					
36	6	E	37	5	E	1020.83452	-8			0.8768E-03					
62	2	E	62	1	E	1020.92319	-2			0.4178E-04					
6	2	E	7	1	E	1020.94892	11			0.4156E-02					
43	4	E	42	5	E	1020.96555	-11			0.4035E-03					
17	1	E	16	2	E	1020.99721	0	.783399E-02	0.5	0.7928E-02	-1.21	3.422E-04	.199E+00	-.370E+00	1.3
43	7	A_1	44	6	A_2	1021.04310	-16			0.2452E-03					
43	7	A_2	44	6	A_1	1021.04310	-16			0.2452E-03					
21	4	A_2	22	3	A_1	1021.11742	-2			0.4830E-02					
21	4	A_1	22	3	A_2	1021.11742	-2			0.4830E-02					
1	1	A_2	0	0	A_1	1021.12258	1		1	0.4521E-02					
9	0	E	8	1	E	1021.14180	1	.763373E-02	0.4	0.7553E-02	1.05	3.194E-04		-.659E+00	1.4
57	9	E	58	8	E	1021.22225	-141			0.8453E-05					
60	2	E	60	1	E	1021.31793	1			0.6736E-04					
26	2	A_2	25	3	A_1	1021.47793	1	.918779E-02	0.5	0.4601E-02	-.16	3.956E-04			1.0
26	2	A_1	25	3	A_2	1021.47793	2			0.4601E-02					
28	5	E	29	4	E	1021.51663	-2	.242945E-02	1.1	0.2555E-02	-5.17	2.710E-04			1.0
13	3	E	14	2	E	1021.55713	1	.613027E-02	0.6	0.6139E-02	-.14	4.193E-04			1.0
44	4	E	43	5	E	1021.69378	40			0.3437E-03					
58	2	E	58	1	E	1021.69987	-16			0.1067E-03					
35	3	E	34	4	E	1021.71443	3			0.1588E-02					
18	1	E	17	2	E	1021.82300	2	.780221E-02	0.3	0.7881E-02	-1.01	4.144E-04			1.0
35	6	E	36	5	E	1021.82768	-31	.102176E-02	3.1	0.9890E-03	3.20	1.562E-04			1.0
5	2	E	6	1	E	1021.85479	1	.342083E-02	0.4	0.3335E-02	2.52	3.904E-04	-.219E-01	-.196E+00	1.1
57	2	E	57	1	E	1021.88639	4			0.1334E-03					
10	0	E	9	1	E	1021.99539	3	.830776E-02	0.4	0.8177E-02	1.57	3.815E-04			1.0
2	1	A_1	1	0	A_2	1022.00189	1	.695543E-02	0.5	0.6760E-02	2.80	5.476E-04			1.0
42	7	A_2	43	6	A_1	1022.05495	-18			0.2866E-03					
42	7	A_1	43	6	A_2	1022.05495	-18			0.2866E-03					
20	4	A_2	21	3	A_1	1022.06884	2	.958316E-02	0.5	0.4976E-02	-3.85	4.118E-04			1.0
20	4	A_1	21	3	A_2	1022.06884	2			0.4976E-02					
56	2	E	56	1	E	1022.06996	45			0.1661E-03					
54	5	A_2	53	6	A_1	1022.08720	-24			0.3870E-04					
54	5	A_1	53	6	A_2	1022.08720	-24			0.3870E-04					
49	8	E	50	7	E	1022.20186	34			0.6293E-04					
55	2	E	55	1	E	1022.24946	-6			0.2058E-03					
56	9	E	57	8	E	1022.27010	-31			0.1055E-04					
27	2	A_2	26	3	A_1	1022.27114	1	.857834E-02	0.3	0.4298E-02	-.20	4.020E-04			1.0
27	2	A_1	26	3	A_2	1022.27114	2			0.4298E-02					
45	4	E	44	5	E	1022.41705	-8			0.2913E-03					
54	2	E	54	1	E	1022.42640	4			0.2538E-03					
36	3	E	35	4	E	1022.47339	-23			0.1410E-02					
12	3	E	13	2	E	1022.48475	4			0.5854E-02					
27	5	E	28	4	E	1022.48807	3			0.2756E-02					
53	2	E	53	1	E	1022.59997	-8			0.3118E-03					
19	1	E	18	2	E	1022.64524	2	.767473E-02	0.5	0.7766E-02	-1.19	3.750E-04	-.480E+00	-.209E+00	1.7
4	2	E	5	1	E	1022.75760	2	.250564E-02	0.4	0.2466E-02	1.57	3.403E-04	-.173E+00	-.182E+00	1.4
55	5	A_1	54	6	A_2	1022.77040	38			0.3123E-04					

(cont.)

J	K_a	Γ	J'	K_a'	Γ'	$\nu^{a)}$	$\delta_\nu{}^{b)}$	$S_\nu^{\rm exp}(294.45)^{c)}$	$\Delta_S{}^{d)}$	$S_\nu^{\rm calc}(294.45)^{e)}$	$\delta_S{}^{f)}$	$\Gamma_0{}^{g)}$	$\Delta_2{}^{g)}$	$\nu_{\rm VC}{}^{g)}$	$R^{h)}$
1			2			3	4	5	6	7	8	9	10	11	12
55	5	A_2	54	6	A_1	1022.77040	38			0.3123E-04					
52	2	E	52	1	E	1022.77040	-16			0.3812E-03					
34	6	E	35	5	E	1022.81855	-11	.112386E-02	1.3	0.1109E-02	1.28	3.866E-04			1.0
11	0	E	10	1	E	1022.84555	4	.884671E-02	0.3	0.8705E-02	1.61	4.052E-04	-.936E-01		1.1
3	1	A_2	2	0	A_1	1022.87745	2	.922872E-02	0.3	0.8947E-02	3.05	4.497E-04	-.123E+00		1.1
51	2	E	51	1	E	1022.93789	-2			0.4639E-03					
19	4	A_2	20	3	A_1	1023.01727	0	.103910E-01	0.3	0.5083E-02	2.16	4.424E-04		-.268E+00	1.1
19	4	A_1	20	3	A_2	1023.01727	0			0.5083E-02					
28	2	A_2	27	3	A_1	1023.06066	3	.808510E-02	0.1	0.3989E-02	1.32	4.566E-04			1.0
28	2	A_1	27	3	A_2	1023.06066	-1			0.3989E-02					
41	7	A_1	42	6	A_2	1023.06434	-4			0.3332E-03					
41	7	A_2	42	6	A_1	1023.06434	-4			0.3332E-03					
50	2	E	50	1	E	1023.10205	-4			0.5621E-03					
46	4	E	45	5	E	1023.13720	31			0.2456E-03					
48	8	E	49	7	E	1023.22905	41			0.7567E-04					
37	3	E	36	4	E	1023.22905	4	.125352E-02	0.8	0.1245E-02	.71	2.974E-04			1.0
49	2	E	49	1	E	1023.26306	-3			0.6779E-03					
11	3	E	12	2	E	1023.40928	3	.544674E-02	0.3	0.5491E-02	-.80	3.595E-04	-.127E+00	-.311E+00	1.4
48	2	E	48	1	E	1023.42105	13			0.8139E-03					
56	5	A_1	55	6	A_2	1023.44853	9			0.2509E-04					
56	5	A_2	55	6	A_1	1023.44853	9			0.2509E-04					
26	5	E	27	4	E	1023.45660	1	.303663E-02	1.2	0.2953E-02	2.74	3.983E-04			1.0
20	1	E	19	2	E	1023.46390	-1	.749747E-02	0.4	0.7590E-02	-1.24	4.242E-04			1.0
47	2	E	47	1	E	1023.57535	-23			0.9728E-03					
3	2	E	4	1	E	1023.65727	6	.157456E-02	0.6	0.1577E-02	-.13	3.908E-04		-.572E+00	1.2
12	0	E	11	1	E	1023.69226	1	.916448E-02	0.2	0.9134E-02	.34	3.483E-04	-.791E-01	-.467E+00	1.8
46	2	E	46	1	E	1023.72687	-18	.122404E-02	1.5	0.1157E-02	5.45	2.991E-04			1.0
4	1	A_1	3	0	A_2	1023.74923	3	.108181E-01	0.4	0.1105E-01	-2.19	2.956E-04	-.427E-01	-.479E+00	1.7
29	2	A_2	28	3	A_1	1023.84648	-4	.732690E-02	0.5	0.3679E-02	-.44	3.244E-04	-.538E-01	-.437E+00	1.2
29	2	A_1	28	3	A_2	1023.84648	4			0.3679E-02					
47	4	E	46	5	E	1023.85266	1			0.2060E-03					
45	2	E	45	1	E	1023.87529	-6	.139920E-02	0.9	0.1370E-02	2.05	2.530E-04	.139E+00		1.1
18	4	A_2	19	3	A_1	1023.96277	1	.103313E-01	0.3	0.5146E-02	.38	4.110E-04			1.0
18	4	A_1	19	3	A_2	1023.96277	1			0.5146E-02					
38	3	E	37	4	E	1023.98049	-5	.108136E-02	2.2	0.1093E-02	-1.05	1.818E-04	-.117E+00		1.0
44	2	E	44	1	E	1024.02052	6			0.1615E-02					
40	7	A_2	41	6	A_1	1024.07091	-7	.746818E-03	0.6	0.3854E-03	-3.20	2.753E-04	-.963E+00	.266E+00	1.7
40	7	A_1	41	6	A_2	1024.07091	-7			0.3854E-03					
57	5	A_2	56	6	A_1	1024.12280	13			0.2006E-04					
57	5	A_1	56	6	A_2	1024.12280	13			0.2006E-04					
43	2	E	43	1	E	1024.16242	4	.192985E-02	0.9	0.1895E-02	1.80	2.557E-04			1.0
21	1	E	20	2	E	1024.27905	2	.728114E-02	0.3	0.7361E-02	-1.10	3.212E-04		-.613E+00	1.8
42	2	E	42	1	E	1024.30116	4	.225226E-02	1.0	0.2213E-02	1.74	3.028E-04	-.163E+00		1.1
10	3	E	11	2	E	1024.33073	2	.496978E-02	0.3	0.5049E-02	-1.59	3.358E-04	-.216E+00	-.172E+00	1.5
25	5	E	26	4	E	1024.42229	-1	.301829E-02	0.5	0.3144E-02	-4.15	3.169E-04	-.355E-01	-.318E+00	1.2
41	2	E	41	1	E	1024.43664	-3	.258016E-02	0.8	0.2572E-02	.31	2.662E-04	-.316E+00		1.5
13	0	E	12	1	E	1024.53558	3	.954921E-02	0.2	0.9463E-02	.91	3.803E-04	.234E-01	-.206E+00	1.1
2	2	E	3	1	E	1024.55353	-13			0.7136E-03					
48	4	E	47	5	E	1024.56449	11			0.1720E-03					
40	2	E	40	1	E	1024.56903	1	.291382E-02	0.4	0.2975E-02	-2.11	2.666E-04			1.0
5	1	A_2	4	0	A_1	1024.61721	3	.134918E-01	0.3	0.1306E-01	3.22	3.527E-04			1.0
30	2	A_1	29	3	A_2	1024.62870	4			0.3373E-02					
30	2	A_2	29	3	A_1	1024.62870	16			0.3373E-02					
39	2	E	39	1	E	1024.69818	0	.336210E-02	0.6	0.3425E-02	-1.88	2.831E-04	-.108E+00		1.1
39	3	E	38	4	E	1024.72821	-1	.984913E-03	1.2	0.9542E-03	3.12	4.937E-04			1.0
38	2	E	38	1	E	1024.82409	-5	.403758E-02	0.5	0.3925E-02	2.80	2.952E-04	-.250E+00	-.293E+00	1.4
17	4	A_2	18	3	A_1	1024.90529	-1	.102950E-01	0.2	0.5158E-02	-.21	4.117E-04	-.133E+00		1.1
17	4	A_1	18	3	A_2	1024.90529	-1			0.5158E-02					
37	2	E	37	1	E	1024.94692	2	.444302E-02	0.5	0.4475E-02	-.72	2.854E-04	-.137E+00		1.2
36	2	E	36	1	E	1025.06639	-7	.524993E-02	0.6	0.5078E-02	3.28	3.014E-04			1.0
39	7	A_2	40	6	A_1	1025.07494	0	.891376E-03	2.6	0.4434E-03	.52	3.249E-04			1.0
39	7	A_1	40	6	A_2	1025.07494	0			0.4434E-03					
22	1	E	21	2	E	1025.09049	-8	.705192E-02	0.4	0.7086E-02	-.48	4.215E-04			1.0
35	2	E	35	1	E	1025.18300	18			0.5733E-02					
9	3	E	10	2	E	1025.24902	-8	.434784E-02	0.4	0.4532E-02	-4.24	4.089E-04			1.0
49	4	E	48	5	E	1025.27192	-15			0.1428E-03					
46	8	E	47	7	E	1025.27527	3			0.1078E-03					
34	2	E	34	1	E	1025.29591	-6	.622806E-02	0.5	0.6442E-02	-3.43	2.895E-04			1.0
14	0	E	13	1	E	1025.37533	-6	.992365E-02	0.4	0.9693E-02	2.32	4.179E-04			1.0
24	5	E	25	4	E	1025.38561	46			0.3322E-02					
33	2	E	33	1	E	1025.40630	39	.136228E-01	0.2	0.7202E-02	2.00	4.968E-04	.123E+00	-.783E+00	3.4
31	2	A_2	30	3	A_1	1025.40630	-77			0.3074E-02					
40	3	E	39	4	E	1025.47210	10	.857686E-03	5.6	0.8288E-03	3.37	1.548E-04			1.0
6	1	A_1	5	0	A_2	1025.48137	2			0.1493E-01					
32	2	E	32	1	E	1025.51256	-9	.780160E-02	0.3	0.8010E-02	-2.68	2.946E-04	-.612E-01	-.190E+00	1.1
31	2	E	31	1	E	1025.61626	9	.957173E-02	0.7	0.8865E-02	7.39	6.173E-04	.129E+01	-.309E+00	2.2
30	2	E	30	1	E	1025.71641	-7	.953285E-02	0.4	0.9759E-02	-2.38	3.338E-04	-.350E-01		1.0
31	6	E	32	5	E	1025.77411	-11	.149039E-02	1.8	0.1513E-02	-1.51	4.317E-04			1.0
29	2	E	29	1	E	1025.81350	-7	.104401E-01	0.5	0.1069E-01	-2.38	3.490E-04			1.0
16	4	A_2	17	3	A_1	1025.84477	-8	.100305E-01	0.5	0.5116E-02	-2.00	4.231E-04			1.0
16	4	A_1	17	3	A_2	1025.84477	-8			0.5116E-02					
23	1	E	22	2	E	1025.89844	-6	.681804E-02	0.5	0.6773E-02	.67	3.691E-04			1.0

(cont.)

J	K_a	Γ	J'	K'_a	Γ'	$\nu^{a)}$	$\delta_\nu{}^{b)}$	$S_\nu^{\rm exp}(294.45)^{c)}$	$\Delta_S{}^{d)}$	$S_\nu^{\rm calc}(294.45)^{e)}$	$\delta_S{}^{f)}$	$\Gamma_0{}^{g)}$	$\Delta_2{}^{g)}$	$\nu_{\rm VC}{}^{g)}$	$R^{h)}$
1			2			3	4	5	6	7	8	9	10	11	12
28	2	E	28	1	E	1025.90740	-5	.114558E-01	0.9	0.1164E-01	-1.64	3.110E-04			1.0
50	4	E	49	5	E	1025.97587	17			0.1180E-03					
27	2	E	27	1	E	1025.99800	-10	.125440E-01	0.5	0.1262E-01	-.57	3.909E-04			1.0
38	7	A_2	39	6	A_1	1026.07617	-6	.107635E-02	7.1	0.5074E-03	5.72	4.276E-04			1.0
38	7	A_1	39	6	A_2	1026.07617	-6			0.5074E-03					
26	2	E	26	1	E	1026.08547	-7	.132599E-01	0.4	0.1359E-01	-2.52	3.618E-04			1.0
8	3	E	9	2	E	1026.16430	-8	.387333E-02	1.5	0.3946E-02	-1.87	3.580E-04			1.0
25	2	E	25	1	E	1026.16969	-6	.145766E-01	0.4	0.1457E-01	.07	3.740E-04			1.0
32	2	A_2	31	3	A_1	1026.18155	5	.547281E-02	1.0	0.2786E-02	-1.80	4.210E-04			1.0
32	2	A_1	31	3	A_2	1026.18155	-19			0.2786E-02					
15	0	E	14	1	E	1026.21171	-5			0.9827E-02					
41	3	E	40	4	E	1026.21171	-18			0.7161E-03					
24	2	E	24	1	E	1026.25067	-7	.157453E-01	0.3	0.1552E-01	1.43	4.082E-04			1.0
45	8	E	46	7	E	1026.29465	-4			0.1278E-03					
23	2	E	23	1	E	1026.32844	-6	.160236E-01	0.2	0.1644E-01	-2.59	3.600E-04			1.0
7	1	A_2	6	0	A_1	1026.34164	-5	.173286E-01	2.2	0.1665E-01	3.89	1.923E-04			1.0
23	5	E	24	4	E	1026.34511	-1	.350566E-02	2.2	0.3485E-02	.58	1.923E-04			1.0
22	2	E	22	1	E	1026.40298	-5	.171398E-01	0.4	0.1731E-01	-.99	3.679E-04	-.413E-01	-.479E+00	1.3
21	2	E	21	1	E	1026.47426	-8	.181276E-01	0.2	0.1811E-01	.09	3.961E-04			1.0
20	2	E	20	1	E	1026.54235	-6	.180928E-01	0.3	0.1883E-01	-4.09	3.877E-04			1.0
19	2	E	19	1	E	1026.60718	-7	.191307E-01	0.3	0.1945E-01	-1.68	3.811E-04			1.0
18	2	E	18	1	E	1026.66878	-8	.192755E-01	0.3	0.1996E-01	-3.53	4.034E-04			1.0
51	4	E	50	5	E	1026.67526	0			0.9707E-04					
24	1	E	23	2	E	1026.70276	-7	.638621E-02	0.5	0.6429E-02	-.67	3.876E-04	-.766E-01	-.973E-01	1.1
17	2	E	17	1	E	1026.72703	-20			0.2032E-01					
30	6	E	31	5	E	1026.75387	0			0.1658E-02					
15	4	A_2	16	3	A_1	1026.78200	59			0.5014E-02					
15	4	A_1	16	3	A_2	1026.78200	59			0.5014E-02					
16	2	E	16	1	E	1026.78200	-37			0.2055E-01					
15	2	E	15	1	E	1026.83422	-5			0.2060E-01					
14	2	E	14	1	E	1026.88287	-6	.202653E-01	0.3	0.2049E-01	-1.09	4.127E-04			1.0
13	2	E	13	1	E	1026.92829	-6	.206226E-01	0.3	0.2018E-01	2.13	4.606E-04	-.166E+00	.838E+00	1.2
42	3	E	41	4	E	1026.94795	9			0.6155E-03					
33	2	A_2	32	3	A_1	1026.95242	-23	.499612E-02	0.5	0.2509E-02	-.46	4.968E-04			1.0
33	2	A_1	32	3	A_2	1026.95242	9			0.2509E-02					
12	2	E	12	1	E	1026.97048	-5	.199146E-01	0.2	0.1969E-01	1.15	3.967E-04	-.914E-02		1.4
11	2	E	11	1	E	1027.00950	3	.186600E-01	0.2	0.1899E-01	-1.76	3.468E-04	-.697E-01	-.364E+00	1.6
16	0	E	15	1	E	1027.04501	37	.279190E-01	0.1	0.9869E-02	-.15	4.120E-04	-.515E-01	-.395E+00	1.7
10	2	E	10	1	E	1027.04501	-16			0.1809E-01					
9	2	E	9	1	E	1027.07752	-11	.212118E-01	0.2	0.1699E-01	4.35	5.213E-04	-.673E+00	-.194E+00	2.7
7	3	E	8	2	E	1027.07752	96			0.3298E-02					
8	2	E	8	1	E	1027.10687	3	.160128E-01	0.2	0.1569E-01	2.02	3.776E-04	-.859E-01	.155E+00	1.1
7	2	E	7	1	E	1027.13283	2	.145874E-01	0.3	0.1419E-01	2.70	3.963E-04	-.160E+00	.133E+00	1.5
6	2	E	6	1	E	1027.15556	3	.126696E-01	0.2	0.1251E-01	1.28	3.387E-04	-.120E+00	-.312E+00	2.4
5	2	E	5	1	E	1027.17507	6	.108990E-01	0.2	0.1063E-01	2.45	3.216E-04	-.192E+00	-.454E+00	2.6
4	2	E	4	1	E	1027.19133	9			0.8564E-02					
8	1	A_1	7	0	A_2	1027.19821	1	.188646E-01	0.2	0.1821E-01	3.47	3.833E-04			1.0
3	2	E	3	1	E	1027.20430	7	.654860E-02	0.7	0.6271E-02	4.24	5.088E-04			1.0
2	2	E	2	1	E	1027.21399	2	.332514E-02	1.3	0.3628E-02	-9.11	5.889E-04			1.0
22	5	E	23	4	E	1027.30219	-1	.349924E-02	0.6	0.3628E-02	-3.68	3.048E-04			1.0
44	8	E	45	7	E	1027.31162	6			0.1506E-03					
52	4	E	51	5	E	1027.37137	65			0.7944E-04					
51	9	E	52	8	E	1027.46744	7			0.2976E-04					
25	1	E	24	2	E	1027.50357	6	.597998E-02	0.4	0.6063E-02	-1.39	6.340E-04	.350E+00	.829E+00	1.2
43	3	E	42	4	E	1027.67989	0			0.5264E-03					
14	4	A_2	15	3	A_1	1027.71500	4	.985649E-02	0.7	0.4849E-02	1.60	4.218E-04			1.0
14	4	A_1	15	3	A_2	1027.71500	4			0.4849E-02					
34	2	A_2	33	3	A_1	1027.71951	14	.452712E-02	0.7	0.2248E-02	.68	4.218E-04			1.0
34	2	A_1	33	3	A_2	1027.71951	-27			0.2248E-02					
29	6	E	30	5	E	1027.73071	-2	.189510E-02	3.7	0.1805E-02	4.75	6.109E-04			1.0
17	0	E	16	1	E	1027.87404	3	.100692E-01	0.4	0.9824E-02	2.44	3.724E-04		-.374E+00	1.2
62	3	E	62	2	E	1027.93541	3			0.3743E-04					
6	3	E	7	2	E	1027.98566	6	.257614E-02	0.4	0.2603E-02	-1.06	3.386E-04			1.0
9	1	A_2	8	0	A_1	1028.05088	3	.188937E-01	0.4	0.1958E-01	-3.64	3.099E-04	.101E+00	-.466E+00	1.3
63	5	A_2	62	6	A_1	1028.07991	79			0.4738E-05					
63	5	A_1	62	6	A_2	1028.07991	79			0.4738E-05					
21	5	E	22	4	E	1028.25635	-2	.363546E-02	0.7	0.3746E-02	-3.04	3.992E-04	-.115E+00	-.293E+00	1.1
26	1	E	25	2	E	1028.30057	2	.560538E-02	0.4	0.5681E-02	-1.34	3.802E-04	-.254E+00		1.2
44	3	E	43	4	E	1028.40790	-7			0.4478E-03					
35	2	A_2	34	3	A_1	1028.48288	-25	.389456E-02	0.9	0.2003E-02	-2.85	5.967E-04	-.263E+00		1.1
35	2	A_1	34	3	A_2	1028.48288	28			0.2003E-02					
50	9	E	51	8	E	1028.49900	-31			0.3610E-04					
57	10	A_1	58	9	A_2	1028.59324	-111			0.5623E-05					
57	10	A_2	58	9	A_1	1028.59324	-111			0.5623E-05					
13	4	A_2	14	3	A_1	1028.64552	3	.933363E-02	0.2	0.4620E-02	1.00	4.428E-04			1.0
13	4	A_1	14	3	A_2	1028.64552	3			0.4620E-02					
18	0	E	17	1	E	1028.69989	2	.968809E-02	0.4	0.9698E-02	-.10	4.534E-04			1.0
28	6	E	29	5	E	1028.70479	1			0.1953E-02					
5	3	E	6	2	E	1028.89142	-9	.194441E-02	2.5	0.1880E-02	3.32	3.821E-04			1.0
10	1	A_1	9	0	A_2	1028.89967	3	.205601E-01	0.2	0.2076E-01	-.97	3.753E-04			1.0
57	3	E	57	2	E	1028.90616	9			0.1201E-03					
2	2	E	1	1	E	1028.98759	6	.679101E-02	0.3	0.6599E-02	2.83	3.741E-04	-.102E+00	-.394E+00	1.6

(cont.)

J	K_a	Γ	J'	K'_a	Γ'	$\nu^{a)}$	$\delta_\nu{}^{b)}$	$S_\nu^{\rm exp}(294.45)^{c)}$	$\Delta_S{}^{d)}$	$S_\nu^{\rm calc}(294.45)^{e)}$	$\delta_S{}^{f)}$	$\Gamma_0{}^{g)}$	$\Delta_2{}^{g)}$	$\nu_{\rm VC}{}^{g)}$	$R^{h)}$
	1			2		3	4	5	6	7	8	9	10	11	12
35	7	A_2	36	6	A_1	1029.06392	-5	.138853E-02	1.5	0.7358E-03	-5.99	2.277E-04			1.0
35	7	A_1	36	6	A_2	1029.06392	-5			0.7358E-03					
27	1	E	26	2	E	1029.09398	5	.528791E-02	4.9	0.5289E-02	-.03	6.640E-04			1.0
45	3	E	44	4	E	1029.13211	3			0.3790E-03					
20	5	E	21	4	E	1029.20772	10			0.3834E-02					
36	2	A_2	35	3	A_1	1029.24234	32	.361754E-02	0.7	0.1774E-02	1.90	6.366E-04			1.0
36	2	A_1	35	3	A_2	1029.24234	-32			0.1774E-02					
55	3	E	55	2	E	1029.27165	-9			0.1856E-03					
42	8	E	43	7	E	1029.33745	-5			0.2061E-03					
55	4	E	54	5	E	1029.43232	-4			0.4228E-04					
54	3	E	54	2	E	1029.44966	-7			0.2291E-03					
19	0	E	18	1	E	1029.52221	3	.988714E-02	0.4	0.9499E-02	3.92	4.211E-04			1.0
49	9	E	50	8	E	1029.52842	-32			0.4357E-04					
12	4	A_2	13	3	A_1	1029.57300	2	.833231E-02	0.4	0.4326E-02	-3.83	3.180E-04		-.389E+00	1.2
12	4	A_1	13	3	A_2	1029.57300	2			0.4326E-02					
53	3	E	53	2	E	1029.62447	-3			0.2817E-03					
56	10	A_2	57	9	A_1	1029.64000	-86			0.7012E-05					
56	10	A_1	57	9	A_2	1029.64000	-86			0.7012E-05					
27	6	E	28	5	E	1029.67596	-6	.209266E-02	1.0	0.2099E-02	-.31	3.588E-04			1.0
11	1	A_2	10	0	A_1	1029.74459	4	.218855E-01	0.2	0.2173E-01	.69	3.894E-04	.117E+00		1.2
4	3	E	5	2	E	1029.79455	29	.110071E-02	1.1	0.1158E-02	-5.25	7.570E-04			1.0
46	3	E	45	4	E	1029.85221	1			0.3192E-03					
3	2	E	2	1	E	1029.86459	5	.745731E-02	0.5	0.7278E-02	2.41	3.866E-04	-.714E+00		2.0
28	1	E	27	2	E	1029.88342	-20			0.4895E-02					
51	3	E	51	2	E	1029.96436	2			0.4200E-03					
37	2	A_2	36	3	A_1	1029.99800	-38	.316476E-02	1.0	0.1564E-02	1.18	7.334E-04			1.0
37	2	A_1	36	3	A_2	1029.99800	41			0.1564E-02					
56	4	E	55	5	E	1030.11182	55			0.3393E-04					
50	3	E	50	2	E	1030.12944	2			0.5093E-03					
19	5	E	20	4	E	1030.15592	-2	.386174E-02	1.0	0.3888E-02	-.68	4.339E-04			1.0
49	3	E	49	2	E	1030.29120	-7			0.6149E-03					
20	0	E	19	1	E	1030.34097	3	.926913E-02	0.3	0.9235E-02	.37	4.052E-04			1.0
48	3	E	48	2	E	1030.45003	14			0.7390E-03					
11	4	A_2	12	3	A_1	1030.49744	2	.788896E-02	0.6	0.3967E-02	-.57	4.198E-04			1.0
11	4	A_1	12	3	A_2	1030.49744	2			0.3967E-02					
48	9	E	49	8	E	1030.55514	-49			0.5234E-04					
47	3	E	46	4	E	1030.56843	11			0.2675E-03					
12	1	A_1	11	0	A_2	1030.58558	2	.225157E-01	0.2	0.2250E-01	.07	3.609E-04	-.151E+00	-.442E+00	1.6
47	3	E	47	2	E	1030.60528	0			0.8841E-03					
26	6	E	27	5	E	1030.64433	-9	.224576E-02	0.6	0.2241E-02	.23	4.282E-04			1.0
29	1	E	28	2	E	1030.66962	1	.456139E-02	0.3	0.4503E-02	1.28	2.658E-04		-.406E+00	1.4
3	3	E	4	2	E	1030.69356	-28			0.4937E-03					
4	2	E	3	1	E	1030.73829	3	.859557E-02	0.6	0.8092E-02	5.86	3.842E-04			1.0
38	2	A_2	37	3	A_1	1030.74993	63			0.1371E-02					
38	2	A_1	37	3	A_2	1030.74993	-32			0.1371E-02					
46	3	E	46	2	E	1030.75696	-49			0.1053E-02					
45	3	E	45	2	E	1030.90636	-2	.130007E-02	4.1	0.1248E-02	4.02	8.018E-05			1.0
33	7	A_2	34	6	A_1	1031.04219	-3	.179418E-02	0.5	0.9164E-03	-2.15	3.022E-04			1.0
33	7	A_1	34	6	A_2	1031.04219	-3			0.9164E-03					
44	3	E	44	2	E	1031.05202	-7	.148884E-02	0.6	0.1472E-02	1.11	3.218E-04			1.0
18	5	E	19	4	E	1031.10132	1	.394886E-02	0.6	0.3903E-02	1.16	4.272E-04	-.272E+00		1.2
21	0	E	20	1	E	1031.15614	1	.897479E-02	0.2	0.8915E-02	.67	4.123E-04	-.141E+00		1.1
43	3	E	43	2	E	1031.19454	-2	.175277E-02	0.6	0.1729E-02	1.36	2.688E-04			1.0
42	3	E	42	2	E	1031.33383	2	.203705E-02	0.6	0.2021E-02	.80	2.593E-04	-.321E+00		1.7
40	8	E	41	7	E	1031.35292	-3			0.2763E-03					
10	4	A_2	11	3	A_1	1031.41900	21			0.3546E-02					
10	4	A_1	11	3	A_2	1031.41900	21			0.3546E-02					
13	1	A_2	12	0	A_1	1031.42270	4	.224722E-01	0.4	0.2306E-01	-2.61	3.652E-04			1.0
30	1	E	29	2	E	1031.45190	2	.416097E-02	0.4	0.4119E-02	1.02	3.066E-04	-.187E+00	-.124E+00	1.4
41	3	E	41	2	E	1031.46981	-1	.233941E-02	0.6	0.2351E-02	-.49	2.592E-04	-.421E+00		1.6
39	2	A_1	38	3	A_2	1031.49770	57			0.1195E-02					
39	2	A_2	38	3	A_1	1031.49770	-57			0.1195E-02					
40	3	E	40	2	E	1031.60258	-3	.265822E-02	1.7	0.2722E-02	-2.39	1.764E-04			1.0
5	2	E	4	1	E	1031.60889	22	.116380E-01	0.4	0.8919E-02	3.40	5.190E-04			1.0
25	6	E	26	5	E	1031.60889	-108			0.2375E-02					
39	3	E	39	2	E	1031.73214	-2	.309031E-02	0.3	0.3136E-02	-1.49	2.192E-04	-.116E+00	-.318E+00	1.6
38	3	E	38	2	E	1031.85847	-1	.363684E-02	0.4	0.3597E-02	1.11	3.054E-04			1.0
22	0	E	21	1	E	1031.96774	1	.862146E-02	0.3	0.8546E-02	.87	4.220E-04			1.0
37	3	E	37	2	E	1031.98157	0	.403059E-02	0.9	0.4104E-02	-1.83	2.671E-04			1.0
49	3	E	48	4	E	1031.98826	-22			0.1850E-03					
32	7	A_1	33	6	A_2	1032.02782	59			0.1014E-02					
32	7	A_2	33	6	A_1	1032.02782	59			0.1014E-02					
17	5	E	18	4	E	1032.04375	4	.369764E-02	1.2	0.3875E-02	-4.81	3.176E-04			1.0
36	3	E	36	2	E	1032.10141	-1	.466251E-02	0.4	0.4661E-02	.03	2.976E-04			1.0
35	3	E	35	2	E	1032.21807	2	.540952E-02	0.9	0.5267E-02	2.63	3.847E-04			1.0
31	1	E	30	2	E	1032.23044	2	.371001E-02	1.0	0.3745E-02	-.95	3.880E-04			1.0
40	2	A_2	39	3	A_1	1032.24180	73			0.1037E-02					
40	2	A_1	39	3	A_2	1032.24180	-62			0.1037E-02					
14	1	A_1	13	0	A_2	1032.25585	2	.231228E-01	0.3	0.2341E-01	-1.26	4.159E-04			1.0
34	3	E	34	2	E	1032.33140	-4	.594432E-02	0.3	0.5922E-02	.37	2.877E-04			1.0
9	4	A_2	10	3	A_1	1032.33716	8	.601153E-02	0.3	0.3070E-02	-2.13	4.030E-04			1.0
9	4	A_1	10	3	A_2	1032.33716	8			0.3070E-02					

(cont.)

J	K_a	Γ	J'	K'_a	Γ'	$\nu^{a)}$	$\delta_\nu{}^{b)}$	$S_\nu^{\rm exp}(294.45)^{c)}$	$\Delta_S{}^{d)}$	$S_\nu^{\rm calc}(294.45)^{e)}$	$\delta_S{}^{f)}$	$\Gamma_0{}^{g)}$	$\Delta_2{}^{g)}$	$\nu_{\rm VC}{}^{g)}$	$R^{h)}$
1			2			3	4	5	6	7	8	9	10	11	12
39	8	E	40	7	E	1032.35662	-8			0.3173E-03					
33	3	E	33	2	E	1032.44190	31			0.6626E-02					
6	2	E	5	1	E	1032.47581	5	.995055E-02	0.2	0.9711E-02	2.41	3.523E-04	-.137E+00		1.2
32	3	E	32	2	E	1032.54853	1	.756276E-02	0.5	0.7375E-02	2.48	3.529E-04			1.0
24	6	E	25	5	E	1032.57258	-8	.247818E-02	0.7	0.2498E-02	-.79	3.531E-04	.476E-01	-.401E+00	1.1
46	9	E	47	8	E	1032.60145	-33			0.7440E-04					
31	3	E	31	2	E	1032.65225	4	.828189E-02	0.4	0.8167E-02	1.39	4.245E-04	.173E+00		1.1
50	3	E	49	4	E	1032.69269	22			0.1528E-03					
30	3	E	30	2	E	1032.75266	0	.881590E-02	0.4	0.8997E-02	-2.05	2.952E-04			1.0
23	0	E	22	1	E	1032.77574	3	.822353E-02	0.3	0.8139E-02	1.03	4.080E-04	-.237E+00		1.8
29	3	E	29	2	E	1032.84988	0	.100259E-01	0.3	0.9859E-02	1.66	3.564E-04			1.0
28	3	E	28	2	E	1032.94382	-4	.107931E-01	0.4	0.1075E-01	.44	5.039E-04	-.567E+00		1.4
41	2	A_2	40	3	A_1	1032.98306	39			0.8945E-03					
16	5	E	17	4	E	1032.98306	-8			0.3801E-02					
32	1	E	31	2	E	1033.00528	7	.338779E-02	1.6	0.3386E-02	.04	2.642E-04			1.0
31	7	A_2	32	6	A_1	1033.00937	-11	.214242E-02	1.6	0.1114E-02	-4.02	2.642E-04			1.0
31	7	A_1	32	6	A_2	1033.00937	-11			0.1114E-02					
27	3	E	27	2	E	1033.03460	-1	.116560E-01	0.3	0.1165E-01	.06	3.544E-04			1.0
15	1	A_2	14	0	A_1	1033.08508	1	.236470E-01	0.1	0.2357E-01	.34	4.169E-04	-.453E-01	-.131E+00	1.2
26	3	E	26	2	E	1033.12213	1	.122407E-01	0.5	0.1256E-01	-2.59	3.409E-04			1.0
25	3	E	25	2	E	1033.20639	0	.131822E-01	0.4	0.1346E-01	-2.11	3.102E-04	-.219E+00	-.264E+00	1.6
8	4	A_2	9	3	A_1	1033.25231	3	.515253E-02	0.9	0.2545E-02	1.20	2.893E-04			1.0
8	4	A_1	9	3	A_2	1033.25231	3			0.2545E-02					
24	3	E	24	2	E	1033.28742	0	.140395E-01	0.2	0.1434E-01	-2.17	3.131E-04	-.161E+00	-.375E+00	1.7
7	2	E	6	1	E	1033.33957	5	.105867E-01	0.5	0.1044E-01	1.36	3.887E-04			1.0
38	8	E	39	7	E	1033.35758	-20			0.3625E-03					
23	3	E	23	2	E	1033.36523	1	.155601E-01	0.4	0.1520E-01	2.34	3.981E-04			1.0
51	3	E	50	4	E	1033.39224	-16			0.1255E-03					
22	3	E	22	2	E	1033.43970	-8			0.1600E-01					
21	3	E	21	2	E	1033.51111	1	.170133E-01	0.3	0.1674E-01	1.59	4.042E-04	-.113E+00		1.1
23	6	E	24	5	E	1033.53242	-5	.266383E-02	1.2	0.2607E-02	2.15	4.025E-04			1.0
20	3	E	20	2	E	1033.57944	26	.257222E-01	0.3	0.1740E-01	2.40	5.180E-04			1.0
24	0	E	23	1	E	1033.57944	-64			0.7701E-02					
19	3	E	19	2	E	1033.64405	3	.178225E-01	0.3	0.1797E-01	-.83	4.181E-04			1.0
18	3	E	18	2	E	1033.70563	0	.185234E-01	0.2	0.1842E-01	.53	3.958E-04			1.0
17	3	E	17	2	E	1033.76400	1	.186864E-01	0.3	0.1875E-01	-.34	4.153E-04			1.0
33	1	E	32	2	E	1033.77623	0	.309872E-02	1.2	0.3045E-02	1.73	3.477E-04			1.0
16	3	E	16	2	E	1033.81913	2	.184681E-01	0.3	0.1893E-01	-2.52	3.964E-04			1.0
15	3	E	15	2	E	1033.87101	2	.193401E-01	0.2	0.1896E-01	1.98	4.367E-04			1.0
16	1	A_1	15	0	A_2	1033.91037	2	.235834E-01	0.5	0.2353E-01	.23	4.564E-04			1.0
15	5	E	16	4	E	1033.91963	6	.216716E-01	0.6	0.3678E-02	-3.78	4.347E-04			1.0
14	3	E	14	2	E	1033.91963	0			0.1881E-01					
13	3	E	13	2	E	1033.96504	1	.185547E-01	4.6	0.1849E-01	.37	4.630E-04			1.0
30	7	A_2	31	6	A_1	1033.98895	0	.235256E-02	0.4	0.1217E-02	-3.48	3.039E-04	-.252E+00		1.2
30	7	A_1	31	6	A_2	1033.98895	0			0.1217E-02					
12	3	E	12	2	E	1034.00721	2	.180056E-01	0.1	0.1797E-01	.20	3.871E-04	-.282E-01	-.105E+00	1.1
11	3	E	11	2	E	1034.04614	4	.172490E-01	0.2	0.1726E-01	-.04	3.421E-04	-.883E-01	-.493E+00	1.7
10	3	E	10	2	E	1034.08182	5	.164191E-01	0.2	0.1634E-01	.48	4.278E-04	-.123E+00		1.3
52	3	E	51	4	E	1034.08823	0			0.1026E-03					
9	3	E	9	2	E	1034.11423	2	.149588E-01	0.3	0.1522E-01	-1.72	3.472E-04	-.155E+00	-.220E+00	1.5
8	3	E	8	2	E	1034.14343	4	.137889E-01	0.5	0.1388E-01	-.69	3.838E-04			1.0
7	4	A_2	8	3	A_1	1034.16443	6	.409409E-02	2.0	0.1986E-02	2.98	4.967E-04			1.0
7	4	A_1	8	3	A_2	1034.16443	6			0.1986E-02					
7	3	E	7	2	E	1034.16936	2	.125741E-01	0.5	0.1234E-01	1.88	3.556E-04			1.0
6	3	E	6	2	E	1034.19209	5	.105439E-01	0.4	0.1057E-01	-.25	3.793E-04			1.0
8	2	E	7	1	E	1034.19999	5	.112307E-01	0.3	0.1110E-01	1.20	4.290E-04			1.0
5	3	E	5	2	E	1034.21157	7	.862715E-02	0.6	0.8560E-02	.78	3.937E-04			1.0
4	3	E	4	2	E	1034.22776	4	.636773E-02	0.5	0.6257E-02	1.74	3.026E-04		-.488E+00	1.3
3	3	E	3	2	E	1034.24067	-2	.358453E-02	1.2	0.3535E-02	1.39	4.931E-04			1.0
37	8	E	38	7	E	1034.35606	-13			0.4118E-03					
25	0	E	24	1	E	1034.38083	3	.742985E-02	0.5	0.7241E-02	2.54	3.520E-04			1.0
22	6	E	23	5	E	1034.48935	-5	.270049E-02	0.8	0.2698E-02	.10	5.283E-04	-.434E+00		1.2
34	1	E	33	2	E	1034.54345	-2			0.2723E-02					
64	4	A_2	64	3	A_1	1034.60617	4			0.2020E-04					
64	4	A_1	64	3	A_2	1034.60617	3			0.2020E-04					
44	9	E	45	8	E	1034.63750	-16			0.1037E-03					
17	1	A_2	16	0	A_1	1034.73168	2	.234069E-01	0.2	0.2331E-01	.40	4.197E-04			1.0
53	3	E	52	4	E	1034.77988	-6			0.8351E-04					
63	4	A_1	63	3	A_2	1034.81341	-28			0.2596E-04					
63	4	A_2	63	3	A_1	1034.81341	-28			0.2596E-04					
14	5	E	15	4	E	1034.85309	9			0.3503E-02					
29	7	A_2	30	6	A_1	1034.96557	-6	.253719E-02	0.8	0.1321E-02	-4.14	2.957E-04	-.149E+00		1.1
29	7	A_1	30	6	A_2	1034.96557	-6			0.1321E-02					
9	2	E	8	1	E	1035.05706	6	.118160E-01	0.4	0.1166E-01	1.30	3.157E-04	-.102E+00	-.428E+00	1.4
6	4	A_2	7	3	A_1	1035.07321	-12	.275005E-02	0.7	0.1411E-02	-2.60	4.899E-04	-.171E+01		2.5
6	4	A_1	7	3	A_2	1035.07321	-12			0.1411E-02					
26	0	E	25	1	E	1035.17788	2	.814736E-02	0.3	0.6768E-02	3.25	5.419E-04	.387E+00		1.4
44	2	A_2	43	3	A_1	1035.17788	35			0.5574E-03					
61	4	A_1	61	3	A_2	1035.21856	-46			0.4233E-04					
61	4	A_2	61	3	A_1	1035.21856	-47			0.4233E-04					
35	1	E	34	2	E	1035.30678	-12	.248939E-02	0.4	0.2422E-02	2.71	4.305E-04	-.220E+00	-.562E+00	1.7
36	8	E	37	7	E	1035.35188	-3			0.4652E-03					

(cont.)

J	K_a	Γ	J'	K'_a	Γ'	$\nu^{a)}$	$\delta_\nu{}^{b)}$	$S_\nu^{\rm exp}(294.45)^{c)}$	$\Delta_S{}^{d)}$	$S_\nu^{\rm calc}(294.45)^{e)}$	$\delta_S{}^{f)}$	$\Gamma_0{}^{g)}$	$\Delta_2{}^{g)}$	$\nu_{\rm VC}{}^{g)}$	$R^{h)}$
1			2			3	4	5	6	7	8	9	10	11	12
21	6	E	22	5	E	1035.44351	9			0.2767E-02					
54	3	E	53	4	E	1035.46747	-6			0.6762E-04					
18	1	A_1	17	0	A_2	1035.54902	3	.225968E-01	0.1	0.2293E-01	-1.48	3.770E-04	-.470E-01	-.258E+00	1.1
59	4	A_1	59	3	A_2	1035.61103	-31			0.6782E-04					
59	4	A_2	59	3	A_1	1035.61103	-31			0.6782E-04					
13	5	E	14	4	E	1035.78347	7	.336931E-02	0.8	0.3277E-02	2.73	4.055E-04			1.0
58	4	A_2	58	3	A_1	1035.80249	-13			0.8527E-04					
58	4	A_1	58	3	A_2	1035.80249	-13			0.8527E-04					
45	2	A_1	44	3	A_2	1035.90201	29			0.4713E-03					
45	2	A_2	44	3	A_1	1035.90459	15			0.4713E-03					
10	2	E	9	1	E	1035.91074	6	.120158E-01	0.5	0.1213E-01	-.97	3.637E-04			1.0
28	7	A_2	29	6	A_1	1035.93952	2			0.1424E-02					
28	7	A_1	29	6	A_2	1035.93952	2			0.1424E-02					
27	0	E	26	1	E	1035.97127	3	.633986E-02	0.4	0.6287E-02	.83	3.659E-04			1.0
5	4	A_2	6	3	A_1	1035.97930	15	.166748E-02	4.6	0.8489E-03	-1.82	1.985E-04			1.0
5	4	A_1	6	3	A_2	1035.97930	15			0.8489E-03					
57	4	A_1	57	3	A_2	1035.99065	0			0.1067E-03					
57	4	A_2	57	3	A_1	1035.99065	0			0.1067E-03					
36	1	E	35	2	E	1036.06655	4	.213201E-02	0.5	0.2143E-02	-.50	2.999E-04			1.0
55	3	E	54	4	E	1036.15096	-1			0.5450E-04					
56	4	A_2	56	3	A_1	1036.17538	-4			0.1330E-03					
56	4	A_1	56	3	A_2	1036.17538	-4			0.1330E-03					
35	8	E	36	7	E	1036.34496	4			0.5224E-03					
19	1	A_2	18	0	A_1	1036.36236	4	.220411E-01	0.2	0.2240E-01	-1.61	3.843E-04	.258E-01	-.192E+00	1.2
20	6	E	21	5	E	1036.39455	4	.275671E-02	0.6	0.2812E-02	-2.00	4.756E-04		-.463E+00	1.2
54	4	A_1	54	3	A_2	1036.53575	52			0.2039E-03					
54	4	A_2	54	3	A_1	1036.53575	53			0.2039E-03					
46	2	A_2	45	3	A_1	1036.62186	-6			0.3965E-03					
46	2	A_1	45	3	A_2	1036.62517	19			0.3965E-03					
42	9	E	43	8	E	1036.66305	-10			0.1414E-03					
53	4	A_1	53	3	A_2	1036.71054	29			0.2507E-03					
53	4	A_1	53	3	A_2	1036.71070	45			0.2507E-03					
53	4	A_2	53	3	A_1	1036.71070	45			0.2507E-03					
12	5	E	13	4	E	1036.71070	-7			0.3001E-02					
28	0	E	27	1	E	1036.76099	6	.184930E-01	0.2	0.5807E-02	.99	3.154E-04		-.548E+00	1.6
11	2	E	10	1	E	1036.76099	2			0.1250E-01					
37	1	E	36	2	E	1036.82235	6	.190931E-02	0.8	0.1886E-02	1.24	2.837E-04	-.255E+00		1.3
4	4	A_1	5	3	A_2	1036.88190	8			0.3488E-03					
4	4	A_2	5	3	A_1	1036.88190	8			0.3488E-03					
52	4	A_2	52	3	A_1	1036.88190	-13			0.3069E-03					
52	4	A_1	52	3	A_2	1036.88190	-13			0.3069E-03					
3	3	E	2	2	E	1036.90094	5	.101152E-01	0.3	0.1026E-01	-1.47	3.676E-04			1.0
27	7	A_2	28	6	A_1	1036.91052	-3	.296773E-02	1.0	0.1525E-02	-2.77	3.891E-04			1.0
27	7	A_1	28	6	A_2	1036.91052	-3			0.1525E-02					
51	4	A_2	51	3	A_1	1037.05057	0			0.3740E-03					
51	4	A_1	51	3	A_2	1037.05057	1			0.3740E-03					
20	1	A_1	19	0	A_2	1037.17168	4	.216029E-01	0.1	0.2173E-01	-.58	3.939E-04		-.141E+00	1.1
50	4	A_1	50	3	A_2	1037.21585	0			0.4537E-03					
50	4	A_2	50	3	A_1	1037.21585	0			0.4537E-03					
34	8	E	35	7	E	1037.33541	20			0.5833E-03					
19	6	E	20	5	E	1037.34250	-18	.281551E-02	1.3	0.2828E-02	-.43	4.533E-04			1.0
49	4	A_1	49	3	A_2	1037.37785	-5			0.5479E-03					
49	4	A_2	49	3	A_1	1037.37785	-5			0.5479E-03					
48	4	A_1	48	3	A_2	1037.53662	-7			0.6587E-03					
48	4	A_2	48	3	A_1	1037.53662	-7			0.6587E-03					
29	0	E	28	1	E	1037.54693	3	.554289E-02	2.0	0.5333E-02	3.79	1.933E-04			1.0
38	1	E	37	2	E	1037.57424	3	.168123E-02	0.5	0.1651E-02	1.81	2.267E-04			1.0
12	2	E	11	1	E	1037.60792	5	.126123E-01	0.3	0.1277E-01	-1.25	4.239E-04			1.0
11	5	E	12	4	E	1037.63515	5	.268958E-02	0.5	0.2675E-02	.53	3.245E-04	-.224E+00	-.413E+00	1.4
41	9	E	42	8	E	1037.67194	-3			0.1639E-03					
47	4	A_2	47	3	A_1	1037.69224	0			0.7882E-03					
47	4	A_1	47	3	A_2	1037.69224	0			0.7882E-03					
4	3	E	3	2	E	1037.77465	8	.106400E-01	0.1	0.1065E-01	-.14	2.829E-04	-.515E-01	-.408E+00	1.9
46	4	A_2	46	3	A_1	1037.84455	0	.198904E-02	0.7	0.9388E-03	5.60	3.489E-04	-.211E+00		1.2
46	4	A_1	46	3	A_2	1037.84455	0			0.9388E-03					
26	7	A_1	27	6	A_2	1037.87855	-23			0.1621E-02					
26	7	A_2	27	6	A_1	1037.87855	-23			0.1621E-02					
48	10	A_1	49	9	A_2	1037.92407	-6			0.3452E-04					
48	10	A_2	49	9	A_1	1037.92407	-6			0.3452E-04					
21	1	A_2	20	0	A_1	1037.97695	2	.206923E-01	0.2	0.2095E-01	-1.22	3.619E-04	.571E-01	-.366E+00	1.4
45	4	A_2	45	3	A_1	1037.99361	-1	.227391E-02	1.0	0.1113E-02	2.10	1.853E-04	.656E-01	-.368E+00	1.1
45	4	A_1	45	3	A_2	1037.99361	-1			0.1113E-02					
48	2	A_2	47	3	A_1	1038.05024	0			0.2765E-03					
48	2	A_1	47	3	A_2	1038.05414	8			0.2765E-03					
44	4	A_2	44	3	A_1	1038.13941	-3	.268169E-02	0.8	0.1314E-02	2.04	1.992E-04			1.0
44	4	A_1	44	3	A_2	1038.13941	-3			0.1314E-02					
43	4	A_2	43	3	A_1	1038.28182	-20	.336462E-02	1.2	0.1543E-02	10.44	5.021E-04			1.0
43	4	A_1	43	3	A_2	1038.28182	-20			0.1543E-02					
18	6	E	19	5	E	1038.28816	27			0.2812E-02					
39	1	E	38	2	E	1038.32244	19	.226408E-02	1.0	0.1438E-02	7.90	2.926E-04			1.0
33	8	E	34	7	E	1038.32244	-32			0.6474E-03					
30	0	E	29	1	E	1038.32918	4	.514187E-02	0.5	0.4870E-02	5.29	3.405E-04			1.0

(cont.)

J	K_a	Γ	J'	K'_a	Γ'	$\nu^{a)}$	$\delta_\nu{}^{b)}$	$S_\nu^{\rm exp}(294.45)^{c)}$	$\Delta_S{}^{d)}$	$S_\nu^{\rm calc}(294.45)^{e)}$	$\delta_S{}^{f)}$	$\Gamma_0{}^{g)}$	$\Delta_2{}^{g)}$	$\nu_{\rm VC}{}^{g)}$	$R^{h)}$
	1			2		3	4	5	6	7	8	9	10	11	12
42	4	A_2	42	3	A_1	1038.42107	-29			0.1804E-02					
42	4	A_1	42	3	A_2	1038.42107	-29			0.1804E-02					
13	2	E	12	1	E	1038.45138	4	.127678E-01	0.3	0.1293E-01	-1.30	3.729E-04		-.422E+00	1.4
41	4	A_2	41	3	A_1	1038.55712	-33	.672395E-02	0.9	0.2099E-02	3.27	5.200E-04			1.0
41	4	A_1	41	3	A_2	1038.55712	-33			0.2099E-02					
5	3	E	4	2	E	1038.64503	7	.111753E-01	0.3	0.1119E-01	-.11	2.898E-04	-.279E-01	-.332E+00	1.2
40	9	E	41	8	E	1038.67803	-12			0.1890E-03					
40	4	A_2	40	3	A_1	1038.69030	0	.480276E-02	0.3	0.2430E-02	-1.21	2.507E-04			1.0
40	4	A_1	40	3	A_2	1038.69030	0			0.2430E-02					
49	2	A_1	48	3	A_2	1038.75819	-14			0.2293E-03					
49	2	A_2	48	3	A_1	1038.76270	14			0.2293E-03					
22	1	A_1	21	0	A_2	1038.77821	3	.198787E-01	0.2	0.2006E-01	-.93	3.505E-04	-.471E-01	-.380E+00	1.4
39	4	A_2	39	3	A_1	1038.81992	0	.565031E-02	0.4	0.2801E-02	.86	3.116E-04	.128E+00	.182E+00	1.2
39	4	A_1	39	3	A_2	1038.81992	0			0.2801E-02					
59	3	E	58	4	E	1038.84401	113			0.2192E-04					
25	7	A_2	26	6	A_1	1038.84413	-2	.332104E-02	0.4	0.1710E-02	-2.95	3.132E-04	-.859E-01	-.402E+00	1.3
25	7	A_1	26	6	A_2	1038.84413	-2			0.1710E-02					
38	4	A_2	38	3	A_1	1038.94629	0	.633212E-02	0.4	0.3212E-02	-1.47	2.044E-04		-.512E+00	1.4
38	4	A_1	38	3	A_2	1038.94629	0			0.3212E-02					
37	4	A_2	37	3	A_1	1039.06941	-1	.743521E-02	1.2	0.3666E-02	1.37	3.075E-04			1.0
37	4	A_1	37	3	A_2	1039.06941	-1			0.3666E-02					
31	0	E	30	1	E	1039.10769	7	.448751E-02	0.7	0.4423E-02	1.44	3.661E-04			1.0
36	4	A_2	36	3	A_1	1039.18931	0	.840894E-02	0.4	0.4164E-02	.96	2.996E-04			1.0
36	4	A_1	36	3	A_2	1039.18931	0			0.4164E-02					
17	6	E	18	5	E	1039.23015	0	.277663E-02	0.5	0.2761E-02	.55	4.057E-04			1.0
14	2	E	13	1	E	1039.29143	4	.128793E-01	0.3	0.1300E-01	-.91	4.240E-04			1.0
35	4	A_2	35	3	A_1	1039.30600	5	.948718E-02	0.4	0.4706E-02	.79	4.017E-04			1.0
35	4	A_1	35	3	A_2	1039.30600	5			0.4706E-02					
34	4	A_2	34	3	A_1	1039.41936	0	.105662E-01	0.2	0.5292E-02	-.16	2.769E-04			1.0
34	4	A_1	34	3	A_2	1039.41936	0			0.5292E-02					
50	2	A_2	49	3	A_1	1039.46245	9			0.1891E-03					
50	2	A_1	49	3	A_2	1039.46699	0			0.1891E-03					
9	5	E	10	4	E	1039.47459	6	.184420E-02	1.0	0.1901E-02	-3.11	3.573E-04	-.369E+00	-.324E+00	1.3
60	3	E	59	4	E	1039.50609	79			0.1725E-04					
6	3	E	5	2	E	1039.51210	6	.116971E-01	0.3	0.1175E-01	-.45	3.162E-04	-.163E+00	-.339E+00	1.7
33	4	A_2	33	3	A_1	1039.52953	0	.121162E-01	0.4	0.5920E-02	2.27	3.317E-04	-.522E+00	-.246E+00	2.8
33	4	A_1	33	3	A_2	1039.52953	0			0.5920E-02					
23	1	A_2	22	0	A_1	1039.57533	-4			0.1910E-01					
32	4	A_2	32	3	A_1	1039.63647	1	.133391E-01	0.3	0.6590E-02	1.20	3.323E-04			1.0
32	4	A_1	32	3	A_2	1039.63647	1			0.6590E-02					
31	4	A_1	31	3	A_2	1039.73980	-34			0.7297E-02					
31	4	A_2	31	3	A_1	1039.73980	-34			0.7297E-02					
24	7	A_2	25	6	A_1	1039.80667	1	.474678E-02	0.5	0.1789E-02	2.00	3.357E-04	-.139E+00		1.0
24	7	A_1	25	6	A_2	1039.80667	1			0.1789E-02					
41	1	E	40	2	E	1039.80667	2			0.1074E-02					
30	4	A_2	30	3	A_1	1039.84052	-7	.165071E-01	0.4	0.8038E-02	2.62	3.984E-04			1.0
30	4	A_1	30	3	A_2	1039.84052	-7			0.8038E-02					
32	0	E	31	1	E	1039.88240	6	.408447E-02	0.2	0.3995E-02	2.20	2.694E-04	-.858E-01	-.391E+00	1.9
29	4	A_2	29	3	A_1	1039.93781	1	.173186E-01	0.2	0.8806E-02	-1.70	3.316E-04			1.0
29	4	A_1	29	3	A_2	1039.93781	1			0.8806E-02					
46	10	A_1	47	9	A_2	1039.96988	8			0.4894E-04					
46	10	A_2	47	9	A_1	1039.96988	8			0.4894E-04					
28	4	A_2	28	3	A_1	1040.03176	0	.184089E-01	0.2	0.9596E-02	-4.26	2.973E-04	-.124E+00	-.389E+00	1.7
28	4	A_1	28	3	A_2	1040.03176	0			0.9596E-02					
27	4	A_2	27	3	A_1	1040.12248	-1	.201799E-01	0.2	0.1040E-01	-3.07	3.376E-04			1.0
27	4	A_1	27	3	A_2	1040.12248	-1			0.1040E-01					
15	2	E	14	1	E	1040.12807	7	.128583E-01	0.4	0.1296E-01	-.81	4.181E-04			1.0
51	2	A_1	50	3	A_2	1040.16242	13			0.1553E-03					
16	6	E	17	5	E	1040.16943	0	.266149E-02	1.4	0.2674E-02	-.46	4.429E-04	-.350E+00		1.1
26	4	A_2	26	3	A_1	1040.20998	1	.216369E-01	0.3	0.1121E-01	-3.59	3.043E-04	-.193E+00	-.355E+00	1.4
26	4	A_1	26	3	A_2	1040.20998	1			0.1121E-01					
25	4	A_1	25	3	A_2	1040.29435	13			0.1201E-01					
25	4	A_2	25	3	A_1	1040.29435	13			0.1201E-01					
24	1	A_1	23	0	A_2	1040.36854	4	.182805E-01	0.5	0.1808E-01	1.10	3.573E-04			1.0
24	4	A_2	24	3	A_1	1040.37541	18	.367159E-01	0.4	0.1279E-01	-3.14	4.423E-04			1.0
24	4	A_1	24	3	A_2	1040.37541	18			0.1279E-01					
7	3	E	6	2	E	1040.37541	-37			0.1229E-01					
8	5	E	9	4	E	1040.38948	-13	.149107E-02	1.7	0.1472E-02	1.30	2.879E-04			1.0
23	4	A_2	23	3	A_1	1040.45301	1	.265153E-01	0.1	0.1354E-01	-2.14	3.802E-04			1.0
23	4	A_1	23	3	A_2	1040.45301	1			0.1354E-01					
22	4	A_2	22	3	A_1	1040.52753	1	.279694E-01	0.2	0.1425E-01	-1.87	4.074E-04	-.167E+00	.320E+00	1.6
22	4	A_1	22	3	A_2	1040.52753	1			0.1425E-01					
21	4	A_1	21	3	A_2	1040.59892	11			0.1489E-01					
21	4	A_2	21	3	A_1	1040.59892	11			0.1489E-01					
33	0	E	32	1	E	1040.65330	4	.366047E-02	4.2	0.3588E-02	1.97	1.165E-04			1.0
20	4	A_2	20	3	A_1	1040.66690	4	.299596E-01	0.4	0.1546E-01	-3.23	3.727E-04			1.0
20	4	A_1	20	3	A_2	1040.66690	4			0.1546E-01					
38	9	E	39	8	E	1040.68232	-22			0.2470E-03					
19	4	A_2	19	3	A_1	1040.73168	2	.308782E-01	0.1	0.1595E-01	-3.28	3.944E-04			1.0
19	4	A_1	19	3	A_2	1040.73168	2			0.1595E-01					
23	7	A_2	24	6	A_1	1040.76627	-3	.356051E-02	1.0	0.1856E-02	-4.26	2.628E-04			1.0
23	7	A_1	24	6	A_2	1040.76627	-3			0.1856E-02					

(cont.)

J	K_a	Γ	J'	K'_a	Γ'	$\nu^{a)}$	$\delta_\nu{}^{b)}$	$S_\nu^{\rm exp}(294.45)^{c)}$	$\Delta_S{}^{d)}$	$S_\nu^{\rm calc}(294.45)^{e)}$	$\delta_S{}^{f)}$	$\Gamma_0{}^{g)}$	$\Delta_2{}^{g)}$	$\nu_{\rm VC}{}^{g)}$	$R^{h)}$
1			2			3	4	5	6	7	8	9	10	11	12
18	4	A_1	18	3	A_2	1040.79334	11			0.1632E-01					
18	4	A_2	18	3	A_1	1040.79334	11			0.1632E-01					
17	4	A_2	17	3	A_1	1040.85157	1	.329431E-01	0.2	0.1658E-01	-.64	3.902E-04			1.0
17	4	A_1	17	3	A_2	1040.85157	1			0.1658E-01					
52	2	A_1	51	3	A_2	1040.86367	4			0.1269E-03					
16	4	A_2	16	3	A_1	1040.90667	2	.325386E-01	0.2	0.1670E-01	-2.64	3.968E-04			1.0
16	4	A_1	16	3	A_2	1040.90667	2			0.1670E-01					
15	4	A_1	15	3	A_2	1040.95855	6			0.1667E-01					
15	4	A_2	15	3	A_1	1040.95855	6			0.1667E-01					
14	4	A_2	14	3	A_1	1041.00712	2	.320887E-01	0.2	0.1648E-01	-2.72	4.305E-04			1.3
14	4	A_1	14	3	A_2	1041.00712	2			0.1648E-01					
13	4	A_2	13	3	A_1	1041.05249	2	.327278E-01	0.2	0.1612E-01	1.49	4.612E-04			1.0
13	4	A_1	13	3	A_2	1041.05249	2			0.1612E-01					
12	4	A_2	12	3	A_1	1041.09462	3	.309124E-01	0.2	0.1558E-01	-.77	4.095E-04			1.0
12	4	A_1	12	3	A_2	1041.09462	3			0.1558E-01					
15	6	E	16	5	E	1041.10574	3	.262797E-02	2.2	0.2548E-02	3.04	6.613E-04			1.8
11	4	A_2	11	3	A_1	1041.13352	4	.291531E-01	0.2	0.1484E-01	-1.81	3.732E-04			1.0
11	4	A_1	11	3	A_2	1041.13352	4			0.1484E-01					
25	1	A_2	24	0	A_1	1041.15756	3	.172735E-01	0.2	0.1701E-01	1.51	3.690E-04			1.0
10	4	A_2	10	3	A_1	1041.16917	5	.274566E-01	0.2	0.1391E-01	-1.29	3.793E-04			1.0
10	4	A_1	10	3	A_2	1041.16917	5			0.1391E-01					
9	4	A_2	9	3	A_1	1041.20157	4	.253504E-01	0.2	0.1276E-01	-.69	3.704E-04			1.0
9	4	A_1	9	3	A_2	1041.20157	4			0.1276E-01					
8	4	A_2	8	3	A_1	1041.23074	5	.222043E-01	0.5	0.1140E-01	-2.70	3.302E-04			1.0
8	4	A_1	8	3	A_2	1041.23074	5			0.1140E-01					
8	3	E	7	2	E	1041.23592	-26			0.1277E-01					
7	4	A_2	7	3	A_1	1041.25665	3	.190941E-01	0.5	0.9809E-02	-2.74	3.068E-04			1.0
7	4	A_1	7	3	A_2	1041.25665	3			0.9809E-02					
30	8	E	31	7	E	1041.26904	16			0.8522E-03					
30	8	E	31	7	E	1041.26882	-6			0.8522E-03					
6	4	A_2	6	3	A_1	1041.27934	4	.153925E-01	0.8	0.7958E-02	-3.40	4.310E-04			1.0
6	4	A_1	6	3	A_2	1041.27934	4			0.7958E-02					
5	4	A_1	5	3	A_2	1041.29881	6	.126344E-01	0.5	0.5800E-02	1.50	5.697E-04	.325E+00	.509E+00	1.3
5	4	A_2	5	3	A_1	1041.29881	6			0.5800E-02					
7	5	E	8	4	E	1041.29881	-278			0.1034E-02					
4	4	A_2	4	3	A_1	1041.31500	5	.601187E-02	0.5	0.3231E-02	-7.48	4.020E-04	-.284E+00		1.2
4	4	A_1	4	3	A_2	1041.31500	5			0.3231E-02					
34	0	E	33	1	E	1041.42040	2	.331288E-02	0.7	0.3207E-02	3.21	3.877E-04	-.352E+00	.226E+00	1.3
53	2	A_1	52	3	A_2	1041.55023	38			0.1032E-03					
53	2	A_2	52	3	A_1	1041.55532	-46			0.1032E-03					
37	9	E	38	8	E	1041.68079	6			0.2800E-03					
22	7	A_2	23	6	A_1	1041.72304	0	.365580E-02	0.4	0.1909E-02	-4.41	3.191E-04	-.222E+00	-.382E+00	1.4
22	7	A_1	23	6	A_2	1041.72304	0			0.1909E-02					
17	2	E	16	1	E	1041.79087	4	.125112E-01	7.1	0.1262E-01	-.88	4.245E-04			1.0
26	1	A_1	25	0	A_2	1041.94252	5	.160282E-01	0.3	0.1592E-01	.69	3.181E-04	-.242E+00	-.393E+00	1.8
44	1	E	43	2	E	1042.00396	21			0.6674E-03					
14	6	E	15	5	E	1042.03899	0	.231850E-02	1.6	0.2383E-02	-2.79	5.334E-04			1.0
9	3	E	8	2	E	1042.09333	11	.130778E-01	0.2	0.1319E-01	-.82	4.297E-04		-.294E+00	1.2
64	3	E	63	4	E	1042.11386	165			0.6318E-05					
62	5	E	62	4	E	1042.15415	-5			0.2853E-04					
35	0	E	34	1	E	1042.18372	5	.285387E-02	0.6	0.2850E-02	.13	3.327E-04			1.0
6	5	E	7	4	E	1042.21029	-15	.587270E-03	1.0	0.6118E-03	-4.19	5.572E-04	-.728E+00		1.5
54	2	A_2	53	3	A_1	1042.23772	28			0.8349E-04					
51	11	E	52	10	E	1042.24510	-34			0.1241E-04					
51	11	E	52	10	E	1042.24521	-23			0.1241E-04					
29	8	E	30	7	E	1042.24521	-15	.963695E-03	2.0	0.9216E-03	4.37	3.499E-04	-.536E+00	-.548E+00	1.2
60	5	E	60	4	E	1042.55327	-4			0.4612E-04					
18	2	E	17	1	E	1042.61707	4	.119089E-01	0.3	0.1233E-01	-3.53	4.205E-04	-.368E+00	-.188E+00	1.8
36	9	E	37	8	E	1042.67683	61	.417396E-02	0.6	0.3155E-03	-.69	4.012E-04	-.218E+00		1.3
21	7	A_1	22	6	A_2	1042.67683	-6			0.1944E-02					
21	7	A_2	22	6	A_1	1042.67683	-6			0.1944E-02					
27	1	A_2	26	0	A_1	1042.72332	3	.151498E-01	0.2	0.1481E-01	2.24	3.731E-04			1.0
45	1	E	44	2	E	1042.72765	-52			0.5640E-03					
59	5	E	59	4	E	1042.74799	2			0.5826E-04					
55	2	A_1	54	3	A_2	1042.92102	13			0.6725E-04					
55	2	A_2	54	3	A_1	1042.92771	7			0.6725E-04					
36	0	E	35	1	E	1042.94333	22			0.2520E-02					
10	3	E	9	2	E	1042.94692	4	.133698E-01	0.3	0.1352E-01	-1.09	3.864E-04			1.0
13	6	E	14	5	E	1042.96930	4	.217305E-02	1.2	0.2180E-02	-.32	4.432E-04	-.192E+00		1.1
43	10	A_2	44	9	A_1	1043.01893	-7			0.7951E-04					
43	10	A_1	44	9	A_2	1043.01893	-7			0.7951E-04					
5	5	E	6	4	E	1043.11575	-41			0.2455E-03					
57	5	E	57	4	E	1043.12753	3			0.9172E-04					
28	8	E	29	7	E	1043.21901	-2			0.9897E-03					
19	2	E	18	1	E	1043.43976	3	.116224E-01	0.2	0.1197E-01	-2.96	4.282E-04			1.0
46	1	E	45	2	E	1043.44871	11			0.4742E-03					
55	5	E	55	4	E	1043.49368	-31			0.1419E-03					
28	1	A_1	27	0	A_2	1043.50001	3	.138569E-01	0.2	0.1370E-01	1.10	3.142E-04		-.269E+00	1.2
56	2	A_2	55	3	A_1	1043.60017	-1			0.5391E-04					
56	2	A_1	55	3	A_2	1043.60729	-1			0.5391E-04					
20	7	A_2	21	6	A_1	1043.62784	3	.403394E-02	0.6	0.1958E-02	2.90	4.933E-04			1.0
20	7	A_1	21	6	A_2	1043.62784	3			0.1958E-02					

(cont.)

J	K_a	Γ	J'	K'_a	Γ'	$\nu^{a)}$	$\delta_\nu{}^{b)}$	$S_\nu^{\exp}(294.45)^{c)}$	$\Delta_S{}^{d)}$	$S_\nu^{\mathrm{calc}}(294.45)^{e)}$	$\delta_S{}^{f)}$	$\Gamma_0{}^{g)}$	$\Delta_2{}^{g)}$	$\nu_{\mathrm{VC}}{}^{g)}$	$R^{h)}$
	1			2		3	4	5	6	7	8	9	10	11	12
35	9	E	36	8	E	1043.66923	22			0.3535E-03					
37	0	E	36	1	E	1043.69874	5	.228110E-02	0.7	0.2217E-02	2.80	2.950E-04	-.259E+00	-.115E+00	1.3
11	3	E	10	2	E	1043.79721	5	.135505E-01	0.2	0.1376E-01	-1.51	3.970E-04			1.0
53	5	E	53	4	E	1043.84763	19			0.2155E-03					
12	6	E	13	5	E	1043.89648	0	.189058E-02	0.8	0.1941E-02	-2.65	3.522E-04			1.0
52	5	E	52	4	E	1044.01924	-5			0.2638E-03					
42	10	A_2	43	9	A_1	1044.03017	-3			0.9249E-04					
42	10	A_1	43	9	A_2	1044.03017	-3			0.9249E-04					
47	1	E	46	2	E	1044.16500	0			0.3968E-03					
27	8	E	28	7	E	1044.18971	-18			0.1055E-02					
20	2	E	19	1	E	1044.25895	2	.112712E-01	0.3	0.1154E-01	-2.39	4.431E-04			1.0
29	1	A_2	28	0	A_1	1044.27256	3	.134947E-01	0.2	0.1261E-01	6.53	3.647E-04			1.0
49	11	E	50	10	E	1044.30561	-19			0.1809E-04					
50	5	E	50	4	E	1044.35352	31			0.3901E-03					
38	0	E	37	1	E	1044.45040	2	.203182E-02	0.9	0.1941E-02	4.49	3.937E-04			1.0
49	5	E	49	4	E	1044.51525	-4			0.4711E-03					
19	7	A_2	20	6	A_1	1044.57580	0	.401890E-02	0.5	0.1951E-02	2.91	4.559E-04	-.577E-01	-.314E+00	1.1
19	7	A_1	20	6	A_2	1044.57580	0			0.1951E-02					
12	3	E	11	2	E	1044.64408	4	.139832E-01	0.2	0.1390E-01	.58	3.952E-04			1.0
34	9	E	35	8	E	1044.65898	-10			0.3937E-03					
48	5	E	48	4	E	1044.67409	-3			0.5664E-03					
11	6	E	12	5	E	1044.82067	1	.175887E-02	0.6	0.1669E-02	5.13	4.700E-04			1.0
47	5	E	47	4	E	1044.82966	-4			0.6778E-03					
4	4	A_2	3	3	A_1	1044.86159	5	.259564E-01	0.2	0.1285E-01	1.02	3.294E-04	-.966E-01		1.1
4	4	A_1	3	3	A_2	1044.86159	5			0.1285E-01					
48	1	E	47	2	E	1044.87736	-1			0.3304E-03					
58	2	A_1	57	3	A_2	1044.95395	-1			0.3416E-04					
41	10	A_2	42	9	A_1	1045.03874	-3			0.1070E-03					
41	10	A_1	42	9	A_2	1045.03874	-3			0.1070E-03					
30	1	A_1	29	0	A_2	1045.04076	-16			0.1155E-01					
21	2	E	20	1	E	1045.07464	4	.108931E-01	0.2	0.1106E-01	-1.56	3.798E-04	-.133E+00	-.219E+00	1.4
45	5	E	45	4	E	1045.13110	-1	.104843E-02	1.3	0.9573E-03	8.69	3.472E-04	-.277E+00		1.1
26	8	E	27	7	E	1045.15793	2	.114521E-02	0.8	0.1116E-02	2.54	3.771E-04			1.0
39	0	E	38	1	E	1045.19822	5	.178575E-02	0.8	0.1690E-02	5.37	1.957E-04			1.0
44	5	E	44	4	E	1045.27692	-2	.122437E-02	0.8	0.1130E-02	7.73	3.184E-04	-.367E+00		1.5
48	11	E	49	10	E	1045.33233	14			0.2168E-04					
43	5	E	43	4	E	1045.41950	-3	.132497E-02	0.9	0.1327E-02	-.15	2.608E-04	-.232E+00		1.2
13	3	E	12	2	E	1045.48755	5	.139893E-01	0.2	0.1396E-01	.24	3.905E-04	-.565E-01	-.175E+00	1.2
18	7	A_2	19	6	A_1	1045.52086	2	.391861E-02	0.4	0.1919E-02	2.07	4.287E-04			1.0
18	7	A_1	19	6	A_2	1045.52086	2			0.1919E-02					
42	5	E	42	4	E	1045.55884	-3	.160191E-02	0.6	0.1551E-02	3.16	2.840E-04			1.0
49	1	E	48	2	E	1045.58561	-7			0.2738E-03					
33	9	E	34	8	E	1045.64640	-1			0.4358E-03					
41	5	E	41	4	E	1045.69495	0	.191669E-02	1.4	0.1805E-02	5.83	3.404E-04			1.0
5	4	A_2	4	3	A_1	1045.73192	5	.262661E-01	0.1	0.1301E-01	.95	3.189E-04			1.0
5	4	A_1	4	3	A_2	1045.73192	5			0.1301E-01					
10	6	E	11	5	E	1045.74178	0	.136952E-02	2.4	0.1370E-02	-.04	5.323E-04			1.0
31	1	A_2	30	0	A_1	1045.80527	14			0.1052E-01					
40	5	E	40	4	E	1045.82800	20			0.2090E-02					
22	2	E	21	1	E	1045.88676	2	.102726E-01	0.2	0.1054E-01	-2.62	3.429E-04	-.128E+00	-.443E+00	1.9
40	0	E	39	1	E	1045.94210	7	.148965E-02	0.9	0.1464E-02	1.70	3.172E-04			1.0
39	5	E	39	4	E	1045.95738	-2	.239262E-02	0.8	0.2409E-02	-.67	3.033E-04			1.0
38	5	E	38	4	E	1046.08375	0	.274264E-02	0.7	0.2762E-02	-.71	2.741E-04	-.179E+00		1.1
25	8	E	26	7	E	1046.12305	-3	.120476E-02	1.1	0.1171E-02	2.78	5.025E-04	-.189E+00	.124E+01	1.1
37	5	E	37	4	E	1046.20690	4	.336082E-02	1.1	0.3152E-02	6.21	1.401E-04			1.0
50	1	E	49	2	E	1046.29011	20			0.2258E-03					
36	5	E	36	4	E	1046.32740	67	.177772E-01	0.3	0.3579E-02	1.59	4.231E-04	-.349E+00	-.549E+00	2.1
14	3	E	13	2	E	1046.32740	-14			0.1391E-01					
47	11	E	48	10	E	1046.35583	-21			0.2585E-04					
35	5	E	35	4	E	1046.44333	-2	.406702E-02	0.5	0.4045E-02	.55	2.763E-04	-.249E+00	-.168E+00	1.3
17	7	A_2	18	6	A_1	1046.46284	-9			0.1860E-02					
17	7	A_1	18	6	A_2	1046.46284	-9			0.1860E-02					
34	5	E	34	4	E	1046.55669	-4	.448932E-02	0.8	0.4547E-02	-1.28	2.678E-04			1.0
32	1	A_1	31	0	A_2	1046.56521	5	.953200E-02	0.4	0.9528E-02	.04	3.334E-04			1.0
6	4	A_2	5	3	A_1	1046.59895	5	.269062E-01	0.2	0.1328E-01	1.25	3.237E-04			1.0
6	4	A_1	5	3	A_2	1046.59895	5			0.1328E-01					
32	9	E	33	8	E	1046.63127	28			0.4792E-03					
9	6	E	10	5	E	1046.65990	8	.100204E-02	3.1	0.1054E-02	-5.22	3.845E-04			1.0
33	5	E	33	4	E	1046.66686	-1	.507701E-02	0.7	0.5086E-02	-.17	3.388E-04			1.0
41	0	E	40	1	E	1046.68193	-2	.135667E-02	2.0	0.1262E-02	6.95	3.595E-04			1.0
23	2	E	22	1	E	1046.69536	4	.988967E-02	0.3	0.9987E-02	-.98	3.889E-04			1.0
32	5	E	32	4	E	1046.77376	0	.555119E-02	0.4	0.5659E-02	-1.95	2.725E-04		-.406E+00	1.3
31	5	E	31	4	E	1046.87740	-2	.631196E-02	0.3	0.6264E-02	.75	3.107E-04	-.201E+00	-.210E+00	1.9
30	5	E	30	4	E	1046.97780	-3	.704004E-02	0.3	0.6898E-02	2.02	3.655E-04			1.0
51	1	E	50	2	E	1046.98983	-22			0.1854E-03					
39	10	A_2	40	9	A_1	1047.04787	-12			0.1409E-03					
39	10	A_1	40	9	A_2	1047.04787	-12			0.1409E-03					
29	5	E	29	4	E	1047.07496	-4	.779352E-02	0.6	0.7554E-02	3.08	3.431E-04			1.0
24	8	E	25	7	E	1047.08528	-12	.121724E-02	2.3	0.1219E-02	-.10	4.881E-04			1.0
15	3	E	14	2	E	1047.16416	3	.137446E-01	0.4	0.1378E-01	-.29	4.275E-04			1.0
28	5	E	28	4	E	1047.16887	-6	.818179E-02	0.5	0.8227E-02	-.55	3.340E-04			1.0
27	5	E	27	4	E	1047.25962	0	.900279E-02	0.3	0.8910E-02	1.03	3.441E-04			1.0

(cont.)

J	K_a	Γ	J'	K'_a	Γ'	$\nu^{a)}$	$\delta_\nu{}^{b)}$	$S_\nu^{\rm exp}(294.45)^{c)}$	$\Delta_S{}^{d)}$	$S_\nu^{\rm calc}(294.45)^{e)}$	$\delta_S{}^{f)}$	$\Gamma_0{}^{g)}$	$\Delta_2{}^{g)}$	$\nu_{\rm VC}{}^{g)}$	$R^{h)}$
	1			2		3	4	5	6	7	8	9	10	11	12
33	1	A_2	32	0	A_1	1047.32102	4	.874264E-02	0.7	0.8589E-02	1.76	3.434E-04			1.0
26	5	E	26	4	E	1047.34704	-2	.991335E-02	0.2	0.9595E-02	3.21	3.831E-04	-.131E+00	.153E+00	1.2
16	7	A_2	17	6	A_1	1047.40206	2	.371394E-02	0.6	0.1774E-02	4.46	5.071E-04	-.467E+00		1.5
16	7	A_1	17	6	A_2	1047.40206	2			0.1774E-02					
42	0	E	41	1	E	1047.41784	-6			0.1083E-02					
25	5	E	25	4	E	1047.43126	-1	.104087E-01	0.4	0.1027E-01	1.31	3.424E-04	-.378E+00	-.219E+00	1.6
7	4	A_2	6	3	A_1	1047.46261	2	.272973E-01	0.4	0.1359E-01	.45	3.516E-04			1.0
7	4	A_1	6	3	A_2	1047.46261	2			0.1359E-01					
24	2	E	23	1	E	1047.50035	0	.932208E-02	0.4	0.9407E-02	-.91	4.496E-04			1.0
24	5	E	24	4	E	1047.51224	0	.109842E-01	0.3	0.1093E-01	.48	3.883E-04			1.0
8	6	E	9	5	E	1047.57482	6	.765300E-03	5.9	0.7345E-03	4.03	1.380E-04			1.0
23	5	E	23	4	E	1047.58997	0	.115812E-01	0.4	0.1156E-01	.18	4.062E-04			1.0
31	9	E	32	8	E	1047.61278	-4			0.5235E-03					
22	5	E	22	4	E	1047.66445	-1	.118936E-01	0.4	0.1215E-01	-2.13	3.244E-04	-.317E-01	-.457E+00	1.3
52	1	E	51	2	E	1047.68597	-10			0.1514E-03					
21	5	E	21	4	E	1047.73569	-2	.126130E-01	0.2	0.1268E-01	-.53	4.107E-04	-.328E+00	-.264E+00	2.6
20	5	E	20	4	E	1047.80374	3	.129703E-01	3.7	0.1314E-01	-1.33	2.042E-04			1.0
19	5	E	19	4	E	1047.86850	2	.129312E-01	0.5	0.1352E-01	-4.59	3.837E-04			1.0
18	5	E	18	4	E	1047.93002	1	.133013E-01	0.2	0.1381E-01	-3.82	4.097E-04			1.0
17	5	E	17	4	E	1047.98831	0	.136902E-01	0.5	0.1398E-01	-2.15	4.106E-04			1.0
16	3	E	15	2	E	1047.99727	1	.135681E-01	0.5	0.1357E-01	-.01	4.465E-04			1.0
16	5	E	16	4	E	1048.04350	14	.145720E-01	0.4	0.1404E-01	-4.94	6.616E-04	.132E+01	-.141E+00	2.7
23	8	E	24	7	E	1048.04350	-134			0.1256E-02					
34	1	A_1	33	0	A_2	1048.07282	24			0.7703E-02					
15	5	E	15	4	E	1048.09519	2	.134980E-01	0.2	0.1395E-01	-3.36	4.195E-04			1.0
14	5	E	14	4	E	1048.14376	1	.149281E-01	0.5	0.1372E-01	1.91	4.959E-04			1.0
43	0	E	42	1	E	1048.14993	5			0.9246E-03					
13	5	E	13	4	E	1048.18910	1	.131895E-01	0.3	0.1333E-01	-1.03	3.353E-04	.724E-01	-.374E+00	1.1
12	5	E	12	4	E	1048.23119	1	.124111E-01	0.2	0.1276E-01	-2.83	3.941E-04			1.0
11	5	E	11	4	E	1048.26977	-27	.128644E-01	0.6	0.1202E-01	6.58	6.522E-04	-.370E+00		1.1
25	2	E	24	1	E	1048.30186	6	.916404E-02	0.4	0.8812E-02	3.84	4.164E-04			1.0
10	5	E	10	4	E	1048.30574	8	.113877E-01	0.4	0.1108E-01	2.70	4.164E-04			1.0
8	4	A_2	7	3	A_1	1048.32297	2	.283518E-01	0.3	0.1387E-01	2.17	3.621E-04	-.331E+00		1.9
8	4	A_1	7	3	A_2	1048.32297	2			0.1387E-01					
15	7	A_2	16	6	A_1	1048.33809	-6	.135704E-01	0.5	0.1660E-02	2.30	3.667E-04	-.398E+00	-.192E+00	2.0
15	7	A_1	16	6	A_2	1048.33809	-6			0.1660E-02					
9	5	E	9	4	E	1048.33809	4			0.9938E-02					
8	5	E	8	4	E	1048.36722	3	.878120E-02	0.3	0.8575E-02	2.35	3.343E-04	-.235E+00	-.363E+00	1.8
53	1	E	52	2	E	1048.37795	-1			0.1231E-03					
7	5	E	7	4	E	1048.39311	1	.723360E-02	0.3	0.6965E-02	3.72	3.233E-04	-.248E+00	-.305E+00	1.9
6	5	E	6	4	E	1048.41575	-2	.526527E-02	0.5	0.5065E-02	3.81	4.862E-04			1.0
5	5	E	5	4	E	1048.43523	4	.283535E-02	0.9	0.2797E-02	1.36	3.859E-04	-.178E+00		1.1
7	6	E	8	5	E	1048.48670	10			0.4299E-03					
30	9	E	31	8	E	1048.59174	-12			0.5680E-03					
35	1	A_2	34	0	A_1	1048.82002	7	.707875E-02	0.5	0.6874E-02	2.89	2.803E-04			1.0
17	3	E	16	2	E	1048.82690	-1	.132655E-01	0.3	0.1327E-01	-.07	4.294E-04			1.0
44	0	E	43	1	E	1048.87788	4			0.7855E-03					
22	8	E	23	7	E	1049.00135	-4	.128839E-02	1.0	0.1282E-02	.48	4.283E-04	-.226E+00		1.1
37	10	A_1	38	9	A_2	1049.04624	-32			0.1813E-03					
37	10	A_2	38	9	A_1	1049.04624	-32			0.1813E-03					
54	1	E	53	2	E	1049.06572	2			0.9956E-04					
26	2	E	25	1	E	1049.09966	-1	.812949E-02	0.6	0.8209E-02	-.97	2.975E-04			1.0
9	4	A_2	8	3	A_1	1049.17997	2	.288209E-01	0.2	0.1410E-01	2.13	3.786E-04			1.0
9	4	A_1	8	3	A_2	1049.17997	2			0.1410E-01					
14	7	A_2	15	6	A_1	1049.27126	-1	.302059E-02	0.4	0.1518E-02	-.53	3.491E-04	-.200E+00	-.309E+00	1.7
14	7	A_1	15	6	A_2	1049.27126	-1		0.4	0.1518E-02		3.491E-04	-.200E+00	-.309E+00	1.7
6	6	E	7	5	E	1049.39478	-54			0.1697E-03					
44	11	E	45	10	E	1049.41149	-71			0.4247E-04					
61	6	E	61	5	E	1049.54056	-4			0.3002E-04					
36	1	A_1	35	0	A_2	1049.56308	0	.630985E-02	0.2	0.6104E-02	3.27	3.543E-04			1.0
45	0	E	44	1	E	1049.60186	8			0.6641E-03					
18	3	E	17	2	E	1049.65309	1	.128665E-01	0.3	0.1291E-01	-.32	3.862E-04	-.445E-01	-.298E+00	1.2
27	2	E	26	1	E	1049.89352	-41			0.7606E-02					
21	8	E	22	7	E	1049.95501	-2	.126718E-02	0.8	0.1295E-02	-2.19	4.109E-04	-.665E+00	.295E+00	1.5
10	4	A_2	9	3	A_1	1050.03360	3	.286430E-01	0.2	0.1427E-01	.33	3.846E-04	-.143E+00	-.133E+00	1.4
10	4	A_1	9	3	A_2	1050.03360	3			0.1427E-01					
36	10	A_1	37	9	A_2	1050.04181	0			0.2038E-03					
36	10	A_2	37	9	A_1	1050.04181	0			0.2038E-03					
58	6	E	58	5	E	1050.12480	12			0.6050E-04					
13	7	A_2	14	6	A_1	1050.20132	-4	.274068E-02	0.7	0.1350E-02	1.45	4.536E-04			1.0
13	7	A_1	14	6	A_2	1050.20132	-4			0.1350E-02					
37	1	A_2	36	0	A_1	1050.30197	4	.554975E-02	0.6	0.5393E-02	2.82	2.756E-04			1.0
57	6	E	57	5	E	1050.31262	-22			0.7575E-04					
46	0	E	45	1	E	1050.32164	-3			0.5587E-03					
19	3	E	18	2	E	1050.47577	2	.125506E-01	0.2	0.1248E-01	.59	3.907E-04	-.462E-01	-.268E+00	1.2
56	6	E	56	5	E	1050.49757	-15			0.9442E-04					
28	9	E	29	8	E	1050.54151	-6			0.6544E-03					
28	2	E	27	1	E	1050.68461	3	.708826E-02	0.3	0.7009E-02	1.12	3.540E-04			1.0
54	6	E	54	5	E	1050.85767	-5			0.1447E-03					
11	4	A_2	10	3	A_1	1050.88382	1	.286143E-01	0.2	0.1437E-01	-.45	3.620E-04	.527E-01	-.862E-01	1.1
11	4	A_1	10	3	A_2	1050.88382	1			0.1437E-01					
20	8	E	21	7	E	1050.90577	1	.126134E-02	1.1	0.1293E-02	-2.47	4.349E-04			1.0

(cont.)

J	K_a	Γ	J'	K'_a	Γ'	$\nu^{a)}$	$\delta_\nu{}^{b)}$	$S_\nu^{\rm exp}(294.45)^{c)}$	$\Delta_S{}^{d)}$	$S_\nu^{\rm calc}(294.45)^{e)}$	$\delta_S{}^{f)}$	$\Gamma_0{}^{g)}$	$\Delta_2{}^{g)}$	$\nu_{\rm VC}{}^{g)}$	$R^{h)}$
1			2			3	4	5	6	7	8	9	10	11	12
38	1	A_1	37	0	A_2	1051.03662	11	.469916E-02	0.3	0.4743E-02	-.92	5.368E-04	-.611E+00	.380E+00	2.4
57	1	E	56	2	E	1051.10381	1			0.5130E-04					
12	7	A_2	13	6	A_1	1051.12843	1	.237743E-02	0.4	0.1159E-02	2.48	4.498E-04		-.346E+00	1.1
12	7	A_1	13	6	A_2	1051.12843	1			0.1159E-02					
52	6	E	52	5	E	1051.20480	14			0.2179E-03					
20	3	E	19	2	E	1051.29494	4	.120464E-01	0.2	0.1199E-01	.46	3.820E-04	-.333E-01	-.425E+00	1.5
51	6	E	51	5	E	1051.37322	-3			0.2656E-03					
29	2	E	28	1	E	1051.47161	0	.651515E-02	0.3	0.6425E-02	1.38	3.361E-04	-.664E-01	-.162E+00	1.1
27	9	E	28	8	E	1051.51230	9	.685800E-03	1.6	0.6943E-03	-1.24	4.601E-04			1.0
50	6	E	50	5	E	1051.53853	-5			0.3222E-03					
49	6	E	49	5	E	1051.70055	-10			0.3891E-03					
12	4	A_2	11	3	A_1	1051.73066	1	.285917E-01	0.1	0.1439E-01	-.66	3.625E-04	-.145E+00	-.357E+00	1.4
12	4	A_1	11	3	A_2	1051.73066	1			0.1439E-01					
48	0	E	47	1	E	1051.74920	-4			0.3899E-03					
39	1	A_2	38	0	A_1	1051.76684	4	.435268E-02	0.3	0.4150E-02	4.66	2.646E-04	-.157E+00	-.173E+00	1.2
19	8	E	20	7	E	1051.85345	-11	.126652E-02	0.9	0.1274E-02	-.55	4.492E-04			1.0
48	6	E	48	5	E	1051.85931	-15			0.4678E-03					
47	6	E	47	5	E	1052.01494	-9			0.5597E-03					
34	10	A_1	35	9	A_2	1052.02408	-11			0.2530E-03					
34	10	A_2	35	9	A_1	1052.02408	-11			0.2530E-03					
11	7	A_2	12	6	A_1	1052.05241	-3	.190006E-02	0.6	0.9494E-03	.07	4.574E-04	-.310E+00		1.2
11	7	A_1	12	6	A_2	1052.05241	-3			0.9494E-03					
21	3	E	20	2	E	1052.11054	1	.113396E-01	0.2	0.1146E-01	-1.05	3.822E-04	-.166E+00	-.310E+00	2.1
46	6	E	46	5	E	1052.16725	-9	.666376E-03	0.8	0.6667E-03	-.05	3.421E-04			1.0
30	2	E	29	1	E	1052.25499	0	.581870E-02	0.5	0.5860E-02	-.70	2.531E-04		-.540E+00	1.4
45	6	E	45	5	E	1052.31640	0	.861522E-03	1.7	0.7904E-03	8.26	4.307E-04	.264E+00	-.412E+00	1.1
59	1	E	58	2	E	1052.44172	31			0.3220E-04					
49	0	E	48	1	E	1052.45692	5			0.3233E-03					
44	6	E	44	5	E	1052.46210	-11	.941474E-03	1.3	0.9327E-03	.94	2.134E-04			1.0
26	9	E	27	8	E	1052.47999	-2	.735090E-03	1.1	0.7307E-03	.60	3.348E-04			1.0
40	1	A_1	39	0	A_2	1052.49284	6	.372922E-02	1.2	0.3614E-02	3.09	2.511E-04			1.0
13	4	A_2	12	3	A_1	1052.57409	1	.288872E-01	0.2	0.1433E-01	.80	3.693E-04			1.0
13	4	A_1	12	3	A_2	1052.57409	1			0.1433E-01					
43	6	E	43	5	E	1052.60472	-4	.111883E-02	0.8	0.1095E-02	2.10	2.293E-04			1.0
42	6	E	42	5	E	1052.74410	3	.135225E-02	1.0	0.1280E-02	5.31	4.615E-04	.225E+00	.421E+00	1.1
18	8	E	19	7	E	1052.79846	5			0.1237E-02					
5	5	E	4	4	E	1052.86789	3	.142334E-01	0.2	0.1412E-01	.78	3.427E-04			1.0
41	6	E	41	5	E	1052.88006	-7	.154949E-02	2.0	0.1490E-02	3.87	2.873E-04			1.0
22	3	E	21	2	E	1052.92264	3	.108313E-01	0.6	0.1089E-01	-.53	3.692E-04			1.0
10	7	A_2	11	6	A_1	1052.97345	6	.136909E-02	1.0	0.7277E-03	-6.30	6.153E-04	.267E+00		1.1
10	7	A_1	11	6	A_2	1052.97345	6			0.7277E-03					
40	6	E	40	5	E	1053.01267	-28	.221312E-02	1.7	0.1724E-02	-3.15	6.386E-04			1.0
33	10	A_1	34	9	A_2	1053.01267	139			0.2791E-03					
33	10	A_2	34	9	A_1	1053.01267	139			0.2791E-03					
31	2	E	30	1	E	1053.03462	-11	.533865E-02	0.4	0.5316E-02	.42	4.599E-04			1.0
60	1	E	59	2	E	1053.10401	19			0.2533E-04					
39	6	E	39	5	E	1053.14247	-4	.205837E-02	0.4	0.1987E-02	3.47	2.807E-04			1.0
50	0	E	49	1	E	1053.16034	-4			0.2669E-03					
41	1	A_2	40	0	A_1	1053.21447	3	.325604E-02	0.5	0.3132E-02	3.79	2.647E-04		-.440E+00	1.3
38	6	E	38	5	E	1053.26882	-1	.250398E-02	0.6	0.2278E-02	9.02	4.981E-04			1.0
37	6	E	37	5	E	1053.39186	-5	.251311E-02	0.6	0.2599E-02	-3.42	3.148E-04			1.0
14	4	A_2	13	3	A_1	1053.41410	2	.280998E-01	0.1	0.1419E-01	-.97	3.827E-04	.291E-01	-.304E+00	1.3
14	4	A_1	13	3	A_2	1053.41410	2			0.1419E-01					
25	9	E	26	8	E	1053.44487	-10			0.7623E-03					
36	6	E	36	5	E	1053.51169	-5	.303650E-02	0.8	0.2951E-02	2.83	2.951E-04			1.0
35	6	E	35	5	E	1053.62832	0	.333359E-02	0.5	0.3333E-02	.03	3.270E-04			1.0
23	3	E	22	2	E	1053.73120	7			0.1029E-01					
6	5	E	5	4	E	1053.73488	8			0.1410E-01					
34	6	E	34	5	E	1053.74141	-25			0.3745E-02					
32	2	E	31	1	E	1053.81060	-20			0.4799E-02					
33	6	E	33	5	E	1053.85173	-3	.423601E-02	0.7	0.4187E-02	1.15	3.164E-04	-.125E+00		1.1
51	0	E	50	1	E	1053.85968	-5			0.2193E-03					
9	7	A_1	10	6	A_2	1053.89076	-51			0.5040E-03					
9	7	A_2	10	6	A_1	1053.89076	-51			0.5040E-03					
42	1	A_1	41	0	A_2	1053.93181	5	.286140E-02	0.3	0.2702E-02	5.57	3.204E-04			1.0
32	6	E	32	5	E	1053.95857	-5	.451311E-02	0.4	0.4657E-02	-3.20	2.538E-04		-.371E+00	1.1
32	10	A_2	33	9	A_1	1053.99553	-9			0.3060E-03					
32	10	A_1	33	9	A_2	1053.99553	-9			0.3060E-03					
31	6	E	31	5	E	1054.06218	-5	.521624E-02	0.3	0.5153E-02	1.22	3.177E-04	-.524E-01	-.177E+00	1.1
30	6	E	30	5	E	1054.16256	-4	.557397E-02	0.4	0.5670E-02	-1.72	3.017E-04	-.122E+00		1.1
15	4	A_2	14	3	A_1	1054.25062	-2	.283542E-01	0.2	0.1396E-01	1.50	4.194E-04			1.0
15	4	A_1	14	3	A_2	1054.25062	-2			0.1396E-01					
29	6	E	29	5	E	1054.25970	-3	.622545E-02	0.9	0.6205E-02	.33	3.763E-04			1.0
28	6	E	28	5	E	1054.35360	-2	.667974E-02	0.4	0.6753E-02	-1.10	3.924E-04			1.0
24	9	E	25	8	E	1054.40701	-5	.774728E-03	1.6	0.7879E-03	-1.71	1.007E-04		-.525E+00	1.0
27	6	E	27	5	E	1054.44417	-10	.759985E-02	0.5	0.7307E-02	3.85	3.654E-04			1.0
39	11	E	40	10	E	1054.45342	-29			0.8718E-04					
26	6	E	26	5	E	1054.53168	0	.767185E-02	0.6	0.7861E-02	-2.47	2.890E-04			1.0
24	3	E	23	2	E	1054.53601	-7	.956482E-02	0.6	0.9672E-02	-1.12	2.890E-04			1.0
33	2	E	32	1	E	1054.58325	5	.434712E-02	1.4	0.4310E-02	.86	2.623E-04			1.0
7	5	E	6	4	E	1054.59842	0	.141485E-01	0.4	0.1416E-01	-.12	3.531E-04			1.0
25	6	E	25	5	E	1054.61580	-5	.819299E-02	0.3	0.8406E-02	-2.60	5.659E-04	.200E+00	.534E+00	1.3

(cont.)

J	K_a	Γ	J'	K'_a	Γ'	$\nu^{a)}$	$\delta_\nu{}^{b)}$	$S_\nu^{\rm exp}(294.45)^{c)}$	$\Delta_S{}^{d)}$	$S_\nu^{\rm calc}(294.45)^{e)}$	$\delta_S{}^{f)}$	$\Gamma_0{}^{g)}$	$\Delta_2{}^{g)}$	$\nu_{\rm VC}{}^{g)}$	$R^{h)}$
	1			2		3	4	5	6	7	8	9	10	11	12
43	1	A_2	42	0	A_1	1054.64469	-3	.243119E-02	0.8	0.2320E-02	4.57	2.829E-04			1.0
16	8	E	17	7	E	1054.67958	36			0.1106E-02					
24	6	E	24	5	E	1054.69673	-5	.897703E-02	0.4	0.8934E-02	.48	3.869E-04			1.0
23	6	E	23	5	E	1054.77442	-5	.952833E-02	0.2	0.9434E-02	.99	3.586E-04	-.502E-01	-.721E-01	1.1
8	7	A_2	9	6	A_1	1054.80592	-14	.593619E-03	1.7	0.2926E-03	1.42	4.498E-04			1.0
8	7	A_1	9	6	A_2	1054.80592	-14			0.2926E-03					
46	12	E	47	11	E	1054.82184	-53			0.1828E-04					
22	6	E	22	5	E	1054.84871	-21			0.9896E-02					
21	6	E	21	5	E	1054.92009	-4	.102297E-01	0.3	0.1031E-01	-.77	3.944E-04			1.0
31	10	A_2	32	9	A_1	1054.97720	0			0.3331E-03					
31	10	A_1	32	9	A_2	1054.97720	0			0.3331E-03					
20	6	E	20	5	E	1054.98807	-4	.107702E-01	0.3	0.1066E-01	1.02	3.890E-04			1.0
19	6	E	19	5	E	1055.05281	-3	.101661E-01	1.6	0.1094E-01	-7.60	1.154E-03			1.0
16	4	A_2	15	3	A_1	1055.08370	-3	.269819E-01	0.2	0.1367E-01	-1.33	3.792E-04			1.0
16	4	A_1	15	3	A_2	1055.08370	-3			0.1367E-01					
18	6	E	18	5	E	1055.11431	-3	.111900E-01	0.4	0.1113E-01	.51	4.107E-04			1.0
17	6	E	17	5	E	1055.17257	-3	.116414E-01	0.3	0.1123E-01	3.54	3.928E-04			1.0
16	6	E	16	5	E	1055.22760	-3	.116788E-01	0.4	0.1122E-01	3.96	4.252E-04			1.0
15	6	E	15	5	E	1055.27924	-17			0.1108E-01					
14	6	E	14	5	E	1055.32792	-4	.107197E-01	0.6	0.1082E-01	-.90	5.098E-04			1.0
25	3	E	24	2	E	1055.33740	-5	.899543E-02	0.9	0.9043E-02	-.52	3.690E-04			1.0
34	2	E	33	1	E	1055.35226	36			0.3851E-02					
23	9	E	24	8	E	1055.36643	15			0.8064E-03					
13	6	E	13	5	E	1055.37386	59			0.1041E-01					
12	6	E	12	5	E	1055.41532	-2	.983428E-02	0.4	0.9841E-02	-.07	5.773E-04	.437E+00	.145E+01	1.4
38	11	E	39	10	E	1055.45419	12	.965171E-02	0.6	0.9896E-04	4.59	4.092E-04			1.0
11	6	E	11	5	E	1055.45419	1			0.9109E-02					
8	5	E	7	4	E	1055.45866	-4	.156349E-01	0.6	0.1424E-01	8.91	4.092E-04			1.0
10	6	E	10	5	E	1055.48980	2	.862382E-02	0.2	0.8198E-02	4.94	3.218E-04	-.127E+00	-.442E+00	1.5
9	6	E	9	5	E	1055.52212	-2	.749188E-02	0.3	0.7091E-02	5.35	3.431E-04	-.208E+00	-.234E+00	1.8
8	6	E	8	5	E	1055.55128	1	.605326E-02	0.3	0.5765E-02	4.76	3.179E-04	-.215E+00	-.288E+00	1.8
7	6	E	7	5	E	1055.57712	-4	.417492E-02	0.5	0.4187E-02	-.29	3.524E-04	-.239E+00	-.299E+00	1.5
6	6	E	6	5	E	1055.59982	0	.236804E-02	1.6	0.2300E-02	2.88	4.865E-04			1.0
15	8	E	16	7	E	1055.61503	-11	.102621E-02	0.5	0.1012E-02	1.40	3.944E-04	-.375E+00		1.5
64	1	E	63	2	E	1055.71048	14			0.9273E-05					
7	7	A_1	8	6	A_2	1055.71797	23			0.1142E-03					
7	7	A_2	8	6	A_1	1055.71797	23			0.1142E-03					
17	4	A_2	16	3	A_1	1055.91333	-3	.265122E-01	0.2	0.1331E-01	-.38	4.435E-04			1.0
17	4	A_1	16	3	A_2	1055.91333	-3			0.1331E-01					
54	0	E	53	1	E	1055.93249	-18			0.1183E-03					
30	10	A_1	31	9	A_2	1055.95596	-5			0.3600E-03					
30	10	A_2	31	9	A_1	1055.95596	-5			0.3600E-03					
45	1	A_2	44	0	A_1	1056.05753	0	.169822E-02	1.3	0.1686E-02	.70	1.811E-04			1.0
35	2	E	34	1	E	1056.11693	3	.334821E-02	1.1	0.3425E-02	-2.28	1.785E-04			1.0
26	3	E	25	2	E	1056.13520	-1	.834387E-02	0.2	0.8410E-02	-.79	3.821E-04	-.133E+00		1.2
9	5	E	8	4	E	1056.31561	-2	.137546E-01	0.2	0.1430E-01	-3.96	3.635E-04			1.0
22	9	E	23	8	E	1056.32232	-30	.846416E-03	5.6	0.8163E-03	3.56	6.973E-04			1.0
37	11	E	38	10	E	1056.45173	-2			0.1117E-03					
14	8	E	15	7	E	1056.54795	-12			0.8998E-03					
55	0	E	54	1	E	1056.61526	5			0.9537E-04					
18	4	A_1	17	3	A_2	1056.73960	10			0.1288E-01					
18	4	A_2	17	3	A_1	1056.73960	10			0.1288E-01					
46	1	A_1	45	0	A_2	1056.75735	1	.152530E-02	1.1	0.1428E-02	6.40	3.305E-04	-.383E+00	.981E+00	1.5
44	12	E	45	11	E	1056.85647	-21			0.2523E-04					
36	2	E	35	1	E	1056.87818	-1	.303573E-02	1.5	0.3030E-02	.18	2.904E-04	.115E+00		1.0
27	3	E	26	2	E	1056.92934	-2	.765210E-02	0.4	0.7780E-02	-1.67	5.526E-04	.402E+00	.256E+00	1.6
10	5	E	9	4	E	1057.16916	-2	.136782E-01	0.3	0.1432E-01	-4.66	3.449E-04	-.251E+00	-.162E+00	1.3
21	9	E	22	8	E	1057.27601	-4			0.8166E-03					
56	0	E	55	1	E	1057.29345	-4			0.7656E-04					
58	7	A_2	58	6	A_1	1057.35605	-8			0.4791E-04					
58	7	A_1	58	6	A_2	1057.35605	-8			0.4791E-04					
47	1	A_2	46	0	A_1	1057.45276	2			0.1203E-02					
13	8	E	14	7	E	1057.47795	-2	.839351E-03	1.6	0.7718E-03	8.05	6.982E-04			1.0
57	7	A_2	57	6	A_1	1057.54428	1			0.5998E-04					
57	7	A_1	57	6	A_2	1057.54428	1			0.5998E-04					
19	4	A_2	18	3	A_1	1057.56211	-2	.256438E-01	0.3	0.1240E-01	3.27	4.562E-04			1.0
19	4	A_1	18	3	A_2	1057.56211	-2			0.1240E-01					
37	2	E	36	1	E	1057.63574	-2	.269921E-02	1.0	0.2668E-02	1.15	2.553E-04	-.250E+00	-.371E+00	1.3
28	3	E	27	2	E	1057.71985	-3	.715362E-02	0.6	0.7161E-02	-.10	2.648E-04		-.458E+00	1.1
43	12	E	44	11	E	1057.86910	-85			0.2941E-04					
28	10	A_2	29	9	A_1	1057.90522	-2			0.4110E-03					
28	10	A_1	29	9	A_2	1057.90522	-2			0.4110E-03					
55	7	A_2	55	6	A_1	1057.91050	-25			0.9276E-04					
55	7	A_1	55	6	A_2	1057.91050	-25			0.9276E-04					
57	0	E	56	1	E	1057.96743	-6			0.6117E-04					
11	5	E	10	4	E	1058.01934	-2	.136041E-01	0.3	0.1428E-01	-4.96	3.544E-04	.411E-01	-.327E+00	1.3
54	7	A_1	54	6	A_2	1058.08903	-7			0.1146E-03					
54	7	A_2	54	6	A_1	1058.08903	-7			0.1146E-03					
48	1	A_1	47	0	A_2	1058.14374	3			0.1009E-02					
20	9	E	21	8	E	1058.22649	-7	.750794E-03	1.8	0.8062E-03	-7.39	4.458E-04	.301E+00		1.1
53	7	A_1	53	6	A_2	1058.26406	-12			0.1409E-03					
53	7	A_2	53	6	A_1	1058.26406	-12			0.1409E-03					

(cont.)

J	K_a	Γ	J'	K'_a	Γ'	$\nu^{a)}$	$\delta_\nu{}^{b)}$	$S_\nu^{\rm exp}(294.45)^{c)}$	$\Delta_S{}^{d)}$	$S_\nu^{\rm calc}(294.45)^{e)}$	$\delta_S{}^{f)}$	$\Gamma_0{}^{g)}$	$\Delta_2{}^{g)}$	$\nu_{\rm VC}{}^{g)}$	$R^{h)}$
	1			2		3	4	5	6	7	8	9	10	11	12
20	4	A_2	19	3	A_1	1058.38124	-2	.237535E-01	0.1	0.1188E-01	.01	4.206E-04			1.0
20	4	A_1	19	3	A_2	1058.38124	-2			0.1188E-01					
38	2	E	37	1	E	1058.38956	-2	.234912E-02	1.4	0.2338E-02	.47	3.669E-04			1.0
12	8	E	13	7	E	1058.40515	30	.656436E-03	4.2	0.6310E-03	3.87	5.280E-04			1.0
52	7	A_1	52	6	A_2	1058.43591	-9			0.1725E-03					
52	7	A_2	52	6	A_1	1058.43591	-9			0.1725E-03					
29	3	E	28	2	E	1058.50674	-2	.669816E-02	0.3	0.6557E-02	2.10	3.031E-04	-.808E-01	-.276E+00	1.2
58	0	E	57	1	E	1058.63711	-7			0.4866E-04					
50	7	A_2	50	6	A_1	1058.76980	-6			0.2550E-03					
50	7	A_1	50	6	A_2	1058.76980	-6			0.2550E-03					
49	1	A_2	48	0	A_1	1058.83024	0			0.8428E-03					
12	5	E	11	4	E	1058.86612	-1	.132973E-01	0.2	0.1418E-01	-6.64	3.954E-04			1.0
27	10	A_1	28	9	A_2	1058.87524	-40			0.4338E-03					
27	10	A_2	28	9	A_1	1058.87524	-40			0.4338E-03					
49	7	A_1	49	6	A_2	1058.93184	-7			0.3079E-03					
49	7	A_2	49	6	A_1	1058.93184	-7			0.3079E-03					
48	7	A_1	48	6	A_2	1059.09064	-5			0.3701E-03					
48	7	A_2	48	6	A_1	1059.09064	-5			0.3701E-03					
39	2	E	38	1	E	1059.13960	-5	.211192E-02	0.6	0.2039E-02	3.46	2.557E-04	.123E+00	-.252E+00	1.1
19	9	E	20	8	E	1059.17411	-3	.759854E-03	1.5	0.7843E-03	-3.21	3.886E-04	.331E+00		1.1
21	4	A_2	20	3	A_1	1059.19681	-4	.226616E-01	0.2	0.1131E-01	.17	3.833E-04		-.276E+00	1.1
21	4	A_1	20	3	A_2	1059.19681	-4			0.1131E-01					
47	7	A_2	47	6	A_1	1059.24601	-22			0.4428E-03					
47	7	A_1	47	6	A_2	1059.24601	-22			0.4428E-03					
30	3	E	29	2	E	1059.28993	-5	.617395E-02	0.6	0.5974E-02	3.23	3.759E-04	.213E+00		1.2
59	0	E	58	1	E	1059.30231	-25			0.3853E-04					
11	8	E	12	7	E	1059.32852	-16	.465585E-03	3.3	0.4824E-03	-3.61	2.879E-04			1.0
46	7	A_2	46	6	A_1	1059.39844	-6	.112742E-02	3.0	0.5274E-03	6.45	1.211E-04			1.0
46	7	A_1	46	6	A_2	1059.39844	-6			0.5274E-03					
34	11	E	35	10	E	1059.42849	-11			0.1545E-03					
50	1	A_1	49	0	A_2	1059.51230	-1			0.7006E-03					
45	7	A_2	45	6	A_1	1059.54746	-7	.125600E-02	0.7	0.6251E-03	.46	2.951E-04	-.190E+00		1.1
45	7	A_1	45	6	A_2	1059.54746	-7			0.6251E-03					
44	7	A_2	44	6	A_1	1059.69320	-10	.146513E-02	1.4	0.7375E-03	-.67	1.991E-04			1.0
44	7	A_1	44	6	A_2	1059.69320	-10			0.7375E-03					
13	5	E	12	4	E	1059.70949	-1	.130446E-01	0.3	0.1402E-01	-7.46	3.756E-04	-.336E-01	-.326E+00	1.1
43	7	A_2	43	6	A_1	1059.83577	-6	.174625E-02	0.6	0.8660E-03	.82	2.777E-04			1.0
43	7	A_1	43	6	A_2	1059.83577	-6			0.8660E-03					
26	10	A_2	27	9	A_1	1059.84313	-7	.898734E-03	1.1	0.4539E-03	-1.01	5.164E-04			1.0
26	10	A_1	27	9	A_2	1059.84313	-7			0.4539E-03					
40	2	E	39	1	E	1059.88595	0	.184459E-02	0.8	0.1770E-02	4.07	2.431E-04		-.656E+00	1.2
60	0	E	59	1	E	1059.96354	-4			0.3037E-04					
42	7	A_2	42	6	A_1	1059.97503	-7	.202331E-02	0.9	0.1012E-02	-.04	3.207E-04			1.0
42	7	A_1	42	6	A_2	1059.97503	-7			0.1012E-02					
22	4	A_2	21	3	A_1	1060.00905	15	.215835E-01	0.2	0.1072E-01	.70	4.040E-04	.813E-01	.219E+00	1.1
22	4	A_1	21	3	A_2	1060.00905	15			0.1072E-01					
31	3	E	30	2	E	1060.06959	5			0.5416E-02					
41	7	A_2	41	6	A_1	1060.11129	17	.246048E-02	0.6	0.1177E-02	4.33	3.104E-04			1.0
41	7	A_1	41	6	A_2	1060.11129	17			0.1177E-02					
18	9	E	19	8	E	1060.11867	-11	.731958E-03	2.4	0.7500E-03	-2.47	5.119E-04			1.0
51	1	A_2	50	0	A_1	1060.18998	8			0.5797E-03					
40	7	A_2	40	6	A_1	1060.24400	10	.278735E-02	0.4	0.1362E-02	2.25	3.020E-04			1.0
40	7	A_1	40	6	A_2	1060.24400	10			0.1362E-02					
10	8	E	11	7	E	1060.24924	-21			0.3328E-03					
39	7	A_2	39	6	A_1	1060.37355	12	.310525E-02	0.5	0.1569E-02	-1.06	2.241E-04		-.310E+00	1.1
39	7	A_1	39	6	A_2	1060.37355	12			0.1569E-02					
33	11	E	34	10	E	1060.41536	-7			0.1700E-03					
38	7	A_2	38	6	A_1	1060.49980	9	.351315E-02	0.5	0.1798E-02	-2.39	2.743E-04			1.0
38	7	A_1	38	6	A_2	1060.49980	9			0.1798E-02					
14	5	E	13	4	E	1060.54961	18	.128167E-01	0.3	0.1379E-01	-7.60	4.145E-04			1.0
37	7	A_2	37	6	A_1	1060.62286	11	.411408E-02	0.5	0.2051E-02	.29	2.807E-04			1.0
37	7	A_1	37	6	A_2	1060.62286	11			0.2051E-02					
41	2	E	40	1	E	1060.62868	20	.159285E-02	1.2	0.1529E-02	4.04	2.291E-04			1.0
36	7	A_2	36	6	A_1	1060.74266	12	.466433E-02	0.5	0.2328E-02	.20	2.975E-04			1.0
36	7	A_1	36	6	A_2	1060.74266	12			0.2328E-02					
25	10	A_2	26	9	A_1	1060.80828	36	.927114E-03	4.5	0.4705E-03	-1.50	3.349E-04			1.0
25	10	A_1	26	9	A_2	1060.80828	36			0.4705E-03					
23	4	A_2	22	3	A_1	1060.81753	14	.196040E-01	0.2	0.1010E-01	-3.04	3.886E-04			1.0
23	4	A_1	22	3	A_2	1060.81753	14			0.1010E-01					
32	3	E	31	2	E	1060.84555	15	.479321E-02	0.6	0.4886E-02	-1.93	3.431E-04	-.163E+00		1.1
35	7	A_2	35	6	A_1	1060.85901	-8			0.2628E-02					
35	7	A_1	35	6	A_2	1060.85901	-8			0.2628E-02					
6	6	E	5	5	E	1060.91832	18	.142575E-01	0.3	0.1410E-01	1.10	2.528E-04		-.447E+00	1.4
34	7	A_2	34	6	A_1	1060.97250	11	.565687E-02	0.7	0.2952E-02	-4.36	2.760E-04			1.0
34	7	A_1	34	6	A_2	1060.97250	11			0.2952E-02					
17	9	E	18	8	E	1061.06059	13	.742336E-03	1.4	0.7032E-03	5.27	3.238E-04			1.0
33	7	A_2	33	6	A_1	1061.08256	11	.677862E-02	0.3	0.3298E-02	2.69	3.512E-04	-.256E+00	-.956E-01	1.3
33	7	A_1	33	6	A_2	1061.08256	11			0.3298E-02					
9	8	E	10	7	E	1061.16675	-39			0.1921E-03					
32	7	A_2	32	6	A_1	1061.18939	12	.718043E-02	0.3	0.3666E-02	-2.11	2.974E-04	-.219E+00	-.159E+00	1.8
32	7	A_1	32	6	A_2	1061.18939	12			0.3666E-02					
62	0	E	61	1	E	1061.27256	5			0.1861E-04					

(cont.)

J	K_a	Γ	J'	K'_a	Γ'	$\nu^{a)}$	$\delta_\nu{}^{b)}$	$S_\nu^{\mathrm{exp}}(294.45)^{c)}$	$\Delta_S{}^{d)}$	$S_\nu^{\mathrm{calc}}(294.45)^{e)}$	$\delta_S{}^{f)}$	$\Gamma_0{}^{g)}$	$\Delta_2{}^{g)}$	$\nu_{\mathrm{VC}}{}^{g)}$	$R^{h)}$
1			2			3	4	5	6	7	8	9	10	11	12
47	13	A_2	48	12	A_1	1061.28101	-109			0.8755E-05					
47	13	A_1	48	12	A_2	1061.28101	-109			0.8755E-05					
31	7	A_2	31	6	A_1	1061.29297	12	.800858E-02	0.3	0.4053E-02	-1.21	3.080E-04	-.253E+00	-.319E+00	2.1
31	7	A_1	31	6	A_2	1061.29297	12			0.4053E-02					
42	2	E	41	1	E	1061.36745	23	.133309E-02	1.0	0.1314E-02	1.43	3.083E-04	-.515E+00	-.152E+00	1.7
15	5	E	14	4	E	1061.38612	20	.127058E-01	0.4	0.1350E-01	-6.24	4.182E-04			1.0
30	7	A_2	30	6	A_1	1061.39328	10	.868644E-02	0.7	0.4456E-02	-2.60	3.325E-04			1.0
30	7	A_1	30	6	A_2	1061.39328	10			0.4456E-02					
32	11	E	33	10	E	1061.39982	30			0.1856E-03					
29	7	A_2	29	6	A_1	1061.49020	-8	.963112E-02	0.3	0.4873E-02	-1.18	3.236E-04		-.200E+00	1.1
29	7	A_1	29	6	A_2	1061.49020	-8			0.4873E-02					
53	1	A_2	52	0	A_1	1061.53134	-25			0.3916E-03					
28	7	A_2	28	6	A_1	1061.58405	-8	.104365E-01	0.4	0.5297E-02	-1.52	3.265E-04			1.0
28	7	A_1	28	6	A_2	1061.58405	-8			0.5297E-02					
33	3	E	32	2	E	1061.61758	1	.448997E-02	2.2	0.4386E-02	2.33	2.404E-04			1.0
24	4	A_2	23	3	A_1	1061.62217	-13	.191312E-01	0.4	0.9470E-02	1.00	4.517E-04			1.0
24	4	A_1	23	3	A_2	1061.62217	-13			0.9470E-02					
27	7	A_2	27	6	A_1	1061.67465	-9	.114545E-01	0.4	0.5726E-02	.02	3.431E-04			1.0
27	7	A_1	27	6	A_2	1061.67465	-9			0.5726E-02					
26	7	A_2	26	6	A_1	1061.76223	11	.130717E-01	1.0	0.6152E-02	5.87	8.492E-04			1.0
26	7	A_1	26	6	A_2	1061.76223	11			0.6152E-02					
24	10	A_2	25	9	A_1	1061.76963	-15			0.4828E-03					
24	10	A_1	25	9	A_2	1061.76963	-15			0.4828E-03					
7	6	E	6	5	E	1061.78163	-2	.140076E-01	2.4	0.1395E-01	.42	1.795E-04			1.0
25	7	A_2	25	6	A_1	1061.84616	-9	.128023E-01	0.5	0.6569E-02	-2.62	3.862E-04			1.0
25	7	A_1	25	6	A_2	1061.84616	-9			0.6569E-02					
24	7	A_2	24	6	A_1	1061.92707	-8	.135671E-01	0.3	0.6969E-02	-2.74	2.961E-04	-.119E+00	-.416E+00	2.0
24	7	A_1	24	6	A_2	1061.92707	-8			0.6969E-02					
16	9	E	17	8	E	1061.99899	-17			0.6440E-03					
23	7	A_2	23	6	A_1	1062.00473	-7	.144044E-01	0.2	0.7345E-02	-1.99	3.777E-04			1.0
23	7	A_1	23	6	A_2	1062.00473	-7			0.7345E-02					
22	7	A_2	22	6	A_1	1062.07914	-8	.155075E-01	0.2	0.7688E-02	.85	4.070E-04	-.738E-01	.114E+00	1.1
22	7	A_1	22	6	A_2	1062.07914	-8			0.7688E-02					
43	2	E	42	1	E	1062.10205	-11	.119616E-02	2.4	0.1124E-02	6.00	2.400E-04			1.0
21	7	A_2	21	6	A_1	1062.15033	-8	.162679E-01	0.2	0.7988E-02	1.79	4.241E-04			1.0
21	7	A_1	21	6	A_2	1062.15033	-8			0.7988E-02					
54	1	A_1	53	0	A_2	1062.19580	14			0.3197E-03					
20	7	A_2	20	6	A_1	1062.21854	19	.298237E-01	0.2	0.8236E-02	.69	4.670E-04			1.0
20	7	A_1	20	6	A_2	1062.21854	19			0.8236E-02					
16	5	E	15	4	E	1062.21854	-42			0.1315E-01					
19	7	A_2	19	6	A_1	1062.28298	-8	.168079E-01	0.2	0.8421E-02	-.20	3.945E-04	-.667E-01	-.950E-01	1.1
19	7	A_1	19	6	A_2	1062.28298	-8			0.8421E-02					
18	7	A_2	18	6	A_1	1062.34444	-9	.170228E-01	0.2	0.8533E-02	-.26	3.921E-04			1.0
18	7	A_1	18	6	A_2	1062.34444	-9			0.8533E-02					
34	3	E	33	2	E	1062.38597	-5	.403842E-02	2.1	0.3917E-02	3.00	3.613E-04			1.0
17	7	A_2	17	6	A_1	1062.40270	-6	.166928E-01	0.5	0.8563E-02	-2.59	4.175E-04			1.0
17	7	A_1	17	6	A_2	1062.40270	-6			0.8563E-02					
25	4	A_2	24	3	A_1	1062.42355	-8	.179725E-01	0.2	0.8833E-02	1.70	3.858E-04			1.0
25	4	A_1	24	3	A_2	1062.42355	-8			0.8833E-02					
16	7	A_2	16	6	A_1	1062.45771	-5	.173242E-01	0.3	0.8498E-02	1.89	4.265E-04			1.0
16	7	A_1	16	6	A_2	1062.45771	-5			0.8498E-02					
15	7	A_2	15	6	A_1	1062.50944	-8	.164009E-01	0.3	0.8331E-02	-1.59	3.758E-04			1.0
15	7	A_1	15	6	A_2	1062.50944	-8			0.8331E-02					
14	7	A_2	14	6	A_1	1062.55798	-7	.165414E-01	0.3	0.8049E-02	2.69	4.185E-04			1.0
14	7	A_1	14	6	A_2	1062.55798	-7			0.8049E-02					
13	7	A_2	13	6	A_1	1062.60325	-9	.157467E-01	0.3	0.7642E-02	2.94	4.041E-04			1.0
13	7	A_1	13	6	A_2	1062.60325	-9			0.7642E-02					
8	6	E	7	5	E	1062.64182	-1	.142908E-01	1.4	0.1385E-01	3.11	3.515E-04			1.0
12	7	A_2	12	6	A_1	1062.64535	-4	.139476E-01	1.4	0.7099E-02	-1.80	3.515E-04			1.0
12	7	A_1	12	6	A_2	1062.64535	-4			0.7099E-02					
11	7	A_2	11	6	A_1	1062.68415	-6	.130698E-01	0.3	0.6409E-02	1.93	4.008E-04			1.0
11	7	A_1	11	6	A_2	1062.68415	-6			0.6409E-02					
10	7	A_2	10	6	A_1	1062.71974	-5	.111340E-01	0.2	0.5557E-02	.19	3.111E-04	-.122E+00	-.307E+00	1.6
10	7	A_1	10	6	A_2	1062.71974	-5			0.5557E-02					
23	10	A_2	24	9	A_1	1062.72855	-21			0.4900E-03					
23	10	A_1	24	9	A_2	1062.72855	-21			0.4900E-03					
9	7	A_2	9	6	A_1	1062.75206	-8	.917907E-02	0.3	0.4524E-02	1.44	3.082E-04	-.147E+00	-.373E+00	1.8
9	7	A_1	9	6	A_2	1062.75206	-8			0.4524E-02					
8	7	A_2	8	6	A_1	1062.78119	-6	.676532E-02	0.3	0.3284E-02	2.92	3.194E-04	-.122E+00	-.287E+00	1.2
8	7	A_1	8	6	A_2	1062.78119	-6			0.3284E-02					
7	7	A_2	7	6	A_1	1062.80707	-6	.372297E-02	0.6	0.1798E-02	3.41	3.821E-04			1.0
7	7	A_1	7	6	A_2	1062.80707	-6			0.1798E-02					
44	2	E	43	1	E	1062.83329	0	.100179E-02	1.3	0.9577E-03	4.40	2.223E-04			1.0
55	1	A_2	54	0	A_1	1062.85520	0			0.2598E-03					
38	12	E	39	11	E	1062.89667	-22			0.5808E-04					
15	9	E	16	8	E	1062.93482	-6			0.5729E-03					
17	5	E	16	4	E	1063.04847	-6	.121539E-01	0.2	0.1274E-01	-4.81	4.057E-04	-.148E+00	-.111E+00	1.3
35	3	E	34	2	E	1063.15070	-5	.350433E-02	0.4	0.3482E-02	.63	3.281E-04	-.261E+00	-.384E+00	1.8
65	0	E	64	1	E	1063.20262	-12			0.8633E-05					
26	4	A_2	25	3	A_1	1063.22131	-4	.166573E-01	0.2	0.8198E-02	1.57	3.685E-04	-.528E-01	-.391E+00	1.6
26	4	A_1	25	3	A_2	1063.22131	-4			0.8198E-02					
45	13	A_2	46	12	A_1	1063.32093	-6			0.1218E-04					

(cont.)

J	K_a	Γ	J'	K'_a	Γ'	$\nu^{a)}$	$\delta_\nu{}^{b)}$	$S_\nu^{\rm exp}(294.45)^{c)}$	$\Delta_S{}^{d)}$	$S_\nu^{\rm calc}(294.45)^{e)}$	$\delta_S{}^{f)}$	$\Gamma_0{}^{g)}$	$\Delta_2{}^{g)}$	$\nu_{\rm VC}{}^{g)}$	$R^{h)}$
1			2			3	4	5	6	7	8	9	10	11	12
45	13	A_1	46	12	A_2	1063.32093	-6			0.1218E-04					
9	6	E	8	5	E	1063.49860	-5	.139356E-01	0.3	0.1375E-01	1.32	3.083E-04		-.375E+00	1.3
56	1	A_1	55	0	A_2	1063.50997	-21			0.2102E-03					
45	2	E	44	1	E	1063.56062	4	.862816E-03	1.5	0.8118E-03	5.91	3.809E-04			1.0
22	10	A_1	23	9	A_2	1063.68474	-12			0.4914E-03					
22	10	A_2	23	9	A_1	1063.68474	-12			0.4914E-03					
18	5	E	17	4	E	1063.87456	-5	.119559E-01	0.4	0.1228E-01	-2.72	4.251E-04			1.0
36	3	E	35	2	E	1063.91167	-6	.318005E-02	0.7	0.3081E-02	3.12	2.750E-04	-.866E-01	-.316E+00	1.1
27	4	A_2	26	3	A_1	1064.01540	-6	.153741E-01	0.1	0.7570E-02	1.53	3.510E-04	-.154E+00	-.835E-01	1.7
27	4	A_1	26	3	A_2	1064.01540	-6			0.7570E-02					
57	1	A_2	56	0	A_1	1064.16015	-44			0.1694E-03					
46	2	E	45	1	E	1064.28411	7			0.6850E-03					
29	11	E	30	10	E	1064.33492	-23			0.2313E-03					
10	6	E	9	5	E	1064.35206	-5	.140459E-01	0.1	0.1364E-01	2.90	3.614E-04	-.135E+00	-.121E+00	1.9
21	10	A_2	22	9	A_1	1064.63803	-2	.911479E-03	2.7	0.4862E-03	-6.68	2.962E-04			1.0
21	10	A_1	22	9	A_2	1064.63803	-2			0.4862E-03					
37	3	E	36	2	E	1064.66889	-7	.280875E-02	0.9	0.2712E-02	3.43	3.044E-04	-.200E+00		1.2
19	5	E	18	4	E	1064.69714	-5	.115329E-01	0.4	0.1178E-01	-2.14	4.158E-04	-.159E+00	-.134E+00	1.5
13	9	E	14	8	E	1064.79719	-10			0.4014E-03					
28	4	A_2	27	3	A_1	1064.80588	-5	.139468E-01	0.4	0.6955E-02	.27	3.045E-04	-.700E-01	-.242E+00	1.1
28	4	A_1	27	3	A_2	1064.80588	-5			0.6955E-02					
36	12	E	37	11	E	1064.88895	-6			0.7310E-04					
47	2	E	46	1	E	1065.00375	10			0.5754E-03					
56	8	E	56	7	E	1065.00375	-88			0.5663E-04					
55	8	E	55	7	E	1065.18551	-70			0.7027E-04					
11	6	E	10	5	E	1065.20214	-4	.139426E-01	0.2	0.1349E-01	3.24	3.393E-04	-.782E-01	-.387E+00	1.6
28	11	E	29	10	E	1065.30804	-6			0.2449E-03					
54	8	E	54	7	E	1065.36450	-2			0.8679E-04					
38	3	E	37	2	E	1065.42233	-8	.241882E-02	0.8	0.2377E-02	1.74	2.811E-04			1.0
59	1	A_2	58	0	A_1	1065.44764	0			0.1084E-03					
20	5	E	19	4	E	1065.51622	-4	.111643E-01	0.5	0.1124E-01	-.69	3.216E-04	-.232E+00	-.399E+00	1.4
53	8	E	53	7	E	1065.53952	-6			0.1067E-03					
20	10	A_2	21	9	A_1	1065.58881	48			0.4739E-03					
20	10	A_1	21	9	A_2	1065.58881	48			0.4739E-03					
29	4	A_2	28	3	A_1	1065.59270	-6	.128051E-01	0.3	0.6358E-02	.69	3.607E-04			1.0
29	4	A_1	28	3	A_2	1065.59270	-6			0.6358E-02					
52	8	E	52	7	E	1065.71139	3			0.1306E-03					
48	2	E	47	1	E	1065.71949	9			0.4811E-03					
12	9	E	13	8	E	1065.72391	-4			0.3064E-03					
51	8	E	51	7	E	1065.87975	-14			0.1592E-03					
35	12	E	36	11	E	1065.88048	-54			0.8122E-04					
12	6	E	11	5	E	1066.04881	-5	.135657E-01	0.2	0.1330E-01	1.94	4.300E-04	-.419E-01	.252E+00	1.1
60	1	A_1	59	0	A_2	1066.08497	72			0.8620E-04					
39	3	E	38	2	E	1066.17204	-4	.204876E-02	1.0	0.2073E-02	-1.18	2.697E-04	-.203E+00		1.1
49	8	E	49	7	E	1066.20722	5			0.2331E-03					
27	11	E	28	10	E	1066.27822	-2			0.2570E-03					
21	5	E	20	4	E	1066.33175	-5	.106866E-01	0.5	0.1067E-01	.13	4.103E-04	.429E-01	-.257E+00	1.1
48	8	E	48	7	E	1066.36610	17			0.2801E-03					
30	4	A_2	29	3	A_1	1066.37587	-6	.118401E-01	0.4	0.5785E-02	2.29	3.048E-04	-.248E+00	-.458E+00	1.9
30	4	A_1	29	3	A_2	1066.37587	-6			0.5785E-02					
49	2	E	48	1	E	1066.43135	9			0.4004E-03					
47	8	E	47	7	E	1066.52141	-2			0.3351E-03					
19	10	A_2	20	9	A_1	1066.53560	-8	.940314E-03	1.1	0.4540E-03	3.43	4.153E-04			1.0
19	10	A_1	20	9	A_2	1066.53560	-8			0.4540E-03					
11	9	E	12	8	E	1066.64749	-8			0.2108E-03					
46	8	E	46	7	E	1066.67361	-6			0.3990E-03					
61	1	A_2	60	0	A_1	1066.71616	-6			0.6822E-04					
45	8	E	45	7	E	1066.82270	4	.469317E-03	1.6	0.4728E-03	-.74	3.524E-04			1.0
34	12	E	35	11	E	1066.87080	49			0.8963E-04					
13	6	E	12	5	E	1066.89208	-5	.133365E-01	0.2	0.1307E-01	2.03	4.122E-04	-.137E+00	.868E-01	1.1
40	3	E	39	2	E	1066.91794	-2	.174720E-02	1.0	0.1799E-02	-2.99	1.909E-04	-.316E+00	-.179E+00	1.3
44	8	E	44	7	E	1066.96842	2			0.5577E-03					
43	8	E	43	7	E	1067.11090	1			0.6546E-03					
22	5	E	21	4	E	1067.14373	-6	.101583E-01	0.4	0.1008E-01	.74	3.887E-04			1.0
31	4	A_2	30	3	A_1	1067.15537	-5	.106024E-01	0.4	0.5237E-02	1.21	3.363E-04			1.0
31	4	A_1	30	3	A_2	1067.15537	-5			0.5237E-02					
26	11	E	27	10	E	1067.24575	20			0.2671E-03					
42	8	E	42	7	E	1067.25008	-5			0.7648E-03					
62	1	A_1	61	0	A_2	1067.34360	5			0.5376E-04					
41	8	E	41	7	E	1067.38661	49			0.8892E-03					
18	10	A_2	19	9	A_1	1067.48004	-4	.820475E-03	1.2	0.4264E-03	-3.93	3.701E-04	-.212E+00		1.1
18	10	A_1	19	9	A_2	1067.48004	-4			0.4264E-03					
40	8	E	40	7	E	1067.51886	0	.100814E-02	0.9	0.1029E-02	-2.05	2.846E-04	-.226E+00		1.2
10	9	E	11	8	E	1067.56804	-9			0.1212E-03					
39	8	E	39	7	E	1067.64827	-9	.118849E-02	0.8	0.1185E-02	.34	2.796E-04			1.0
41	3	E	40	2	E	1067.66005	3	.151893E-02	0.7	0.1555E-02	-2.35	3.150E-04			1.0
14	6	E	13	5	E	1067.73199	2	.131271E-01	0.2	0.1278E-01	2.65	3.913E-04		-.314E+00	1.2
38	8	E	38	7	E	1067.77462	2	.137521E-02	0.7	0.1357E-02	1.32	5.622E-04	-.134E+00	.189E+01	1.2
51	2	E	50	1	E	1067.84337	5			0.2736E-03					
33	12	E	34	11	E	1067.85687	0			0.9822E-04					
37	8	E	37	7	E	1067.89760	-1	.158022E-02	0.9	0.1547E-02	2.11	3.287E-04	-.461E+00	-.118E+00	1.4
32	4	A_2	31	3	A_1	1067.93127	5	.988340E-02	0.2	0.4718E-02	4.53	3.072E-04	-.215E+00	-.173E+00	2.0

(cont.)

J	K_a	Γ	J'	K'_a	Γ'	$\nu^{a)}$	$\delta_\nu{}^{b)}$	$S_\nu^{\exp}(294.45)^{c)}$	$\Delta_S{}^{d)}$	$S_\nu^{\mathrm{calc}}(294.45)^{e)}$	$\delta_S{}^{f)}$	$\Gamma_0{}^{g)}$	$\Delta_2{}^{g)}$	$\nu_{\mathrm{VC}}{}^{g)}$	$R^{h)}$
	1			2		3	4	5	6	7	8	9	10	11	12
32	4	A_1	31	3	A_2	1067.93127	5			0.4718E-02					
23	5	E	22	4	E	1067.95225	2	.953290E-02	0.4	0.9478E-02	.58	4.131E-04	-.208E+00	-.196E+00	1.2
36	8	E	36	7	E	1068.01737	1	.174274E-02	0.6	0.1754E-02	-.67	3.002E-04	-.144E+00		1.1
35	8	E	35	7	E	1068.13385	-3	.189311E-02	1.2	0.1980E-02	-4.57	2.276E-04	-.167E+00		1.1
25	11	E	26	10	E	1068.20980	-21			0.2749E-03					
34	8	E	34	7	E	1068.24708	-7	.218964E-02	0.7	0.2222E-02	-1.48	3.012E-04			1.0
33	8	E	33	7	E	1068.35740	23			0.2481E-02					
40	13	A_2	41	12	A_1	1068.37283	7			0.2521E-04					
40	13	A_1	41	12	A_2	1068.37283	7			0.2521E-04					
42	3	E	41	2	E	1068.39823	-2	.135825E-02	0.6	0.1337E-02	1.57	3.485E-04	-.121E+00		1.1
17	10	A_2	18	9	A_1	1068.42154	2	.775618E-03	0.8	0.3909E-03	-.80	3.780E-04	.137E+00		1.1
17	10	A_1	18	9	A_2	1068.42154	2			0.3909E-03					
32	8	E	32	7	E	1068.46397	1	.288056E-02	0.7	0.2756E-02	4.33	3.655E-04			1.0
9	9	E	10	8	E	1068.48566	4			0.4664E-04					
52	2	E	51	1	E	1068.54360	12			0.2246E-03					
31	8	E	31	7	E	1068.56822	72	.161339E-01	0.3	0.3044E-02	4.00	4.288E-04	-.294E+00	-.624E+00	2.2
15	6	E	14	5	E	1068.56822	-15			0.1245E-01					
30	8	E	30	7	E	1068.66773	-8	.342154E-02	0.9	0.3344E-02	2.28	3.995E-04			1.0
33	4	A_2	32	3	A_1	1068.70332	0	.880202E-02	0.3	0.4230E-02	3.88	3.859E-04	-.228E+00		1.5
33	4	A_1	32	3	A_2	1068.70332	0			0.4230E-02					
24	5	E	23	4	E	1068.75710	1	.890469E-02	0.5	0.8865E-02	.45	3.773E-04			1.0
29	8	E	29	7	E	1068.76485	-2	.367559E-02	1.3	0.3652E-02	.65	3.798E-04			1.0
32	12	E	33	11	E	1068.84045	-23			0.1069E-03					
28	8	E	28	7	E	1068.85868	-1	.397304E-02	0.5	0.3965E-02	.19	3.238E-04	.811E-01	-.376E+00	1.2
27	8	E	27	7	E	1068.94928	0	.419161E-02	0.6	0.4280E-02	-2.11	3.744E-04			1.0
7	7	A_2	6	6	A_1	1069.01062	2	.255173E-01	0.2	0.1299E-01	-1.80	2.552E-04	-.294E+00	-.340E+00	2.1
7	7	A_1	6	6	A_2	1069.01062	2			0.1299E-01					
26	8	E	26	7	E	1069.03660	-2	.458797E-02	0.4	0.4591E-02	-.07	4.355E-04	-.469E+00	-.364E+00	2.0
25	8	E	25	7	E	1069.12072	-1	.509120E-02	0.8	0.4894E-02	3.88	3.861E-04			1.0
43	3	E	42	2	E	1069.13210	-54			0.1144E-02					
24	11	E	25	10	E	1069.17157	-4			0.2797E-03					
24	8	E	24	7	E	1069.20142	-18			0.5181E-02					
53	2	E	52	1	E	1069.23980	8			0.1836E-03					
23	8	E	23	7	E	1069.27923	0	.543066E-02	0.6	0.5448E-02	-.32	3.728E-04			1.0
22	8	E	22	7	E	1069.35365	3	.585150E-02	0.4	0.5686E-02	2.82	4.159E-04			1.0
16	10	A_1	17	9	A_2	1069.35999	0			0.3481E-03					
16	10	A_2	17	9	A_1	1069.35999	0			0.3481E-03					
39	13	A_1	40	12	A_2	1069.37528	6			0.2865E-04					
39	13	A_2	40	12	A_1	1069.37528	6			0.2865E-04					
16	6	E	15	5	E	1069.40134	2	.126697E-01	0.4	0.1206E-01	4.77	4.194E-04			1.0
21	8	E	21	7	E	1069.42477	-1	.593657E-02	0.4	0.5889E-02	.80	3.853E-04	-.153E+00		1.1
34	4	A_2	33	3	A_1	1069.47171	1	.783045E-02	0.3	0.3775E-02	3.59	3.015E-04			1.0
34	4	A_1	33	3	A_2	1069.47171	1			0.3775E-02					
20	8	E	20	7	E	1069.49270	0	.605183E-02	0.4	0.6049E-02	.05	4.041E-04			1.0
19	8	E	19	7	E	1069.55797	58	.148769E-01	0.3	0.6157E-02	3.16	6.249E-04			1.0
25	5	E	24	4	E	1069.55797	-39			0.8250E-02					
18	8	E	18	7	E	1069.61883	-1	.609432E-02	0.2	0.6205E-02	-1.81	3.201E-04	-.132E+00	-.409E+00	1.7
17	8	E	17	7	E	1069.67704	-1	.646871E-02	0.3	0.6185E-02	4.39	4.309E-04			1.0
16	8	E	16	7	E	1069.73210	7			0.6088E-02					
15	8	E	15	7	E	1069.78373	-4	.625250E-02	0.4	0.5905E-02	5.55	4.273E-04			1.0
31	12	E	32	11	E	1069.82112	-60			0.1154E-03					
14	8	E	14	7	E	1069.83227	0	.561748E-02	0.6	0.5628E-02	-.19	3.762E-04			1.0
44	3	E	43	2	E	1069.86336	18			0.9751E-03					
8	7	A_2	7	6	A_1	1069.87065	0	.248541E-01	0.2	0.1275E-01	-2.62	3.076E-04			1.0
8	7	A_1	7	6	A_2	1069.87065	0			0.1275E-01					
13	8	E	13	7	E	1069.87754	-1	.556322E-02	1.0	0.5247E-02	5.69	4.403E-04			1.0
12	8	E	12	7	E	1069.91956	-3	.478583E-02	0.3	0.4751E-02	.73	3.218E-04	.365E-01	-.322E+00	1.2
54	2	E	53	1	E	1069.93217	15			0.1494E-03					
11	8	E	11	7	E	1069.95839	0	.419577E-02	0.3	0.4129E-02	1.59	3.177E-04	-.174E+00	-.374E+00	2.0
10	8	E	10	7	E	1069.99398	2	.359714E-02	0.7	0.3366E-02	6.41	4.692E-04	.231E+00		1.2
9	8	E	9	7	E	1070.02629	0	.256624E-02	0.7	0.2445E-02	4.74	3.311E-04	-.414E+00	-.352E+00	1.7
8	8	E	8	7	E	1070.05538	-2	.144578E-02	0.7	0.1336E-02	7.58	4.198E-04	-.386E+00	-.144E+00	1.6
23	11	E	24	10	E	1070.13035	1			0.2812E-03					
17	6	E	16	5	E	1070.23067	-13			0.1164E-01					
35	4	A_2	34	3	A_1	1070.23638	4	.687854E-02	0.8	0.3352E-02	2.53	2.907E-04			1.0
35	4	A_1	34	3	A_2	1070.23638	4			0.3352E-02					
15	10	A_2	16	9	A_1	1070.29543	-5			0.2986E-03					
15	10	A_1	16	9	A_2	1070.29543	-5			0.2986E-03					
26	5	E	25	4	E	1070.35601	-3	.789910E-02	0.5	0.7641E-02	3.27	3.981E-04			1.0
38	13	A_2	39	12	A_1	1070.37484	-17			0.3236E-04					
38	13	A_1	39	12	A_2	1070.37484	-17			0.3236E-04					
45	3	E	44	2	E	1070.58988	4	.882544E-03	1.3	0.8270E-03	6.29	3.202E-04	.927E-01	.176E+00	1.0
55	2	E	54	1	E	1070.62023	-13			0.1210E-03					
9	7	A_2	8	6	A_1	1070.72735	1	.246542E-01	0.4	0.1255E-01	-1.78	3.051E-04	-.266E+00	-.960E-01	1.6
9	7	A_1	8	6	A_2	1070.72735	1			0.1255E-01					
30	12	E	31	11	E	1070.80002	2			0.1236E-03					
36	4	A_2	35	3	A_1	1070.99726	3	.600240E-02	0.4	0.2963E-02	1.27	2.306E-04		-.432E+00	1.3
36	4	A_1	35	3	A_2	1070.99726	3			0.2963E-02					
18	6	E	17	5	E	1071.05682	3	.117695E-01	0.2	0.1118E-01	5.00	4.095E-04	-.817E-01	-.191E+00	1.2
22	11	E	23	10	E	1071.08587	-31			0.2789E-03					
27	5	E	26	4	E	1071.15009	0	.727898E-02	0.2	0.7042E-02	3.25	3.987E-04	-.237E+00		1.7
14	10	A_2	15	9	A_1	1071.22792	-4			0.2439E-03					

(cont.)

J	K_a	Γ	J'	K'_a	Γ'	$\nu^{a)}$	$\delta_\nu{}^{b)}$	$S_\nu^{\rm exp}(294.45)^{c)}$	$\Delta_S{}^{d)}$	$S_\nu^{\rm calc}(294.45)^{e)}$	$\delta_S{}^{f)}$	$\Gamma_0{}^{g)}$	$\Delta_2{}^{g)}$	$\nu_{\rm VC}{}^{g)}$	$R^{h)}$
	1			2		3	4	5	6	7	8	9	10	11	12
14	10	A_1	15	9	A_2	1071.22792	-4			0.2439E-03					
56	2	E	55	1	E	1071.30503	28			0.9760E-04					
46	3	E	45	2	E	1071.31263	1			0.6983E-03					
37	13	A_1	38	12	A_2	1071.37205	-8			0.3631E-04					
37	13	A_2	38	12	A_1	1071.37205	-8			0.3631E-04					
10	7	A_2	9	6	A_1	1071.58068	1	.243543E-01	0.1	0.1234E-01	-1.34	3.432E-04	-.562E-01	-.178E+00	1.4
10	7	A_1	9	6	A_2	1071.58068	1			0.1234E-01					
37	4	A_2	36	3	A_1	1071.75438	2	.542061E-02	0.6	0.2607E-02	3.83	2.947E-04	.115E+00		1.1
37	4	A_1	36	3	A_2	1071.75438	2			0.2607E-02					
29	12	E	30	11	E	1071.77528	-20			0.1313E-03					
19	6	E	18	5	E	1071.87932	3	.112528E-01	0.2	0.1069E-01	5.03	4.120E-04	-.155E+00		1.2
28	5	E	27	4	E	1071.94052	1	.647969E-02	0.4	0.6459E-02	.32	3.475E-04	-.104E+00	-.250E+00	1.2
57	2	E	56	1	E	1071.98513	-2			0.7836E-04					
21	11	E	22	10	E	1072.03865	-46			0.2725E-03					
11	7	A_2	10	6	A_1	1072.43062	1	.236504E-01	0.3	0.1212E-01	-2.49	3.484E-04	.181E+00	-.282E+00	1.2
11	7	A_1	10	6	A_2	1072.43062	1			0.1212E-01					
38	4	A_2	37	3	A_1	1072.50773	2	.468378E-02	0.3	0.2282E-02	2.54	2.632E-04	-.228E+00	-.392E+00	1.8
38	4	A_1	37	3	A_2	1072.50773	2			0.2282E-02					
58	2	E	57	1	E	1072.66168	11			0.6264E-04					
54	9	E	54	8	E	1072.68214	6			0.6281E-04					
20	6	E	19	5	E	1072.69830	3	.105802E-01	0.1	0.1017E-01	3.91	4.044E-04	-.147E+00	-.260E+00	1.7
29	5	E	28	4	E	1072.72729	1	.611304E-02	0.2	0.5895E-02	3.57	3.081E-04	-.134E+00	-.321E+00	2.0
48	3	E	47	2	E	1072.74660	13			0.4910E-03					
43	14	E	44	13	E	1072.86207	-30			0.8866E-05					
20	11	E	21	10	E	1072.98909	-5			0.2616E-03					
52	9	E	52	8	E	1073.02917	31			0.9451E-04					
12	10	A_1	13	9	A_2	1073.08378	-7			0.1278E-03					
12	10	A_2	13	9	A_1	1073.08378	-7			0.1278E-03					
51	9	E	51	8	E	1073.19733	-3			0.1151E-03					
39	4	A_2	38	3	A_1	1073.25727	0	.407125E-02	0.4	0.1989E-02	2.28	2.627E-04	-.162E+00	-.186E+00	1.2
39	4	A_1	38	3	A_2	1073.25727	0			0.1989E-02					
12	7	A_2	11	6	A_1	1073.27718	2	.235542E-01	0.1	0.1187E-01	-.81	3.503E-04	-.403E-01	-.228E+00	1.2
12	7	A_1	11	6	A_2	1073.27718	2			0.1187E-01					
59	2	E	58	1	E	1073.33396	-2			0.4985E-04					
35	13	A_2	36	12	A_1	1073.35813	-13			0.4484E-04					
35	13	A_1	36	12	A_2	1073.35813	-13			0.4484E-04					
50	9	E	50	8	E	1073.36257	-3			0.1396E-03					
49	3	E	48	2	E	1073.45755	5			0.4089E-03					
30	5	E	29	4	E	1073.51044	5	.554221E-02	1.4	0.5355E-02	3.38	2.901E-04			1.0
21	6	E	20	5	E	1073.51375	2	.908847E-02	1.4	0.9624E-02	-5.89	2.901E-04			1.0
48	9	E	48	8	E	1073.68347	17			0.2025E-03					
27	12	E	28	11	E	1073.71793	-9			0.1441E-03					
47	9	E	47	8	E	1073.83878	1			0.2422E-03					
19	11	E	20	10	E	1073.93616	-7			0.2461E-03					
46	9	E	46	8	E	1073.99095	-4			0.2883E-03					
60	2	E	59	1	E	1074.00295	57			0.3950E-04					
40	4	A_2	39	3	A_1	1074.00295	-7	.353218E-02	0.6	0.1726E-02	2.29	4.221E-04	-.345E+00	-.225E+00	1.6
40	4	A_1	39	3	A_2	1074.00295	-7			0.1726E-02					
11	10	A_1	12	9	A_2	1074.00698	-26			0.7327E-04					
11	10	A_2	12	9	A_1	1074.00698	-26			0.7327E-04					
13	7	A_2	12	6	A_1	1074.12031	0	.230562E-01	0.1	0.1160E-01	-.58	3.684E-04	-.106E+00	-.301E+00	2.5
13	7	A_1	12	6	A_2	1074.12031	0			0.1160E-01					
45	9	E	45	8	E	1074.13989	-6			0.3415E-03					
50	3	E	49	2	E	1074.16462	2			0.3390E-03					
31	5	E	30	4	E	1074.28984	1	.497127E-02	0.4	0.4841E-02	2.62	3.759E-04			1.0
22	6	E	21	5	E	1074.32565	1	.938315E-02	0.2	0.9068E-02	3.36	3.555E-04	-.520E-01	-.392E+00	1.2
34	13	A_1	35	12	A_2	1074.34714	-12			0.4931E-04					
34	13	A_2	35	12	A_1	1074.34714	-12			0.4931E-04					
43	9	E	43	8	E	1074.42818	6			0.4725E-03					
42	9	E	42	8	E	1074.56721	-12	.545872E-03	2.5	0.5518E-03	-1.09	2.233E-04			1.0
26	12	E	27	11	E	1074.68507	3			0.1487E-03					
41	9	E	41	8	E	1074.70328	-1			0.6413E-03					
41	4	A_2	40	3	A_1	1074.74492	-2	.310026E-02	0.6	0.1490E-02	3.88	2.811E-04			1.0
41	4	A_1	40	3	A_2	1074.74492	-2			0.1490E-02					
40	9	E	40	8	E	1074.83599	-1			0.7417E-03					
51	3	E	50	2	E	1074.86775	1			0.2798E-03					
18	11	E	19	10	E	1074.88033	-4			0.2260E-03					
10	10	A_2	11	9	A_1	1074.92796	39			0.2809E-04					
10	10	A_1	11	9	A_2	1074.92796	39			0.2809E-04					
14	7	A_2	13	6	A_1	1074.96003	0	.226893E-01	0.2	0.1128E-01	.54	4.038E-04			1.0
14	7	A_1	13	6	A_2	1074.96003	0			0.1128E-01					
39	9	E	39	8	E	1074.96546	0	.835825E-03	5.8	0.8535E-03	-2.12	1.813E-04			1.0
32	5	E	31	4	E	1075.06560	3	.449886E-02	0.5	0.4355E-02	3.19	2.727E-04	-.189E+00	-.403E+00	1.6
38	9	E	38	8	E	1075.09166	-2	.996161E-03	1.4	0.9773E-03	1.89	2.406E-04			1.0
23	6	E	22	5	E	1075.13400	1	.883646E-02	0.4	0.8502E-02	3.78	3.929E-04			1.0
37	9	E	37	8	E	1075.21464	-2	.111509E-02	0.8	0.1113E-02	.15	2.501E-04	-.174E+00	-.217E+00	1.1
35	9	E	35	8	E	1075.45084	-3	.138961E-02	1.0	0.1423E-02	-2.40	2.024E-04			1.0
36	9	E	36	8	E	1075.33429	-10			0.1262E-02					
42	4	A_2	41	3	A_1	1075.48303	0	.274174E-02	0.6	0.1281E-02	6.58	2.935E-04			1.0
42	4	A_1	41	3	A_2	1075.48303	0			0.1281E-02					
34	9	E	34	8	E	1075.56412	1	.157533E-02	1.0	0.1596E-02	-1.31	4.661E-04	.139E+01	.550E+00	1.9
25	12	E	26	11	E	1075.64907	-16			0.1517E-03					

(cont.)

J	K_a	Γ	J'	K'_a	Γ'	$\nu^{a)}$	$\delta_\nu{}^{b)}$	$S_\nu^{\rm exp}(294.45)^{c)}$	$\Delta_S{}^{d)}$	$S_\nu^{\rm calc}(294.45)^{e)}$	$\delta_S{}^{f)}$	$\Gamma_0{}^{g)}$	$\Delta_2{}^{g)}$	$\nu_{\rm VC}{}^{g)}$	$R^{h)}$
1			2			3	4	5	6	7	8	9	10	11	12
33	9	E	33	8	E	1075.67411	-1	.181548E-02	0.7	0.1781E-02	1.93	3.630E-04	-.335E+00		1.3
32	9	E	32	8	E	1075.78087	0	.188043E-02	2.9	0.1976E-02	-5.06	2.397E-04			1.0
15	7	A_2	14	6	A_1	1075.79632	1	.217608E-01	0.3	0.1094E-01	-.52	4.691E-04			1.0
15	7	A_1	14	6	A_2	1075.79632	1			0.1094E-01					
17	11	E	18	10	E	1075.82121	-35			0.2014E-03					
33	5	E	32	4	E	1075.83762	1	.402410E-02	0.5	0.3900E-02	3.08	2.756E-04	.890E-01	-.430E+00	1.3
40	14	E	41	13	E	1075.88432	-35			0.1339E-04					
40	14	E	41	13	E	1075.88442	-25			0.1339E-04					
31	9	E	31	8	E	1075.88442	3			0.2180E-02					
31	9	E	31	8	E	1075.88432	-7			0.2180E-02					
24	6	E	23	5	E	1075.93877	0	.823338E-02	0.3	0.7934E-02	3.63	3.980E-04			1.0
30	9	E	30	8	E	1075.98462	-5	.236146E-02	0.4	0.2392E-02	-1.28	4.009E-04			1.0
29	9	E	29	8	E	1076.08172	1	.267088E-02	0.4	0.2609E-02	2.33	3.630E-04			1.0
28	9	E	28	8	E	1076.17548	-3	.267652E-02	1.0	0.2828E-02	-5.67	3.498E-04			1.0
43	4	A_2	42	3	A_1	1076.21732	6	.230225E-02	0.6	0.1096E-02	4.81	2.633E-04	-.106E+00	-.315E+00	1.2
43	4	A_1	42	3	A_2	1076.21732	6			0.1096E-02					
27	9	E	27	8	E	1076.26606	-1	.292794E-02	0.9	0.3048E-02	-4.09	3.821E-04			1.0
32	13	A_2	33	12	A_1	1076.31744	41			0.5831E-04					
32	13	A_1	33	12	A_2	1076.31744	41			0.5831E-04					
47	15	E	48	14	E	1076.33955	-173			0.2412E-05					
26	9	E	26	8	E	1076.35340	1	.320346E-02	0.8	0.3263E-02	-1.86	3.697E-04	.182E+00	-.380E+00	1.1
25	9	E	25	8	E	1076.43745	-3	.337832E-02	0.5	0.3470E-02	-2.72	3.509E-04			1.0
24	9	E	24	8	E	1076.51832	-1	.358080E-02	0.3	0.3665E-02	-2.35	3.428E-04	-.582E-01	-.339E+00	1.3
23	9	E	23	8	E	1076.59594	0	.382827E-02	0.6	0.3842E-02	-.37	3.550E-04			1.0
34	5	E	33	4	E	1076.60583	-10	.347850E-02	3.2	0.3476E-02	.07	3.398E-04			1.0
24	12	E	25	11	E	1076.61005	-50			0.1529E-03					
16	7	A_2	15	6	A_1	1076.62914	1	.212258E-01	0.2	0.1056E-01	.51	4.018E-04			1.0
16	7	A_1	15	6	A_2	1076.62914	1			0.1056E-01					
22	9	E	22	8	E	1076.67033	2	.391825E-02	2.2	0.3997E-02	-2.01	3.864E-04			1.0
25	6	E	24	5	E	1076.74034	37	.104814E-01	0.7	0.7369E-02	-9.64	7.155E-04			1.0
21	9	E	21	8	E	1076.74034	-111			0.4123E-02					
16	11	E	17	10	E	1076.75953	-24			0.1730E-03					
20	9	E	20	8	E	1076.80933	-2	.406976E-02	1.5	0.4215E-02	-3.57	3.751E-04			1.0
19	9	E	19	8	E	1076.87399	-3	.429858E-02	0.5	0.4266E-02	.76	4.062E-04			1.0
18	9	E	18	8	E	1076.93557	12			0.4270E-02					
44	4	A_2	43	3	A_1	1076.94766	3	.195278E-02	2.5	0.9333E-03	4.42	3.410E-04			1.0
44	4	A_1	43	3	A_2	1076.94766	3			0.9333E-03					
54	3	E	53	2	E	1076.95306	-20			0.1532E-03					
17	9	E	17	8	E	1076.99362	-3	.442197E-02	1.1	0.4220E-02	4.57	4.049E-04			1.0
16	9	E	16	8	E	1077.04856	-5	.431359E-02	0.3	0.4109E-02	4.74	4.375E-04			1.0
15	9	E	15	8	E	1077.10029	-5	.397894E-02	0.6	0.3931E-02	1.21	5.101E-04			1.0
8	8	E	7	7	E	1077.14342	0	.114499E-01	0.3	0.1113E-01	2.79	2.739E-04			1.0
14	9	E	14	8	E	1077.14888	5	.374112E-02	1.0	0.3677E-02	1.72	3.558E-04			1.0
13	9	E	13	8	E	1077.19404	-5	.350937E-02	0.4	0.3339E-02	4.84	4.247E-04			1.0
12	9	E	12	8	E	1077.23607	-5	.296327E-02	0.6	0.2909E-02	1.82	3.486E-04	-.219E+00	-.459E+00	1.5
11	9	E	11	8	E	1077.27491	0	.244546E-02	0.7	0.2377E-02	2.82	3.891E-04	-.156E+00		1.1
31	13	A_1	32	12	A_2	1077.29789	10			0.6266E-04					
31	13	A_2	32	12	A_1	1077.29789	10			0.6266E-04					
10	9	E	10	8	E	1077.31047	0	.172107E-02	0.5	0.1727E-02	-.36	2.915E-04	-.153E+00	-.398E+00	1.4
9	9	E	9	8	E	1077.34272	-7	.984310E-03	0.9	0.9436E-03	4.14	4.316E-04	.891E-01	-.428E+00	1.1
35	5	E	34	4	E	1077.37051	1	.315603E-02	0.5	0.3084E-02	2.29	3.346E-04	-.141E+00		1.1
17	7	A_2	16	6	A_1	1077.45850	1	.203616E-01	0.2	0.1015E-01	.31	3.955E-04	.595E-01		1.0
17	7	A_1	16	6	A_2	1077.45850	1			0.1015E-01					
26	6	E	25	5	E	1077.53758	2	.717708E-02	0.4	0.6811E-02	5.10	4.111E-04	-.710E-01		1.0
23	12	E	24	11	E	1077.56889	-11			0.1521E-03					
45	4	A_2	44	3	A_1	1077.67419	8	.169509E-02	1.0	0.7912E-03	6.64	3.025E-04	-.179E+00		1.1
45	4	A_1	44	3	A_2	1077.67419	8			0.7912E-03					
15	11	E	16	10	E	1077.69505	5			0.1413E-03					
38	14	E	39	13	E	1077.88644	14			0.1710E-04					
9	8	E	8	7	E	1077.99994	-1	.110236E-01	0.2	0.1086E-01	1.48	2.786E-04	-.109E+00	-.327E+00	1.7
36	5	E	35	4	E	1078.13113	-20	.291584E-02	0.7	0.2723E-02	6.62	5.623E-04	-.774E+00	-.514E+00	2.2
18	7	A_2	17	6	A_1	1078.28437	0	.197469E-01	0.1	0.9713E-02	1.62	4.054E-04	-.126E+00		1.5
18	7	A_1	17	6	A_2	1078.28437	0			0.9713E-02					
56	3	E	55	2	E	1078.32295	-59			0.1003E-03					
27	6	E	26	5	E	1078.33154	1	.647304E-02	0.4	0.6265E-02	3.21	3.244E-04	-.163E+00	-.397E+00	1.5
46	4	A_2	45	3	A_1	1078.39669	-1	.143772E-02	0.8	0.6678E-03	7.10	3.434E-04	-.175E+00	.135E+00	1.1
46	4	A_1	45	3	A_2	1078.39669	-1			0.6678E-03					
22	12	E	23	11	E	1078.52453	-4			0.1489E-03					
14	11	E	15	10	E	1078.62717	-6			0.1078E-03					
10	8	E	9	7	E	1078.85312	1	.108850E-01	0.3	0.1060E-01	2.58	3.310E-04	-.219E+00		1.3
37	5	E	36	4	E	1078.88840	1	.252676E-02	0.7	0.2393E-02	5.29	3.325E-04	-.208E+00		1.2
57	3	E	56	2	E	1079.00259	-4			0.8059E-04					
19	7	A_2	18	6	A_1	1079.10674	0	.189130E-01	0.2	0.9254E-02	2.14	4.098E-04			1.0
19	7	A_1	18	6	A_2	1079.10674	0			0.9254E-02					
47	4	A_2	46	3	A_1	1079.11547	10			0.5611E-03					
47	4	A_1	46	3	A_2	1079.11547	10			0.5611E-03					
28	6	E	27	5	E	1079.12188	1	.593848E-02	0.5	0.5736E-02	3.40	3.809E-04			1.0
29	13	A_2	30	12	A_1	1079.25110	15			0.7050E-04					
29	13	A_1	30	12	A_2	1079.25110	15			0.7050E-04					
21	12	E	22	11	E	1079.47725	2			0.1433E-03					
57	10	A_1	57	9	A_2	1079.49430	-98			0.2274E-04					
57	10	A_2	57	9	A_1	1079.49430	-98			0.2274E-04					

(cont.)

J	K_a	Γ	J'	K'_a	Γ'	$\nu^{a)}$	$\delta_\nu{}^{b)}$	$S_\nu^{\rm exp}(294.45)^{c)}$	$\Delta_S{}^{d)}$	$S_\nu^{\rm calc}(294.45)^{e)}$	$\delta_S{}^{f)}$	$\Gamma_0{}^{g)}$	$\Delta_2{}^{g)}$	$\nu_{\rm VC}{}^{g)}$	$R^{h)}$
	1			2		3	4	5	6	7	8	9	10	11	12
38	5	E	37	4	E	1079.64163	-4	.203259E-02	0.8	0.2094E-02	-3.00	3.025E-04	-.582E-01		1.0
11	8	E	10	7	E	1079.70290	0	.102762E-01	0.2	0.1035E-01	-.70	2.965E-04		-.427E+00	1.5
48	4	A_2	47	3	A_1	1079.83011	0			0.4693E-03					
48	4	A_1	47	3	A_2	1079.83011	0			0.4693E-03					
55	10	A_1	55	9	A_2	1079.86142	-18			0.3514E-04					
55	10	A_2	55	9	A_1	1079.86142	-18			0.3514E-04					
36	14	E	37	13	E	1079.87764	42			0.2126E-04					
29	6	E	28	5	E	1079.90857	0	.553622E-02	0.6	0.5227E-02	5.58	3.869E-04			1.0
20	7	A_2	19	6	A_1	1079.92563	2	.175356E-01	0.2	0.8777E-02	-.11	4.070E-04			1.0
20	7	A_1	19	6	A_2	1079.92563	2			0.8777E-02					
28	13	A_2	29	12	A_1	1080.22304	-29			0.7373E-04					
28	13	A_1	29	12	A_2	1080.22304	-29			0.7373E-04					
59	3	E	58	2	E	1080.34840	-19			0.5137E-04					
39	5	E	38	4	E	1080.39104	-11	.195779E-02	1.1	0.1823E-02	6.88	2.584E-04	-.256E+00	-.338E+00	1.1
20	12	E	21	11	E	1080.42700	3			0.1351E-03					
12	11	E	13	10	E	1080.48289	28			0.4235E-04					
49	4	A_1	48	3	A_2	1080.54081	-10			0.3908E-03					
49	4	A_2	48	3	A_1	1080.54081	-10			0.3908E-03					
12	8	E	11	7	E	1080.54930	0	.103854E-01	0.4	0.1008E-01	2.95	3.665E-04			1.0
30	6	E	29	5	E	1080.69162	2	.505276E-02	0.3	0.4741E-02	6.17	3.293E-04	-.117E+00	-.341E+00	1.7
50	10	A_1	50	9	A_2	1080.72030	3			0.9634E-04					
50	10	A_2	50	9	A_1	1080.72030	3			0.9634E-04					
21	7	A_2	20	6	A_1	1080.74095	0	.166303E-01	0.2	0.8287E-02	.34	3.658E-04		-.280E+00	1.2
21	7	A_1	20	6	A_2	1080.74095	0			0.8287E-02					
35	14	E	36	13	E	1080.86843	-20			0.2346E-04					
49	10	A_2	49	9	A_1	1080.88221	-1			0.1163E-03					
49	10	A_1	49	9	A_2	1080.88221	-1			0.1163E-03					
48	10	A_2	48	9	A_1	1081.04087	-5			0.1396E-03					
48	10	A_1	48	9	A_2	1081.04087	-5			0.1396E-03					
40	5	E	39	4	E	1081.13685	3	.158783E-02	0.7	0.1580E-02	.48	1.972E-04		-.434E+00	1.2
27	13	A_1	28	12	A_2	1081.19273	-17			0.7632E-04					
27	13	A_2	28	12	A_1	1081.19273	-17			0.7632E-04					
47	10	A_1	47	9	A_2	1081.19634	-3			0.1670E-03					
47	10	A_2	47	9	A_1	1081.19634	-3			0.1670E-03					
50	4	A_2	49	3	A_1	1081.24776	1			0.3239E-03					
50	4	A_1	49	3	A_2	1081.24776	1			0.3239E-03					
46	10	A_1	46	9	A_2	1081.34855	-1			0.1987E-03					
46	10	A_2	46	9	A_1	1081.34855	-1			0.1987E-03					
19	12	E	20	11	E	1081.37372	-6			0.1242E-03					
13	8	E	12	7	E	1081.39227	-1	.964268E-02	0.3	0.9792E-02	-1.55	3.709E-04	.170E+00	-.216E+00	1.1
31	6	E	30	5	E	1081.47097	1	.443701E-02	0.3	0.4280E-02	3.54	2.896E-04	-.128E+00	-.311E+00	1.4
45	10	A_2	45	9	A_1	1081.49776	27			0.2353E-03					
45	10	A_1	45	9	A_2	1081.49776	27			0.2353E-03					
22	7	A_2	21	6	A_1	1081.55276	1	.158396E-01	0.2	0.7788E-02	1.66	3.973E-04			1.0
22	7	A_1	21	6	A_2	1081.55276	1			0.7788E-02					
44	10	A_2	44	9	A_1	1081.64320	2			0.2773E-03					
44	10	A_1	44	9	A_2	1081.64320	2			0.2773E-03					
61	3	E	60	2	E	1081.67802	-17			0.3218E-04					
43	10	A_1	43	9	A_2	1081.78561	0			0.3253E-03					
43	10	A_2	43	9	A_1	1081.78561	0			0.3253E-03					
41	5	E	40	4	E	1081.87869	3	.140469E-02	0.6	0.1364E-02	2.93	2.475E-04	-.960E-01		1.0
42	10	A_1	42	9	A_2	1081.92477	-3			0.3797E-03					
42	10	A_2	42	9	A_1	1081.92477	-3			0.3797E-03					
51	4	A_1	50	3	A_2	1081.95075	14			0.2673E-03					
51	4	A_2	50	3	A_1	1081.95075	14			0.2673E-03					
41	10	A_2	41	9	A_1	1082.06072	-2	.876079E-03	1.6	0.4411E-03	-.69	3.682E-04			1.0
41	10	A_1	41	9	A_2	1082.06072	-2			0.4411E-03					
40	10	A_2	40	9	A_1	1082.19341	-2	.101750E-02	1.5	0.5098E-03	-.21	2.782E-04			1.0
40	10	A_1	40	9	A_2	1082.19341	-2			0.5098E-03					
14	8	E	13	7	E	1082.23185	0	.928328E-02	0.3	0.9485E-02	-2.17	4.259E-04			1.0
32	6	E	31	5	E	1082.24662	-1	.395202E-02	0.9	0.3846E-02	2.69	3.361E-04			1.0
39	10	A_2	39	9	A_1	1082.32285	-2			0.5864E-03					
39	10	A_1	39	9	A_2	1082.32285	-2			0.5864E-03					
62	3	E	61	2	E	1082.33697	15			0.2530E-04					
23	7	A_2	22	6	A_1	1082.36100	1	.145284E-01	0.2	0.7286E-02	-.29	3.373E-04	-.145E+00	-.445E+00	1.9
23	7	A_1	22	6	A_2	1082.36100	1			0.7286E-02					
38	10	A_2	38	9	A_1	1082.44907	0	.132836E-02	0.9	0.6710E-03	-1.02	2.152E-04	-.183E+00		1.1
38	10	A_1	38	9	A_2	1082.44907	0			0.6710E-03					
37	10	A_2	37	9	A_1	1082.57197	-5			0.7639E-03					
37	10	A_1	37	9	A_2	1082.57197	-5			0.7639E-03					
42	5	E	41	4	E	1082.61672	5	.117214E-02	1.0	0.1171E-02	.08	2.604E-04	-.417E+00	-.351E+00	1.6
52	4	A_2	51	3	A_1	1082.64942	-6			0.2196E-03					
52	4	A_1	51	3	A_2	1082.64942	-7			0.2196E-03					
36	10	A_2	36	9	A_1	1082.69172	-1	.170448E-02	1.5	0.8651E-03	-1.51	3.032E-04			1.0
36	10	A_1	36	9	A_2	1082.69172	-1			0.8651E-03					
35	10	A_2	35	9	A_1	1082.80819	-1	.193832E-02	0.8	0.9746E-03	-.57	3.149E-04	-.388E+00		1.1
35	10	A_1	35	9	A_2	1082.80819	-1			0.9746E-03					
33	14	E	34	13	E	1082.84330	4			0.2793E-04					
34	10	A_2	34	9	A_1	1082.92133	-9	.215705E-02	0.7	0.1092E-02	-1.26	2.757E-04	-.269E+00	-.389E+00	1.4
34	10	A_1	34	9	A_2	1082.92133	-9			0.1092E-02					
33	6	E	32	5	E	1083.01860	2	.362427E-02	0.3	0.3440E-02	5.10	3.366E-04			1.0
33	10	A_2	33	9	A_1	1083.03137	-3	.245686E-02	0.5	0.1217E-02	.91	3.841E-04			1.0

(cont.)

J	K_a	Γ	J'	K'_a	Γ'	$\nu^{a)}$	$\delta_\nu{}^{b)}$	$S_\nu^{\rm exp}(294.45)^{c)}$	$\Delta_S{}^{d)}$	$S_\nu^{\rm calc}(294.45)^{e)}$	$\delta_S{}^{f)}$	$\Gamma_0{}^{g)}$	$\Delta_2{}^{g)}$	$\nu_{\rm VC}{}^{g)}$	$R^{h)}$
	1			2		3	4	5	6	7	8	9	10	11	12
33	10	A_1	33	9	A_2	1083.03137	-3			0.1217E-02					
15	8	E	14	7	E	1083.06798	1	.898461E-02	0.4	0.9155E-02	-1.90	5.749E-04			1.0
25	13	A_2	26	12	A_1	1083.12349	-3			0.7895E-04					
25	13	A_1	26	12	A_2	1083.12349	-3			0.7895E-04					
32	10	A_2	32	9	A_1	1083.13812	-2	.267566E-02	0.6	0.1349E-02	-.84	3.293E-04	-.450E+00	-.381E+00	1.6
32	10	A_1	32	9	A_2	1083.13812	-2			0.1349E-02					
24	7	A_2	23	6	A_1	1083.16567	2	.134162E-01	0.2	0.6784E-02	-1.13	4.250E-04			1.0
24	7	A_1	23	6	A_2	1083.16567	2			0.6784E-02					
31	10	A_2	31	9	A_1	1083.24161	-3	.303529E-02	0.6	0.1487E-02	2.04	6.312E-04	.306E+00	.203E+01	1.4
31	10	A_1	31	9	A_2	1083.24161	-3			0.1487E-02					
30	10	A_2	30	9	A_1	1083.34189	-1	.334294E-02	0.9	0.1629E-02	2.56	5.351E-04			1.0
30	10	A_1	30	9	A_2	1083.34189	-1			0.1629E-02					
43	5	E	42	4	E	1083.35085	4	.103783E-02	1.8	0.1001E-02	3.51	1.738E-04			1.0
29	10	A_2	29	9	A_1	1083.43891	-1	.358829E-02	0.6	0.1774E-02	1.14	3.635E-04			1.0
29	10	A_1	29	9	A_2	1083.43891	-1			0.1774E-02					
28	10	A_1	28	9	A_2	1083.53246	-24			0.1920E-02					
28	10	A_2	28	9	A_1	1083.53246	-24			0.1920E-02					
27	10	A_2	27	9	A_1	1083.62324	-1	.409820E-02	0.6	0.2064E-02	-.74	3.798E-04			1.0
27	10	A_1	27	9	A_2	1083.62324	-1			0.2064E-02					
64	3	E	63	2	E	1083.64161	-3			0.1544E-04					
26	10	A_2	26	9	A_1	1083.71053	-2	.439017E-02	0.2	0.2205E-02	-.46	3.544E-04	-.166E+00	-.189E+00	1.3
26	10	A_1	26	9	A_2	1083.71053	-2			0.2205E-02					
34	6	E	33	5	E	1083.78680	-2	.316032E-02	0.7	0.3062E-02	3.11	3.132E-04			1.0
25	10	A_2	25	9	A_1	1083.79460	-2	.469598E-02	0.5	0.2339E-02	.38	4.240E-04			1.0
25	10	A_1	25	9	A_2	1083.79460	-2			0.2339E-02					
24	10	A_2	24	9	A_1	1083.87542	-4	.480506E-02	0.6	0.2463E-02	-2.52	3.601E-04			1.0
24	10	A_1	24	9	A_2	1083.87542	-4			0.2463E-02					
16	8	E	15	7	E	1083.90065	0	.868818E-02	0.2	0.8804E-02	-1.33	4.257E-04			1.0
23	10	A_2	23	9	A_1	1083.95300	-5	.515408E-02	0.7	0.2573E-02	.15	3.656E-04			1.0
23	10	A_1	23	9	A_2	1083.95300	-5			0.2573E-02					
25	7	A_2	24	6	A_1	1083.96674	0	.126559E-01	0.3	0.6288E-02	.64	3.850E-04			1.0
25	7	A_1	24	6	A_2	1083.96674	0			0.6288E-02					
22	10	A_2	22	9	A_1	1084.02743	2	.518198E-02	0.7	0.2666E-02	-2.88	2.813E-04		-.444E+00	1.1
22	10	A_1	22	9	A_2	1084.02743	2		0.7	0.2666E-02		2.813E-04		-.444E+00	1.1
54	4	A_1	53	3	A_2	1084.03509	-11			0.1463E-03					
54	4	A_2	53	3	A_1	1084.03509	-11			0.1463E-03					
44	5	E	43	4	E	1084.08112	4	.885729E-03	3.0	0.8524E-03	3.77	3.064E-04			1.0
21	10	A_2	21	9	A_1	1084.09856	2	.558037E-02	0.3	0.2736E-02	1.93	4.445E-04			1.0
21	10	A_1	21	9	A_2	1084.09856	2			0.2736E-02					
20	10	A_2	20	9	A_1	1084.16647	5	.550824E-02	0.2	0.2781E-02	-.98	4.197E-04			1.0
20	10	A_1	20	9	A_2	1084.16647	5			0.2781E-02					
16	12	E	17	11	E	1084.19641	-9			0.7799E-04					
19	10	A_2	19	9	A_1	1084.23110	2	.581367E-02	0.3	0.2795E-02	3.85	3.835E-04	-.156E+00	-.290E+00	1.3
19	10	A_1	19	9	A_2	1084.23110	2			0.2795E-02					
18	10	A_2	18	9	A_1	1084.29253	3	.549260E-02	0.2	0.2773E-02	-.98	3.798E-04	-.271E+00	-.926E-01	3.0
18	10	A_1	18	9	A_2	1084.29253	3			0.2773E-02					
17	10	A_2	17	9	A_1	1084.35073	5	.553348E-02	0.3	0.2711E-02	2.03	4.015E-04			1.0
17	10	A_1	17	9	A_2	1084.35073	5			0.2711E-02					
16	10	A_2	16	9	A_1	1084.40567	3	.503889E-02	0.4	0.2602E-02	-3.28	3.714E-04			1.0
16	10	A_1	16	9	A_2	1084.40567	3			0.2602E-02					
39	15	E	40	14	E	1084.42861	-79			0.7622E-05					
15	10	A_2	15	9	A_1	1084.45737	2	.478113E-02	0.3	0.2442E-02	-2.16	3.473E-04	.379E-01	-.366E+00	1.3
15	10	A_1	15	9	A_2	1084.45737	2			0.2442E-02					
14	10	A_2	14	9	A_1	1084.50587	3	.447677E-02	0.3	0.2225E-02	.60	3.341E-04	-.109E+00	-.436E+00	1.6
14	10	A_1	14	9	A_2	1084.50587	3			0.2225E-02					
13	10	A_2	13	9	A_1	1084.55123	14	.646086E-02	0.2	0.1944E-02	-2.16	3.234E-04	-.561E-01	-.340E+00	1.8
13	10	A_1	13	9	A_2	1084.55123	14			0.1944E-02					
35	6	E	34	5	E	1084.55123	-9			0.2713E-02					
12	10	A_2	12	9	A_1	1084.59314	3	.328559E-02	0.5	0.1591E-02	3.16	3.692E-04			1.0
12	10	A_1	12	9	A_2	1084.59314	3			0.1591E-02					
11	10	A_2	11	9	A_1	1084.63193	4	.235004E-02	0.3	0.1158E-02	1.47	3.390E-04	-.698E-01	-.222E+00	1.2
11	10	A_1	11	9	A_2	1084.63193	4			0.1158E-02					
10	10	A_1	10	9	A_2	1084.66741	-4			0.6326E-03					
10	10	A_2	10	9	A_1	1084.66741	-4			0.6326E-03					
55	4	A_1	54	3	A_2	1084.72249	48			0.1186E-03					
55	4	A_2	54	3	A_1	1084.72249	47			0.1186E-03					
17	8	E	16	7	E	1084.72993	7	.834884E-02	0.5	0.8433E-02	-1.01	2.867E-04		-.565E+00	1.3
26	7	A_2	25	6	A_1	1084.76428	6	.117407E-01	0.1	0.5801E-02	1.19	3.319E-04	-.842E-01	-.248E+00	2.5
26	7	A_1	25	6	A_2	1084.76428	6			0.5801E-02					
31	14	E	32	13	E	1084.80745	55			0.3219E-04					
45	5	E	44	4	E	1084.80745	-1	.723508E-03	1.6	0.7222E-03	.18	4.101E-04			1.0
23	13	A_1	24	12	A_2	1085.04253	-17			0.7727E-04					
23	13	A_2	24	12	A_1	1085.04253	-17			0.7727E-04					
15	12	E	16	11	E	1085.13143	-2			0.5949E-04					
9	9	E	8	8	E	1085.31470	3	.830682E-02	3.4	0.8916E-02	-7.34	1.844E-04			1.0
56	4	A_2	55	3	A_1	1085.40476	-2			0.9574E-04					
56	4	A_1	55	3	A_2	1085.40476	-2			0.9574E-04					
46	5	E	45	4	E	1085.52996	2	.656146E-03	2.0	0.6092E-03	7.15	2.458E-04	-.494E+00	.326E+00	1.2
27	7	A_2	26	6	A_1	1085.55801	-7			0.5327E-02					
27	7	A_1	26	6	A_2	1085.55801	-7			0.5327E-02					
30	14	E	31	13	E	1085.78487	31			0.3410E-04					
22	13	A_2	23	12	A_1	1085.99740	-56			0.7452E-04					

(cont.)

J	K_a	Γ	J'	K'_a	Γ'	$\nu^{a)}$	$\delta_\nu{}^{b)}$	$S_\nu^{\rm exp}(294.45)^{c)}$	$\Delta_S{}^{d)}$	$S_\nu^{\rm calc}(294.45)^{e)}$	$\delta_S{}^{f)}$	$\Gamma_0{}^{g)}$	$\Delta_2{}^{g)}$	$\nu_{\rm VC}{}^{g)}$	$R^{h)}$
	1			2		3	4	5	6	7	8	9	10	11	12
22	13	A_1	23	12	A_2	1085.99740	-56			0.7452E-04					
37	6	E	36	5	E	1086.06917	13	.202456E-02	1.4	0.2101E-02	-3.79	4.687E-04			1.0
57	4	A_1	56	3	A_2	1086.08360	13			0.7694E-04					
57	4	A_2	56	3	A_1	1086.08360	13			0.7694E-04					
10	9	E	9	8	E	1086.16769	5	.887897E-02	0.5	0.8651E-02	2.57	3.304E-04			1.0
47	5	E	46	4	E	1086.24851	1			0.5116E-03					
28	7	A_2	27	6	A_1	1086.34838	6	.986456E-02	0.2	0.4869E-02	1.28	3.266E-04	-.242E-01	-.299E+00	1.3
28	7	A_1	27	6	A_2	1086.34838	6			0.4869E-02					
19	8	E	18	7	E	1086.37786	5	.767580E-02	0.2	0.7642E-02	.44	3.351E-04	-.835E-01	-.532E+00	1.7
58	4	A_2	57	3	A_1	1086.75818	10			0.6156E-04					
58	4	A_1	57	3	A_2	1086.75818	10			0.6156E-04					
38	6	E	37	5	E	1086.82236	12	.181127E-02	0.8	0.1837E-02	-1.40	3.018E-04			1.0
21	13	A_1	22	12	A_2	1086.95020	-13			0.7040E-04					
21	13	A_2	22	12	A_1	1086.95020	-13			0.7040E-04					
48	5	E	47	4	E	1086.96317	5			0.4277E-03					
11	9	E	10	8	E	1087.01729	4	.833229E-02	0.3	0.8393E-02	-.73	2.809E-04		-.353E+00	1.5
29	7	A_2	28	6	A_1	1087.13498	8	.900081E-02	0.3	0.4430E-02	1.56	3.708E-04	.793E-01	-.305E+00	1.3
29	7	A_1	28	6	A_2	1087.13498	8			0.4430E-02					
20	8	E	19	7	E	1087.19659	6	.738959E-02	0.3	0.7228E-02	2.18	3.902E-04	-.533E-01	-.323E+00	1.4
59	4	A_2	58	3	A_1	1087.42861	2			0.4905E-04					
59	4	A_1	58	3	A_2	1087.42861	2			0.4905E-04					
39	6	E	38	5	E	1087.57170	6	.155601E-02	0.9	0.1598E-02	-2.69	3.875E-04	-.148E+00		1.1
49	5	E	48	4	E	1087.67385	5			0.3560E-03					
28	14	E	29	13	E	1087.73142	-8			0.3714E-04					
12	9	E	11	8	E	1087.86350	5	.820262E-02	0.2	0.8133E-02	.85	3.087E-04	-.786E-01	-.422E+00	1.6
20	13	A_2	21	12	A_1	1087.89982	5			0.6489E-04					
20	13	A_1	21	12	A_2	1087.89982	5			0.6489E-04					
30	7	A_2	29	6	A_1	1087.91790	7	.823692E-02	0.2	0.4012E-02	2.47	3.261E-04	-.883E-01	-.145E+00	1.3
30	7	A_1	29	6	A_2	1087.91790	7			0.4012E-02					
12	12	E	13	11	E	1087.91790	-33			0.8913E-05					
21	8	E	20	7	E	1088.01176	4	.705397E-02	0.2	0.6807E-02	3.50	3.949E-04	-.441E-01	-.212E+00	1.1
60	4	A_1	59	3	A_2	1088.09497	-1			0.3890E-04					
60	4	A_2	59	3	A_1	1088.09497	-1			0.3890E-04					
50	11	E	50	10	E	1088.11616	-5			0.6336E-04					
40	6	E	39	5	E	1088.31709	-14			0.1384E-02					
50	5	E	49	4	E	1088.38048	-2			0.2950E-03					
48	11	E	48	10	E	1088.43689	5			0.9178E-04					
47	11	E	47	10	E	1088.59226	0			0.1097E-03					
31	7	A_2	30	6	A_1	1088.69716	8	.735526E-02	0.4	0.3617E-02	1.65	3.377E-04			1.0
31	7	A_1	30	6	A_2	1088.69716	8			0.3617E-02					
13	9	E	12	8	E	1088.70631	5	.777370E-02	0.4	0.7864E-02	-1.17	4.032E-04			1.0
46	11	E	46	10	E	1088.74439	-5			0.1305E-03					
61	4	A_1	60	3	A_2	1088.75714	-10			0.3072E-04					
61	4	A_2	60	3	A_1	1088.75714	-11			0.3072E-04					
22	8	E	21	7	E	1088.82344	7	.661340E-02	0.4	0.6382E-02	3.49	4.336E-04			1.0
19	13	A_1	20	12	A_2	1088.84603	-26			0.5804E-04					
19	13	A_2	20	12	A_1	1088.84603	-26			0.5804E-04					
45	11	E	45	10	E	1088.89344	8			0.1544E-03					
44	11	E	44	10	E	1089.03904	1			0.1820E-03					
41	6	E	40	5	E	1089.05908	10	.118888E-02	1.0	0.1193E-02	-.37	2.377E-04			1.0
51	5	E	50	4	E	1089.08326	3			0.2433E-03					
43	11	E	43	10	E	1089.18144	0			0.2133E-03					
42	11	E	42	10	E	1089.32061	-1			0.2489E-03					
34	15	E	35	14	E	1089.39824	-3			0.1267E-04					
62	4	A_2	61	3	A_1	1089.41544	8			0.2416E-04					
62	4	A_1	61	3	A_2	1089.41544	8			0.2416E-04					
41	11	E	41	10	E	1089.45626	-28			0.2889E-03					
32	7	A_2	31	6	A_1	1089.47273	9	.664305E-02	0.4	0.3246E-02	2.28	3.433E-04	-.114E+00		1.1
32	7	A_1	31	6	A_2	1089.47273	9			0.3246E-02					
14	9	E	13	8	E	1089.54569	6	.764259E-02	0.2	0.7585E-02	.75	3.230E-04	-.101E+00	-.398E+00	1.7
40	11	E	40	10	E	1089.58923	2			0.3338E-03					
23	8	E	22	7	E	1089.63152	5	.605359E-02	0.4	0.5958E-02	1.59	3.435E-04	-.190E+00	-.374E+00	1.6
26	14	E	27	13	E	1089.66703	-14			0.3865E-04					
39	11	E	39	10	E	1089.71857	-7			0.3836E-03					
52	5	E	51	4	E	1089.78201	5			0.1998E-03					
18	13	A_2	19	12	A_1	1089.78972	-14			0.4997E-04					
18	13	A_1	19	12	A_2	1089.78972	-14			0.4997E-04					
42	6	E	41	5	E	1089.79688	-2	.110088E-02	1.1	0.1024E-02	6.97	3.704E-04			1.0
38	11	E	38	10	E	1089.84481	-2			0.4386E-03					
63	4	A_1	62	3	A_2	1090.06950	19			0.1892E-04					
63	4	A_2	62	3	A_1	1090.06950	18			0.1892E-04					
36	11	E	36	10	E	1090.08757	11			0.5646E-03					
35	11	E	35	10	E	1090.20400	9	.631594E-03	1.3	0.6354E-03	-.61	1.592E-04		-.564E+00	1.1
33	7	A_2	32	6	A_1	1090.24456	7	.593120E-02	0.6	0.2900E-02	2.23	3.351E-04			1.0
33	7	A_1	32	6	A_2	1090.24456	7			0.2900E-02					
34	11	E	34	10	E	1090.31713	1	.694168E-03	2.4	0.7113E-03	-2.47	4.752E-04			1.0
15	9	E	14	8	E	1090.38165	7	.770295E-02	0.6	0.7293E-02	5.32	4.760E-04	.364E+00		1.2
33	11	E	33	10	E	1090.42720	11	.760362E-03	3.1	0.7918E-03	-4.13	2.303E-04			1.0
24	8	E	23	7	E	1090.43606	7	.575071E-02	0.4	0.5536E-02	3.73	3.951E-04			1.0
53	5	E	52	4	E	1090.47675	7			0.1634E-03					
43	6	E	42	5	E	1090.53117	22			0.8751E-03					
31	11	E	31	10	E	1090.63736	5	.926251E-03	1.6	0.9643E-03	-4.11	3.706E-04	-.941E-01		1.0

(cont.)

J	K_a	Γ	J'	K_a'	Γ'	$\nu^{a)}$	$\delta_\nu{}^{b)}$	$S_\nu^{\rm exp}(294.45)^{c)}$	$\Delta_S{}^{d)}$	$S_\nu^{\rm calc}(294.45)^{e)}$	$\delta_S{}^{f)}$	$\Gamma_0{}^{g)}$	$\Delta_2{}^{g)}$	$\nu_{\rm VC}{}^{g)}$	$R^{h)}$
	1			2		3	4	5	6	7	8	9	10	11	12
30	11	E	30	10	E	1090.73755	-1			0.1055E-02					
29	11	E	29	10	E	1090.83460	3	.114375E-02	0.9	0.1146E-02	-.24	2.785E-04			1.0
28	11	E	28	10	E	1090.92837	3	.123640E-02	1.1	0.1238E-02	-.15	4.492E-04	-.497E+00	.323E+00	1.2
34	7	A_2	33	6	A_1	1091.01271	9	.533935E-02	0.3	0.2578E-02	3.42	3.290E-04			1.0
34	7	A_1	33	6	A_2	1091.01271	9			0.2578E-02					
27	11	E	27	10	E	1091.01896	9	.131494E-02	1.6	0.1328E-02	-1.02	5.148E-04			1.0
26	11	E	26	10	E	1091.10630	13			0.1415E-02					
54	5	E	53	4	E	1091.16746	10			0.1330E-03					
25	11	E	25	10	E	1091.19032	9	.153097E-02	0.9	0.1496E-02	2.25	4.326E-04			1.0
16	9	E	15	8	E	1091.21412	5	.732447E-02	0.2	0.6989E-02	4.58	4.337E-04	-.131E+00		1.1
25	8	E	24	7	E	1091.23701	8	.535607E-02	0.2	0.5121E-02	4.38	3.899E-04	-.200E+00	-.904E-01	1.3
44	6	E	43	5	E	1091.26116	3	.793540E-03	1.8	0.7443E-03	6.20	3.703E-04			1.0
24	11	E	24	10	E	1091.27112	7	.155167E-02	0.6	0.1570E-02	-1.18	3.623E-04			1.0
23	11	E	23	10	E	1091.34865	1	.160197E-02	0.9	0.1633E-02	-1.95	3.457E-04		-.622E+00	1.2
22	11	E	22	10	E	1091.42300	1	.175308E-02	1.0	0.1684E-02	3.96	5.301E-04	.191E+00	.616E+00	1.1
21	11	E	21	10	E	1091.49413	2	.164531E-02	0.8	0.1718E-02	-4.42	3.426E-04	.555E-01	-.261E+00	1.0
20	11	E	20	10	E	1091.56199	0	.172496E-02	1.0	0.1733E-02	-.49	4.128E-04	-.913E-01		1.0
19	11	E	19	10	E	1091.62673	9			0.1727E-02					
16	13	A_1	17	12	A_2	1091.66818	7			0.3123E-04					
16	13	A_2	17	12	A_1	1091.66818	7			0.3123E-04					
18	11	E	18	10	E	1091.68802	-3	.168822E-02	1.1	0.1694E-02	-.34	3.960E-04			1.0
17	11	E	17	10	E	1091.74624	1	.161703E-02	0.8	0.1632E-02	-.93	3.595E-04	-.224E+00	-.313E+00	1.2
35	7	A_2	34	6	A_1	1091.77707	6	.460349E-02	0.5	0.2282E-02	.85	3.574E-04			1.0
35	7	A_1	34	6	A_2	1091.77707	6			0.2282E-02					
16	11	E	16	10	E	1091.80119	2	.151292E-02	1.0	0.1537E-02	-1.59	3.410E-04	-.214E+00		1.1
15	11	E	15	10	E	1091.85294	5	.151224E-02	0.6	0.1404E-02	7.13	4.620E-04	-.204E+00	-.198E+00	1.2
14	11	E	14	10	E	1091.90139	3	.123230E-02	1.0	0.1230E-02	.16	3.572E-04	-.248E+00	-.349E+00	1.1
13	11	E	13	10	E	1091.94661	0	.977155E-03	1.3	0.1009E-02	-3.29	2.148E-04		-.928E+00	1.2
12	11	E	12	10	E	1091.98790	-72	.148471E-02	0.8	0.7358E-03	7.99	7.169E-04	.558E+00		1.3
45	6	E	44	5	E	1091.98805	63			0.6303E-03					
11	11	E	11	10	E	1092.02778	38	.418656E-03	6.1	0.4024E-03	3.88	2.360E-04			1.0
26	8	E	25	7	E	1092.03434	7	.473739E-02	0.5	0.4716E-02	.44	3.336E-04			1.0
17	9	E	16	8	E	1092.04315	6	.676366E-02	0.4	0.6673E-02	1.34	4.492E-04			1.0
31	15	E	32	14	E	1092.34714	27			0.1562E-04					
36	7	A_2	35	6	A_1	1092.53769	4	.412763E-02	0.4	0.2011E-02	2.56	3.203E-04	-.122E+00		1.1
36	7	A_1	35	6	A_2	1092.53769	4			0.2011E-02					
23	14	E	24	13	E	1092.54933	4			0.3677E-04					
15	13	A_1	16	12	A_2	1092.60289	13			0.2144E-04					
15	13	A_2	16	12	A_1	1092.60289	13			0.2144E-04					
46	6	E	45	5	E	1092.70988	8	.563270E-03	1.2	0.5314E-03	5.66	2.069E-04			1.0
27	8	E	26	7	E	1092.82804	5	.447815E-02	0.4	0.4324E-02	3.44	3.891E-04	-.213E+00		1.2
18	9	E	17	8	E	1092.86869	5	.661453E-02	0.2	0.6347E-02	4.05	4.032E-04	-.281E+00	-.186E+00	1.6
57	5	E	56	4	E	1093.21527	18			0.6991E-04					
37	7	A_2	36	6	A_1	1093.29462	9	.353287E-02	0.7	0.1764E-02	.14	3.000E-04	-.159E+00		1.1
37	7	A_1	36	6	A_2	1093.29462	9			0.1764E-02					
47	6	E	46	5	E	1093.42831	5			0.4460E-03					
10	10	A_2	9	9	A_1	1093.52242	4	.131624E-01	0.2	0.6696E-02	-1.75	2.610E-04	-.116E+00	-.143E+00	1.6
10	10	A_1	9	9	A_2	1093.52242	4			0.6696E-02					
14	13	A_2	15	12	A_1	1093.53445	4			0.1226E-04					
14	13	A_1	15	12	A_2	1093.53445	4			0.1226E-04					
28	8	E	27	7	E	1093.61816	7	.397312E-02	0.3	0.3946E-02	.68	3.159E-04	-.987E-01	-.313E+00	1.5
19	9	E	18	8	E	1093.69074	5	.608762E-02	0.4	0.6013E-02	1.23	3.715E-04	-.110E+00	-.398E+00	1.4
58	5	E	57	4	E	1093.88952	1			0.5592E-04					
37	16	A_2	38	15	A_1	1093.99514	-53			0.4504E-05					
37	16	A_1	38	15	A_2	1093.99514	-53			0.4504E-05					
38	7	A_2	37	6	A_1	1094.04769	7	.318376E-02	0.5	0.1540E-02	3.24	3.319E-04			1.0
38	7	A_1	37	6	A_2	1094.04769	7			0.1540E-02					
48	6	E	47	5	E	1094.14284	5			0.3726E-03					
11	10	A_2	10	9	A_1	1094.37179	2	.127511E-01	0.1	0.6463E-02	-1.37	2.874E-04	-.939E-01	-.295E+00	1.2
11	10	A_1	10	9	A_2	1094.37179	2			0.6463E-02					
29	8	E	28	7	E	1094.40460	7	.372795E-02	0.2	0.3585E-02	3.83	3.344E-04	-.220E+00	-.386E+00	1.7
21	14	E	22	13	E	1094.45642	14			0.3216E-04					
20	9	E	19	8	E	1094.50927	5	.582482E-02	0.3	0.5673E-02	2.61	4.041E-04	-.154E+00	-.156E+00	1.4
59	5	E	58	4	E	1094.55998	17			0.4454E-04					
39	7	A_2	38	6	A_1	1094.79697	6	.274185E-02	0.5	0.1339E-02	2.33	3.089E-04			1.0
39	7	A_1	38	6	A_2	1094.79697	6			0.1339E-02					
49	6	E	48	5	E	1094.85359	23			0.3100E-03					
36	16	A_2	37	15	A_1	1094.98913	1			0.4967E-05					
36	16	A_1	37	15	A_2	1094.98913	1			0.4967E-05					
30	8	E	29	7	E	1095.18739	7	.342918E-02	0.6	0.3242E-02	5.44	3.950E-04			1.0
12	10	A_2	11	9	A_1	1095.21779	3	.123421E-01	0.2	0.6234E-02	-1.01	3.276E-04	-.646E-01	-.208E+00	1.4
12	10	A_1	11	9	A_2	1095.21779	3			0.6234E-02					
28	15	E	29	14	E	1095.27058	10			0.1762E-04					
21	9	E	20	8	E	1095.32431	7	.562364E-02	0.3	0.5330E-02	5.23	4.326E-04			1.0
40	7	A_2	39	6	A_1	1095.54245	6	.231551E-02	0.6	0.1159E-02	-.09	2.274E-04		-.464E+00	1.2
40	7	A_1	39	6	A_2	1095.54245	6			0.1159E-02					
50	6	E	49	5	E	1095.56003	7			0.2567E-03					
49	12	E	49	11	E	1095.70968	-68			0.4784E-04					
48	12	E	48	11	E	1095.86907	4			0.5743E-04					
61	5	E	60	4	E	1095.88802	0			0.2789E-04					
31	8	E	30	7	E	1095.96651	8	.305916E-02	0.6	0.2919E-02	4.57	3.139E-04	-.306E+00	-.348E+00	1.6
47	12	E	47	11	E	1096.02452	7			0.6860E-04					

(cont.)

J	K_a	Γ	J'	K'_a	Γ'	$\nu^{a)}$	$\delta_\nu{}^{b)}$	$S_\nu^{\rm exp}(294.45)^{c)}$	$\Delta_S{}^{d)}$	$S_\nu^{\rm calc}(294.45)^{e)}$	$\delta_S{}^{f)}$	$\Gamma_0{}^{g)}$	$\Delta_2{}^{g)}$	$\nu_{\rm VC}{}^{g)}$	$R^{h)}$
1			2			3	4	5	6	7	8	9	10	11	12
13	10	A_2	12	9	A_1	1096.06038	3	.118777E-01	0.2	0.6003E-02	-1.07	3.210E-04	-.223E+00	-.224E+00	2.3
13	10	A_1	12	9	A_2	1096.06038	3			0.6003E-02					
22	9	E	21	8	E	1096.13578	7	.506913E-02	0.2	0.4986E-02	1.64	3.601E-04	-.226E+00	-.401E+00	2.1
46	12	E	46	11	E	1096.17669	7			0.8156E-04					
51	6	E	50	5	E	1096.26261	3			0.2117E-03					
41	7	A_2	40	6	A_1	1096.28412	8	.207409E-02	0.6	0.9983E-03	3.74	3.774E-04			1.0
41	7	A_1	40	6	A_2	1096.28412	8			0.9983E-03					
45	12	E	45	11	E	1096.32562	9			0.9650E-04					
19	14	E	20	13	E	1096.35148	-13			0.2486E-04					
44	12	E	44	11	E	1096.47122	3			0.1136E-03					
62	5	E	61	4	E	1096.54569	-21			0.2193E-04					
43	12	E	43	11	E	1096.61365	4			0.1331E-03					
32	8	E	31	7	E	1096.74194	9	.274517E-02	0.4	0.2617E-02	4.69	3.681E-04	-.189E+00		1.2
42	12	E	42	11	E	1096.75277	0			0.1552E-03					
41	12	E	41	11	E	1096.88904	36			0.1801E-03					
14	10	A_2	13	9	A_1	1096.89954	3	.110458E-01	0.2	0.5767E-02	-4.42	3.029E-04	-.469E-01	-.419E+00	1.3
14	10	A_1	13	9	A_2	1096.89954	3			0.5767E-02					
23	9	E	22	8	E	1096.94369	6	.474449E-02	0.4	0.4645E-02	2.10	3.673E-04		-.413E+00	1.4
52	6	E	51	5	E	1096.96123	3			0.1738E-03					
40	12	E	40	11	E	1097.02193	58	.186268E-02	1.7	0.2079E-03	-3.09	2.382E-04			1.0
42	7	A_1	41	6	A_2	1097.02193	8			0.8562E-03					
42	7	A_2	41	6	A_1	1097.02193	8			0.8562E-03					
39	12	E	39	11	E	1097.15075	-3			0.2387E-03					
38	12	E	38	11	E	1097.27726	31			0.2727E-03					
18	14	E	19	13	E	1097.29506	20			0.2039E-04					
37	12	E	37	11	E	1097.39993	4			0.3100E-03					
33	8	E	32	7	E	1097.51364	7	.244931E-02	0.3	0.2335E-02	4.68	3.830E-04			1.0
36	12	E	36	11	E	1097.51942	-16	.384493E-03	2.2	0.3504E-03	8.87	5.197E-04			1.0
35	12	E	35	11	E	1097.63597	-6			0.3939E-03					
53	6	E	52	5	E	1097.65582	2			0.1420E-03					
15	10	A_2	14	9	A_1	1097.73528	4	.112435E-01	0.3	0.5526E-02	1.71	3.969E-04			1.0
15	10	A_1	14	9	A_2	1097.73528	4			0.5526E-02					
24	9	E	23	8	E	1097.74808	10	.482599E-02	0.6	0.4308E-02	1.61	4.737E-04			1.0
34	12	E	34	11	E	1097.74808	-115			0.4404E-03					
43	7	A_2	42	6	A_1	1097.75585	5	.150361E-02	1.9	0.7310E-03	2.76	3.447E-04			1.0
43	7	A_1	42	6	A_2	1097.75585	5			0.7310E-03					
33	12	E	33	11	E	1097.85921	1			0.4895E-03					
33	16	A_1	34	15	A_2	1097.95342	26			0.6348E-05					
33	16	A_2	34	15	A_1	1097.95342	26			0.6348E-05					
32	12	E	32	11	E	1097.96595	3			0.5410E-03					
31	12	E	31	11	E	1098.06943	3			0.5942E-03					
30	12	E	30	11	E	1098.16964	-1	.643061E-03	2.3	0.6487E-03	-.88	5.102E-04			1.0
17	14	E	18	13	E	1098.23453	-63			0.1558E-04					
29	12	E	29	11	E	1098.26665	0			0.7036E-03					
34	8	E	33	7	E	1098.28162	6	.217143E-02	0.5	0.2074E-02	4.50	3.503E-04	-.141E+00	.999E-01	1.2
54	6	E	53	5	E	1098.34641	5			0.1156E-03					
28	12	E	28	11	E	1098.36048	6	.750803E-03	2.1	0.7581E-03	-.97	3.786E-04			1.0
27	12	E	27	11	E	1098.45099	4	.807108E-03	1.1	0.8110E-03	-.48	3.406E-04	-.163E+00		1.1
44	7	A_2	43	6	A_1	1098.48591	4	.121707E-02	0.6	0.6214E-03	-2.11	1.934E-04			1.0
44	7	A_1	43	6	A_2	1098.48591	4			0.6214E-03					
26	12	E	26	11	E	1098.53829	5	.861222E-03	2.2	0.8612E-03	.00	3.762E-04			1.0
25	9	E	24	8	E	1098.54878	4	.391468E-02	0.5	0.3978E-02	-1.62	3.769E-04			1.0
16	10	A_2	15	9	A_1	1098.56754	2	.102899E-01	0.4	0.5278E-02	-2.59	3.489E-04	-.610E-01	-.211E+00	1.1
16	10	A_1	15	9	A_2	1098.56754	2			0.5278E-02					
25	12	E	25	11	E	1098.62229	-1	.913628E-03	1.4	0.9073E-03	.69	3.932E-04	-.298E+00		1.1
24	12	E	24	11	E	1098.70319	7	.985143E-03	0.8	0.9478E-03	3.79	5.636E-04			1.0
23	12	E	23	11	E	1098.78051	-19			0.9810E-03					
22	12	E	22	11	E	1098.85505	0	.992155E-03	1.8	0.1005E-02	-1.30	4.469E-04			1.0
21	12	E	21	11	E	1098.92619	2	.102600E-02	1.4	0.1018E-02	.77	4.273E-04	-.207E+00	-.516E+00	1.1
20	12	E	20	11	E	1098.99404	0	.102193E-02	0.9	0.1018E-02	.39	2.022E-04		-.105E+01	1.8
55	6	E	54	5	E	1099.03286	-2			0.9364E-04					
35	8	E	34	7	E	1099.04595	14	.197959E-02	0.7	0.1834E-02	7.37	3.310E-04			1.0
19	12	E	19	11	E	1099.05868	-1	.102274E-02	1.5	0.1002E-02	1.99	4.360E-04			1.0
18	12	E	18	11	E	1099.11967	-43			0.9692E-03					
17	12	E	17	11	E	1099.17831	3			0.9158E-03					
45	7	A_2	44	6	A_1	1099.21203	-2	.110119E-02	1.3	0.5259E-03	4.49	3.204E-04			1.0
45	7	A_1	44	6	A_2	1099.21203	-2			0.5259E-03					
16	12	E	16	11	E	1099.23324	2	.906158E-03	0.8	0.8396E-03	7.35	1.174E-04		-.405E+00	1.1
15	12	E	15	11	E	1099.28493	0	.804913E-03	1.2	0.7375E-03	8.37	5.960E-04	.314E+00	.151E+01	1.2
14	12	E	14	11	E	1099.33342	1	.589840E-03	2.6	0.6066E-03	-2.84	2.615E-04	-.696E+00		1.5
26	9	E	25	8	E	1099.34596	6	.369320E-02	0.8	0.3657E-02	.97	3.901E-04			1.0
13	12	E	13	11	E	1099.37868	3			0.4431E-03					
17	10	A_2	16	9	A_1	1099.39637	4	.100468E-01	0.3	0.5024E-02	-.02	3.919E-04	.798E-01		1.1
17	10	A_1	16	9	A_2	1099.39637	4			0.5024E-02					
12	12	E	12	11	E	1099.42062	-4			0.2427E-03					
56	6	E	55	5	E	1099.71541	7			0.7554E-04					
36	8	E	35	7	E	1099.80641	9	.174875E-02	0.8	0.1614E-02	7.70	4.026E-04	-.182E+00		1.1
46	7	A_2	45	6	A_1	1099.93438	6	.873974E-03	1.4	0.4431E-03	-1.39	2.221E-04	-.364E+00		1.2
46	7	A_1	45	6	A_2	1099.93438	6			0.4431E-03					
23	15	E	24	14	E	1100.08639	-21			0.1632E-04					
15	14	E	16	13	E	1100.10699	19			0.6125E-05					
27	9	E	26	8	E	1100.13952	7	.338649E-02	0.6	0.3348E-02	1.15	3.220E-04	-.462E-01	-.390E+00	1.1

(cont.)

J	K_a	Γ	J'	K_a'	Γ'	$\nu^{a)}$	$\delta_\nu{}^{b)}$	$S_\nu^{\mathrm{exp}}(294.45)^{c)}$	$\Delta_S{}^{d)}$	$S_\nu^{\mathrm{calc}}(294.45)^{e)}$	$\delta_S{}^{f)}$	$\Gamma_0{}^{g)}$	$\Delta_2{}^{g)}$	$\nu_{\mathrm{VC}}{}^{g)}$	$R^{h)}$
1			2			3	4	5	6	7	8	9	10	11	12
18	10	A_2	17	9	A_1	1100.22170	4	.931373E-02	0.2	0.4766E-02	-2.34	3.359E-04		-.444E+00	1.6
18	10	A_1	17	9	A_2	1100.22170	4			0.4766E-02					
57	6	E	56	5	E	1100.39379	8			0.6067E-04					
37	8	E	36	7	E	1100.56319	13	.137179E-02	1.0	0.1415E-02	-3.12	2.636E-04	-.382E+00		1.3
47	7	A_1	46	6	A_2	1100.65272	5			0.3716E-03					
47	7	A_2	46	6	A_1	1100.65272	5			0.3716E-03					
28	9	E	27	8	E	1100.92942	5	.308705E-02	0.4	0.3051E-02	1.18	3.259E-04	.345E-01	-.405E+00	1.2
19	10	A_2	18	9	A_1	1101.04352	2	.881077E-02	0.2	0.4503E-02	-2.22	3.557E-04	-.357E-01	-.387E+00	1.6
19	10	A_1	18	9	A_2	1101.04352	2			0.4503E-02					
58	6	E	57	5	E	1101.06793	-5			0.4852E-04					
38	8	E	37	7	E	1101.31608	6	.134414E-02	1.0	0.1234E-02	8.19	4.394E-04	-.683E+00	.274E+00	1.5
48	7	A_1	47	6	A_2	1101.36715	6			0.3103E-03					
48	7	A_2	47	6	A_1	1101.36715	6			0.3103E-03					
29	9	E	28	8	E	1101.71573	8	.279457E-02	0.4	0.2768E-02	.96	3.094E-04	-.183E+00	-.326E+00	1.4
11	11	E	10	10	E	1101.76457	4	.473679E-02	0.2	0.4723E-02	.29	2.572E-04	-.168E+00	-.269E+00	2.0
20	10	A_2	19	9	A_1	1101.86186	4	.838586E-02	0.2	0.4239E-02	-1.09	3.583E-04	-.797E-01	-.251E+00	1.4
20	10	A_1	19	9	A_2	1101.86186	4			0.4239E-02					
21	15	E	22	14	E	1101.99260	-32			0.1357E-04					
39	8	E	38	7	E	1102.06526	9	.110688E-02	0.9	0.1072E-02	3.16	2.248E-04	-.367E+00		1.5
49	7	A_1	48	6	A_2	1102.07761	6			0.2580E-03					
49	7	A_2	48	6	A_1	1102.07761	6			0.2580E-03					
60	6	E	59	5	E	1102.40419	2			0.3063E-04					
30	9	E	29	8	E	1102.49829	3	.255903E-02	0.5	0.2500E-02	2.31	3.264E-04	-.359E+00	-.397E+00	1.5
12	11	E	11	10	E	1102.61027	-1	.442993E-02	0.8	0.4536E-02	-2.39	2.939E-04	-.179E+00	-.336E+00	1.2
50	7	A_2	49	6	A_1	1102.78408	5			0.2136E-03					
50	7	A_1	49	6	A_2	1102.78408	5			0.2136E-03					
40	8	E	39	7	E	1102.81046	-6			0.9268E-03					
28	16	A_1	29	15	A_2	1102.83832	30			0.7864E-05					
28	16	A_2	29	15	A_1	1102.83832	30			0.7864E-05					
61	6	E	60	5	E	1103.06610	4			0.2418E-04					
31	9	E	30	8	E	1103.27724	4	.237948E-02	0.8	0.2248E-02	5.53	3.706E-04	-.365E+00		1.3
13	11	E	12	10	E	1103.45262	0	.443969E-02	0.3	0.4351E-02	2.00	3.525E-04	-.221E+00		1.5
41	8	E	40	7	E	1103.55210	6	.749429E-03	1.3	0.7979E-03	-6.46	9.285E-05	-.166E+00		1.1
46	13	A_1	46	12	A_2	1103.64257	-46			0.4848E-04					
46	13	A_2	46	12	A_1	1103.64257	-46			0.4848E-04					
62	6	E	61	5	E	1103.72365	-13			0.1900E-04					
19	15	E	20	14	E	1103.88720	-37			0.9635E-05					
44	13	A_2	44	12	A_1	1103.93766	5			0.6747E-04					
44	13	A_1	44	12	A_2	1103.93766	5			0.6747E-04					
32	9	E	31	8	E	1104.05250	5	.211825E-02	0.7	0.2012E-02	4.99	3.525E-04	-.429E+00		1.5
43	13	A_2	43	12	A_1	1104.08002	-1			0.7900E-04					
43	13	A_1	43	12	A_2	1104.08002	-1			0.7900E-04					
52	7	A_2	51	6	A_1	1104.18517	13			0.1444E-03					
52	7	A_1	51	6	A_2	1104.18517	13			0.1444E-03					
42	13	A_2	42	12	A_1	1104.21927	7			0.9205E-04					
42	13	A_1	42	12	A_2	1104.21927	7			0.9205E-04					
14	11	E	13	10	E	1104.29153	-1	.432008E-02	0.7	0.4166E-02	3.57	2.770E-04			1.0
41	13	A_2	41	12	A_1	1104.35517	5			0.1067E-03					
41	13	A_1	41	12	A_2	1104.35517	5			0.1067E-03					
40	13	A_1	40	12	A_2	1104.48785	6			0.1231E-03					
40	13	A_2	40	12	A_1	1104.48785	6			0.1231E-03					
39	13	A_2	39	12	A_1	1104.61724	3			0.1412E-03					
39	13	A_1	39	12	A_2	1104.61724	3			0.1412E-03					
38	13	A_2	38	12	A_1	1104.74346	6			0.1612E-03					
38	13	A_1	38	12	A_2	1104.74346	6			0.1612E-03					
26	16	A_2	27	15	A_1	1104.77167	-65			0.7827E-05					
26	16	A_1	27	15	A_2	1104.77167	-65			0.7827E-05					
33	9	E	32	8	E	1104.82407	7	.182420E-02	0.7	0.1794E-02	1.68	3.286E-04	-.128E+00	-.255E+00	1.2
37	13	A_2	37	12	A_1	1104.86633	0			0.1830E-03					
37	13	A_1	37	12	A_2	1104.86633	0			0.1830E-03					
53	7	A_2	52	6	A_1	1104.87988	36			0.1180E-03					
53	7	A_1	52	6	A_2	1104.87988	36			0.1180E-03					
36	13	A_2	36	12	A_1	1104.98597	-5			0.2066E-03					
36	13	A_1	36	12	A_2	1104.98597	-5			0.2066E-03					
43	8	E	42	7	E	1105.02360	8			0.5834E-03					
15	11	E	14	10	E	1105.12703	1	.400751E-02	0.3	0.3979E-02	.72	3.453E-04	-.177E+00	-.337E+00	1.6
34	13	A_2	34	12	A_1	1105.21546	-22			0.2589E-03					
34	13	A_1	34	12	A_2	1105.21546	-22			0.2589E-03					
33	13	A_2	33	12	A_1	1105.32567	2			0.2873E-03					
33	13	A_1	33	12	A_2	1105.32567	2			0.2873E-03					
32	13	A_2	32	12	A_1	1105.43239	1			0.3170E-03					
32	13	A_1	32	12	A_2	1105.43239	1			0.3170E-03					
31	13	A_2	31	12	A_1	1105.53589	3			0.3475E-03					
31	13	A_1	31	12	A_2	1105.53589	3			0.3475E-03					
54	7	A_1	53	6	A_2	1105.56971	-26			0.9598E-04					
54	7	A_2	53	6	A_1	1105.56971	-26			0.9598E-04					
34	9	E	33	8	E	1105.59188	6	.168779E-02	1.3	0.1591E-02	5.71	3.979E-04	-.306E+00		1.2
30	13	A_2	30	12	A_1	1105.63613	2	.739643E-03	0.7	0.3785E-03	-2.35	3.585E-04	-.688E+00		2.3
30	13	A_1	30	12	A_2	1105.63613	2			0.3785E-03					
29	13	A_2	29	12	A_1	1105.73312	0	.852635E-03	1.7	0.4095E-03	3.94	6.411E-04			1.0
29	13	A_1	29	12	A_2	1105.73312	0			0.4095E-03					
44	8	E	43	7	E	1105.75352	6			0.4956E-03					

(cont.)

J	K_a	Γ	J'	K_a'	Γ'	$\nu^{a)}$	$\delta_\nu{}^{b)}$	$S_\nu^{\rm exp}(294.45)^{c)}$	$\Delta_S{}^{d)}$	$S_\nu^{\rm calc}(294.45)^{e)}$	$\delta_S{}^{f)}$	$\Gamma_0{}^{g)}$	$\Delta_2{}^{g)}$	$\nu_{\rm VC}{}^{g)}$	$R^{h)}$
	1			2		3	4	5	6	7	8	9	10	11	12
28	13	A_2	28	12	A_1	1105.82691	2	.873582E-03	1.4	0.4399E-03	-.72	4.349E-04	-.414E+00		1.2
28	13	A_1	28	12	A_2	1105.82691	2			0.4399E-03					
27	13	A_2	27	12	A_1	1105.91741	-1	.929038E-03	1.6	0.4691E-03	-.99	2.929E-04	-.364E+00		1.2
27	13	A_1	27	12	A_2	1105.91741	-1			0.4691E-03					
16	11	E	15	10	E	1105.95908	2	.386033E-02	0.3	0.3789E-02	1.85	4.117E-04			1.0
26	13	A_2	26	12	A_1	1106.00472	0	.948366E-03	1.3	0.4963E-03	-4.66	2.888E-04			1.0
26	13	A_1	26	12	A_2	1106.00472	0			0.4963E-03					
25	13	A_2	25	12	A_1	1106.08876	-2	.100092E-02	0.7	0.5205E-03	-4.01	2.257E-04		-.794E+00	1.7
25	13	A_1	25	12	A_2	1106.08876	-2			0.5205E-03					
24	13	A_2	24	12	A_1	1106.16935	-25	.107808E-02	1.8	0.5409E-03	-.35	4.031E-04			1.0
24	13	A_1	24	12	A_2	1106.16935	-25			0.5409E-03					
23	13	A_2	23	12	A_1	1106.24719	1	.111124E-02	1.0	0.5564E-03	-.14	1.144E-04		-.847E+00	1.5
23	13	A_1	23	12	A_2	1106.24719	1			0.5564E-03					
55	7	A_2	54	6	A_1	1106.25632	-4			0.7774E-04					
55	7	A_1	54	6	A_2	1106.25632	-4			0.7774E-04					
22	13	A_1	22	12	A_2	1106.32155	1			0.5658E-03					
22	13	A_2	22	12	A_1	1106.32155	1			0.5658E-03					
35	9	E	34	8	E	1106.35597	6	.144844E-02	1.1	0.1406E-02	2.94	3.361E-04			1.0
21	13	A_2	21	12	A_1	1106.39278	13			0.5679E-03					
21	13	A_1	21	12	A_2	1106.39278	13			0.5679E-03					
20	13	A_2	20	12	A_1	1106.46053	0	.111709E-02	1.5	0.5613E-03	-.50	4.744E-04			1.0
20	13	A_1	20	12	A_2	1106.46053	0			0.5613E-03					
45	8	E	44	7	E	1106.47946	-5	.423710E-03	1.3	0.4191E-03	1.08	1.306E-04		-.556E+00	1.3
19	13	A_2	19	12	A_1	1106.52513	-5	.108945E-02	0.8	0.5447E-03	.01	2.843E-04	.285E+00	-.229E+00	1.3
19	13	A_1	19	12	A_2	1106.52513	-5			0.5447E-03					
18	13	A_2	18	12	A_1	1106.58658	-1	.103818E-02	1.6	0.5164E-03	.51	3.238E-04	.614E+00	-.295E+00	1.4
18	13	A_1	18	12	A_2	1106.58658	-1			0.5164E-03					
17	13	A_2	17	12	A_1	1106.64474	-3	.961171E-03	1.0	0.4750E-03	1.17	6.000E-04		.236E+01	1.2
17	13	A_1	17	12	A_2	1106.64474	-3			0.4750E-03					
15	13	A_2	15	12	A_1	1106.75140	-2	.749077E-03	1.9	0.3451E-03	7.86	2.927E-04			1.0
15	13	A_1	15	12	A_2	1106.75140	-2			0.3451E-03					
17	11	E	16	10	E	1106.78766	3	.354621E-02	0.3	0.3597E-02	-1.43	3.549E-04	-.127E+00	-.388E+00	1.6
14	13	A_1	14	12	A_2	1106.79986	-4			0.2526E-03					
14	13	A_2	14	12	A_1	1106.79986	-4			0.2526E-03					
13	13	A_1	13	12	A_2	1106.84513	-2			0.1386E-03					
13	13	A_2	13	12	A_1	1106.84513	-2			0.1386E-03					
56	7	A_1	55	6	A_2	1106.93885	16			0.6268E-04					
56	7	A_2	55	6	A_1	1106.93885	16			0.6268E-04					
36	9	E	35	8	E	1107.11630	5	.115542E-02	1.7	0.1236E-02	-7.01	2.593E-04	.238E+00		1.1
46	8	E	45	7	E	1107.20133	-32			0.3529E-03					
18	11	E	17	10	E	1107.61274	2	.346136E-02	0.4	0.3403E-02	1.69	4.058E-04	-.416E+00	-.107E+00	1.9
37	9	E	36	8	E	1107.87289	7	.115302E-02	1.3	0.1083E-02	6.11	3.463E-04	-.203E+00		1.1
47	8	E	46	7	E	1107.91997	11			0.2959E-03					
58	7	A_1	57	6	A_2	1108.29092	-17			0.4023E-04					
58	7	A_2	57	6	A_1	1108.29092	-17			0.4023E-04					
19	11	E	18	10	E	1108.43430	-1	.315213E-02	0.3	0.3208E-02	-1.77	3.607E-04	-.317E-01	-.463E+00	1.5
38	9	E	37	8	E	1108.62571	10	.948532E-03	0.7	0.9436E-03	.52	5.909E-04	-.143E+00	.202E+01	1.7
48	8	E	47	7	E	1108.63418	4			0.2469E-03					
59	7	A_1	58	6	A_2	1108.96107	-5			0.3202E-04					
59	7	A_2	58	6	A_1	1108.96107	-5			0.3202E-04					
20	11	E	19	10	E	1109.25240	0	.291086E-02	0.5	0.3013E-02	-3.50	3.199E-04		-.532E+00	1.4
49	8	E	48	7	E	1109.34451	5			0.2052E-03					
39	9	E	38	8	E	1109.37468	8	.849004E-03	1.1	0.8189E-03	3.54	3.163E-04	-.272E+00		1.2
21	16	A_2	22	15	A_1	1109.55816	17			0.5241E-05					
21	16	A_1	22	15	A_2	1109.55816	17			0.5241E-05					
60	7	A_1	59	6	A_2	1109.62686	-16			0.2538E-04					
60	7	A_2	59	6	A_1	1109.62686	-16			0.2538E-04					
12	12	E	11	11	E	1110.03899	-2			0.3132E-02					
50	8	E	49	7	E	1110.05090	8			0.1698E-03					
21	11	E	20	10	E	1110.06698	2	.276668E-02	0.6	0.2819E-02	-1.88	3.316E-04	-.170E+00	-.395E+00	1.2
40	9	E	39	8	E	1110.11987	9	.716271E-03	1.0	0.7076E-03	1.22	3.460E-04	-.242E+00	.626E+00	1.2
51	8	E	50	7	E	1110.75302	-17			0.1398E-03					
41	9	E	40	8	E	1110.86137	24			0.6086E-03					
22	11	E	21	10	E	1110.87805	6	.269863E-02	2.3	0.2627E-02	2.67	1.429E-04			1.0
13	12	E	12	11	E	1110.88108	0	.321711E-02	2.3	0.2994E-02	6.95	1.429E-04			1.0
52	8	E	51	7	E	1111.45160	5			0.1147E-03					
19	16	A_2	20	15	A_1	1111.45160	-32			0.3308E-05					
19	16	A_1	20	15	A_2	1111.45160	-32			0.3308E-05					
63	7	A_2	62	6	A_1	1111.59875	-99			0.1231E-04					
63	7	A_1	62	6	A_2	1111.59875	-99			0.1231E-04					
42	9	E	41	8	E	1111.59875	11			0.5212E-03					
23	11	E	22	10	E	1111.68549	4	.241027E-02	0.7	0.2438E-02	-1.13	3.457E-04	-.236E+00	-.416E+00	1.3
14	12	E	13	11	E	1111.71972	-1	.288382E-02	0.4	0.2857E-02	.94	4.270E-04	-.407E+00		2.1
41	14	E	41	13	E	1111.85375	5			0.6004E-04					
40	14	E	40	13	E	1111.98648	10			0.6918E-04					
39	14	E	39	13	E	1112.11584	2			0.7929E-04					
53	8	E	52	7	E	1112.14590	0			0.9366E-04					
38	14	E	38	13	E	1112.24202	0			0.9040E-04					
43	9	E	42	8	E	1112.33238	9			0.4444E-03					
37	14	E	37	13	E	1112.36474	-23			0.1025E-03					
37	14	E	37	13	E	1112.36500	3			0.1025E-03					
26	17	E	27	16	E	1112.36500	-38			0.3184E-05					

(cont.)

J	K_a	Γ	J'	K'_a	Γ'	$\nu^{a)}$	$\delta_\nu{}^{b)}$	$S_\nu^{\rm exp}(294.45)^{c)}$	$\Delta_S{}^{d)}$	$S_\nu^{\rm calc}(294.45)^{e)}$	$\delta_S{}^{f)}$	$\Gamma_0{}^{g)}$	$\Delta_2{}^{g)}$	$\nu_{\rm VC}{}^{g)}$	$R^{h)}$
1			2			3	4	5	6	7	8	9	10	11	12
26	17	E	27	16	E	1112.36474	-64			0.3184E-05					
24	11	E	23	10	E	1112.48933	-2	.214563E-02	0.9	0.2253E-02	-5.00	3.399E-04	.211E+00	-.293E+00	1.2
15	12	E	14	11	E	1112.55489	-5	.269692E-02	0.8	0.2720E-02	-.86	3.253E-04	.185E+00	-.507E+00	1.3
35	14	E	35	13	E	1112.60098	-15			0.1296E-03					
34	14	E	34	13	E	1112.71437	2			0.1444E-03					
33	14	E	33	13	E	1112.82436	3			0.1600E-03					
54	8	E	53	7	E	1112.83637	16			0.7615E-04					
32	14	E	32	13	E	1112.93114	7			0.1761E-03					
31	14	E	31	13	E	1113.03470	14			0.1926E-03					
44	9	E	43	8	E	1113.06218	12	.365643E-03	2.8	0.3773E-03	-3.19	4.190E-04			1.0
30	14	E	30	13	E	1113.13459	-23			0.2093E-03					
29	14	E	29	13	E	1113.23186	2			0.2257E-03					
25	11	E	24	10	E	1113.28968	1	.212829E-02	0.6	0.2074E-02	2.56	4.102E-04	.168E+00	-.258E+00	1.2
28	14	E	28	13	E	1113.32561	-1			0.2417E-03					
16	12	E	15	11	E	1113.38670	0	.262442E-02	0.5	0.2583E-02	1.56	4.350E-04			1.0
27	14	E	27	13	E	1113.41614	-2			0.2567E-03					
26	14	E	26	13	E	1113.50342	-5			0.2704E-03					
55	8	E	54	7	E	1113.52253	6			0.6165E-04					
25	14	E	25	13	E	1113.58754	1			0.2821E-03					
24	14	E	24	13	E	1113.66832	-4			0.2913E-03					
23	14	E	23	13	E	1113.74593	-3			0.2974E-03					
45	9	E	44	8	E	1113.78803	8			0.3189E-03					
22	14	E	22	13	E	1113.82026	-5			0.2996E-03					
21	14	E	21	13	E	1113.89143	-1			0.2972E-03					
20	14	E	20	13	E	1113.95935	2			0.2895E-03					
19	14	E	19	13	E	1114.02399	1			0.2754E-03					
26	11	E	25	10	E	1114.08636	-3	.205836E-02	0.7	0.1901E-02	7.66	5.281E-04	-.751E+00		1.6
17	14	E	17	13	E	1114.14358	0			0.2246E-03					
16	14	E	16	13	E	1114.19852	-1			0.1857E-03					
17	12	E	16	11	E	1114.21498	-2	.247525E-02	0.7	0.2446E-02	1.17	3.741E-04	-.269E+00	-.386E+00	1.4
15	14	E	15	13	E	1114.25020	-5			0.1363E-03					
14	14	E	14	13	E	1114.29867	-6			0.7493E-04					
36	10	A_2	35	9	A_1	1114.46563	6	.173931E-02	0.4	0.9029E-03	-3.83	2.659E-04	-.200E+00	-.321E+00	1.4
36	10	A_1	35	9	A_2	1114.46563	6			0.9029E-03					
46	9	E	45	8	E	1114.50997	5			0.2683E-03					
27	11	E	26	10	E	1114.87950	1	.168902E-02	0.6	0.1735E-02	-2.71	3.269E-04	-.350E+00	-.382E+00	1.6
18	12	E	17	11	E	1115.03981	0	.228601E-02	0.4	0.2309E-02	-1.00	3.520E-04	-.430E+00	-.269E+00	1.5
37	10	A_2	36	9	A_1	1115.22200	6	.167843E-02	0.6	0.7899E-03	5.88	3.730E-04			1.0
37	10	A_1	36	9	A_2	1115.22200	6			0.7899E-03					
47	9	E	46	8	E	1115.22795	-3			0.2248E-03					
58	8	E	57	7	E	1115.55675	-3			0.3187E-04					
28	11	E	27	10	E	1115.66900	3	.160673E-02	0.4	0.1577E-02	1.87	4.073E-04	-.169E+00	.276E+00	1.1
19	12	E	18	11	E	1115.86114	0	.209372E-02	0.5	0.2172E-02	-3.73	3.813E-04	-.312E+00		1.6
48	9	E	47	8	E	1115.94216	7			0.1875E-03					
38	10	A_2	37	9	A_1	1115.97458	5	.133158E-02	0.5	0.6880E-03	-3.33	2.871E-04	-.236E+00		1.2
38	10	A_1	37	9	A_2	1115.97458	5			0.6880E-03					
29	11	E	28	10	E	1116.45486	5	.145769E-02	1.0	0.1427E-02	2.12	4.161E-04	-.190E+00	.230E+00	1.1
49	9	E	48	8	E	1116.65233	8			0.1558E-03					
20	12	E	19	11	E	1116.67889	-6			0.2036E-02					
39	10	A_2	38	9	A_1	1116.72340	8	.117701E-02	1.0	0.5966E-03	-1.37	3.138E-04	-.167E+00		1.1
39	10	A_1	38	9	A_2	1116.72340	8			0.5966E-03					
30	11	E	29	10	E	1117.23704	6	.128392E-02	1.1	0.1286E-02	-.13	2.222E-04		-.402E+00	1.1
50	9	E	49	8	E	1117.35853	8			0.1288E-03					
40	10	A_2	39	9	A_1	1117.46837	7	.999718E-03	0.9	0.5151E-03	-3.04	2.959E-04	-.181E+00	-.249E+00	1.1
40	10	A_1	39	9	A_2	1117.46837	7			0.5151E-03					
21	12	E	20	11	E	1117.49327	3	.190824E-02	0.3	0.1901E-02	.39	4.061E-04	-.959E-01	-.256E+00	1.1
31	11	E	30	10	E	1118.01552	4	.115415E-02	0.8	0.1153E-02	.07	3.970E-04			1.0
51	9	E	50	8	E	1118.06070	5			0.1061E-03					
41	10	A_1	40	9	A_2	1118.20952	6			0.4427E-03					
41	10	A_1	40	9	A_2	1118.20955	9			0.4427E-03					
41	10	A_2	40	9	A_1	1118.20955	9			0.4427E-03					
41	10	A_2	40	9	A_1	1118.20952	6			0.4427E-03					
22	12	E	21	11	E	1118.30401	2	.173205E-02	0.6	0.1768E-02	-2.08	3.751E-04	-.189E+00	-.236E+00	1.2
13	13	A_2	12	12	A_1	1118.34366	0	.380666E-02	0.3	0.1953E-02	-2.63	2.352E-04	-.131E+00	-.347E+00	1.7
13	13	A_1	12	12	A_2	1118.34366	0			0.1953E-02					
52	9	E	51	8	E	1118.75853	-32			0.8694E-04					
32	11	E	31	10	E	1118.79031	2	.101543E-02	1.5	0.1030E-02	-1.46	4.089E-04	-.905E+00		1.5
42	10	A_2	41	9	A_1	1118.94682	5			0.3789E-03					
42	10	A_1	41	9	A_2	1118.94682	5			0.3789E-03					
23	12	E	22	11	E	1119.11121	2	.157969E-02	0.6	0.1638E-02	-3.70	3.254E-04	-.132E+00	-.275E+00	1.1
14	13	A_2	13	12	A_1	1119.18198	-3	.361305E-02	0.3	0.1859E-02	-2.88	2.264E-04		-.543E+00	1.5
14	13	A_1	13	12	A_2	1119.18198	-3			0.1859E-02					
53	9	E	52	8	E	1119.45298	-6			0.7096E-04					
40	15	E	40	14	E	1119.51474	-21			0.3689E-04					
33	11	E	32	10	E	1119.56145	5	.890986E-03	1.3	0.9163E-03	-2.84	4.527E-04	-.362E+00		1.2
39	15	E	39	14	E	1119.64395	-47			0.4223E-04					
38	15	E	38	14	E	1119.77014	-49			0.4808E-04					
37	15	E	37	14	E	1119.89361	1			0.5444E-04					
24	12	E	23	11	E	1119.91484	2	.150522E-02	1.1	0.1512E-02	-.43	3.606E-04	-.132E+00	-.285E+00	1.1
15	13	A_2	14	12	A_1	1120.01694	2	.355440E-02	0.4	0.1765E-02	.69	3.871E-04	-.207E+00	-.181E+00	1.6
15	13	A_1	14	12	A_2	1120.01694	2			0.1765E-02					
35	15	E	35	14	E	1120.12980	-1			0.6859E-04					

(cont.)

J	K_a	Γ	J'	K'_a	Γ'	$\nu^{a)}$	$\delta_\nu{}^{b)}$	$S_\nu^{\rm exp}(294.45)^{c)}$	$\Delta_S{}^{d)}$	$S_\nu^{\rm calc}(294.45)^{e)}$	$\delta_S{}^{f)}$	$\Gamma_0{}^{g)}$	$\Delta_2{}^{g)}$	$\nu_{\rm VC}{}^{g)}$	$R^{h)}$
	1			2		3	4	5	6	7	8	9	10	11	12
54	9	E	53	8	E	1120.14324	5			0.5767E-04					
34	15	E	34	14	E	1120.24300	-5			0.7631E-04					
34	11	E	33	10	E	1120.32886	8	.808190E-03	1.0	0.8115E-03	-.41	3.558E-04	-.139E+00		1.0
33	15	E	33	14	E	1120.35309	4			0.8436E-04					
44	10	A_2	43	9	A_1	1120.40986	6			0.2739E-03					
44	10	A_1	43	9	A_2	1120.40986	6			0.2739E-03					
32	15	E	32	14	E	1120.45987	6			0.9265E-04					
31	15	E	31	14	E	1120.56335	3			0.1011E-03					
30	15	E	30	14	E	1120.66365	5			0.1095E-03					
25	12	E	24	11	E	1120.71487	0	.134638E-02	1.0	0.1389E-02	-3.19	3.984E-04	-.422E+00		1.2
29	15	E	29	14	E	1120.76066	3			0.1177E-03					
55	9	E	54	8	E	1120.82936	7			0.4667E-04					
16	13	A_2	15	12	A_1	1120.84835	-3	.330286E-02	0.7	0.1672E-02	-1.24	3.579E-04	-.412E+00	-.347E+00	1.5
16	13	A_1	15	12	A_2	1120.84835	-3			0.1672E-02					
28	15	E	28	14	E	1120.85467	24			0.1255E-03					
27	15	E	27	14	E	1120.94520	21			0.1327E-03					
26	15	E	26	14	E	1121.03224	-7			0.1390E-03					
35	11	E	34	10	E	1121.09249	5			0.7155E-03					
25	15	E	25	14	E	1121.11640	1			0.1441E-03					
45	10	A_1	44	9	A_2	1121.13555	7			0.2314E-03					
45	10	A_2	44	9	A_1	1121.13555	7			0.2314E-03					
24	15	E	24	14	E	1121.19722	-1			0.1477E-03					
23	15	E	23	14	E	1121.27473	-11			0.1494E-03					
22	15	E	22	14	E	1121.34926	5			0.1488E-03					
21	15	E	21	14	E	1121.42043	8			0.1454E-03					
20	15	E	20	14	E	1121.48815	-10			0.1388E-03					
26	12	E	25	11	E	1121.51134	2	.122064E-02	0.8	0.1272E-02	-4.18	2.757E-04	-.562E+00	-.404E+00	1.3
19	15	E	19	14	E	1121.55291	0			0.1285E-03					
18	15	E	18	14	E	1121.61429	-5			0.1139E-03					
17	13	A_2	16	12	A_1	1121.67637	0	.331960E-02	0.4	0.1579E-02	4.84	5.169E-04	-.367E+00	.861E+00	1.2
17	13	A_1	16	12	A_2	1121.67637	0			0.1579E-02					
16	15	E	16	14	E	1121.72750	0			0.6952E-04					
15	15	E	15	14	E	1121.77919	-3			0.3831E-04					
36	11	E	35	10	E	1121.85246	12			0.6281E-03					
46	10	A_1	45	9	A_2	1121.85725	-1			0.1946E-03					
46	10	A_2	45	9	A_1	1121.85725	-1			0.1946E-03					
27	12	E	26	11	E	1122.30417	2	.110024E-02	1.2	0.1159E-02	-5.35	2.768E-04	-.244E+00		1.1
18	13	A_2	17	12	A_1	1122.50086	-3	.287025E-02	0.8	0.1488E-02	-3.65	3.841E-04			1.0
18	13	A_1	17	12	A_2	1122.50086	-3			0.1488E-02					
47	10	A_2	46	9	A_1	1122.57531	19			0.1630E-03					
47	10	A_1	46	9	A_2	1122.57531	19			0.1630E-03					
37	11	E	36	10	E	1122.60854	6			0.5490E-03					
28	12	E	27	11	E	1123.09338	2	.101895E-02	1.2	0.1052E-02	-3.25	4.094E-04	-.742E+00	.275E+00	1.7
48	10	A_2	47	9	A_1	1123.28913	8			0.1359E-03					
48	10	A_1	47	9	A_2	1123.28913	8			0.1359E-03					
19	13	A_2	18	12	A_1	1123.32191	0	.272394E-02	0.4	0.1396E-02	-2.53	3.551E-04	-.275E+00	-.353E+00	1.9
19	13	A_1	18	12	A_2	1123.32191	0			0.1396E-02					
38	11	E	37	10	E	1123.36089	5			0.4778E-03					
29	12	E	28	11	E	1123.87894	2	.961834E-03	1.0	0.9510E-03	1.13	4.487E-04			1.0
49	10	A_1	48	9	A_2	1123.99908	7			0.1128E-03					
49	10	A_2	48	9	A_1	1123.99908	7			0.1128E-03					
39	11	E	38	10	E	1124.10947	7			0.4140E-03					
20	13	A_2	19	12	A_1	1124.13945	3	.253255E-02	0.5	0.1306E-02	-3.16	3.754E-04	-.280E+00	-.264E+00	1.2
20	13	A_1	19	12	A_2	1124.13945	3			0.1306E-02					
30	12	E	29	11	E	1124.66085	2	.846626E-03	1.2	0.8559E-03	-1.10	2.886E-04			1.0
50	10	A_2	49	9	A_1	1124.70507	6			0.9322E-04					
50	10	A_1	49	9	A_2	1124.70507	6			0.9322E-04					
40	11	E	39	10	E	1124.85422	7			0.3572E-03					
21	13	A_2	20	12	A_1	1124.95341	0	.239813E-02	0.6	0.1218E-02	-1.54	3.908E-04	-.169E+00		1.1
21	13	A_1	20	12	A_2	1124.95341	0			0.1218E-02					
51	10	A_2	50	9	A_1	1125.40710	8			0.7671E-04					
51	10	A_1	50	9	A_2	1125.40710	8			0.7671E-04					
31	12	E	30	11	E	1125.43909	3	.772699E-03	1.0	0.7671E-03	.73	4.198E-04			1.0
41	11	E	40	10	E	1125.59515	8			0.3069E-03					
22	13	A_2	21	12	A_1	1125.76386	0	.220209E-02	0.7	0.1131E-02	-2.69	3.765E-04			1.0
22	13	A_1	21	12	A_2	1125.76386	0			0.1131E-02					
52	10	A_2	51	9	A_1	1126.10457	-47			0.6286E-04					
52	10	A_1	51	9	A_2	1126.10457	-47			0.6286E-04					
32	12	E	31	11	E	1126.21363	3	.702667E-03	1.3	0.6845E-03	2.58	6.336E-04	-.456E+00	.493E+00	1.2
42	11	E	41	10	E	1126.33218	3			0.2624E-03					
23	13	A_2	22	12	A_1	1126.57071	-5	.209787E-02	0.5	0.1046E-02	.29	4.181E-04	-.361E+00		1.7
23	13	A_1	22	12	A_2	1126.57071	-5			0.1046E-02					
14	14	E	13	13	E	1126.67625	-2	.115389E-02	1.1	0.1147E-02	.64	3.123E-04			1.0
53	10	A_2	52	9	A_1	1126.79907	4			0.5129E-04					
53	10	A_1	52	9	A_2	1126.79907	4			0.5129E-04					
33	12	E	32	11	E	1126.98449	5	.625192E-03	1.7	0.6083E-03	2.71	4.017E-04	.948E-01	.921E+00	1.1
43	11	E	42	10	E	1127.06545	8			0.2235E-03					
24	13	A_2	23	12	A_1	1127.37407	-1	.182795E-02	0.5	0.9638E-03	-5.45	3.369E-04	-.257E+00		1.5
24	13	A_1	23	12	A_2	1127.37407	-1			0.9638E-03					
54	10	A_1	53	9	A_2	1127.48871	-28			0.4166E-04					
54	10	A_2	53	9	A_1	1127.48871	-28			0.4166E-04					
15	14	E	14	13	E	1127.51087	2	.108481E-02	1.3	0.1086E-02	-.11	3.067E-04	-.488E+00		1.3

(cont.)

J	K_a	Γ	J'	K'_a	Γ'	$\nu^{a)}$	$\delta_\nu{}^{b)}$	$S_\nu^{\rm exp}(294.45)^{c)}$	$\Delta_S{}^{d)}$	$S_\nu^{\rm calc}(294.45)^{e)}$	$\delta_S{}^{f)}$	$\Gamma_0{}^{g)}$	$\Delta_2{}^{g)}$	$\nu_{\rm VC}{}^{g)}$	$R^{h)}$
1			2			3	4	5	6	7	8	9	10	11	12
35	16	A_1	35	15	A_2	1127.68635	9			0.3439E-04					
35	16	A_2	35	15	A_1	1127.68635	9			0.3439E-04					
34	12	E	33	11	E	1127.75159	3			0.5382E-03					
44	11	E	43	10	E	1127.79479	7			0.1895E-03					
34	16	A_2	34	15	A_1	1127.79934	-19			0.3817E-04					
34	16	A_1	34	15	A_2	1127.79934	-19			0.3817E-04					
33	16	A_1	33	15	A_2	1127.90953	-3			0.4210E-04					
33	16	A_2	33	15	A_1	1127.90953	-3			0.4210E-04					
32	16	A_2	32	15	A_1	1128.01627	-8			0.4611E-04					
32	16	A_1	32	15	A_2	1128.01627	-8			0.4611E-04					
31	16	A_2	31	15	A_1	1128.11985	-4			0.5015E-04					
31	16	A_1	31	15	A_2	1128.11985	-4			0.5015E-04					
25	13	A_2	24	12	A_1	1128.17388	5	.178505E-02	0.3	0.8845E-03	.90	3.843E-04	-.944E-01	-.335E+00	1.2
25	13	A_1	24	12	A_2	1128.17388	5			0.8845E-03					
55	10	A_1	54	9	A_2	1128.17388	-101			0.3370E-04					
55	10	A_2	54	9	A_1	1128.17388	-101			0.3370E-04					
30	16	A_1	30	15	A_2	1128.22025	6			0.5413E-04					
30	16	A_2	30	15	A_1	1128.22025	6			0.5413E-04					
29	16	A_1	29	15	A_2	1128.31728	3			0.5796E-04					
29	16	A_2	29	15	A_1	1128.31728	3			0.5796E-04					
16	14	E	15	13	E	1128.34200	2	.102605E-02	1.3	0.1026E-02	-.03	3.363E-04			1.0
28	16	A_2	28	15	A_1	1128.41111	4			0.6153E-04					
28	16	A_1	28	15	A_2	1128.41111	4			0.6153E-04					
27	16	A_1	27	15	A_2	1128.50194	29			0.6471E-04					
27	16	A_2	27	15	A_1	1128.50194	29			0.6471E-04					
35	12	E	34	11	E	1128.51504	9			0.4741E-03					
45	11	E	44	10	E	1128.52024	6			0.1600E-03					
26	16	A_2	26	15	A_1	1128.58900	0			0.6735E-04					
26	16	A_1	26	15	A_2	1128.58900	0			0.6735E-04					
25	16	A_2	25	15	A_1	1128.67316	6			0.6929E-04					
25	16	A_1	25	15	A_2	1128.67316	6			0.6929E-04					
24	16	A_1	24	15	A_2	1128.75402	5			0.7034E-04					
24	16	A_2	24	15	A_1	1128.75402	5			0.7034E-04					
23	16	A_2	23	15	A_1	1128.83181	21			0.7030E-04					
23	16	A_1	23	15	A_2	1128.83181	21			0.7030E-04					
56	10	A_1	55	9	A_2	1128.85610	-63			0.2714E-04					
56	10	A_2	55	9	A_1	1128.85610	-63			0.2714E-04					
22	16	A_1	22	15	A_2	1128.90594	-5			0.6895E-04					
22	16	A_2	22	15	A_1	1128.90594	-5			0.6895E-04					
26	13	A_2	25	12	A_1	1128.96993	-5	.156607E-02	1.0	0.8086E-03	-3.26	3.289E-04	-.294E+00	-.442E+00	1.2
26	13	A_1	25	12	A_2	1128.96993	-5			0.8086E-03					
21	16	A_1	21	15	A_2	1128.97674	-40			0.6606E-04					
21	16	A_2	21	15	A_1	1128.97674	-40			0.6606E-04					
20	16	A_1	20	15	A_2	1129.04485	-21			0.6137E-04					
20	16	A_2	20	15	A_1	1129.04485	-21			0.6137E-04					
19	16	A_1	19	15	A_2	1129.10973	-1			0.5458E-04					
19	16	A_2	19	15	A_1	1129.10973	-1			0.5458E-04					
17	14	E	16	13	E	1129.16973	9	.964861E-03	1.0	0.9675E-03	-.27	4.841E-04			1.0
17	16	A_2	17	15	A_1	1129.22953	14			0.3350E-04					
17	16	A_1	17	15	A_2	1129.22953	14			0.3350E-04					
46	11	E	45	10	E	1129.24181	8			0.1345E-03					
36	12	E	35	11	E	1129.27498	40			0.4159E-03					
16	16	A_1	16	15	A_2	1129.28422	-15			0.1851E-04					
16	16	A_2	16	15	A_1	1129.28422	-15			0.1851E-04					
27	13	A_2	26	12	A_1	1129.76251	0	.143406E-02	0.9	0.7361E-03	-2.66	3.078E-04	-.309E+00		1.2
27	13	A_1	26	12	A_2	1129.76251	0			0.7361E-03					
47	11	E	46	10	E	1129.95943	7			0.1125E-03					
18	14	E	17	13	E	1129.99384	2	.905456E-03	0.6	0.9093E-03	-.42	4.018E-04	.191E+00	-.247E+00	1.2
37	12	E	36	11	E	1130.03048	2			0.3632E-03					
28	13	A_2	27	12	A_1	1130.55142	0	.137903E-02	0.7	0.6674E-03	3.21	5.910E-04	-.698E-01	.147E+01	1.3
28	13	A_1	27	12	A_2	1130.55142	0			0.6674E-03					
48	11	E	47	10	E	1130.67313	7			0.9378E-04					
38	12	E	37	11	E	1130.78261	6			0.3159E-03					
19	14	E	18	13	E	1130.81451	0	.847331E-03	1.2	0.8519E-03	-.54	4.799E-04			1.0
29	13	A_2	28	12	A_1	1131.33667	-1	.121683E-02	1.0	0.6025E-03	.97	3.961E-04	-.387E+00		1.1
29	13	A_1	28	12	A_2	1131.33667	-1			0.6025E-03					
49	11	E	48	10	E	1131.38287	7			0.7781E-04					
39	12	E	38	11	E	1131.53092	8			0.2735E-03					
20	14	E	19	13	E	1131.63167	-2	.790713E-03	1.6	0.7955E-03	-.61	4.764E-04			1.0
50	11	E	49	10	E	1132.08862	5			0.6429E-04					
30	13	A_2	29	12	A_1	1132.11828	0	.102648E-02	1.0	0.5417E-03	-5.55	3.812E-04	-.292E+00		1.1
30	13	A_1	29	12	A_2	1132.11828	0			0.5417E-03					
40	12	E	39	11	E	1132.27538	5			0.2358E-03					
21	14	E	20	13	E	1132.44536	1	.734946E-03	1.1	0.7403E-03	-.73	3.209E-04		-.459E+00	1.1
31	13	A_2	30	12	A_1	1132.89623	2	.977246E-03	1.2	0.4850E-03	.73	4.633E-04	-.457E+00		1.2
31	13	A_1	30	12	A_2	1132.89623	2			0.4850E-03					
41	12	E	40	11	E	1133.01603	4			0.2024E-03					
22	14	E	21	13	E	1133.25548	1	.680171E-03	1.2	0.6864E-03	-.91	6.702E-04	-.118E+00	.152E+01	1.2
32	13	A_2	31	12	A_1	1133.67047	2	.878418E-03	0.9	0.4324E-03	1.54	1.563E-04			1.0
32	13	A_1	31	12	A_2	1133.67047	2			0.4324E-03					
42	12	E	41	11	E	1133.75284	4			0.1730E-03					
23	14	E	22	13	E	1134.06205	2	.629560E-03	1.3	0.6340E-03	-.71	8.778E-04	-.510E+00	.221E+01	1.2

(cont.)

J	K_a	Γ	J'	K'_a	Γ'	$\nu^{a)}$	$\delta_\nu{}^{b)}$	$S_\nu^{\mathrm{exp}}(294.45)^{c)}$	$\Delta_S{}^{d)}$	$S_\nu^{\mathrm{calc}}(294.45)^{e)}$	$\delta_S{}^{f)}$	$\Gamma_0{}^{g)}$	$\Delta_2{}^{g)}$	$\nu_{\mathrm{VC}}{}^{g)}$	$R^{h)}$
	1			2		3	4	5	6	7	8	9	10	11	12
33	13	A_2	32	12	A_1	1134.44100	1	.789301E-03	0.7	0.3839E-03	2.72	1.663E-05		-.630E+00	1.4
33	13	A_1	32	12	A_2	1134.44100	1			0.3839E-03					
43	12	E	42	11	E	1134.48581	5			0.1473E-03					
24	14	E	23	13	E	1134.86499	-4	.576572E-03	1.7	0.5834E-03	-1.18	1.853E-04	-.124E+01		1.5
15	15	E	14	14	E	1135.03459	4			0.6333E-03					
36	17	E	36	16	E	1135.15127	-34			0.1463E-04					
34	13	A_2	33	12	A_1	1135.20783	2	.693135E-03	1.0	0.3394E-03	2.07	5.128E-04			1.0
34	13	A_1	33	12	A_2	1135.20783	2			0.3394E-03					
44	12	E	43	11	E	1135.21505	21			0.1248E-03					
32	17	E	32	16	E	1135.59847	9			0.2168E-04					
25	14	E	24	13	E	1135.66442	-2			0.5347E-03					
31	17	E	31	16	E	1135.70156	-40			0.2350E-04					
30	17	E	30	16	E	1135.80231	1			0.2526E-04					
16	15	E	15	14	E	1135.86534	2			0.5972E-03					
29	17	E	29	16	E	1135.89948	9			0.2692E-04					
45	12	E	44	11	E	1135.93990	-14			0.1053E-03					
35	13	A_2	34	12	A_1	1135.97092	2	.585076E-03	2.6	0.2987E-03	-2.12	3.356E-04			1.0
35	13	A_1	34	12	A_2	1135.97092	2			0.2987E-03					
28	17	E	28	16	E	1135.99319	-6			0.2842E-04					
27	17	E	27	16	E	1136.08386	0			0.2970E-04					
26	17	E	26	16	E	1136.17132	8			0.3067E-04					
25	20	E	26	19	E	1136.23880	-44			0.1187E-06					
25	17	E	25	16	E	1136.25551	14			0.3125E-04					
24	17	E	24	16	E	1136.33640	13			0.3135E-04					
23	17	E	23	16	E	1136.41396	4			0.3086E-04					
26	14	E	25	13	E	1136.46024	-1			0.4882E-03					
22	17	E	22	16	E	1136.48851	17			0.2967E-04					
21	17	E	21	16	E	1136.55956	4			0.2766E-04					
20	17	E	20	16	E	1136.62740	-6			0.2468E-04					
46	12	E	45	11	E	1136.66144	11			0.8847E-04					
19	17	E	19	16	E	1136.69265	49			0.2059E-04					
17	15	E	16	14	E	1136.69265	3			0.5618E-03					
36	13	A_2	35	12	A_1	1136.73025	1	.505775E-03	1.8	0.2619E-03	-3.55	3.120E-04			1.0
36	13	A_1	35	12	A_2	1136.73025	1			0.2619E-03					
18	17	E	18	16	E	1136.75435	72			0.1524E-04					
17	17	E	17	16	E	1136.81216	30			0.8441E-05					
27	14	E	26	13	E	1137.25243	-3			0.4439E-03					
47	12	E	46	11	E	1137.37802	-68			0.7400E-04					
37	13	A_2	36	12	A_1	1137.48582	1			0.2285E-03					
37	13	A_1	36	12	A_2	1137.48582	1			0.2285E-03					
18	15	E	17	14	E	1137.51648	4			0.5270E-03					
28	14	E	27	13	E	1138.04103	0			0.4020E-03					
48	12	E	47	11	E	1138.09219	6			0.6164E-04					
38	13	A_1	37	12	A_2	1138.23764	4			0.1986E-03					
38	13	A_2	37	12	A_1	1138.23764	4			0.1986E-03					
19	15	E	18	14	E	1138.33672	-5			0.4929E-03					
29	14	E	28	13	E	1138.82596	0			0.3626E-03					
39	13	A_1	38	12	A_2	1138.98565	5			0.1719E-03					
39	13	A_2	38	12	A_1	1138.98565	5			0.1719E-03					
20	15	E	19	14	E	1139.15357	-2			0.4595E-03					
30	14	E	29	13	E	1139.60724	1			0.3257E-03					
40	13	A_1	39	12	A_2	1139.72985	7			0.1481E-03					
40	13	A_2	39	12	A_1	1139.72985	7			0.1481E-03					
21	15	E	20	14	E	1139.96689	0			0.4269E-03					
31	14	E	30	13	E	1140.38502	20			0.2914E-03					
41	13	A_1	40	12	A_2	1140.47012	-2			0.1270E-03					
41	13	A_2	40	12	A_1	1140.47012	-2			0.1270E-03					
22	15	E	21	14	E	1140.77654	-10			0.3952E-03					
32	14	E	31	13	E	1141.15859	-14			0.2595E-03					
42	13	A_1	41	12	A_2	1141.20623	-43			0.1085E-03					
42	13	A_2	41	12	A_1	1141.20623	-43			0.1085E-03					
23	15	E	22	14	E	1141.58274	-10			0.3646E-03					
33	14	E	32	13	E	1141.92883	-11			0.2302E-03					
43	13	A_2	42	12	A_1	1141.93927	-5			0.9232E-04					
43	13	A_1	42	12	A_2	1141.93927	-5			0.9232E-04					
24	15	E	23	14	E	1142.38535	-12			0.3351E-03					
44	13	A_1	43	12	A_2	1142.66807	-3			0.7819E-04					
44	13	A_2	43	12	A_1	1142.66807	-3			0.7819E-04					
34	14	E	33	13	E	1142.69536	-6			0.2034E-03					
25	15	E	24	14	E	1143.18434	-18			0.3067E-03					
45	13	A_2	44	12	A_1	1143.39259	-41			0.6594E-04					
45	13	A_1	44	12	A_2	1143.39259	-41			0.6594E-04					
16	16	A_2	15	15	A_1	1143.41616	-1			0.3291E-03					
16	16	A_1	15	15	A_2	1143.41616	-1			0.3291E-03					
35	14	E	34	13	E	1143.45809	-9			0.1789E-03					
28	18	E	28	17	E	1143.59860	0			0.1234E-04					
26	15	E	25	14	E	1143.97987	-10			0.2797E-03					
36	14	E	35	13	E	1144.21712	-6			0.1567E-03					
17	16	A_2	16	15	A_1	1144.24306	-2			0.3090E-03					
17	16	A_1	16	15	A_2	1144.24306	-2			0.3090E-03					
27	15	E	26	14	E	1144.77169	-12			0.2541E-03					
37	14	E	36	13	E	1144.97237	-5			0.1366E-03					

(cont.)

J	K_a	Γ	J'	K'_a	Γ'	$\nu^{a)}$	$\delta_\nu{}^{b)}$	$S_\nu^{\rm exp}(294.45)^{c)}$	$\Delta_S{}^{d)}$	$S_\nu^{\rm calc}(294.45)^{e)}$	$\delta_S{}^{f)}$	$\Gamma_0{}^{g)}$	$\Delta_2{}^{g)}$	$\nu_{\rm VC}{}^{g)}$	$R^{h)}$
	1			2		3	4	5	6	7	8	9	10	11	12
18	16	A_1	17	15	A_2	1145.06648	-3			0.2894E-03					
18	16	A_2	17	15	A_1	1145.06648	-3			0.2894E-03					
28	15	E	27	14	E	1145.55980	-21			0.2299E-03					
38	14	E	37	13	E	1145.72378	-9			0.1187E-03					
23	21	E	24	20	E	1145.82614	37			0.1785E-07					
19	16	A_1	18	15	A_2	1145.88639	-6			0.2702E-03					
19	16	A_2	18	15	A_1	1145.88639	-6			0.2702E-03					
29	15	E	28	14	E	1146.34451	-7			0.2071E-03					
39	14	E	38	13	E	1146.47144	-10			0.1026E-03					
20	16	A_1	19	15	A_2	1146.70286	-2			0.2515E-03					
20	16	A_2	19	15	A_1	1146.70286	-2			0.2515E-03					
30	15	E	29	14	E	1147.12542	-6			0.1859E-03					
40	14	E	39	13	E	1147.21535	-4			0.8837E-04					
21	16	A_1	20	15	A_2	1147.51569	-9			0.2333E-03					
21	16	A_2	20	15	A_1	1147.51569	-9			0.2333E-03					
31	15	E	30	14	E	1147.90258	-13			0.1661E-03					
22	16	A_1	21	15	A_2	1148.32506	-8			0.2157E-03					
22	16	A_2	21	15	A_1	1148.32506	-8			0.2157E-03					
32	15	E	31	14	E	1148.67635	9			0.1479E-03					
23	16	A_2	22	15	A_1	1149.13089	-6			0.1987E-03					
23	16	A_1	22	15	A_2	1149.13089	-6			0.1987E-03					
43	14	E	42	13	E	1149.42387	-5			0.5500E-04					
33	15	E	32	14	E	1149.44578	-31			0.1311E-03					
33	15	E	32	14	E	1149.44589	-20			0.1311E-03					
24	16	A_2	23	15	A_1	1149.93296	-22			0.1824E-03					
24	16	A_1	23	15	A_2	1149.93296	-22			0.1824E-03					
34	15	E	33	14	E	1150.21204	-17			0.1157E-03					
25	16	A_1	24	15	A_2	1150.73167	-17			0.1668E-03					
25	16	A_2	24	15	A_1	1150.73167	-17			0.1668E-03					
31	19	A_2	31	18	A_1	1150.93235	-73			0.4313E-05					
31	19	A_1	31	18	A_2	1150.93235	-73			0.4313E-05					
35	15	E	34	14	E	1150.97434	-26			0.1017E-03					
26	19	A_1	26	18	A_2	1151.40288	0			0.5218E-05					
26	19	A_2	26	18	A_1	1151.40288	0			0.5218E-05					
25	19	A_1	25	18	A_2	1151.48771	60			0.5174E-05					
25	19	A_2	25	18	A_1	1151.48771	60			0.5174E-05					
26	16	A_1	25	15	A_2	1151.52684	-5			0.1520E-03					
26	16	A_2	25	15	A_1	1151.52684	-5			0.1520E-03					
24	19	A_1	24	18	A_2	1151.56874	65			0.5010E-05					
24	19	A_2	24	18	A_1	1151.56874	65			0.5010E-05					
36	15	E	35	14	E	1151.73306	-17			0.8900E-04					
17	17	E	16	16	E	1151.81877	4			0.1609E-03					
27	16	A_1	26	15	A_2	1152.31789	-44			0.1379E-03					
27	16	A_2	26	15	A_1	1152.31789	-44			0.1379E-03					
37	15	E	36	14	E	1152.48819	9			0.7757E-04					
18	17	E	17	16	E	1152.64150	-24			0.1504E-03					
28	16	A_2	27	15	A_1	1153.10622	8			0.1246E-03					
28	16	A_1	27	15	A_2	1153.10622	8			0.1246E-03					
38	15	E	37	14	E	1153.23913	-6			0.6733E-04					
19	17	E	18	16	E	1153.46159	33			0.1402E-03					
29	16	A_1	28	15	A_2	1153.89036	6			0.1122E-03					
29	16	A_2	28	15	A_1	1153.89036	6			0.1122E-03					
20	17	E	19	16	E	1154.27736	10			0.1303E-03					
30	16	A_1	29	15	A_2	1154.67069	-12			0.1006E-03					
30	16	A_2	29	15	A_1	1154.67069	-12			0.1006E-03					
40	15	E	39	14	E	1154.72967	-29			0.5008E-04					
21	17	E	20	16	E	1155.08963	-11			0.1208E-03					
31	16	A_2	30	15	A_1	1155.44753	-11			0.8984E-04					
31	16	A_1	30	15	A_2	1155.44753	-11			0.8984E-04					
22	17	E	21	16	E	1155.89817	-50			0.1115E-03					
32	16	A_1	31	15	A_2	1156.22093	15			0.7990E-04					
32	16	A_2	31	15	A_1	1156.22093	15			0.7990E-04					
23	17	E	22	16	E	1156.70397	-9			0.1026E-03					
24	17	E	23	16	E	1157.50601	14			0.9407E-04					
34	16	A_1	33	15	A_2	1157.75587	-7			0.6242E-04					
34	16	A_2	33	15	A_1	1157.75587	-7			0.6242E-04					
25	17	E	24	16	E	1158.30406	-3			0.8593E-04					
35	16	A_1	34	15	A_2	1158.51779	-13			0.5483E-04					
35	16	A_2	34	15	A_1	1158.51779	-13			0.5483E-04					
26	17	E	25	16	E	1159.09796	-76			0.7820E-04					
23	20	E	23	19	E	1159.29137	83			0.1595E-05					
27	17	E	26	16	E	1159.88897	-76			0.7090E-04					
37	16	A_2	36	15	A_1	1160.02987	-74			0.4178E-04					
37	16	A_1	36	15	A_2	1160.02987	-74			0.4178E-04					
18	18	E	17	17	E	1160.24009	31			0.7393E-04					
28	17	E	27	16	E	1160.67710	0			0.6402E-04					
19	18	E	18	17	E	1161.05890	5			0.6883E-04					
29	17	E	28	16	E	1161.46078	-6			0.5759E-04					
20	18	E	19	17	E	1161.87441	1			0.6389E-04					
30	17	E	29	16	E	1162.24080	-11			0.5160E-04					
21	18	E	20	17	E	1162.68664	22			0.5912E-04					
31	17	E	30	16	E	1163.01740	9			0.4604E-04					

(cont.)

J K_a Γ	J' K'_a Γ'	$\nu^{a)}$	$\delta_\nu{}^{b)}$	$S_\nu^{\rm exp}(294.45)^{c)}$	$\Delta_S{}^{d)}$	$S_\nu^{\rm calc}(294.45)^{e)}$	$\delta_S{}^{f)}$	$\Gamma_0{}^{g)}$	$\Delta_2{}^{g)}$	$\nu_{\rm VC}{}^{g)}$	$R^{h)}$
1	2	3	4	5	6	7	8	9	10	11	12
22 18 E	21 17 E	1163.49503	13			0.5453E-04					
32 17 E	31 16 E	1163.78981	-20			0.4092E-04					
23 18 E	22 17 E	1164.29986	4			0.5012E-04					
33 17 E	32 16 E	1164.55903	2			0.3622E-04					
25 18 E	24 17 E	1165.89894	0			0.4190E-04					
26 18 E	25 17 E	1166.69323	13			0.3809E-04					
23 21 E	23 20 E	1166.95096	-14			0.4785E-06					
27 18 E	26 17 E	1167.48342	-23			0.3450E-04					
37 17 E	36 16 E	1167.59704	-61			0.2133E-04					
28 18 E	27 17 E	1168.27080	24			0.3113E-04					
19 19 A_2	18 18 A_1	1168.67688	5			0.3193E-04					
19 19 A_1	18 18 A_2	1168.67688	5			0.3193E-04					
29 18 E	28 17 E	1169.05385	3			0.2798E-04					
20 19 A_2	19 18 A_1	1169.49204	15			0.2960E-04					
20 19 A_1	19 18 A_2	1169.49204	15			0.2960E-04					
30 18 E	29 17 E	1169.83348	5			0.2505E-04					
21 19 A_2	20 18 A_1	1170.30359	16			0.2736E-04					
21 19 A_1	20 18 A_2	1170.30359	16			0.2736E-04					
22 19 A_2	21 18 A_1	1171.11167	25			0.2521E-04					
22 19 A_1	21 18 A_2	1171.11167	25			0.2521E-04					
32 18 E	31 17 E	1171.38086	-73			0.1984E-04					
23 19 A_1	22 18 A_2	1171.91583	-2			0.2315E-04					
23 19 A_2	22 18 A_1	1171.91583	-2			0.2315E-04					
24 19 A_2	23 18 A_1	1172.71630	-41			0.2118E-04					
24 19 A_1	23 18 A_2	1172.71630	-41			0.2118E-04					
25 19 A_1	24 18 A_2	1173.51422	25			0.1931E-04					
25 19 A_2	24 18 A_1	1173.51422	25			0.1931E-04					
26 19 A_2	25 18 A_1	1174.30760	-4			0.1754E-04					
26 19 A_1	25 18 A_2	1174.30760	-4			0.1754E-04					
27 19 A_2	26 18 A_1	1175.09785	17			0.1588E-04					
27 19 A_1	26 18 A_2	1175.09785	17			0.1588E-04					
28 19 A_2	27 18 A_1	1175.88408	-1			0.1432E-04					
28 19 A_1	27 18 A_2	1175.88408	-1			0.1432E-04					
29 19 A_2	28 18 A_1	1176.66679	-6			0.1286E-04					
29 19 A_1	28 18 A_2	1176.66679	-6			0.1286E-04					
20 20 E	19 19 E	1177.12792	62			0.1294E-04					
22 20 E	21 19 E	1178.74584	6			0.1100E-04					
24 20 E	23 19 E	1180.34984	-16			0.9225E-05					
25 20 E	24 19 E	1181.14680	6			0.8405E-05					
26 20 E	25 19 E	1181.94005	18			0.7629E-05					
