# Supplementary material for: Effective Dipole Moment Model for Axially Symmetric C3v Molecules: Application to the Precise Study of Absolute Line Strengths of the ν6 Fundamental of CH335Cl
Source: Int J Mol Sci. 2023 Jul 28;24(15):12122. doi: 10.3390/ijms241512122 (PMC10418978; doi:10.3390/ijms241512122)
Supplement: Supplementary file 1 [file ijms-24-12122-s001.zip › Supplementary Data S2.pdf]

Supplementary material 2

Ro–vibrational term values for the v_6 vibrational state of the $CH_3{}^{35}Cl$ molecule (in cm^{-1})

Electronic supplementary materials to:

Effective dipole moment model for axially symmetric C_{3v} molecules: Application to the precise study of absolute line strengths of the ν_6 fundamental of CH_3Cl

O. N. Ulenikov[‡,a], E. S. Bekhtereva[a], O. V. Gromova[a], A. L. Fomchenko[a], Yu. B. Morzhikova[a], S. S. Sidko[a], C. Sydow[b], and S. Bauerecker[§,b]

[a] **Research School of High–Energy Physics, National Research Tomsk Polytechnic University, Tomsk 634050, Russia;**

[b] **Institut für Physikalische und Theoretische Chemie, Technische Universität Braunschweig, D - 38106, Braunschweig, Germany.**

Ro–vibrational term values for the v_6 vibrational state of the $CH_3{}^{35}Cl$ molecule (in cm^{-1})$^{a)}$.

J K Γ	E^{exp}	Δ	δ
1	2	3	4
1 0 E	1018.95434	2	0
1 1 A_1	1021.12358	1	4
1 1 A_2	1021.12259		2
1 1 E	1026.36326		-1
2 0 E	1020.72141	1	3
2 1 A_1	1022.88870	1	2
2 1 A_2	1022.89165		5
2 1 E	1028.13024	0	0
2 2 E	1034.63621	2	4
2 2 A_1	1045.11498		-4
2 2 A_2	1045.11498		-4
3 0 E	1023.37195	4	2
3 1 A_1	1025.54370		4
3 1 A_2	1025.53785	1	2
3 1 E	1030.78066	1	0
3 2 E	1037.28680	2	6
3 2 A_1	1047.76518	2	-3
3 2 A_2	1047.76518	2	-3
3 3 E	1058.60755	3	2
3 3 E	1074.32179		-7
4 0 E	1026.90593	4	1
4 1 A_1	1029.06997	1	2
4 1 A_2	1029.07969		3
4 1 E	1034.31447	3	0
4 2 E	1040.82081	3	4
4 2 A_1	1051.29871	1	-3
4 2 A_2	1051.29871	1	-3
4 3 E	1062.14146	2	5
4 3 E	1077.85496	2	-3
4 4 A_1	1093.03230	2	5
4 4 A_2	1093.03230	2	5
4 4 E	1113.97733		-6
5 0 E	1031.32331	1	0
5 1 A_1	1033.49958		2
5 1 A_2	1033.48501	1	1
5 1 E	1038.73164	2	1
5 2 E	1045.23823	3	3
5 2 A_1	1055.71552	2	-2
5 2 A_2	1055.71552	2	-2
5 3 E	1066.55864	8	-1
5 3 E	1082.27127	4	-2
5 4 A_1	1097.44922	2	4
5 4 A_2	1097.44922	2	4
5 4 E	1118.39302	3	-5
5 5 E	1137.90328	1	3
5 5 A_1	1164.07304		3
5 5 A_2	1164.07304		3
6 0 E	1036.62402	2	2
6 1 A_1	1038.78290	1	1
6 1 A_2	1038.80328		0
6 1 E	1044.03207	3	1
6 2 E	1050.53899	3	4
6 2 A_1	1061.01552	2	-2
6 2 A_2	1061.01552	2	-2
6 3 E	1071.85924	1	5
6 3 E	1087.57064	2	-6
6 4 A_1	1102.74930	8	-3
6 4 A_2	1102.74930	8	-3
6 4 E	1123.69171	8	0
6 5 E	1143.20289	4	3
6 5 A_1	1169.37076		2
6 5 A_2	1169.37076		2
6 6 E	1193.21121		0
6 6 E	1224.59789		1
7 0 E	1042.80794	2	2
7 1 A_1	1044.99081	-	7
7 1 A_2	1044.96352	2	-2
7 1 E	1050.21567	4	0
7 2 E	1056.72298	2	3
7 2 A_1	1067.19864	3	-1
7 2 A_2	1067.19864	3	-1
7 3 E	1078.04292	3	-1
7 3 E	1093.75308	2	-3
7 4 A_1	1108.93266	2	4
7 4 A_2	1108.93266	2	4
7 4 E	1129.87320	4	-5
7 5 E	1149.38552	1	0
7 5 A_1	1175.55118	4	-2
7 5 A_2	1175.55118	4	-2
7 6 E	1199.39307	7	3
7 6 E	1230.77713	21	7
7 7 A_1	1258.94454	4	-3
7 7 A_2	1258.94454	4	-3
7 7 E	1295.53902		9
8 0 E	1049.87499	3	2
8 1 A_1	1052.02686	2	0
8 1 A_2	1052.06183		0
8 1 E	1057.28237	3	0
8 2 E	1063.79011	2	3
8 2 A_1	1074.26477	2	0
8 2 A_2	1074.26477	2	0
8 3 E	1085.10974	6	-2
8 3 E	1100.81845	7	1
8 4 A_1	1115.99898	2	3
8 4 A_2	1115.99898	2	3
8 4 E	1136.93760	9	3
8 5 E	1156.45108	8	-4
8 5 A_1	1182.61425	5	-4
8 5 A_2	1182.61425	5	-4
8 6 E	1206.45773	3	2
8 6 E	1237.83872	2	0
8 7 A_1	1266.00803	7	-5
8 7 A_2	1266.00803	7	-5
8 7 E	1302.59892		1
8 8 E	1335.08956	1	-1
8 8 A_1	1376.88095		5
8 8 A_2	1376.88095		5
9 0 E	1057.82504	0	0
9 1 A_1	1060.01644		2
9 1 A_2	1059.97274	1	2
9 1 E	1065.23203	2	0
9 2 E	1071.74027	3	3

J K Γ	E^{exp}	Δ	δ
1	2	3	4
9 2 A_1	1082.21378	3	0
9 2 A_2	1082.21378	3	-1
9 3 E	1093.05960	8	2
9 3 E	1108.76654	2	-3
9 4 A_1	1123.94824	3	4
9 4 A_2	1123.94824	3	4
9 4 E	1144.88457	8	4
9 5 E	1164.39958	4	2
9 5 A_1	1190.55987	3	-2
9 5 A_2	1190.55987	3	-2
9 6 E	1214.40510	6	2
9 6 E	1245.78276	13	7
9 7 A_1	1273.95415	5	-2
9 7 A_2	1273.95415	5	-2
9 7 E	1310.54103	3	1
9 8 E	1343.03411	0	-1
9 8 A_1	1384.82095	5	6
9 8 A_2	1384.82095	5	6
9 9 E	1421.63023	6	-1
9 9 E	1468.60642		7
10 0 E	1066.65799	1	2
10 1 A_1	1068.80103	0	3
10 1 A_2	1068.85442		2
10 1 E	1074.06450	3	-2
10 2 E	1080.57334	1	5
10 2 A_1	1091.04554	4	-3
10 2 A_2	1091.04554	4	-3
10 3 E	1101.89229	1	3
10 3 E	1117.59733	3	-3
10 4 A_1	1132.78027	1	3
10 4 A_2	1132.78027	1	3
10 4 E	1153.71410	8	2
10 5 E	1173.23072	5	3
10 5 A_1	1199.38786	5	-3
10 5 A_2	1199.38786	5	-3
10 6 E	1223.23504	3	-1
10 6 E	1254.60888	1	0
10 7 A_1	1282.78268	5	-1
10 7 A_2	1282.78268	5	-1
10 7 E	1319.36514	4	2
10 8 E	1351.86095	1	0
10 8 A_1	1393.64269	3	5
10 8 A_2	1393.64269	3	5
10 9 E	1430.45509	5	0
10 9 E	1477.42551	7	4
10 10 A_1	1518.54838	5	0
10 10 A_2	1518.54838	5	0
10 10 E	1570.69559		0
11 0 E	1076.37365	2	1
11 1 A_1	1078.57564		2
11 1 A_2	1078.51157	2	2
11 1 E	1083.77970	2	-1
11 2 E	1090.28914	6	4
11 2 A_1	1100.75991	1	-6
11 2 A_2	1100.75991	1	-5
11 3 E	1111.60769	1	4
11 3 E	1127.31062	3	-5
11 4 A_1	1142.49495	2	2
11 4 A_2	1142.49495	2	2
11 4 E	1163.42599	5	-1
11 5 E	1182.94440	5	1
11 5 A_1	1209.09808	5	-4
11 5 A_2	1209.09808	5	-4
11 6 E	1232.94744	3	-1
11 6 E	1264.31710	5	-2
11 7 A_1	1292.49350	5	-1
11 7 A_2	1292.49350	5	-1
11 7 E	1329.07107	3	0
11 8 E	1361.56988	0	0
11 8 A_1	1403.34605	5	4
11 8 A_2	1403.34605	5	4
11 9 E	1440.16186	5	-1
11 9 E	1487.12593		-2
11 10 A_1	1528.25273	1	2
11 10 A_2	1528.25273	1	2
11 10 E	1580.39287	7	-1
11 11 E	1625.82364		1
11 11 A_1	1683.12673		-8
11 11 A_2	1683.12673		-8
12 0 E	1086.97187	1	0
12 1 A_1	1089.10422	1	2
12 1 A_2	1089.17994		2
12 1 E	1094.37741	2	-1
12 2 E	1100.88749	5	0
12 2 A_1	1111.35680	4	-2
12 2 A_2	1111.35680	4	-3
12 3 E	1122.20561	2	2
12 3 E	1137.90629	4	-4
12 4 A_1	1153.09212	1	2
12 4 A_2	1153.09212	1	2
12 4 E	1174.02012	5	-3
12 5 E	1193.54045	4	-2
12 5 A_1	1219.69039	4	-4
12 5 A_2	1219.69039	4	-4
12 6 E	1243.54211	3	-3
12 6 E	1274.90725	12	-1
12 7 A_1	1303.08644	3	-2
12 7 A_2	1303.08644	3	-2
12 7 E	1339.65873	4	1
12 8 E	1372.16079	2	0
12 8 A_1	1413.93090	3	7
12 8 A_2	1413.93090	3	7
12 9 E	1450.75041	5	0
12 9 E	1497.70766	6	4
12 10 A_1	1538.83857	4	0
12 10 A_2	1538.83857	4	0
12 10 E	1590.97108		1
12 11 E	1636.40650		-1
12 11 A_1	1693.70118		-1
12 11 A_2	1693.70118		-1
12 12 E	1743.43340		-1
12 12 E	1805.87590		-8

J K Γ	E^{exp}	Δ	δ
1	2	3	4
13 0 E	1098.45251	2	1
13 1 A_1	1100.66713	-	1
13 1 A_2	1100.57882	2	2
13 1 E	1105.85747	6	-2
13 2 E	1112.36828	4	-2
13 2 A_1	1122.83593	4	-4
13 2 A_2	1122.83593	4	-2
13 3 E	1133.68594	2	3
13 3 E	1149.38416	5	-2
13 4 A_1	1164.57161	1	2
13 4 A_2	1164.57161	1	2
13 4 E	1185.49635	4	-2
13 5 E	1205.01881	4	2
13 5 A_1	1231.16463	7	-2
13 5 A_2	1231.16463	7	-2
13 6 E	1255.01892	5	0
13 6 E	1286.37912	3	-1
13 7 A_1	1314.56135	5	-3
13 7 A_2	1314.56135	5	-3
13 7 E	1351.12788	4	0
13 8 E	1383.63347	3	-2
13 8 A_1	1425.39701	8	7
13 8 A_2	1425.39701	8	7
13 9 E	1462.22054	6	-1
13 9 E	1509.17034	6	3
13 10 A_1	1550.30584	10	3
13 10 A_2	1550.30584	10	3
13 10 E	1602.43000		1
13 11 E	1647.87051	1	1
13 11 A_1	1705.15596	2	-3
13 11 A_2	1705.15596	2	-3
13 12 E	1754.89392	10	9
13 12 E	1817.32620		-8
13 13 A_1	1871.35298	2	-1
13 13 A_2	1871.35298	2	-1
13 13 E	1938.91668	-	-22
14 0 E	1110.81531	2	-2
14 1 A_1	1112.93515	1	1
14 1 A_2	1113.03704		1
14 1 E	1118.21970	4	-2
14 2 E	1124.73133	5	-1
14 2 A_1	1135.19715	6	-3
14 2 A_2	1135.19715	6	-5
14 3 E	1146.04842		0
14 3 E	1161.74399	1	-4
14 4 A_1	1176.93323	1	3
14 4 A_2	1176.93323	1	3
14 4 E	1197.85445	6	0
14 5 E	1217.37919	10	6
14 5 A_1	1243.52055	5	-3
14 5 A_2	1243.52055	5	-3
14 6 E	1267.37762	3	0
14 6 E	1298.73257	6	4
14 7 A_1	1326.91804	4	-3
14 7 A_2	1326.91804	4	-3
14 7 E	1363.47840	4	3
14 8 E	1395.98776	5	-3
14 8 A_1	1437.74420	10	6
14 8 A_2	1437.74420	10	6
14 9 E	1474.57214	1	5
14 9 E	1521.51386	9	3
14 10 A_1	1562.65426	6	3
14 10 A_2	1562.65426	6	3
14 10 E	1614.76943	4	-3
14 11 E	1660.21542	5	-1
14 11 A_1	1717.49103	10	3
14 11 A_2	1717.49103	10	3
14 12 E	1767.23496	6	5
14 12 E	1829.65637	4	-10
14 13 A_1	1883.68985	5	0
14 13 A_2	1883.68985	5	0
14 13 E	1951.24172		-15
14 14 E	2009.55543	5	-1
14 14 A_1	2082.22112		-11
14 14 A_2	2082.22112		-11
15 0 E	1124.06015	3	-2
15 1 A_1	1126.28946		0
15 1 A_2	1126.17301	3	-1
15 1 E	1131.46392	6	1
15 2 E	1137.97642	6	1
15 2 A_1	1148.44032	5	0
15 2 A_2	1148.44032	5	2
15 3 E	1159.29293	2	2
15 3 E	1174.98570	8	3
15 4 A_1	1190.17676	4	2
15 4 A_2	1190.17676	4	2
15 4 E	1211.09418	3	-1
15 5 E	1230.62140	10	9
15 5 A_1	1256.75799	5	-4
15 5 A_2	1256.75799	5	-4
15 6 E	1280.61799	11	-5
15 6 E	1311.96720	15	-7
15 7 A_1	1340.15630	6	-2
15 7 A_2	1340.15630	6	-2
15 7 E	1376.71001	6	2
15 8 E	1409.22347	3	-2
15 8 A_1	1450.97228	10	5
15 8 A_2	1450.97228	10	6
15 9 E	1487.80479	15	-5
15 9 E	1534.73803	8	5
15 10 A_1	1575.88364	4	1
15 10 A_2	1575.88364	4	1
15 10 E	1627.98929	2	3
15 11 E	1673.44110	1	1
15 11 A_1	1730.70601	4	-3
15 11 A_2	1730.70601	4	-3
15 12 E	1780.45642	3	-2
15 12 E	1842.86625	8	-8
15 13 A_1	1896.90684	7	-2
15 13 A_2	1896.90684	7	-2
15 13 E	1964.44600	7	-14
15 14 E	2022.76750	7	-6

J K Γ	E^{exp}	Δ	δ
1	2	3	4
15 14 A_1	2095.41934	4	-15
15 14 A_2	2095.41934	4	-15
15 15 E	2158.01168		-4
15 15 E	2235.75834		-14
16 0 E	1138.18676	3	-2
16 1 A_1	1140.29224	1	1
16 1 A_2	1140.42415		-2
16 1 E	1145.58982	1	-2
16 2 E	1152.10329		0
16 2 A_1	1162.56511	5	2
16 2 A_2	1162.56511	5	0
16 3 E	1173.41919	1	1
16 3 E	1189.10891	8	3
16 4 A_1	1204.30195	5	-3
16 4 A_2	1204.30195	5	-3
16 4 E	1225.21537	3	-2
16 5 E	1244.74512	9	3
16 5 A_1	1270.87673	5	-4
16 5 A_2	1270.87673	5	-4
16 6 E	1294.73995	3	0
16 6 E	1326.08310	8	-2
16 7 A_1	1354.27592	4	-1
16 7 A_2	1354.27592	4	-1
16 7 E	1390.82251	8	-1
16 8 E	1423.34039	5	1
16 8 A_1	1465.08104	7	5
16 8 A_2	1465.08104	7	5
16 9 E	1501.91857	5	0
16 9 E	1548.84253	4	0
16 10 A_1	1589.99380	2	1
16 10 A_2	1589.99380	2	1
16 10 E	1642.08917	2	-1
16 11 E	1687.54731	6	4
16 11 A_1	1744.80089	2	1
16 11 A_2	1744.80089	2	1
16 12 E	1794.55820	6	-1
16 12 E	1856.95560	3	-5
16 13 A_1	1911.00378		-3
16 13 A_2	1911.00378		-3
16 13 E	1978.52948	10	-1
16 14 E	2036.85928	2	0
16 14 A_1	2109.49638		-6
16 14 A_2	2109.49638		-6
16 15 E	2172.09782	1	0
16 15 E	2249.82850		-6
16 16 A_1	2316.69058	7	-6
16 16 A_2	2316.69058	7	-6
16 16 E	2399.49611		12
17 0 E	1153.19494	3	0
17 1 A_1	1155.44097	-	2
17 1 A_2	1155.29251	5	-3
17 1 E	1160.59725	4	-3
17 2 E	1167.11176	3	0
17 2 A_1	1177.57129	5	-6
17 2 A_2	1177.57129	5	1
17 3 E	1188.42698	2	0
17 3 E	1204.11346	7	3
17 4 A_1	1219.30869	1	0
17 4 A_2	1219.30869	1	0
17 4 E	1240.21778	6	-2
17 5 E	1259.75025	5	-1
17 5 A_1	1285.87654	7	-3
17 5 A_2	1285.87654	7	-3
17 6 E	1309.74312	2	-1
17 6 E	1341.07985	2	-1
17 7 A_1	1369.27664	6	-2
17 7 A_2	1369.27664	6	-2
17 7 E	1405.81573	4	1
17 8 E	1438.33823	4	2
17 8 A_1	1480.07018	14	-1
17 8 A_2	1480.07018	14	-1
17 9 E	1516.91310	8	4
17 9 E	1563.82730	8	2
17 10 A_1	1604.98448	4	0
17 10 A_2	1604.98448	4	0
17 10 E	1657.06901	2	2
17 11 E	1702.53373	2	1
17 11 A_1	1759.77532	3	2
17 11 A_2	1759.77532	3	2
17 12 E	1809.54000	3	2
17 12 E	1871.92415	2	-5
17 13 A_1	1925.98046	1	-1
17 13 A_2	1925.98046	1	-1
17 13 E	1993.49168	5	-3
17 14 E	2051.83043	5	4
17 14 A_1	2124.45178	4	-6
17 14 A_2	2124.45178	4	-6
17 15 E	2187.06296		3
17 15 E	2264.77670		3
17 16 A_1	2331.64938	9	3
17 16 A_2	2331.64938	9	3
17 16 E	2414.43644		11
17 17 E	2485.55890		4
18 0 E	1169.08435	6	-4
18 1 A_1	1171.17370	2	1
18 1 A_2	1171.33957		3
18 1 E	1176.48595	3	-3
18 2 E	1183.00154	6	-2
18 2 A_1	1193.45875	8	2
18 2 A_2	1193.45875	8	-2
18 3 E	1204.31608	1	0
18 3 E	1219.99908	5	0
18 4 A_1	1235.19664	9	1
18 4 A_2	1235.19664	9	1
18 4 E	1256.10114	3	-4
18 5 E	1275.63655	3	-2
18 5 A_1	1301.75714	4	-5
18 5 A_2	1301.75714	4	-5
18 6 E	1325.62732	4	0
18 6 E	1356.95721	7	-1
18 7 A_1	1385.15824	6	-2
18 7 A_2	1385.15824	6	-2

J K Γ	E^{exp}	Δ	δ
1	2	3	4
18 7 E	1421.68934	6	-1
18 8 E	1454.21676	3	2
18 8 A_1	1495.93961	9	3
18 8 A_2	1495.93961	9	2
18 9 E	1532.78806	11	1
18 9 E	1579.69200	8	5
18 10 A_1	1620.85546	4	0
18 10 A_2	1620.85546	4	0
18 10 E	1672.92848	7	4
18 11 E	1718.40020	3	-1
18 11 A_1	1775.62904	3	0
18 11 A_2	1775.62904	3	0
18 12 E	1825.40152		0
18 12 E	1887.77169	4	-4
18 13 A_1	1941.83656	11	-3
18 13 A_2	1941.83656	11	-3
18 13 E	2009.33243	12	-10
18 14 E	2067.68065		2
18 14 A_1	2140.28540	6	-5
18 14 A_2	2140.28540	6	-5
18 15 E	2202.90684	5	-1
18 15 E	2280.60255	26	-2
18 16 A_1	2347.48647		0
18 16 A_2	2347.48647		0
18 16 E	2430.25425		26
18 17 E	2501.38865		-9
18 18 E	2664.58105	-	8
19 0 E	1185.85487	4	-1
19 1 A_1	1188.11976		9
19 1 A_2	1187.93545	3	1
19 1 E	1193.25565	4	-2
19 2 E	1199.77242	5	-1
19 2 A_1	1210.22706	6	-6
19 2 A_2	1210.22706	6	-2
19 3 E	1221.08620	2	0
19 3 E	1236.76554	3	-2
19 4 A_1	1251.96553	2	0
19 4 A_2	1251.96553	2	0
19 4 E	1272.86524	5	-2
19 5 E	1292.40373	3	-2
19 5 A_1	1318.51837	10	1
19 5 A_2	1318.51837	10	1
19 6 E	1342.39227	4	0
19 6 E	1373.71496	5	-1
19 7 A_1	1401.92047	4	-1
19 7 A_2	1401.92047	4	-1
19 7 E	1438.44313	8	-1
19 8 E	1470.97574	3	2
19 8 A_1	1512.68896	6	5
19 8 A_2	1512.68896	6	5
19 9 E	1549.54330	4	1
19 9 E	1596.43632	6	3
19 10 A_1	1637.60644	6	-2
19 10 A_2	1637.60644	6	-2
19 10 E	1689.66727	3	-1
19 11 E	1735.14650	9	3
19 11 A_1	1792.36184	5	0
19 11 A_2	1792.36184	5	0
19 12 E	1842.14255	6	-1
19 12 E	1904.49798	8	0
19 13 A_1	1958.57190	8	-1
19 13 A_2	1958.57190	8	-1
19 13 E	2026.05168	3	-2
19 14 E	2084.40973	9	-2
19 14 A_1	2156.99706	6	5
19 14 A_2	2156.99706	6	5
19 15 E	2219.62926	3	-2
19 15 E	2297.30601	9	3
19 16 A_1	2364.20170	3	-3
19 16 A_2	2364.20170	3	-3
19 16 E	2446.94870		-2
19 18 E	2681.28044		-4
19 19 A_1	2853.71949		5
19 19 A_2	2853.71949		5
20 0 E	1203.50610	5	-2
20 1 A_1	1205.57750	3	1
20 1 A_2	1205.78109		1
20 1 E	1210.90611	8	2
20 2 E	1217.42410	4	-1
20 2 A_1	1227.87608	5	0
20 2 A_2	1227.87608	5	-4
20 3 E	1238.73708	3	0
20 3 E	1254.41258	3	-1
20 4 A_1	1269.61513	3	1
20 4 A_2	1269.61513	3	1
20 4 E	1290.50975	3	-2
20 5 E	1310.05154	7	2
20 5 A_1	1336.15978	6	-3
20 5 A_2	1336.15978	6	-3
20 6 E	1360.03770	4	0
20 6 E	1391.35282	6	1
20 7 A_1	1419.56305	1	2
20 7 A_2	1419.56305	1	2
20 7 E	1456.07686	9	3
20 8 E	1488.61486	3	0
20 8 A_1	1530.31794	5	4
20 8 A_2	1530.31794	5	4
20 9 E	1567.17849	6	0
20 9 E	1614.06004	6	1
20 10 A_1	1655.23724	1	4
20 10 A_2	1655.23724	1	4
20 10 E	1707.28521	2	0
20 11 E	1752.77224	5	0
20 11 A_1	1809.97345	5	1
20 11 A_2	1809.97345	5	1
20 12 E	1859.76280	8	-2
20 12 E	1922.10268		0
20 13 A_1	1976.18616	4	0
20 13 A_2	1976.18616	4	0
20 13 E	2043.64891	7	-3
20 14 E	2102.01747	4	0
20 14 A_1	2174.58628	4	4

Continued

J	K	Γ	E^{exp}	Δ	δ
1			2	3	4
20	14	A_2	2174.58628	4	4
20	15	E	2237.22991	4	-5
20	16	A_1	2381.79480	12	-7
20	16	A_2	2381.79480	12	-7
20	16	E	2464.52036		13
20	17	E	2535.68148	7	1
20	18	E	2698.85705		1
20	19	A_1	2871.28711		14
20	19	A_2	2871.28711		14
21	0	E	1222.03784	4	1
21	1	A_1	1224.32346		-1
21	1	A_2	1224.09955	4	-1
21	1	E	1229.43691	4	-2
21	2	E	1235.95628	6	-2
21	2	A_1	1246.40544	4	-4
21	2	A_2	1246.40544	4	0
21	3	E	1257.26842	2	-1
21	3	E	1272.93988	6	0
21	4	A_1	1288.14508	1	-3
21	4	A_2	1288.14508	1	-3
21	4	E	1309.03445	5	2
21	5	E	1328.57959	3	-2
21	5	A_1	1354.68127	7	2
21	5	A_2	1354.68127	7	2
21	6	E	1378.56335	6	3
21	6	E	1409.87048	7	2
21	7	A_1	1438.08560	4	-3
21	7	A_2	1438.08560	4	-3
21	7	E	1474.59014	10	3
21	8	E	1507.13390	3	2
21	8	A_1	1548.82631	5	6
21	8	A_2	1548.82631	5	6
21	9	E	1585.69340	4	2
21	9	E	1632.56293	3	6
21	10	A_1	1673.74741	3	0
21	10	A_2	1673.74741	3	0
21	10	E	1725.78199	2	2
21	11	E	1771.27723	2	2
21	11	A_1	1828.46352	1	-1
21	11	A_2	1828.46352	1	-1
21	12	E	1878.26202	2	1
21	12	E	1940.58554	2	1
21	13	A_1	1994.67903	9	-1
21	13	A_2	1994.67903	9	-1
21	13	E	2062.12396		0
21	14	E	2120.50349	1	0
21	14	A_1	2193.05287	2	2
21	14	A_2	2193.05287	2	2
21	15	E	2255.70867	10	7
21	15	E	2333.34427	13	3
21	16	A_1	2400.26552		-7
21	16	A_2	2400.26552		-7
21	17	E	2554.14376		2
21	18	E	2717.31039		3
21	19	A_1	2889.73096		15
21	19	A_2	2889.73096		15
21	20	E	3071.36840		-13
22	0	E	1241.44967	6	-2
22	1	A_1	1243.50137		1
22	1	E	1248.84787	5	-3
22	2	E	1255.36869	4	-1
22	2	A_1	1265.81490	7	4
22	2	A_2	1265.81490	7	0
22	3	E	1276.67991	6	-3
22	3	E	1292.34712	4	-1
22	4	A_1	1307.55526	8	6
22	4	A_2	1307.55526	8	6
22	4	E	1328.43888	4	-3
22	5	E	1347.98766	3	-3
22	5	A_1	1374.08239	8	2
22	5	A_2	1374.08239	8	2
22	6	E	1397.96881	3	-1
22	6	E	1429.26762	7	1
22	7	A_1	1457.48794	4	-3
22	7	A_2	1457.48794	4	-3
22	7	E	1493.98273	10	4
22	8	E	1526.53248	4	1
22	8	A_1	1568.21371	6	5
22	8	A_2	1568.21371	6	5
22	9	E	1605.08767	4	3
22	9	E	1651.94454	3	3
22	10	A_1	1693.13678		1
22	10	A_2	1693.13678		1
22	10	E	1745.15725	3	2
22	11	E	1790.66109	5	0
22	11	A_1	1847.83186	6	5
22	11	A_2	1847.83186	6	5
22	12	E	1897.63985	3	1
22	12	E	1959.94613	11	-9
22	13	A_1	2014.05025	1	0
22	13	A_2	2014.05025	1	0
22	13	E	2081.47649	4	3
22	14	E	2139.86754	5	2
22	14	A_1	2212.39651		-3
22	14	A_2	2212.39651		-1
22	15	E	2275.06486	12	-3
22	15	E	2352.67858		8
22	16	A_1	2419.61354	2	-5
22	16	A_2	2419.61354	2	-5
22	17	E	2573.48294		4
22	18	E	2736.64015		2
22	19	A_1	2909.05084		19
22	19	A_2	2909.05084		19
22	20	E	3090.67792	-	2
22	21	E	3281.48342	35	2
23	0	E	1261.74138	3	0
23	1	A_1	1264.04995		0
23	1	A_2	1263.78251	3	-4
23	1	E	1269.13866	6	0
23	2	E	1275.66098	5	-1
23	2	A_1	1286.10404	8	0
23	2	A_2	1286.10404	8	3
23	3	E	1296.97130	1	1
23	3	E	1312.63400	4	-2
23	4	A_1	1327.84510	10	4
23	4	A_2	1327.84510	10	4
23	4	E	1348.72291		0
23	5	E	1368.27546	2	0
23	5	A_1	1394.36282	7	-3
23	5	A_2	1394.36282	7	-3
23	6	E	1418.25387	3	-2
23	6	E	1449.54402	4	8
23	7	A_1	1477.76970	5	-3
23	7	A_2	1477.76970	5	-3
23	7	E	1514.25429	9	4
23	8	E	1546.81034	3	3
23	8	A_1	1588.47986	4	5
23	8	A_2	1588.47986	4	5
23	9	E	1625.36099	3	3
23	9	E	1672.20465	6	2
23	10	A_1	1713.40493	3	-3
23	10	A_2	1713.40493	3	-3
23	10	E	1765.41069		0
23	11	E	1810.92356	2	1
23	11	A_1	1868.07796	6	-1
23	11	A_2	1868.07796	6	-1
23	12	E	1917.89595	7	-3
23	12	E	1980.18443	6	-2
23	13	A_1	2034.29946	4	-1
23	13	A_2	2034.29946	4	-1
23	13	E	2101.70618	4	6
23	14	E	2160.10924	4	0
23	14	A_1	2232.61705	19	6
23	14	A_2	2232.61705	19	6
23	15	E	2295.29849	8	-3
23	15	E	2372.88918		9
23	16	A_1	2439.83861	15	6
23	16	A_2	2439.83861	15	6
23	17	E	2593.69859	6	-3
23	18	E	2756.84607		4
23	19	A_1	2929.24615		-2
23	19	A_2	2929.24628		-2
23	20	E	3110.86248	25	0
24	0	E	1282.91255	2	-2
24	1	A_1	1284.94280	5	0
24	1	A_2	1285.23338		-1
24	1	E	1290.30886	5	-4
24	2	E	1296.83281	4	-3
24	2	A_1	1307.27257	3	1
24	2	A_2	1307.27257	3	-2
24	3	E	1318.14212	4	-3
24	3	E	1333.80021	1	-1
24	4	A_1	1349.01432	9	-4
24	4	A_2	1349.01432	9	-4
24	4	E	1369.88609	5	0
24	5	E	1389.44258	1	0
24	5	A_1	1415.52234	5	-1
24	5	A_2	1415.52234	5	-1
24	6	E	1439.41817	5	-3
24	6	E	1470.69915		4
24	7	A_1	1498.93055	5	-3
24	7	A_2	1498.93055	5	-3
24	7	E	1535.40447	11	3
24	8	E	1567.96708	8	1
24	8	A_1	1609.62439	5	2
24	8	A_2	1609.62439	5	2
24	9	E	1646.51304	7	4
24	9	E	1693.34288	4	-1
24	10	A_1	1734.55163		-2
24	10	A_2	1734.55163		-2
24	10	E	1786.54202	2	2
24	11	E	1832.06427	5	0
24	11	A_1	1889.20167	3	0
24	11	A_2	1889.20167	3	0
24	12	E	1939.03010	2	0
24	12	E	2001.29988	4	0
24	13	A_1	2055.42628	10	-9
24	13	A_2	2055.42628	10	-9
24	13	E	2122.81269	6	8
24	14	E	2181.22828	1	-3
24	14	A_1	2253.71382	1	-3
24	14	A_2	2253.71382	1	-3
24	15	E	2316.40911	8	-4
24	15	E	2393.97579		12
24	16	A_1	2460.94007	14	-7
24	16	A_2	2460.94007	14	-7
24	17	E	2614.79065	5	9
24	20	E	3131.92184		-10
25	0	E	1304.96289	5	-2
25	1	A_1	1307.29649		0
25	1	A_2	1306.98179		2
25	1	E	1312.35823	7	-1
25	2	E	1318.88392	7	1
25	2	A_1	1329.32015	3	-3
25	2	A_2	1329.32015	3	-1
25	3	E	1340.19215	3	-3
25	3	E	1355.84535	4	-1
25	4	A_1	1371.06278	10	2
25	4	A_2	1371.06278	10	2
25	4	E	1391.92809	3	0
25	5	E	1411.48870	1	-1
25	5	A_1	1437.56055	5	2
25	5	A_2	1437.56055	5	2
25	6	E	1461.46135		-3
25	6	E	1492.73280	5	1
25	7	A_1	1520.97013	5	-4
25	7	A_2	1520.97013	5	-4
25	7	E	1557.43290	11	-3
25	8	E	1590.00243	7	2
25	8	A_1	1631.64701	6	3
25	8	A_2	1631.64701	6	3
25	9	E	1668.54343	3	1
25	9	E	1715.35891		-4
25	10	A_1	1756.57648		-1
25	10	A_2	1756.57648		-1
25	10	E	1808.55085	1	3
25	11	E	1854.08283	15	-5
25	11	A_1	1911.20259	3	3
25	11	A_2	1911.20259	3	3
25	12	E	1961.04185	4	1
25	12	E	2023.29218	4	3
25	13	A_1	2077.43061	4	1
25	13	A_2	2077.43061	4	1
25	13	E	2144.79555	18	-2
25	14	E	2203.22438	1	-1
25	14	A_1	2275.68677		-4
25	14	A_2	2275.68677		-4
25	15	E	2338.39637	11	-7
25	15	E	2415.93790		-1
25	16	A_1	2482.91796	10	-5
25	16	A_2	2482.91796	10	-5
25	17	E	2636.75842	9	3
25	18	E	2799.88490		0
25	19	A_1	2972.26308		14
25	19	A_2	2972.26308		14
25	20	E	3153.85597		3
26	0	E	1327.89203	1	1
26	1	A_1	1329.89908	5	-1
26	1	A_2	1330.23890		-1
26	1	E	1335.28631	6	-3
26	2	E	1341.81380	3	-2
26	2	A_1	1352.24643	4	-2
26	2	A_2	1352.24643	4	-2
26	3	E	1363.12100	2	0
26	3	E	1378.76905	6	-4
26	4	A_1	1393.98987	3	-2
26	4	A_2	1393.98987	3	-2
26	4	E	1414.84858	7	2
26	5	E	1434.41346	2	-1
26	5	A_1	1460.47703	5	0
26	5	A_2	1460.47703	5	0
26	6	E	1484.38306	5	-3
26	6	E	1515.64456	7	-4
26	7	A_1	1543.88812	17	-1
26	7	A_2	1543.88812	17	-1
26	7	E	1580.33937	9	2
26	8	E	1612.91597	5	2
26	8	A_1	1654.54730	5	1
26	8	A_2	1654.54730	5	1
26	9	E	1691.45186	5	1
26	9	E	1738.25242	6	-3
26	10	A_1	1779.47909	3	-3
26	10	A_2	1779.47909	3	-3
26	10	E	1831.43680	3	1
26	11	E	1876.97910	8	5
26	11	A_1	1934.08027	9	-1
26	11	A_2	1934.08027	9	-1
26	12	E	1983.93088	2	2
26	12	E	2046.16097	1	7
26	13	A_1	2100.31179	3	-1
26	13	A_2	2100.31179	3	-1
26	13	E	2167.65474	11	10
26	14	E	2226.09708	2	-3
26	14	A_1	2298.53532		-15
26	14	A_2	2298.53532		-15
26	15	E	2361.25998	6	-4
26	15	E	2438.77547		4
26	16	A_1	2505.77178	4	-2
26	16	A_2	2505.77178	4	-2
26	17	E	2659.60178		5
26	18	E	2822.71728		12
26	19	A_1	2995.08344	2	-2
26	19	A_2	2995.08344	2	-2
26	20	E	3176.66419		9
27	0	E	1351.69949	7	-4
27	1	A_1	1354.06026		1
27	1	A_2	1353.69437	4	-1
27	1	E	1359.09279	8	-2
27	2	E	1365.62219	7	-3
27	2	A_1	1376.05103	4	0
27	2	A_2	1376.05103	4	-2
27	3	E	1386.92822	3	-3
27	3	E	1402.57101	3	-3
27	4	A_1	1417.79534	3	-3
27	4	A_2	1417.79534	3	-3
27	4	E	1438.64715	7	3
27	5	E	1458.21651	2	2
27	5	A_1	1484.27148	6	2
27	5	A_2	1484.27148	6	2
27	6	E	1508.18291	5	-3
27	6	E	1539.43417	7	0
27	7	A_1	1567.68404	4	-6
27	7	A_2	1567.68404	4	-6
27	7	E	1604.12334	11	3
27	8	E	1636.70734	3	3
27	8	A_1	1678.32495	4	3
27	8	A_2	1678.32495	4	3
27	9	E	1715.23790	6	-1
27	9	E	1762.02298	9	-2
27	10	A_1	1803.25918		1
27	10	A_2	1803.25918		1
27	10	E	1855.19955	5	3
27	11	E	1900.75233	5	-4
27	11	A_1	1957.83450	0	4
27	11	A_2	1957.83450	0	4
27	12	E	2007.69675	6	-2
27	12	E	2069.90582	4	4
27	13	A_1	2124.06955	10	-5
27	13	A_2	2124.06955	10	-5
27	13	E	2191.38944	18	-1
27	14	E	2249.84608	1	-2
27	15	E	2384.99955	15	3
27	15	E	2462.48793		7
27	16	A_1	2529.50106	29	-7
27	16	A_2	2529.50106	29	-7
27	17	E	2683.32022		0
27	18	E	2846.42408		-6
27	19	A_1	3018.77838		12
27	19	A_2	3018.77838		12
28	0	E	1376.38503	7	-1
28	1	A_1	1378.36729	4	3
28	1	A_2	1378.76007		-5
28	1	E	1383.77718	10	-8
28	2	E	1390.30869	4	-1
28	2	A_1	1400.73351	3	-5
28	2	A_2	1400.73351	3	0
28	3	E	1411.61350	1	-3
28	3	E	1427.25081	11	1
28	4	A_1	1442.47879	3	-3
28	4	A_2	1442.47879	3	-3
28	4	E	1463.32336	4	-1
28	5	E	1482.89736	4	-2
28	5	A_1	1508.94344	6	1
28	5	A_2	1508.94344	6	1
28	6	E	1532.86054	2	-1
28	6	E	1564.10101	6	-9
28	7	A_1	1592.35768	6	0
28	7	A_2	1592.35768	6	0
28	7	E	1628.78443	9	0
28	8	E	1661.37615	5	3
28	8	A_1	1702.97949	4	2
28	8	A_2	1702.97949	4	2
28	9	E	1739.90120	4	-1
28	9	E	1786.67021	6	0
28	10	A_1	1827.91621		-2
28	10	A_2	1827.91621		-2
28	10	E	1879.83863	2	0
28	11	E	1925.40245	5	-2
28	11	A_1	1982.46474	2	5
28	11	A_2	1982.46474	2	5
28	12	E	2032.33919	3	2
28	12	E	2094.52644	4	8
28	13	A_1	2148.70359	2	0
28	13	A_2	2148.70359	2	0
28	13	E	2215.99970	9	9
28	14	E	2274.47096	1	0
28	15	E	2409.61452	15	-3
28	15	E	2487.07483		2
28	16	A_1	2554.10564	5	2
28	16	A_2	2554.10564	5	2
28	17	E	2707.91343	3	-3
28	18	E	2871.00555	12	11
28	19	A_1	3043.34692		-2
28	19	A_2	3043.34692		-2
29	0	E	1401.94814	4	-1
29	1	A_1	1404.33812		1
29	1	A_2	1403.91731	3	0
29	1	E	1409.33932	6	5
29	2	E	1415.87281	5	-5
29	2	A_1	1426.29352	3	2
29	2	A_2	1426.29352	3	-6
29	3	E	1437.17644	5	1
29	3	E	1452.80799	1	2
29	4	A_1	1468.03979	4	-3
29	4	A_2	1468.03979	4	-3
29	4	E	1488.87690	4	0
29	5	E	1508.45572	3	-2
29	5	A_1	1534.49259	2	5
29	5	A_2	1534.49259	2	5
29	6	E	1558.41548	2	-1
29	6	E	1589.64497	15	-1
29	7	A_1	1617.90845	8	0
29	7	A_2	1617.90845	8	0
29	7	E	1654.32225	14	-6
29	8	E	1686.92198	5	2
29	8	A_1	1728.51053	8	-1
29	8	A_2	1728.51053	8	-1
29	9	E	1765.44137	2	2
29	9	E	1812.19366	8	-2
29	10	A_1	1853.44990		-1
29	10	A_2	1853.44990		-1
29	10	E	1905.35368	3	-2
29	11	E	1950.92896	3	3
29	11	A_1	2007.97054	19	-4
29	11	A_2	2007.97054	19	-4
29	12	E	2057.85760	9	-7
29	12	E	2120.02237	10	11
29	13	A_1	2174.21340	9	2
29	13	A_2	2174.21340	9	2
29	13	E	2241.48471	16	0
29	14	E	2299.97131	0	0
29	15	E	2435.10468	5	-2
29	15	E	2512.53589		2
29	16	A_1	2579.58487	4	3
29	16	A_2	2579.58487	4	3
29	17	E	2733.38106	7	1
29	18	E	2896.46069		2
29	19	A_1	3068.78903		-6
29	19	A_2	3068.78903		-6
30	0	E	1428.38843	4	1
30	1	A_1	1430.34406	9	-6
30	1	A_2	1430.79380		-1
30	1	E	1435.77843	6	0
30	2	E	1442.31425	4	-3
30	2	A_1	1452.73062	9	-6
30	2	A_2	1452.73062	9	6
30	3	E	1463.61651	3	-2
30	3	E	1479.24207	4	-5
30	4	A_1	1494.47791		-4
30	4	A_2	1494.47791		-4
30	4	E	1515.30733	5	3
30	5	E	1534.89114	4	1
30	5	A_1	1560.91838	4	2
30	5	A_2	1560.91838	4	2
30	6	E	1584.84736	2	0
30	6	E	1616.06539	11	-1
30	7	A_1	1644.33606	5	5
30	7	A_2	1644.33606	5	5
30	7	E	1680.73653	12	2
30	8	E	1713.34440	7	-1
30	8	A_1	1754.91774	13	4
30	8	A_2	1754.91774	13	4
30	9	E	1791.85788	5	-2
30	9	E	1838.59292	9	-6
30	10	A_1	1879.85976	3	-2
30	10	A_2	1879.85976	3	-2
30	10	E	1931.74436	4	3
30	11	E	1977.33135	4	1
30	11	A_1	2034.35174	2	5
30	11	A_2	2034.35174	2	5
30	12	E	2084.25185	2	1
30	12	E	2146.39313	4	9
30	13	A_1	2200.59855	1	1
30	13	A_2	2200.59855	1	1
30	13	E	2267.84443	2	11
30	14	E	2326.34670		1
30	15	E	2461.46954	6	-1
30	16	A_1	2605.93837	11	-3
30	16	A_2	2605.93837	11	-3
30	17	E	2759.72252	6	-5
30	18	E	2922.78945		5
30	19	A_1	3095.10424		-5
30	19	A_2	3095.10424		-5
31	0	E	1455.70541	8	-1
31	1	A_2	1458.12677		0
31	1	A_1	1457.64726	11	2
31	1	E	1463.09432	6	2
31	2	E	1469.63252	10	-1
31	2	A_1	1480.04434	5	0
31	2	A_2	1480.04434	5	-9
31	3	E	1490.93341	5	0
31	3	E	1506.55281	4	-1
31	4	A_1	1521.79273	1	-5
31	4	A_2	1521.79273	1	-5
31	4	E	1542.61411	4	-1
31	5	E	1562.20311	4	-2
31	5	A_2	1588.22048	4	3
31	5	A_1	1588.22048	4	3
31	6	E	1612.15570	4	-2
31	6	E	1643.36194	11	2
31	7	A_1	1671.63991	12	1
31	7	A_2	1671.63991	12	1
31	7	E	1708.02656	13	-4
31	8	E	1740.64308	-	5
31	8	A_1	1782.20048	13	-5
31	8	A_2	1782.20048	13	-5
31	9	E	1819.15041	5	-2
31	9	E	1865.86768	7	-1
31	10	A_2	1907.14540	1	-1
31	10	A_1	1907.14540	1	-1
31	10	E	1959.01011	6	5
31	11	E	2004.60928	3	1
31	11	A_1	2061.60767	3	6
31	11	A_2	2061.60767	3	6
31	12	E	2111.52127	2	2
31	12	E	2173.63820	20	-7
31	13	A_1	2227.85865	5	1
31	13	A_2	2227.85865	5	1
31	13	E	2295.07803	5	1
31	14	E	2353.59678	10	9
31	15	E	2488.70862	9	-4
31	16	A_2	2633.16582	7	-3
31	16	A_1	2633.16582	7	-3
31	17	E	2786.93762		4
31	18	E	2949.99129		9
32	0	E	1483.89867	8	-2
32	1	A_1	1485.82622	7	-2
32	1	A_2	1486.33652		-2
32	1	E	1491.28642	6	1
32	2	E	1497.82709	2	-7
32	3	E	1519.12663	11	3
32	3	E	1534.73962	13	0
32	4	A_2	1549.98386	5	0
32	4	A_1	1549.98386	5	0
32	4	E	1570.79694	5	2
32	5	E	1590.39128	5	-1
32	5	A_1	1616.39837	6	0
32	5	A_2	1616.39837	6	0
32	6	E	1640.34010	3	-2
32	6	E	1671.53406	6	-2
32	7	A_2	1699.81976	6	6
32	7	A_1	1699.81976	6	6
32	7	E	1736.19211	8	-3
32	8	E	1768.81740	6	2
32	8	A_2	1810.35849	16	-7
32	8	A_1	1810.35849	16	-7
32	9	E	1847.31851	2	2
32	9	E	1894.01733	6	-1
32	10	A_1	1935.30637	4	2
32	10	A_2	1935.30637	4	2
32	10	E	1987.15048	3	2
32	11	E	2032.76226		1
32	11	A_2	2089.73791	2	5
32	11	A_1	2089.73791	2	5
32	12	E	2139.66547	1	2
32	12	E	2201.75760		9
32	13	A_1	2255.99324	1	1
32	13	A_2	2255.99324	1	1
32	13	E	2323.18543	12	9
32	14	E	2381.72083	10	-3
32	15	E	2516.82163	5	3
32	16	A_1	2661.26678	11	3
32	16	A_2	2661.26678	11	3
32	17	E	2815.02558	15	-5
32	19	A_2	3150.35214		2

Continued

J K Γ	E^{exp}	Δ	δ
1	2	3	4
32 19 A_1	3150.35214		2
33 0 E	1512.96776	6	-1
33 1 A_2	1515.42268		2
33 1 A_1	1514.88060	8	-3
33 1 E	1520.35431	1	-1
33 2 E	1526.89770	4	0
33 3 E	1548.19567		2
33 3 E	1563.80207	10	2
33 4 A_2	1579.05074	2	1
33 4 A_1	1579.05074	2	1
33 4 E	1599.85523	4	0
33 5 E	1619.45511	5	-3
33 5 A_1	1645.45165	10	1
33 5 A_2	1645.45165	10	1
33 6 E	1669.40008	3	0
33 6 E	1700.58144	9	2
33 7 A_2	1728.87495	7	3
33 7 A_1	1728.87495	7	3
33 7 E	1765.23262	10	-2
33 8 E	1797.86695	25	-3
33 8 A_1	1839.39131	7	-2
33 8 A_2	1839.39131	7	-2
33 9 E	1876.36164	4	3
33 9 E	1923.04145	8	-2
33 10 A_1	1964.34210		-3
33 10 A_2	1964.34210		-3
33 10 E	2016.16507	2	2
33 11 E	2061.78984	9	2
33 11 A_1	2118.74205	3	5
33 11 A_2	2118.74205	3	5
33 12 E	2168.68399	3	2
33 12 E	2230.75037	5	9
33 13 A_2	2285.00186	1	1
33 13 A_1	2285.00186	1	1
33 13 E	2352.16591		7
33 14 E	2410.71870	7	-3
33 15 E	2545.80784	16	-4
33 16 A_2	2690.24059		-3
33 16 A_1	2690.24059		-3
33 17 E	2843.98628		1
33 18 E	3007.01222		2
33 19 A_1	3179.28388		5
33 19 A_2	3179.28388		5
34 0 E	1542.91217	2	0
34 1 A_1	1544.81002	17	7
34 1 A_2	1545.38466	-	2
34 1 E	1550.29753	4	0
34 2 E	1556.84365	4	-4
34 3 E	1578.14004	1	-4
34 3 E	1593.73965	10	2
34 4 A_1	1608.99289	3	-1
34 4 A_2	1608.99289	3	-1
34 4 E	1629.78859	5	3
34 5 E	1649.39413	5	-6
34 5 A_1	1675.37983	10	4
34 5 A_2	1675.37983	10	4
34 6 E	1699.33511	3	-3
34 6 E	1730.50346	10	-1
34 7 A_2	1758.80513	7	5
34 7 A_1	1758.80513	7	5
34 7 E	1795.14766	5	1
34 8 E	1827.79140	15	4
34 8 A_2	1869.29833	8	-3
34 8 A_1	1869.29833	8	-3
34 9 E	1906.27931	7	-1
34 9 E	1952.93962		1
34 10 A_1	1994.25223	4	-4
34 10 A_2	1994.25223	4	-4
34 10 E	2046.05331	8	-4
34 11 E	2091.69151	10	-1
34 11 A_2	2148.61957	6	3
34 11 A_1	2148.61957	6	3
34 12 E	2198.57635		1
34 12 E	2260.61620	7	8
34 13 A_1	2314.88398	8	-3
34 13 A_2	2314.88398	8	-3
34 13 E	2382.01910	8	8
34 14 E	2440.58980	4	-2
34 15 E	2575.66701	2	-3
34 16 A_1	2720.08694	10	-5
34 16 A_2	2720.08694	10	-5
34 19 A_1	3209.08677		-1
34 19 A_2	3209.08677		-1
35 0 E	1573.73140	4	1
35 1 A_2	1576.22195		-4
35 1 A_1	1575.61370	9	0
35 1 E	1581.11550	7	-4
35 2 E	1587.66459	7	-3
35 3 E	1608.95937	4	-1
35 3 E	1624.55185	5	-1
35 4 A_1	1639.80983	10	-5
35 4 A_2	1639.80983	10	-5
35 4 E	1660.59646	6	4
35 5 E	1680.20794	4	-3
35 5 A_1	1706.18235	10	2
35 5 A_2	1706.18235	10	2
35 6 E	1730.14476	5	-4
35 6 E	1761.29973	15	2
35 7 A_2	1789.60970	6	-1
35 7 A_1	1789.60970	6	-1
35 7 E	1825.93663	10	-2
35 8 E	1858.59006	9	3
35 8 A_1	1900.07912	11	-5
35 8 A_2	1900.07912	11	-5
35 9 E	1937.07116	6	4
35 9 E	1983.71122	6	-3
35 10 A_2	2025.03628		0
35 10 A_1	2025.03628		0
35 10 E	2076.81488	2	0
35 11 E	2122.46686	5	3
35 11 A_2	2179.37004	6	5
35 11 A_1	2179.37004	6	5

J K Γ	E^{exp}	Δ	δ
1	2	3	4
35 12 E	2229.34207	8	2
35 12 E	2291.35460	2	8
35 13 A_1	2345.63918	8	-5
35 13 A_2	2345.63918	8	-5
35 13 E	2412.74436	7	-4
35 14 E	2471.33356	7	-8
35 15 E	2606.39858		-1
35 16 A_2	2750.80536	10	-2
35 16 A_1	2750.80536	10	-2
35 17 E	2904.52340	19	4
36 0 E	1605.42501	11	9
36 1 A_1	1607.29140	5	3
36 1 A_2	1607.93418		-1
36 1 E	1612.80786	3	1
36 2 E	1619.35995	7	-5
36 3 E	1640.65301	4	-5
36 3 E	1656.23825	10	2
36 4 A_2	1671.50114	5	-1
36 4 A_1	1671.50114	5	-2
36 4 E	1692.27833	9	3
36 5 E	1711.89593	13	-1
36 5 A_2	1737.85876	10	3
36 5 A_1	1737.85876	10	3
36 6 E	1761.82853	3	-1
36 6 E	1792.96957	7	-8
36 7 A_1	1821.28834	3	7
36 7 A_2	1821.28834	3	7
36 7 E	1857.59912	9	-2
36 8 E	1890.26250	5	3
36 8 A_1	1931.73316	8	-7
36 8 A_2	1931.73316	8	-7
36 9 E	1968.73651	4	2
36 9 E	2015.35586	9	-4
36 10 A_2	2056.69367	5	2
36 10 A_1	2056.69367	5	2
36 10 E	2108.44908	8	-4
36 11 E	2154.11534	7	8
36 11 A_2	2210.99281	10	-2
36 11 A_1	2210.99281	10	-2
36 12 E	2260.98062	20	1
36 12 E	2322.96503	7	6
36 13 A_1	2377.26698	4	-1
36 13 A_2	2377.26698	4	-1
36 13 E	2444.34140	11	-6
36 14 E	2502.94965		-4
36 15 E	2638.00190		-11
36 16 A_1	2782.39528	4	1
36 16 A_2	2782.39528	4	1
36 17 E	2936.09875		-6
37 0 E	1637.99226	6	4
37 1 A_2	1640.52072		0
37 1 A_1	1639.84247	1	3
37 1 E	1645.37395	4	2
37 2 E	1651.92926	4	-3
37 3 E	1673.22057	5	-3
37 3 E	1688.79824	10	2
37 4 A_2	1704.06622	1	1
37 4 A_1	1704.06622	1	1
37 4 E	1724.83376	11	8
37 5 E	1744.45763	2	2
37 5 A_2	1770.40852	10	4
37 5 A_1	1770.40852	10	4
37 6 E	1794.38587	9	2
37 6 E	1825.51273	9	-1
37 7 A_1	1853.84031	6	5
37 7 A_2	1853.84031	6	5
37 7 E	1890.13455	13	-5
37 8 E	1922.80815	15	-2
37 8 A_2	1964.25993	12	-9
37 8 A_1	1964.25993	12	-9
37 9 E	2001.27496	4	3
37 9 E	2047.87298	7	-3
37 10 A_2	2089.22387	5	1
37 10 A_1	2089.22387	5	1
37 10 E	2140.95559	3	5
37 11 E	2186.63632	8	4
37 11 A_1	2243.48756	6	1
37 11 A_2	2243.48756	6	1
37 12 E	2293.49152	1	2
37 12 E	2355.44700	17	5
37 13 A_2	2409.76677	7	0
37 13 A_1	2409.76677	7	0
37 14 E	2535.43743	4	0
37 15 E	2670.47679	8	1
37 16 A_1	2814.85613	5	0
37 16 A_2	2814.85613	5	0
38 0 E	1671.43279	7	2
38 1 A_1	1673.26636	10	0
38 1 A_2	1673.98102		-3
38 1 E	1678.81322	8	-2
38 2 E	1685.37194	2	-2
38 3 E	1706.66140	4	-4
38 3 E	1722.23126	7	-3
38 4 A_2	1737.50451	2	0
38 4 A_1	1737.50451	2	0
38 4 E	1758.26204	2	2
38 5 E	1777.89240	6	-1
38 5 A_1	1803.83106	9	2
38 5 A_2	1803.83106	9	2
38 6 E	1827.81623	7	4
38 6 E	1858.92848	14	-2
38 7 A_2	1887.26515	7	2
38 7 A_1	1887.26515	7	2
38 7 E	1923.54245	11	-3
38 8 E	1956.22661	4	3
38 8 A_2	1997.65895	5	-6
38 8 A_1	1997.65895	5	-6
38 9 E	2034.68591	7	3
38 9 E	2081.26203	5	-4
38 10 A_2	2122.62640	4	3
38 10 A_1	2122.62640	4	3
38 10 E	2174.33361	3	-1

J K Γ	E^{exp}	Δ	δ
1	2	3	4
38 11 E	2220.02936	4	1
38 11 A_2	2276.85363	7	3
38 11 A_1	2276.85363	7	3
38 12 E	2326.87408	14	-6
38 12 E	2388.79995	3	2
38 13 A_1	2443.13807	2	3
38 13 A_2	2443.13807	2	3
38 14 E	2568.79630	6	-4
38 15 E	2703.82236	6	-1
38 16 A_1	2848.18750	11	6
38 16 A_2	2848.18750	11	6
38 17 E	3001.86091	19	-2
39 0 E	1705.74600	4	0
39 1 A_2	1708.31459		-1
39 1 A_1	1707.56256	7	-2
39 1 E	1713.12527	12	5
39 2 E	1719.68745	2	-2
39 3 E	1740.97500	3	-4
39 3 E	1756.53684	8	-4
39 4 A_2	1771.81552	5	3
39 4 A_1	1771.81552	5	3
39 4 E	1792.56275	9	0
39 5 E	1812.19977	6	-4
39 5 A_1	1838.12588	6	3
39 5 A_2	1838.12588	6	3
39 6 E	1862.11900	6	0
39 6 E	1893.21629	9	-3
39 7 A_2	1921.56235	10	2
39 7 A_1	1921.56235	10	2
39 7 E	1957.82219	11	-5
39 8 E	1990.51715	8	0
39 8 A_2	2031.92957	12	-7
39 8 A_1	2031.92957	12	-7
39 9 E	2068.96883	4	3
39 10 A_1	2156.90069	10	7
39 10 A_2	2156.90069	10	7
39 10 E	2208.58280	3	1
39 11 E	2254.29392	7	-1
39 11 A_2	2311.09047	7	4
39 11 A_1	2311.09047	7	4
39 12 E	2361.12805	5	2
39 12 E	2423.02327	5	-7
39 13 A_2	2477.38024	4	0
39 13 A_1	2477.38024	4	0
39 14 E	2603.02588		2
39 15 E	2738.03821	7	-1
39 16 A_2	2882.38865	10	1
39 16 A_1	2882.38865	10	1
39 17 E	3036.04653		2
40 0 E	1740.93136	4	3
40 1 A_1	1742.73052	9	-3
40 1 A_2	1743.52081		-2
40 1 E	1748.30929	4	-1
40 2 E	1754.87527	4	2
40 3 E	1776.16079	7	-5
40 3 E	1791.71442	8	0
40 4 A_2	1806.99856	4	-3
40 4 A_1	1806.99856	4	-3
40 4 E	1827.73542		10
40 5 E	1847.37924	5	0
40 5 A_1	1873.29237	8	3
40 5 A_2	1873.29237	8	3
40 6 E	1897.29370	10	-1
40 6 E	1928.37560	7	-4
40 7 A_2	1956.73131	9	2
40 7 A_1	1956.73131	9	2
40 7 E	1992.97325	11	-4
40 8 E	2025.67929	3	-2
40 8 A_1	2067.07126	12	-8
40 8 A_2	2067.07126	12	-8
40 9 E	2104.12309	9	-3
40 9 E	2150.65376	3	1
40 10 A_1	2192.04608	4	3
40 10 A_2	2192.04608	4	3
40 10 E	2243.70244	10	-5
40 11 E	2289.42947	3	4
40 11 A_1	2346.19753	8	5
40 11 A_2	2346.19753	8	5
40 12 E	2396.25262		4
40 12 E	2458.11663	10	-1
40 13 A_2	2512.49282	5	1
40 13 A_1	2512.49282	5	1
40 14 E	2638.12545	8	2
40 15 E	2773.12381	25	4
40 16 A_1	2917.45916		-1
40 16 A_2	2917.45916		-1
41 0 E	1776.98817	4	-1
41 1 A_2	1779.59913		0
41 1 A_1	1778.76963	12	-4
41 1 E	1784.36489	3	0
41 2 E	1790.93475	15	3
41 3 E	1812.21826	3	0
41 3 E	1827.76334	3	1
41 4 A_1	1843.05318	6	-5
41 4 A_2	1843.05318	6	-5
41 4 E	1863.77915	25	1
41 5 E	1883.43011	7	1
41 5 A_2	1909.32996	6	3
41 5 A_1	1909.32996	6	3
41 6 E	1933.33976	9	1
41 6 E	1964.40590	20	2
41 7 A_1	1992.77148	12	4
41 7 A_2	1992.77148	12	4
41 7 E	2028.99499	11	-8
41 8 E	2061.71255		6
41 8 A_2	2103.08358	17	5
41 8 A_1	2103.08358	17	5
41 9 E	2140.14831	18	5
41 9 E	2186.65523	1	0
41 10 A_2	2228.06211	6	3
41 10 A_1	2228.06211	6	3
41 10 E	2279.69212	4	-2

J K Γ	E^{exp}	Δ	δ
1	2	3	4
41 11 E	2325.43522	19	-7
41 11 A_1	2382.17424		8
41 11 A_2	2382.17424		8
41 12 E	2432.24720	27	-1
41 12 E	2494.07922	5	0
41 13 A_1	2548.47518	4	2
41 13 A_2	2548.47518	4	2
41 14 E	2674.09444	7	-1
41 15 E	2809.07845	10	2
41 16 A_2	2953.39842	8	-3
41 16 A_1	2953.39842	8	-3
41 17 E	3107.02388		0
42 0 E	1813.91596	7	0
42 1 A_1	1815.67931		-4
42 1 E	1821.29139	4	-2
42 2 E	1827.86529	19	2
42 3 E	1849.14669	6	0
42 3 E	1864.68300	9	-1
42 4 A_1	1879.97882	0	1
42 4 A_2	1879.97882	0	1
42 4 E	1900.69357	14	-3
42 5 E	1920.35181	3	0
42 5 A_1	1946.23804	9	1
42 5 A_2	1946.23804	9	1
42 6 E	1970.25653	3	1
42 6 E	2001.30644	15	-2
42 7 A_2	2029.68212	13	-4
42 7 A_1	2029.68212	13	-4
42 7 E	2065.88693	12	-5
42 8 E	2098.61603	1	-5
42 8 A_1	2139.96560	7	-1
42 8 A_2	2139.96560	7	-1
42 9 E	2177.04361	11	0
42 9 E	2223.52630	5	-5
42 10 A_1	2264.94811	2	0
42 10 A_2	2264.94811	2	0
42 10 E	2316.55108	16	-5
42 11 E	2362.31095	6	4
42 11 A_1	2419.01990	4	2
42 11 A_2	2419.01990	4	2
42 12 E	2469.11135	2	2
42 12 E	2530.91039	9	-11
42 13 A_2	2585.32671	6	1
42 13 A_1	2585.32671	6	1
42 15 E	2845.90159	5	-3
42 16 A_1	2990.20583		-4
42 16 A_2	2990.20583		-4
43 0 E	1851.71407	10	4
43 1 A_2	1854.36955		-1
43 1 A_1	1853.45899	5	2
43 1 E	1859.08822	7	0
43 2 E	1865.66630	8	-2
43 3 E	1886.94547	6	-5
43 3 E	1902.47282	3	-2
43 4 A_1	1917.77466	13	-6
43 4 A_2	1917.77466	13	-6
43 4 E	1938.47810	12	0
43 5 E	1958.14375	4	-1
43 5 A_1	1984.01599	13	-2
43 5 A_2	1984.01599	13	-2
43 6 E	2008.04344	16	4
43 6 E	2039.07670	15	-4
43 7 A_2	2067.46285	5	-1
43 7 A_1	2067.46285	5	-1
43 7 E	2103.64833	18	-6
43 8 E	2136.38951	4	4
43 8 A_2	2177.71694	7	-4
43 8 A_1	2177.71694	7	-4
43 9 E	2214.80860	6	2
43 9 E	2261.26650	1	1
43 10 A_2	2302.70350	4	-3
43 10 A_1	2302.70350	4	-3
43 10 E	2354.27888	2	2
43 11 E	2400.05568	6	2
43 11 A_1	2456.73404	3	2
43 11 A_2	2456.73404	3	2
43 12 E	2506.84435	2	3
43 12 E	2568.60987	5	1
43 13 A_2	2623.04682	5	0
43 13 A_1	2623.04682	5	0
43 14 E	2748.63852	4	1
43 15 E	2883.59274		2
43 16 A_1	3027.88087	14	3
43 16 A_2	3027.88087	14	3
43 17 E	3181.47226	7	1
44 0 E	1890.38176	11	-1
44 1 A_1	1892.10800		9
44 1 A_2	1893.06043		-2
44 1 E	1897.75473	15	2
44 2 E	1904.33724	4	2
44 3 E	1925.61410	19	-3
44 3 E	1941.13223	10	3
44 4 A_1	1956.44035	4	2
44 4 A_2	1956.44035	4	2
44 4 E	1977.13200	12	-1
44 5 E	1996.80532	4	1
44 5 A_2	2022.66324	10	-2
44 5 A_1	2022.66324	10	-2
44 6 E	2046.69975	8	-2
44 6 E	2077.71607	14	-5
44 7 A_2	2106.11288	9	-2
44 7 A_1	2106.11288	9	-2
44 7 E	2142.27861	14	-8
44 8 E	2175.03207	3	3
44 8 A_2	2216.33695	9	-5
44 8 A_1	2216.33695	9	-5
44 9 E	2253.44250	14	-2
44 9 E	2299.87502	3	0
44 10 A_1	2341.32775	2	4
44 10 A_2	2341.32775	2	4
44 10 E	2392.87467	16	-4
44 11 E	2438.66896	6	2

J K Γ	E^{exp}	Δ	δ
1	2	3	4
44 11 A_1	2495.31599	8	3
44 11 A_2	2495.31599	8	3
44 12 E	2545.44558		2
44 12 E	2607.17668		0
44 13 A_1	2661.63489	4	0
44 13 A_2	2661.63489	4	0
44 14 E	2787.21229	12	0
44 16 A_1	3066.42266		-6
44 16 A_2	3066.42266		-6
45 0 E	1929.91854	4	0
45 1 A_2	1932.62093		0
45 1 A_1	1931.62553	2	0
45 1 E	1937.29022	6	0
45 2 E	1943.87732	4	-3
45 2 A_1	1954.20261	14	0
45 2 A_2	1954.20540	10	7
45 3 E	1965.15188	5	-1
45 3 E	1980.66046	9	1
45 4 A_2	1995.97500	5	0
45 4 A_1	1995.97500	5	0
45 4 E	2016.65464	7	-5
45 5 E	2036.33582	2	-1
45 5 A_2	2062.17911	6	-1
45 5 A_1	2062.17911	6	-1
45 6 E	2086.22506	15	8
45 6 E	2117.22394	-	1
45 7 A_1	2145.63165	12	0
45 7 A_2	2145.63165	12	0
45 7 E	2181.77717	13	-5
45 8 E	2214.54315	4	1
45 8 A_2	2255.82498	14	-4
45 8 A_1	2255.82498	14	-4
45 9 E	2292.94483	6	2
45 9 E	2339.35131	2	0
45 10 A_1	2380.82008		7
45 10 A_2	2380.82008		7
45 10 E	2432.33804	5	2
45 11 E	2478.15012	5	2
45 11 A_2	2534.76508		2
45 11 A_1	2534.76508		2
45 12 E	2584.91444	5	4
45 12 E	2646.61036	2	4
45 13 A_2	2701.09033	11	5
45 13 A_1	2701.09033	11	5
45 14 E	2826.65311	2	4
45 15 E	2961.57618		3
45 16 A_2	3105.83096		7
45 16 A_1	3105.83096		7
46 0 E	1970.32366	2	-1
46 1 A_1	1972.01118	1	0
46 1 A_2	1973.05035		0
46 1 E	1977.69411	5	1
46 2 E	1984.28604	6	0
46 2 A_1	1994.60643	13	6
46 2 A_2	1994.60325	13	-6
46 3 E	2005.55810	10	-2
46 3 E	2021.05688	7	-4
46 4 A_1	2036.37805	8	-3
46 4 A_2	2036.37805	8	-3
46 4 E	2057.04546	19	-3
46 5 E	2076.73463	6	-2
46 5 A_1	2102.56289	10	-5
46 5 A_2	2102.56289	10	-5
46 6 E	2126.61839	9	0
46 7 A_1	2186.01845	6	1
46 7 A_2	2186.01845	6	1
46 7 E	2222.14332		-3
46 8 E	2254.92212	6	0
46 8 A_1	2296.18036	10	-5
46 8 A_2	2296.18036	10	-5
46 9 E	2333.31482	9	4
46 9 E	2379.69472	6	3
46 10 A_1	2421.17978	8	0
46 10 A_2	2421.17978	8	0
46 10 E	2472.66816	4	2
46 11 A_1	2575.08070	10	4
46 11 A_2	2575.08070	10	4
46 12 E	2625.25022	8	2
46 12 E	2686.91002	24	-10
46 13 A_1	2741.41236	5	4
46 13 A_2	2741.41236	5	4
46 14 E	2866.96021	6	2
46 15 E	3001.86720	7	2
46 16 A_1	3146.10466	20	-1
46 16 A_2	3146.10466	20	-1
47 0 E	2011.59649	2	0
47 1 A_1	2014.34802		-1
47 1 A_2	2013.26419		2
47 1 E	2018.96569	5	1
47 2 E	2025.56260	17	-4
47 2 A_1	2035.87162		-1
47 2 A_2	2035.87512	16	6
47 3 E	2046.83219	3	1
47 3 E	2062.32093	6	-2
47 4 A_1	2077.64888	10	-2
47 4 A_2	2077.64888	10	-2
47 4 E	2098.30367	9	-2
47 5 E	2118.00111	4	-1
47 5 A_1	2143.81402	7	-4
47 5 A_2	2143.81402	7	-4
47 6 E	2167.87929	7	-2
47 6 E	2198.84225	13	0
47 7 A_1	2227.27263	3	2
47 7 A_2	2227.27263	3	2
47 7 E	2263.37632	16	-6
47 8 E	2296.16835	7	4
47 8 A_1	2337.40242	8	1
47 8 A_2	2337.40242	8	1
47 9 E	2374.55177	3	0
47 9 E	2420.90448		-2
47 10 A_1	2462.40640	15	5
47 10 A_2	2462.40640	15	5

Continued

J K Γ	E^{exp}	Δ	δ
1	2	3	4
47 10 E	2513.86445	7	4
47 11 A_1	2616.26216	17	8
47 11 A_2	2616.26216	17	8
47 12 E	2666.45230	7	1
47 12 E	2728.07545	13	5
47 13 A_1	2782.60038	6	2
47 13 A_2	2782.60038	6	2
47 14 E	2908.13296		-2
47 16 A_1	3187.24350	8	8
47 16 A_2	3187.24350	8	8
48 0 E	2053.73627	11	-3
48 1 A_1	2055.38382	3	-2
48 1 A_2	2056.51327		-2
48 1 E	2061.10427	5	1
48 2 E	2067.70662	8	6
48 2 A_1	2078.01076	18	5
48 2 A_2	2078.00690	2	0
48 3 E	2088.97338	11	2
48 3 E	2104.45186	15	2
48 4 A_1	2119.78674	8	-2
48 4 A_2	2119.78674	8	-2
48 4 E	2140.42866	21	0
48 5 E	2160.13454	5	0
48 5 A_1	2185.93181		4
48 5 A_2	2185.93181		4
48 6 E	2210.00705	13	-2
48 6 E	2240.95145	14	6
48 7 A_1	2269.39348	6	1
48 7 A_2	2269.39348	6	1
48 7 E	2305.47561	14	-3
48 8 E	2338.28110	10	7
48 8 A_1	2379.49048	9	-6
48 8 A_2	2379.49048	9	-6
48 9 E	2416.65511		2
48 9 E	2462.98008	4	2
48 10 A_1	2504.49905	7	2
48 10 A_2	2504.49905	7	2
48 10 E	2555.92614		1
48 11 A_1	2658.30869		3
48 11 A_2	2658.30869		3
48 12 E	2708.51998	5	1
48 12 E	2770.10535		-14
48 13 A_1	2824.65368	10	-2
48 13 A_2	2824.65368	10	-2
48 14 E	2950.17077		1
48 16 A_1	3229.24651		8
48 16 A_2	3229.24651		8
49 0 E	2096.74241	4	1
49 1 A_1	2099.54541		0
49 1 A_2	2098.36947	1	0
49 1 E	2104.10915	7	0
49 2 E	2110.71677	13	-3
49 2 A_1	2121.00839	13	-1
49 2 A_2	2121.01265	12	2
49 3 E	2131.98097	6	0
49 3 E	2147.44886	15	-3
49 4 A_1	2162.79098	9	0
49 4 A_2	2162.79098	9	0
49 4 E	2183.41964	12	-4
49 5 E	2203.13421	5	0
49 5 A_1	2228.91534	15	-5
49 5 A_2	2228.91534	15	-5
49 6 E	2253.00105	21	8
49 6 E	2283.92626	5	-1
49 7 A_1	2312.38032	7	0
49 7 A_2	2312.38032	7	0
49 7 E	2348.44038	11	-5
49 8 E	2381.25957	4	1
49 8 A_1	2422.44387	9	-3
49 8 A_2	2422.44387	9	-3
49 9 E	2459.62407		3
49 9 E	2505.92069	8	4
49 10 A_1	2547.45713	5	1
49 10 A_2	2547.45713	5	1
49 11 A_1	2701.21966		-1
49 11 A_2	2701.21966		-1
49 12 E	2751.45257		2
49 13 A_1	2867.57169	11	5
49 13 A_2	2867.57169	11	5
49 14 E	2993.07289		5
50 0 E	2140.61409	7	1

J K Γ	E^{exp}	Δ	δ
1	2	3	4
50 1 A_1	2142.22037		2
50 1 A_2	2143.44369		0
50 1 E	2147.97957		-4
50 2 E	2154.59292	12	-2
50 2 A_1	2164.88008		0
50 2 A_2	2164.87548	6	4
50 3 E	2175.85432	0	2
50 3 E	2191.31140	13	0
50 4 A_1	2206.66083	1	0
50 4 A_2	2206.66083	1	0
50 4 E	2227.27600	8	-2
50 5 E	2246.99945	5	2
50 5 A_1	2272.76415	8	-4
50 5 A_2	2272.76415	8	-4
50 6 E	2296.86029	6	1
50 7 A_1	2356.23244	6	-1
50 7 A_2	2356.23244	6	-1
50 7 E	2392.26996	17	-6
50 8 E	2425.10326		5
50 8 A_1	2466.26189	11	5
50 8 A_2	2466.26189	11	5
50 9 E	2503.45792	7	2
50 9 E	2549.72558	5	1
50 10 A_1	2591.27993	3	2
50 10 A_2	2591.27993	3	2
50 10 E	2642.64313	-	0
50 11 A_1	2744.99438	6	-4
50 11 A_2	2744.99438	6	-4
50 12 E	2795.24932		0
50 13 A_1	2911.35345	15	-3
50 13 A_2	2911.35345	15	-3
50 14 E	3036.83845	10	-1
51 0 E	2185.35056	3	-2
51 1 A_1	2188.20740		0
51 1 A_2	2186.93576	5	1
51 1 E	2192.71492	5	-1
51 2 E	2199.33419	4	2
51 2 A_1	2209.60731	7	4
51 2 A_2	2209.61226	12	-8
51 3 E	2220.59262	1	0
51 3 E	2236.03861	14	0
51 4 A_1	2251.39564	8	5
51 4 A_2	2251.39564	8	5
51 4 E	2271.99694	4	-2
51 5 E	2291.72943	10	-2
51 5 A_1	2317.47741	10	-3
51 5 A_2	2317.47741	10	-3
51 6 E	2341.58426	9	-2
51 6 E	2372.47026	6	-5
51 7 E	2436.96374	8	5
51 8 E	2469.81117	10	-6
51 8 A_1	2510.94366	1	3
51 8 A_2	2510.94366	1	6
51 9 E	2548.15597	5	2
51 9 E	2594.39404	23	-4
51 10 A_1	2635.96668		2
51 10 A_2	2635.96668		2
51 10 E	2687.29703		6
51 11 A_1	2789.63222		5
51 11 A_2	2789.63222		5
51 12 E	2839.90958	15	4
51 13 A_1	2955.99846	6	-2
51 13 A_2	2955.99846	6	-2
52 0 E	2230.95117	10	0
52 1 A_1	2232.51492		-1
52 1 A_2	2233.83576		-1
52 1 E	2238.31434	9	-1
52 2 E	2244.93973	13	-2
52 2 A_1	2255.20860	28	-5
52 2 A_2	2255.20320		5
52 3 E	2281.62979	13	-1
52 4 A_1	2296.99450	7	-1
52 4 A_2	2296.99450	7	-1
52 4 E	2317.58174	10	-1
52 5 E	2337.32353	5	0
52 5 A_1	2363.05440	15	0
52 5 A_2	2363.05440	15	0
52 6 E	2387.17227	10	4
52 6 E	2418.03798	11	-2
52 7 A_1	2446.52962	11	2
52 7 A_2	2446.52962	11	2

J K Γ	E^{exp}	Δ	δ
1	2	3	4
52 7 E	2482.52068	3	-2
52 8 E	2515.38291	2	2
52 8 A_1	2556.48851	9	-2
52 8 A_2	2556.48851	9	-2
52 9 E	2639.92546	4	2
52 10 A_1	2681.51664		0
52 10 A_2	2681.51664		0
52 10 E	2732.81341	1	4
53 0 E	2277.41506	6	-2
53 1 A_1	2280.32809		3
53 1 A_2	2278.95713	10	0
53 1 E	2284.77713	5	2
53 2 E	2291.40890	8	0
53 2 A_1	2301.66238	8	5
53 2 A_2	2301.66825	7	-1
53 3 E	2312.66125	5	2
53 3 E	2328.08420	8	1
53 4 E	2364.02968		4
53 5 E	2383.78100	11	8
53 5 A_1	2409.49430	12	0
53 5 A_2	2409.49430	12	0
53 6 E	2433.62334	6	-2
53 6 E	2464.46845	10	-2
53 7 A_1	2492.97304	10	-8
53 7 A_2	2492.97304	10	-8
53 7 E	2528.94028	18	0
53 8 E	2561.81740	3	-2
53 8 A_1	2602.89576	5	-1
53 8 A_2	2602.89576	5	-1
53 10 A_1	2727.92912		3
53 10 A_2	2727.92912		3
53 12 E	2931.81736	23	-2
54 0 E	2324.74149	27	-3
54 1 A_1	2326.26160	9	3
54 1 A_2	2327.68348		0
54 1 E	2332.10245	18	1
54 2 E	2338.74088	14	0
54 2 A_1	2348.99037		0
54 2 A_2	2348.98398	28	-4
54 3 E	2359.99009		10
54 3 E	2375.40101	5	0
54 4 A_1	2390.78178	10	-1
54 4 A_2	2390.78178	10	0
54 4 E	2411.33979	5	-5
54 5 E	2431.10086	7	2
54 5 A_1	2456.79630	10	-7
54 5 A_2	2456.79630	10	-7
54 6 E	2480.93691	5	0
54 7 A_1	2540.27888	4	-2
54 7 A_2	2540.27888	4	-2
54 7 E	2576.22156		-10
54 8 E	2609.11408	5	3
54 8 A_1	2650.16449	16	-9
54 8 A_2	2650.16449	16	-9
54 9 E	2687.42771	4	1
54 9 E	2733.57372	-	6
54 10 A_1	2775.20319	19	-3
54 10 A_2	2775.20319	19	-3
55 0 E	2372.92976	1	4
55 1 A_1	2375.90120		-5
55 1 A_2	2374.42746		0
55 1 E	2380.28953	8	0
55 2 E	2386.93485	7	-3
55 2 A_1	2397.16750	7	5
55 2 A_2	2397.17417	10	-3
55 3 E	2408.18062	17	-9
55 3 E	2423.57949	2	2
55 4 A_1	2438.96857	6	-1
55 4 A_2	2438.96857	6	-1
55 4 E	2459.51151		-5
55 5 A_1	2504.95980	16	-3
55 5 A_2	2504.95980	16	-3
55 6 E	2529.11215	15	7
55 7 A_1	2588.44612	13	-5
55 7 A_2	2588.44612	13	-5
55 7 E	2624.36405		1
55 8 E	2657.27202		2
55 8 A_1	2698.29419		2
55 8 A_2	2698.29419		2
55 9 E	2781.68903		8
55 10 A_1	2823.33820	8	-6

J K Γ	E^{exp}	Δ	δ
1	2	3	4
55 10 A_2	2823.33820	8	-6
55 10 E	2874.53048		18
55 11 A_1	2976.79809		3
55 11 A_2	2976.79809		3
56 0 E	2421.97874	5	-10
56 1 A_1	2423.45403	5	2
56 1 A_2	2424.98060		5
56 1 E	2429.33758	2	0
56 2 A_1	2446.21897	7	4
56 2 A_2	2446.21184	5	4
56 3 E	2457.23245		-3
56 3 E	2472.61871	9	-5
56 4 A_1	2488.01641	5	0
56 4 A_2	2488.01641	5	0
56 4 E	2508.54395		-4
56 5 A_1	2553.98382	13	-3
56 5 A_2	2553.98382	13	-3
56 6 E	2578.14803	10	-3
56 6 E	2608.92865	26	-3
56 7 E	2673.36666		3
56 10 A_1	2872.33334	21	-6
56 10 A_2	2872.33334	21	-6
56 10 E	2923.48942		20
56 11 A_1	3025.73948		14
56 11 A_2	3025.73948		14
57 0 E	2471.88806	5	-1
57 1 A_1	2474.92060	9	4
57 1 A_2	2473.34032		-7
57 1 E	2479.24580	7	3
57 2 E	2485.90575	3	1
57 2 A_2	2496.12371	5	-2
57 3 E	2507.14462	7	3
57 3 E	2522.51804	18	-2
57 4 A_1	2537.92445		0
57 4 A_2	2537.92445		0
57 4 E	2558.43629	5	-4
57 5 E	2578.22785	15	3
57 5 A_1	2603.86767	8	4
57 5 A_2	2603.86767	8	4
57 6 E	2628.04401	16	-3
57 7 A_1	2687.36186	6	-4
57 7 A_2	2687.36186	6	-4
57 7 E	2723.22861	1	1
57 8 A_1	2797.13242		-4
57 8 A_2	2797.13242		-4
57 9 E	2834.44940	6	-4
57 9 E	2880.49789		0
57 10 A_1	2922.18779		-3
57 10 A_2	2922.18779		-3
57 11 E	3019.36521		7
57 11 A_1	3075.53900		6
57 11 A_2	3075.53900		6
58 2 E	2536.68094	12	-2
58 2 A_1	2546.88772	2	0
58 2 A_2	2546.88001		-1
58 3 E	2573.27649	22	-5
58 4 A_1	2588.69188	13	0
58 4 A_2	2588.69188	13	0
58 4 E	2609.18767	28	-5
58 5 E	2628.98984		1
58 6 E	2678.79924	12	5
58 6 E	2709.53474	12	0
58 7 A_1	2738.10865	5	-7
58 7 A_2	2738.10865	5	-7
58 8 E	2806.90556	-	-3
58 8 A_1	2847.83950	2	-1
58 8 A_2	2847.83950	2	-1
58 9 E	2885.17510		-2
58 10 A_1	2972.90067		-2
58 10 A_2	2972.90067		-2
58 11 E	3070.06378		-1
59 0 E	2574.28352		3
59 1 A_1	2577.37936		-1
59 1 A_2	2575.68934	6	5
59 1 E	2581.63925		6
59 2 E	2588.31491		0
59 2 A_1	2598.50227		5
59 4 A_1	2640.31786		1
59 4 A_2	2640.31786		1
59 4 E	2660.79734		1
59 5 E	2680.61027	2	0

J K Γ	E^{exp}	Δ	δ
1	2	3	4
59 5 A_1	2706.21114		5
59 5 A_2	2706.21114		5
59 6 E	2761.12506		2
59 7 A_1	2789.71366		-5
59 7 A_2	2789.71366		-5
59 8 E	2858.50059		0
59 8 A_1	2899.40400		-3
59 8 A_2	2899.40400		-3
59 9 E	2936.75857		-4
60 1 A_1	2628.15023		12
60 2 E	2640.80675		1
60 3 E	2677.36765	6	5
60 4 A_1	2692.80152	4	3
60 4 A_2	2692.80152	4	3
60 5 E	2733.08827		-1
60 5 A_1	2758.66905		-1
60 5 A_2	2758.66905		-1
60 7 A_1	2842.17591		-10
60 7 A_2	2842.17591		-10
60 8 E	2910.95275	15	1
60 8 A_1	2951.82521		5
60 8 A_2	2951.82521		5
61 1 A_2	2681.46735	10	2
61 2 E	2694.15559		2
61 3 E	2715.37914	12	-4
61 4 A_1	2746.14189		-3
61 4 A_2	2746.14189		-3
61 4 E	2766.58762		-7
61 5 E	2786.42299		0
61 6 E	2836.21107	5	0
61 7 A_1	2895.49474	8	-1
61 7 A_2	2895.49474	8	-1
61 8 E	2964.26104		-11
61 10 A_1	3130.18157	15	15
61 10 A_2	3130.18157	15	15
62 0 E	2734.30599		5
62 1 A_1	2735.64011		5
62 1 A_2	2737.50156		3
62 1 E	2741.65895		0
62 2 E	2748.36050		-1
62 3 E	2769.58005	10	3
62 3 E	2784.88490		-5
62 4 A_1	2800.33829		4
62 4 A_2	2800.33829		3
62 5 E	2840.61345		-5
62 5 A_1	2866.15302	7	-5
62 5 A_2	2866.15302	7	-5
62 6 E	2890.39422	10	-2
62 7 A_1	2949.66899		-3
62 7 A_2	2949.66899		-3
62 9 E	3096.64723		-5
62 10 A_1	3184.31950		5
62 10 A_2	3184.31950		5
63 1 A_2	2790.66740		-1
63 2 E	2803.42063		-2
63 4 A_1	2855.38956	23	-2
63 4 A_2	2855.38956	23	-3
63 5 A_1	2921.17716		-14
63 5 A_2	2921.17716		-14
63 6 E	2945.43219		1
64 0 E	2845.26330		0
64 1 A_1	2846.54834		-9
64 1 E	2852.61478		5
64 3 E	2880.54602		0
64 4 A_1	2911.29498	3	0
64 4 A_2	2911.29498	3	0
64 5 E	2951.55829		2
64 7 A_1	3060.58056		3
64 7 A_2	3060.58056		3
65 1 E	2909.37254		-4
65 4 A_2	2968.05361		9
65 4 A_1	2968.05361		9
66 4 A_1	3025.66416		-6
66 4 A_2	3025.66416		-6
67 4 A_1	3084.12616		1
67 4 A_2	3084.12616		1
68 4 A_1	3143.43826		-3
68 4 A_2	3143.43826		-3

[a] In Table, Δ is the experimental uncertainty of the energy value, equal to one standard uncertainty in units of 10^{-5} cm^{-1}; δ is the difference, $E^{\mathrm{exp}} - E^{\mathrm{calc}}$ also in units of 10^{-5} cm^{-1}. When the $\Delta-$value is absent, the corresponding energy level was obtained from a single transition.
